# Transcriptome analysis of amoeboid and ramified microglia isolated from the corpus callosum of rat brain

**DOI:** 10.1186/1471-2202-13-64

**Published:** 2012-06-14

**Authors:** Rangarajan Parakalan, Boran Jiang, Baby Nimmi, Manivannan Janani, Manikandan Jayapal, Jia Lu, Samuel SW Tay, Eng-Ang Ling, S Thameem. Dheen

**Affiliations:** 1 Department of Anatomy, Yong Loo Lin School of Medicine, National University of Singapore, Blk MD10, 4 Medical Drive, Singapore, 117597, Singapore; 2 Center of Excellence in Genomic Medicine Research, King Fahd Medical Research Center, King Abdulaziz University, PO Box 80216, Jeddah, 21589, Kingdom of Saudi Arabia

## Abstract

**Background:**

Microglia, the resident immune cells of the central nervous system (CNS), have two distinct phenotypes in the developing brain: amoeboid form, known to be amoeboid microglial cells (AMC) and ramified form, known to be ramified microglial cells (RMC). The AMC are characterized by being proliferative, phagocytic and migratory whereas the RMC are quiescent and exhibit a slow turnover rate. The AMC transform into RMC with advancing age, and this transformation is indicative of the gradual shift in the microglial functions. Both AMC and RMC respond to CNS inflammation, and they become hypertrophic when activated by trauma, infection or neurodegenerative stimuli. The molecular mechanisms and functional significance of morphological transformation of microglia during normal development and in disease conditions is not clear. It is hypothesized that AMC and RMC are functionally regulated by a specific set of genes encoding various signaling molecules and transcription factors.

**Results:**

To address this, we carried out cDNA microarray analysis using lectin-labeled AMC and RMC isolated from frozen tissue sections of the corpus callosum of 5-day and 4-week old rat brain respectively, by laser capture microdissection. The global gene expression profiles of both microglial phenotypes were compared and the differentially expressed genes in AMC and RMC were clustered based on their functional annotations. This genome wide comparative analysis identified genes that are specific to AMC and RMC.

**Conclusions:**

The novel and specific molecules identified from the trancriptome explains the quiescent state functioning of microglia in its two distinct morphological states.

## Background

Microglia are the prime immune effector cells of the central nervous system (CNS). The origin, morphology and role of microglia in health and disease were first elaborately described in 1939 [1]. The amoeboid microglial cells (AMC), which are abundant in the periventricular white matter, namely the corpus callosum (CC) of the brain function as macrophages in the developing brain. Studies have demonstrated that AMC gradually transform into ramified microglial cells (RMC) with advancing age [2].

The time course of development of microglia differs in different regions of the brain [3]. In the CC, AMC preponderate a week before birth in mice and rats [4-6] and actively phagocytose the cellular debris and refine axonal connectivity during the first postnatal week [7-9]. This is followed by the gradual transition into RMC, which survey the brain parenchyma with their fine and non-overlapping ramifications, thereby monitoring chronic and acute insults [10]. Upon activation by trauma, infection or any other neurodegenerative stimuli, microglia retract their ramifications, transform into amoeboid or spherical shape, produce pro-inflammatory cytokines and display phagocytosis [11]. These microglia are known to be activated or reactive.

Studies in chronic, aging associated neuropathologies such as Alzheimer’s disease (AD) [12-14], and Parkinson’s disease (PD) [15] indicate persistent microglial activation as the major causative factor in disease exacerbation. Aging brains are often characterized by the presence of primed microglia, which present an altered cytokine profile in comparison to their counterparts in younger brains [16,17]. These microglia produce an exaggerated inflammatory response when activated [18,19] leading to prolonged cycles of proliferation and production of pro-inflammatory cytokines which eventually render them neurotoxic. Further, chronic microglial activation has been shown to cause the impairment of adult neurogenesis in hippocampus [20] and damage to the periventricular white matter (PWMD) in the early postnatal brain [21]. Hence activated microglia in both postnatal and adult stages can have neurotoxic effects on the CNS by causing excessive inflammation. Identification of ways to attenuate microglia-mediated neuroinflammation, therefore, has been the primary consideration in therapeutic strategy. There is accumulated information on the factors that contribute to the activation, migration, proliferation and immune response of microglia over the years [22,23], but the gene expression and signaling networks that function within these cells are yet to be fully clarified.

Gene expression profiles of microglia from primary cultures are available, but their expression profiles have been found to be altered once isolated from their natural milieu [24]. It is striking that investigation on the expression profiles of functioning genes of AMC and RMC *in vivo* in their quiescent state have remained elusive. In this connection, we carried out a global gene expression profiling of AMC and RMC *in situ* by isolating them from the CC of rat brain using laser-capture microdissection (LCM) tool. Overlapping the transcriptome onto several online and commercial databases, our current study aimed to identify molecular candidates that are associated with the morphological transformation and physiological functioning of microglia in the developing brain. Further, we have identified the genes that render ‘stemness’ and ‘monocytic’ functions to AMC and RMC. The transcriptome profiling has also led to the identification of several genes that may be vital in regulating microglial proliferation, differentiation, migration, and ramification.

## Methods

### Ethics statement

In the handling and care of animals, the International Guiding Principles for Animals Research, as adopted by the Institutional Animal Care and Use Committee (IACUC), National University of Singapore, were followed. All efforts were made to minimize pain and the number of rats used.

### Laser-capture microdissection (LCM)

Whole fresh brains were removed from 5-day postnatal Wistar rat pups (n = 3) and 4-week old Wistar rats (n = 3) and placed in liquid nitrogen immediately for a short time and then frozen in a cryostat (Model No. CM 3050 S, Leica Microsystems GmbH, Wetzlar, Germany). The forebrain was sectioned coronally through the CC at 5 μm thickness and mounted on precleaned slides. The sections were fixed in 75% ethanol for 1 min and incubated with peroxidase conjugated isolectin (1: 50, Cat. No. L5391, Sigma-Aldrich Co., MO,USA) for 15 min. The sections were then dehydrated by a graded series of ethanol and cleaned in xylene. The slide was placed on the microscope stage of MMI CellCut (Molecular Machines & Industries, Glattbrugg, Switzerland). The 4 X, 10 X to 40 X objective lenses were used to achieve the proper placement of the cap (for cell collection) above the CC. Lectin stained microglia cells (AMC from 5-day and RMC from 4-week old rat brain CC) were selected and cut by laser and collected into the cap of tube (Cat No. 50202, Molecular Machines &Industries, Glattbrugg, Switzerland). Extra care was taken to minimize the contamination of materials from other cell types while laser dissecting microglia from the CC.

### Microarray analysis

Total RNA was extracted from 600 isolated microglia cells per group using RNeasy micro kit (Cat. No. 74004, Qiagen, CA, USA), quantified by Nanodrop 1000 (Thermo Scientific, MA, USA) and hybridized to each microarray chip. RNA (15 ng) was reverse transcripted into the first-strand cDNA using a T7-Oligo (dT) Primer (Two-Cycle Target Labeling and Control Reagent package, Affymetrix, CA, USA). After second-strand cDNA synthesis, the double-stranded cDNA was purified and served as a template in the first cycle of *in vitro* transcription (IVT) reaction. The unlabeled cRNA was then reverse transcripted into the first-strand cDNA of the second cycle using random primers. Subsequently, the T7-Oligo(dT) Promoter Primer was used in the second-strand cDNA synthesis to generate double-stranded cDNA template containing T7 promoter sequences. Then the double-stranded cDNA was amplified and labeled using a biotinylated nucleotide analog/ribonucleotide mix in the second IVT reaction. The labeled cRNA was then cleaned up, fragmented, and hybridized to Rat Genome 230 2.0 Array (Cat. No.900506, Affymetrix, CA, USA). A total of six arrays (three each for AMC and RMC) were carried out in the present study. The arrays were stained according to the manufacturer’s protocols and then scanned with the Genechip scanner (Affymetrix, CA, USA). Initial analysis of the scanned images was performed by GeneChip Operating Software (GCOS, Affymetrix, CA, USA). For absolute analysis, each chip was normalized to a target intensity of 500, and probe sets were assigned a signal intensity and detection call of Present, Marginal or Absent.

### Data analysis and generation of gene lists

The absolute data (signal intensity, detection call and detection p-value) were exported into GeneSpring GX 7.3 software (Agilent Technologies, CA, USA). All the six chips were globally normalized and the genes of over 2-fold differential expression were filtered out and used for functional analysis.

### Data normalization and generation of gene lists using MATLAB

Raw CEL files of the six chips were RMA (Robust Multichip Average) normalized using the Affymetrix Expression Console Version 1.1 (Affymetrix, CA, USA). The normalized data was then used to identify differentially expressed genes between AMC and RMC in MATLAB R2009a (MathWorks, MA, USA). For the statistical analysis, we used the ‘Exploring Gene Expression Data’ demo scripts in the Bioinformatics Toolbox^™^. The data was filtered for removing genes with low expression values and low variance across chips. Further, t-test was performed to retain genes with p-values less than 0.05 and a Volcano Plot was generated to identify the two-fold differentially expressed genes. The microarray data discussed in this publication is MIAME compliant and has been deposited in NCBIs Gene Expression Omnibus (GEO, http://www.ncbi.nlm.nih.gov/geo/). It is accessible through GEO Series accession number GSE29885.

### Gene expression profile clustering and pathway analysis

Agglomerative average-linkage hierarchical clustering of the different experimental groups was obtained for selected groups of genes with GeneSpring GX 7.3 software (Agilent Technologies, CA, USA) with standard correlation used as the similarity matrix. The gene lists obtained was fed into Pathway Studio 6 software (Ariadne, MD, USA) to generate pathways for identifying interactions between the genes for validation purposes.

### Analysis of gene lists

The gene list generated from MATLAB was used to identify functional groups enriched in the AMC and RMC using DAVID Bioinformatics Database [25,26]. To identify the ‘Stemness’ of AMC and RMC, we compared our gene lists to gene lists enriched in embryonic, neural and hematopoietic stem cells [27]. Since the data were accumulated from a different microarray platform, we found orthologs to their genes pertaining to our platform using the online NetAffyx application (Affymetrix, CA, USA). For comparison of our gene expression data to that of peripheral blood monocytes [28], the raw CEL files of monocyte expression data were downloaded from NCBI GEO (Gene Expression Omnibus) and the orthologs pertaining to our platform were identified using the online NetAffyx application. These files were RMA normalized in Affymetrix Expression Console Version 1.1 (Affymetrix, CA, USA) and subsequently the average expression values of the monocyte genes were compared to our microglia gene lists.

### Double immunofluorescence staining on postnatal rat brain sections

5-day and 4-week old Wistar rat pups were purchased from the Laboratory Animal Centre, National University of Singapore. The animals were perfused and fixed with 4% paraformaldehyde for further procedure. For double immunofluorescence staining, forebrain sections at 30μm were cut through the corpus callosum using cryostat (Model No. CM 3050 S, Leica Microsystems GmbH, Wetzlar, Germany). The sections were incubated with purified mouse anti-OX-42 Ig (1:50; Cat No. CBL1512, Millipore, MA,USA) along with rabbit anti-ETO (1:100; Cat No. sc-28693, Santa Cruz Biotechnology, Inc. CA, USA) or with rabbit anti-Dcx (1:100, Cat No. ab18723, abcam, Cambridge, UK) or with rabbit anti-Sox4 (1:100; Cat No.sc-20090,Santa Cruz Biotechnology, Inc. CA, USA) or with rabbit anti-Sox11 (1:100; Cat No.sc-20096 ,Santa Cruz Biotechnology, Inc. CA, USA) or with rabbit anti-Sept9 (1:100; Cat No. sc-130263, Santa Cruz Biotechnology, Inc. CA, USA) with rabbit anti-Sept4 (1:100; Cat No. sc-20179, Santa Cruz Biotechnology, Inc. CA, USA) overnight at 4°C. On the following day, the sections were further incubated with either FITC-conjugated goat-anti-mouse IgG (1:100; Cat No. F9137, Sigma-Aldrich Co., MO, USA) or Cy3-conjugated sheep-anti-rabbit IgG secondary antibody (1:100; Cat No. C2306, Sigma-Aldrich Co., MO, USA). The sections were counterstained with DAPI (1 μg/ml, Cat. No. D1306, Invitrogen, CA, USA) and mounted with a fluorescent mounting medium (DakoCytomation, Glostrop, Denmark). Photo-images were captured using a confocal microscope (Olympus FV1000, Tokyo, Japan).

### Cell culture

BV-2 cells (a widely used murine microglial cell line) were maintained at 75 cm^2^ culture flasks in Dulbecco’s Modified Eagle’s Medium (DMEM, Sigma, St. Louis, MO, USA; Cat. No. 1152) supplemented with 10% fetal bovine serum (FBS, HyClone, Logan, UT) and cultured in 37°C in a humidified atmosphere of 5% CO_2_ and 95% air incubator. Cells were seeded on 6-well plates at about a density of 1.0 × 10^6^ per well for RNA isolation.

### RNA isolation and real-time RT-PCR for validation of microarray data

Total RNA from laser-captured AMC and RMC was extracted using miRNeasy Mini Kit (Qiagen, Germany, Cat. No.217004) and RNA from BV-2 cells was extracted with RNeasy Mini Kit (Qiagen, Germany, Cat. No. 75161) according to the manufacturer’s instructions and quantified spectrophotometrically. 2 μg of RNA from each sample was added to a total volume of 25 μl reaction mixture containing 2.5 μM of oligo (dT) primer (Promega, Madison, WI USA; Cat. No. C110A), and 200U of Molony Murine Leukemia Virus Reverse Transcriptase (M-MLV, Promega, Madison, WI, USA; Cat. No. M5314). The reaction was initiated by incubating the reaction mixture for 1 h at 42°C for reverse transcription, and stopped by heating for 10 min at 70°C. Aliquot (0.5 μl) of the each reverse transcription product was added to the 10 μl reaction mixture containing QuantiTect^R^SYBR^R^ Green I, 0.5 μM of each primer corresponding to Runx1t1, Sept9, Sept4, Mbp (rat), Gapdh, Dcx, Mbp (mouse), or β-actin and 4 mM MgCl_2_ to amplify the genes in ABi 7900HT Fast PCR system (Applied Biosystems, USA). The primer sequences of Runx1t1 are forward: 5′-ACGAACAGCTGCTTCTGGAT-3′and reverse: 5′-TGCTTGGATGTTCTGAGTGC-3′, Sept 9 are forward: 5′-AACCATGTCCCTCGAACTTG-3′ and reverse: 5′-AAGAGAGAGGGGACACGACA-3′, Sept 4 are forward: 5′-CTCATCCGGGAGAAAGATGA-3′ and everse: 5′-GAGCTGATGCAGGGAAG-3′, Mbp are forward: 5′-TACTTGGCCACAGCAAGTACC-3′ and reverse: 5′-GGGTGTACGAGGTGTCACAAT-3′, Gapdh are forward: 5′-ACATGCCGCCTGGAGAAACCTGCCA-3′ and reverse: 5′-TGCCAGCCCCAGCATCAAAGGTGGA-3′. The primer sequences used for the data reported in the supplementary figure are listed in Additional file 1: Sheet S1. After pre-incubation at 95°C for 15 min, the polymerase chain reaction (PCR) was performed as follows: 45 cycles of denaturation at 94°C for 15 s, annealing at 57°C for 25 s, and elongation at 72°C for 15 s.

## Results

Laser-capture microdissection of microglial cells from the corpus callosum of 5-day and 4-week old rat brain.

**Figure 1:**
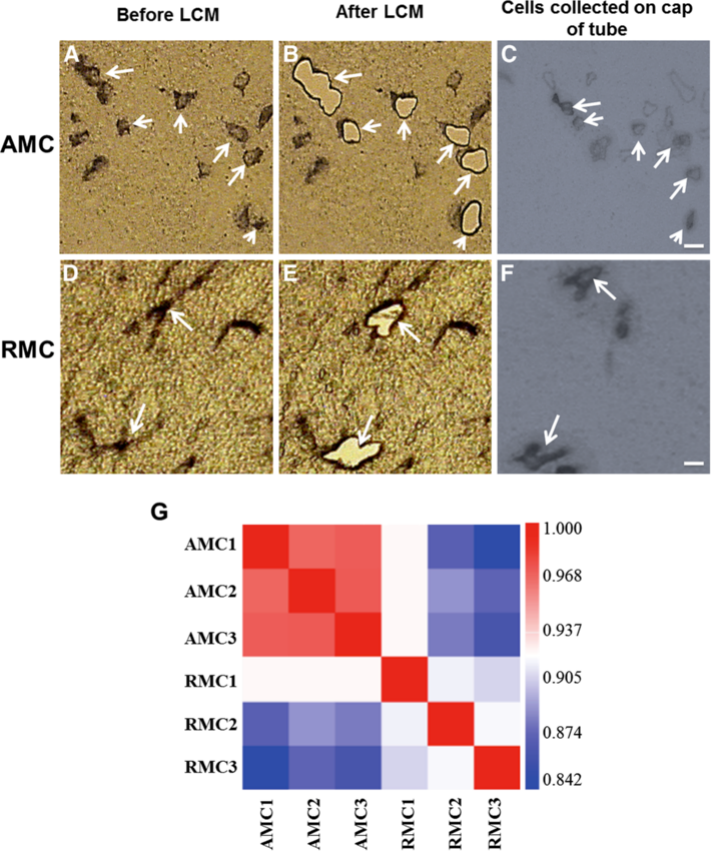
**A-F. Identification and isolation of amoeboid microglial cells (AMC) and ramified microglial cells (RMC) from the corpus callosum (CC) of 5-day old and 4-weeks old rat brain respectively.** Figure A shows AMC and Figure D shows RMC stained with lectin under laser capture microscopy. These cells are laser-cut along their periphery and isolated. Figure B and E show the region of the stained tissue section wherein the cells have been removed, and figure C and F show the isolated cells collected in the cap of vial. Arrows indicate the same cells in all the three images. Scale bars: A-F 50 μm. **G. Correlation plots**. Correlation plots were generated in Affymetrix Expression Console 1.1 after RMA normalizing raw CEL files of AMC and RMC expression data. The color scale indicates the degree of correlation between two different samples. A value of close to 1 refers to a high correlation.

To compare the gene expression profiles of AMC and RMC, we stained both microglial cell types with peroxidase-conjugated lectin and isolated them from the CC of 5-day and 4-week old rat brain respectively. LCM of AMC and RMC from the CC of 5-day old rat brain has been shown in Figure1A-F. Lectin staining has been widely used to selectively stain microglia for study of microglial development in the CNS [29-31]. The cells isolated by LCM were further confirmed to be microglia since the mRNA expression of oligodendrocyte (CNPase), astrocyte (GFAP) and endothelial cell-specific genes (Vimentin) was undetectable (data not shown).

### cDNA microarray and generation of gene lists specific to AMC and RMC

To identify the genes that are differentially expressed between AMC and RMC, we extracted total RNA from AMC and RMC and carried out cDNA microarray using Rat Genome 230 2.0 array (Affymetrix). Each sample contained RNA from six hundred laser-captured microglial cells. To ensure gene expression consistency between samples within the groups, we determined the Pearson’s rank correlation coefficient after normalizing the raw expression data (Figure1G). The gene expression profile from the samples of same group showed a very high correlation of 0.97 ± 0.03 while, a relatively lower correlation value of 0.87 ± 0.03 was observed between samples of different groups. A high correlation coefficient of above 0.8 between the AMC and RMC may be due to the fact that the comparison is between the gene expression profiles of the same cell type., i.e. microglia regardless of the differences in age (5-day and 4-week rat brain) and morphology (amoeboid and ramified).

**Figure 2:**
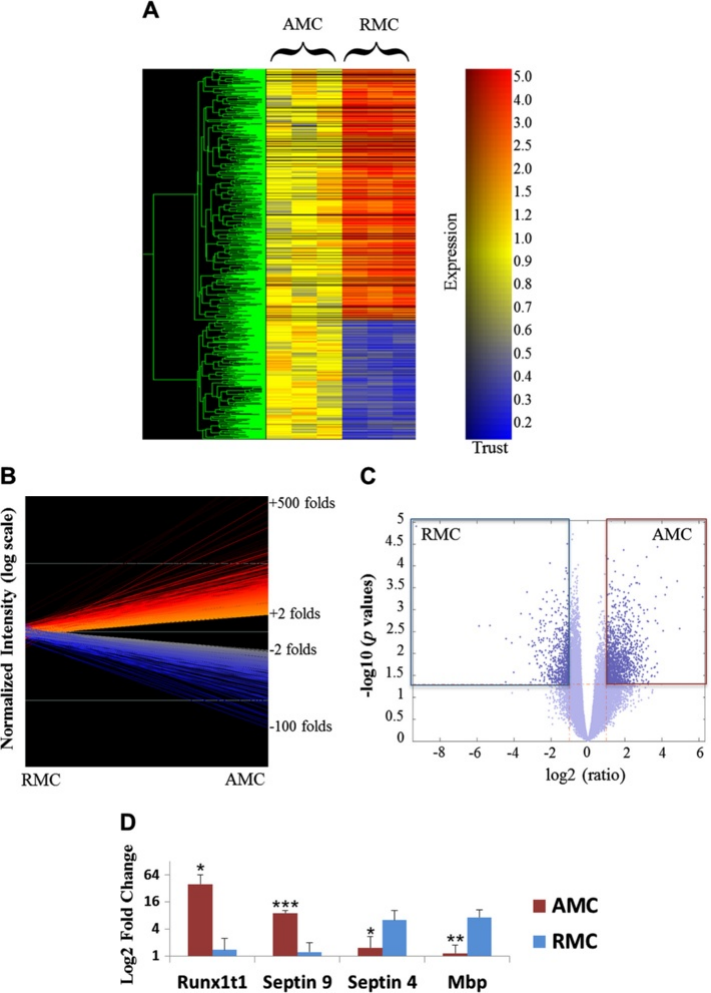
**A. Cluster Analysis.** Cluster analysis shows changes in gene expression profiles of AMC and RMC. Agglomerative average-linkage hierarchical clustering of the six independent samples was obtained for selected groups of genes using GeneSpring 7.3. Each colored box represents the normalized expression level of a given gene in each sample and is colored according to the fold change. **B. Line Graph.** Represents a two-fold differential gene expression between the AMC and RMC. The lines in red represent gens upregulated and those in blue represent genes downregulated in AMC in comparison to RMC. **C. Volcano Plot.** Within the lateral quadrants (red and blue box) are the genes with two-fold difference and P Value < 0.05. These genes were chosen for generation of functional group lists. **D.Validation of Microarray Profile.** Histogram shows the qRTPCR validation of two AMC-specific genes (Runx1t1 and Sept9) and two RMC-specific genes (Sept4 and Mbp).

Agglomerative average-linked hierarchical clustering was performed and genes showing over two-fold differential expression between AMC and RMC were identified using GeneSpring 7.3 (Figure2A & B). About 800 genes were found to be differentially regulated in the two sample groups - 537 with upregulation and 258 with downregulation in AMC. A high number of differentially expressed genes identified were either novel or did not have any functional annotation. In view of this, a list of genes with known functional annotations was generated using the statistical functions in the Bioinformatics Toolbox in MATLAB R2009a [32]. This list was generated using a less stringent filtering (*p* value < 0.05, in contrast to the GeneSpring list which has a *p* value < 0.01) and contained close to 1400 upregulated genes and 700 downregulated genes in AMC compared to RMC (Figure2C).

### Functional categorization of AMC and RMC

**Figure 3:**
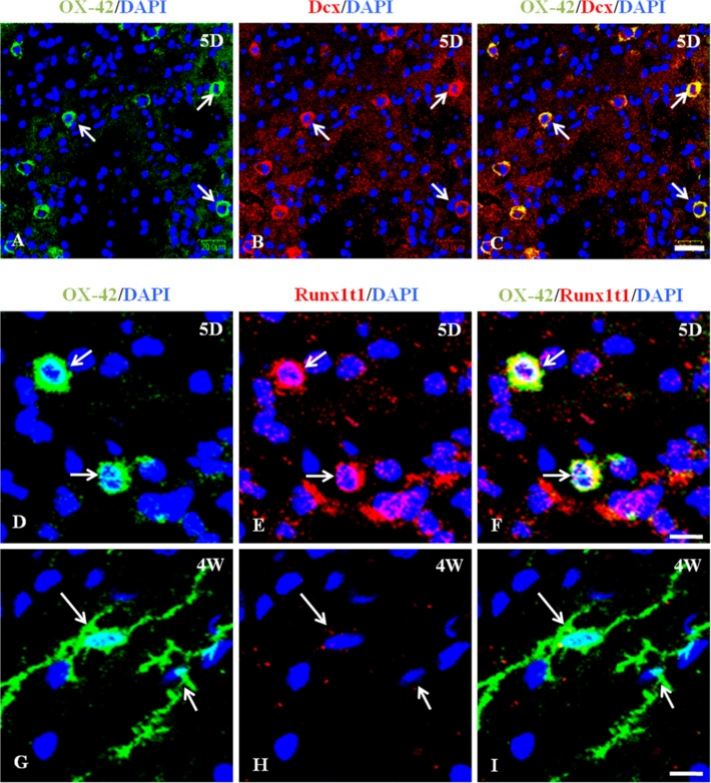
**A-F. Differential immunoexpression of Dcx andRunx1t1 in AMC and RMC.** Confocal images showing the immunoexpression of Dcx (B; red) and its co-localization (C) in OX42 (A; green) labeled AMC. Immunoexpression of Runx1t1 (E, H; red) and its co-localization (F, I) in OX42 (D, G; green) labeled AMC and RMC in the CC from 5-day (5D) and 4-week (4 W) old rat brain was also observed. Runx1t1 immunoexpression is undetectable in RMC (I) compared to that in the AMC (F). (DAPI – blue). Scale bars: A-C 50 μm, D-I 10 μm.

The genes with highest fold change values in AMC and RMC (Table1 and 2) clearly delineate their functions and residing environment. For example, the AMC express genes that are shown to be involved in nervous system development (such as Dpysl3, Crmp1 and Smarca1) [33,34], immune system development (such as Hmgb3 and Sla) [35-38], cell migration during neurodevelopment (Dcx) [39] and the immune response as well in migration of microglia (Cxcr4, a chemokine receptor) [40-42]. The finding of expression of some genes that are known to be neuron-specific (such as Dcx), is interesting and has been further confirmed by immunohistochemical analysis which revealed the expression of Dcx by the AMC in the CC of 5 day old rat brain (Figure3A-C). In addition, mRNA expression of Dcx was detected in the BV-2 microglia (a murine microglia cell line) by RTPCR (Additional file 2: Figure S1).

**Table 1:** Top 25 highly-expressed genes in AMC based on fold change. Both MATLAB and GeneSpring analysis are represented

	**Genes from MATLAB analysis**				**Genes from Genespring analysis**		
Gene Symbol	Gene Title	Fold Change	Function	Gene Symbol	Gene Title	Fold Change	Function
Sla	Src-like adaptor	73.34	protein binding	LRRGT00193	Unknown	574.79	Unknown
Dpysl3	dihydropyrimidinase-like 3	31.01	nervous system development	Rnf152	ring finger protein 152	35.04	Unknown
Sox4	SRY (sex determining region Y)-box 4	28.33	pro-B cell differentiation	Syncrip	Synaptotagmin binding, cytoplasmic RNA interacting protein	26.62	mRNA processing
Rprm	reprimo, TP53 dependent G2 arrest mediator candidate	19.13	cell cycle arrest	Chst8	carbohydrate (N- acetylgalactosamine 4–0) sulfotransferase 8	26.17	sulfur metabolic process
Satb2	SATB homeobox 2	18.05	negative regulation of transcription	Rpl28	ribosomal protein L28	19.32	translation
Dcx	Doublecortin	16.64	neuronmigration	Slc16a7	solute carrier family 16, member 7 (monocarboxylic acid transporter 2)	18.85	transport
Crmp1	collapsin response mediator protein 1	15.50	neuron development	Crmp1	collapsin response mediator protein 1	18.54	neuron development
Syt16	synaptotagmin XVI	13.53	protein binding	LOC100158225	hypothetical protein LOC100158225	17.44	Unknown
Cct8	chaperonin containing Tcp1, subunit 8 (theta)	13.53	protein folding	Bmp2	bone morphogenetic protein 2	16.43	ossification
RGD1310352	similar to HTGN29 protein; keratinocytes associated transmembrane protein 2	13.42	Unknown	Dcx	doublecortin	15.26	neuron migration
Appbp2	amyloid beta precursor protein (cytoplasmic tail) binding protein 2	13.28	Transport	Mab21l1	mab-21-like 1 (C. elegans)	14.54	positive regulation of cell proliferation
Cxcr4	chemokine (C-X-C motif) receptor 4	12.76	ameboidal cell migration	Hmgb3	high mobility group box 3	14.14	negative regulation of myeloid cell differentiation
Ect2	epithelial cell transforming sequence 2 oncogene	12.50	cell morphogenesis	Baz1a	bromodomain adjacent to zinc finger domain, 1A	13.89	protein binding
Hmgb3	high mobility group box 3	12.47	negative regulation ofB cell differentiation	Sox4	SRY (sex determining region Y)-box 4	13.43	pro-B cell differentiation
Hs3st5	heparan sulfate (glucosamine) 3-O-sulfotransferase 5	12.32	protein amino acid sulfation	LOC688455	hypothetical protein LOC688455	12.93	Unknown
Cfl2	cofilin 2, muscle	12.21	protein binding	Cxcr4	chemokine (C-X-C motif) receptor 4	12.92	ameboidal cell migration
Tnc	Tenascin C	11.93	negative regulation of cell adhesion	Spint2	serine peptidase inhibitor, Kunitz type, 2	12.49	serine-type endopeptidase inhibitor activity
Nap1l3	nucleosome assembly protein 1-like 3	10.88	nucleosome assembly	Zfml	zinc finger, matrin-like	12.20	nucleic acid binding
Mex3b	mex3 homolog B (C. elegans)	10.78	RNA binding	Nradd	neurotrophin receptor associated death domain	11.65	signal transduction
Smarca1	SWI/SNF related, matrix associated, actin dependent regulator of chromatin, subfamily a, member 1	10.72	brain development	Bcl7c	B-cell CLL/lymphoma 7 C	11.44	Unknown
Sh3bgrl	SH3 domain binding glutamic acid-rich protein like	9.74	Unknown	Tmeff1	transmembrane protein with EGF-like and two follistatin-like domains 1	11.43	multicellular organismal development
Ascc3l1	Activating signal cointegrator 1 complex subunit 3-like 1	9.58	RNA splicing	Mtus1	mitochondrial tumor suppressor 1	11.12	cell cycle
Bex4	brain expressed gene 4	9.50	Nucleus	Adfp	Adipose differentiation related protein	10.85	response to organic cyclic substance
Fam164a	family with sequence similarity 164, member A	9.49	Unknown	Ttk	Ttk protein kinase	10.65	protein amino acid fig7 phosphorylation
LOC294446	similar to Myristoylated alanine-rich C-kinase substrate (MARCKS) (ACAMP-81)	9.44	calmodulin binding	Lrrc20	leucine rich repeat containing 20	10.63	protein binding

**Table 2:** Top 25 genes in RMC based on fold change. Both MATLAB and GeneSpring analysis are represented

	**Genes from MATLAB analysis**				**Genes from Genespring analysis**		
Gene Symbol	Gene Title	Fold Change	Function	Gene Symbol	Gene Title	Fold Change	Function
Mobp	myelin-associated oligodendrocyte basic protein	623.18	nervous system development	Mog	myelin oligodendrocyte glycoprotein	26.34	cell adhesion
Mog	myelin oligodendrocyte glycoprotein	39.14	cell adhesion	Slc5a11	solute carrier family 5 (sodium/glucose cotransporter), member 11	24.41	antigen processing and presentation
Mbp	myelin basic protein	21.57	myelination	Mbp	myelin basic protein	22.86	myelination
Robo3	roundabout homolog 3 (Drosophila)	17.39	neuron migration	Cyp3a9	cytochrome P450, family 3, subfamily a, polypeptide 9	19.31	sensory perception of smell
Mal	mal, T-celldifferentiation protein	16.13	intracellular protein transport	Opalin	oligodendrocytic myelin paranodal and inner loop protein	15.59	Golgi apparatus
Dnah12	dynein, axonemal, heavy polypeptide 12	12.77	microtubule-based movement	Plp1	proteolipid protein 1	12.00	glial cell differentiation
Slc5a11	solute carrier family 5 (sodium/glucose cotransporter), member 11	12.34	antigen processing and presentation	Camk2a	calcium/calmodulin- dependent protein kinase II alpha	9.59	G1/S transition of mitotic cell cycle
Hapln2	hyaluronan and proteoglycan link protein 2	9.84	cell adhesion	Lgi4	leucine-rich repeat LGI family, member 4	9.39	neuron maturation
Plp1	proteolipid protein 1	9.00	glial cell differentiation	Cpne9	copine family member IX	8.71	unknown
Ermn	ermin, ERM-like protein	8.82	morphogenesis of a branching structure	Znf76	zinc finger protein 76 (expressed in testis)	8.55	transcription
Tnnc2	troponin C type 2 (fast)	8.60	skeletal muscle contraction	Rhpn1	rhophilin, Rho GTPase binding protein 1	8.35	signal transduction
Mag	myelin-associated glycoprotein	8.12	cell adhesion	Gng13	guanine nucleotide binding protein (G protein), gamma 13	8.16	G-protein coupled receptor protein signaling pathway
Lgi4	leucine-rich repeat LGI family, member 4	8.10	neuron maturation	Mag	myelin-associated glycoprotein	7.99	cell adhesion
Extl1	exostoses (multiple)-like 1	8.09	protein binding	Herc6	hect domain and RLD 6	7.71	protein modification process
Aldh3b1	aldehyde dehydrogenase 3 family, member B1	7.81	cellular aldehyde metabolic process	Chn1	Chimerin (chimaerin) 1	6.31	signal transduction
Hhatl	hedgehog acyltransferase-like	7.79	negative regulation of N-terminal protein palmitoylation	Sept4	septin 4	5.81	cell cycle
Gng13	guanine nucleotide binding protein (G protein), gamma 13	7.44	G-protein coupled receptor protein signaling pathway	Ccdc37	coiled-coil domain containing 37	5.66	unknown
Akap8l	A kinase (PRKA) anchor protein 8-like	7.30	DNA binding	Cmtm5	CKLF-like MARVEL transmembrane domain containing 5	5.30	membrane
Cpne9	copine family member IX	7.24	Unknown	Amhr2	anti-Mullerian hormone receptor, type II	5.08	protein amino acid phosphorylation
Car12	Carbonic anyhydrase 12	7.21	one-carbon metabolic process	Sptbn4	spectrin, beta, non-erythrocytic 4	4.84	axonogenesis
Srpk3	SFRS protein kinase 3	7.11	protein amino acid phosphorylation	Gsta3	glutathione S-transferase A3	4.55	glutathione metabolic process
Casc1	cancer susceptibility candidate 1	6.90	Unknown	Grin2c	glutamate receptor, ionotropic, N-methyl D-aspartate 2 C	4.53	startle response
S1pr5	sphingosine-1-phosphate receptor 5	6.82	signal transduction	Fah	fumarylacetoacetate hydrolase	4.50	arginine catabolic process
Gpd1	glycerol-3-phosphate dehydrogenase 1 (soluble)	6.75	carbohydrate metabolic process	Ggn	gametogenetin	4.49	multicellular organismal development
Itsn2	intersectin 2	6.67	regulation of Rho protein signal transduction	Ntsr2	neurotensin receptor 2	4.13	signal transduction

Interestingly, the RMC express genes involved in myelination (such as Mbp). Mbp-like proteins, also known as Golli proteins have previously been shown to be localized in human microglia at 22 weeks postnatally [43]. MBP mRNA was found to be expressed by laser captured-RMC (Figure2D) and BV-2 microglia (Additional file 2: Figure S1). Other myelin-related genes like Plp1 [44] and Lgi4 [45] have also been found with high expression values in the RMC. Plp1 and Lgi4 were found to be expressed by non-myelinating cells such as the Bergmann glia in cerebellum of the developing mouse brain [46].

On sorting the genes based on *p* values (Additional file 3: Sheet S2), we found several genes that are specific to AMC such as, genes involved in transcriptional repression (Mbd1 which binds to methylated sites on DNA) [47,48], vesicular trafficking (Snx6, a component of the retromer complex) [49], and microtubule depolymerization (Stmn1) [50]. RMC express genes involved in immune functions such as RT1-A2, which is the MHC of rat and C1ql3, a protein of the complement system [51,52], calcium ion signaling pathway protein, Camk2 [53,54] and sodium dependent glucose transporter gene Slc5a11, known to interact with immune-related genes [55].

Functional clusters of genes specific to AMC and RMC were derived using Database for Annotation, Visualization and Integrated Discovery (DAVID) v6.7. The functional groups in AMC involved cell cycle, mRNA processing, ribosome activity, cytoskeleton, and migration (CDC42-RAC pathway) and those in RMC were cellular homeostasis, cell projection, glial cell development, axon ensheathment, and regulation of synaptic transmission and plasticity (Additional file 4: Sheet S3 & Additional file 5: Sheet S4).

**Figure 4:**
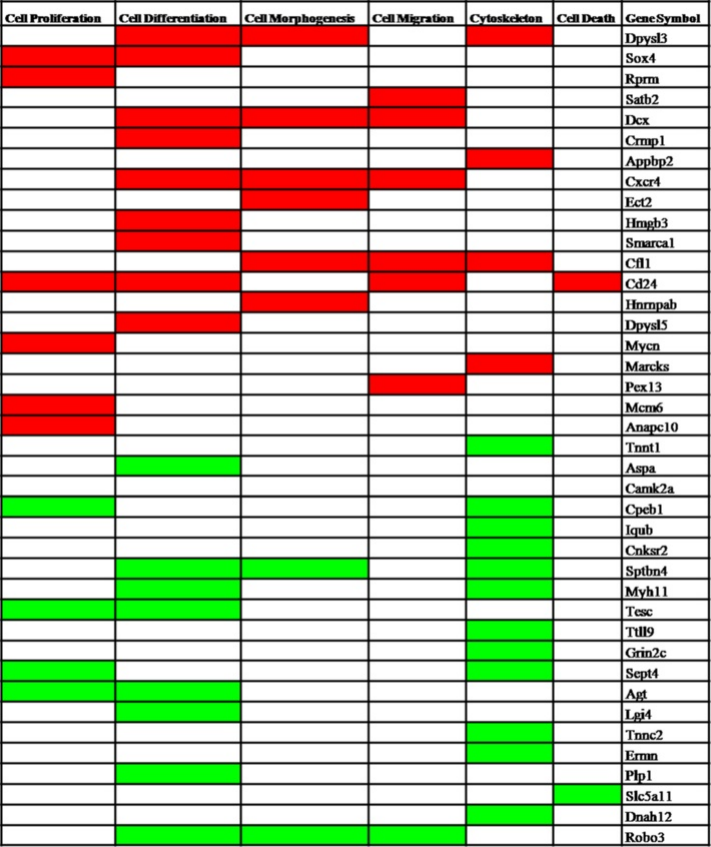
**Functional clusters to highly-expressed AMC and RMC genes.** Heat map shows the top 25 AMC and RMC (arranged according to fold change) and their involvement in major cellular functions. Red shading indicates AMC genes and green shading indicates RMC genes.

**Figure 5:**
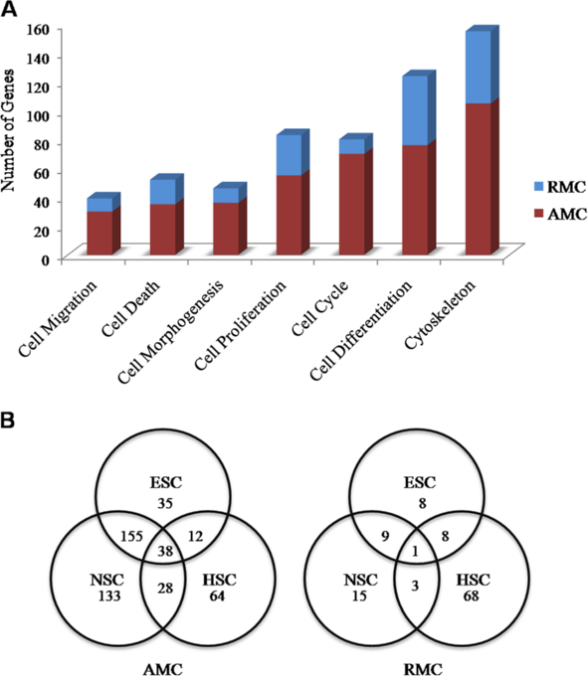
**A. Functional pathways.** Graph shows the number of AMC and RMC genes enriched in different functions. It can be noted that the AMC apart from proliferation and differentiation express a high number of genes involved in cell death whereas the RMC express a high number of cytoskeletal genes. **B. Stemness of AMC and RMC.** Venn diagrams represent the share of Embryonic (ESC), Neural (NSC) and Hematopoietic (HSC) stem cell genes in AMC and RMC. The AMC express a high number of stem cell genes compared to the RMC. The RMC express more HSC-specific genes than NSC- and ESC- specific genes.

It has been observed that in AMC, genes which are involved in cell proliferation, death, and differentiation are highly expressed, whereas in RMC, genes that are mainly involved in cytoskeletal organization and cell differentiation are highly expressed (Figure4 &5A, Refer Additional file 6: Sheet S5 for entire list of AMC and RMC genes in these functional pathways).

### Expression of monocyte-specific genes by AMC and RMC

Microglia have been proposed to originate from two distinct sources: the mesenchymal progenitor cells at the yolk sac [56] and bone-marrow derived circulating monocytes [2]. The availability of gene expression profiles for circulating monocytes prompted us to compare and study the functional similarities of AMC and RMC to that of circulating monocytes [28] (Refer Table3 &4 for pathways, Additional file 7: Sheet S6 for full gene list). Monocytic genes highly expressed by AMC (Cxcr4, Csk and Rac1) are known to be involved in several disease pathways such as Parkinson’s disease, Huntington’s disease and HIV infection, phagocytosis and chemokine signaling pathways. Further, the monocytic genes (Hla-c, Cd74, Cd302, Lsp1 and Runx3) expressed by RMC are involved in antigen presentation and lysosome-related functions. The peripheral monocytic genes expressed by the AMC and RMC may be further investigated if they reflect the microglia property in pathological brain. These genes may also provide deeper insights into the similarities between peripheral monocytes and microglia and the re-appearance of these genes during infection or neurodegeneration in the activated microglia may be critical for immune response.

**Table 3:** Pathways involving monocytic genes expressed by AMC

**Pathway**	**Nr. of Genes**	**P-Value**	**Benjamini corrected P-value**
REACT_17015:Metabolism of proteins	37	2.00E-16	1.20E-14
REACT_71:Gene Expression	42	3.30E-13	8.60E-12
REACT_1762:3′ -UTR-mediated translational regulation	24	6.20E-13	1.10E-11
Ribosome	17	1.30E-10	1.40E-08
REACT_6167:Influenza Infection	24	1.90E-09	2.50E-08
Proteasome	10	1.20E-06	6.90E-05
Pathogenic Escherichia coli infection	10	6.70E-06	2.50E-04
P00029:Huntington disease	16	7.20E-06	5.80E-04
REACT_13635:Regulation of activated PAK-2p34 by proteasome mediated degradation	10	1.50E-05	1.50E-04
P00049:Parkinson disease	12	3.40E-05	1.40E-03
REACT_9035:APC/C:Cdh1-mediated degradation of Skp2	11	3.80E-05	3.30E-04
REACT_6850:Cdc20:Phospho-APC/C mediated degradation of Cyclin A	11	7.60E-05	5.70E-04
REACT_6185:HIV Infection	18	1.70E-04	1.10E-03
REACT_11045:Signaling by Wnt	10	2.20E-04	1.30E-03
P00016:Cytoskeletal regulation by Rho GTPase	10	1.40E-03	3.70E-02
Fc gamma R-mediated phagocytosis	9	1.90E-03	5.10E-02
P00018:EGF receptor signaling pathway	11	2.40E-03	4.80E-02
P00034:Integrin signalling pathway	14	2.70E-03	4.20E-02
Neurotrophin signaling pathway	10	2.70E-03	6.00E-02
P00060:Ubiquitin proteasome pathway	8	4.30E-03	5.70E-02
REACT_578:Apoptosis	12	4.90E-03	2.50E-02
Chemokine signaling pathway	12	4.90E-03	8.80E-02
REACT_1538:Cell Cycle Checkpoints	11	5.30E-03	2.50E-02
P00021:FGF signaling pathway	10	5.80E-03	6.50E-02
REACT_383:DNA Replication	10	6.20E-03	2.70E-02

**Table 4:** Pathways involving monocytic genes expressed by RMC

**Pathway**	**Nr. of Genes**	**P-Value**	**Benjamini corrected P-value**
Lysosome	5	2.00E-03	1.00E-01
Antigen processing and presentation	4	6.60E-03	1.60E-01
70.Signal_peptides_(MHC)_class_I _molecules	2	3.30E-02	1.60E-01
Other glycan degradation	2	7.30E-02	7.40E-01
114.Genomic_reformatting_Brain _Ischemia	2	9.70E-02	2.30E-01

### Expression of neural, embryonic and hematopoietic stem cell specific genes by AMC and RMC

Both AMC and RMC express a number of stem cell specific genes [27] (Table5 &6, Additional file 8: Sheet S7). However, the RMC express a lesser number of neural stem cell (NSC) and embryonic stem cell (ESC) specific genes, compared with the AMC. In spite of this, higher percentage of hematopoietic stem cell specific genes (HSC) was found to be expressed in RMC (Figure5B). Our finding that microglia retain HSC specific properties even in the adult brain is suggestive of their hematopoietic lineage.

**Table 5:** Stem-cell genes enriched in AMC

**Gene Symbol**	**Gene Title**	**Fold Changes**	**Function**
Rprm	reprimo, TP53 dependent G2 arrest mediator candidate	19.125	cell cycle arrest
Cct8	chaperonin containing Tcp1, subunit 8 (theta)	13.525	protein folding
RGD1310352	similar to HTGN29 protein; keratinocytes associated transmembrane protein 2	13.419	Unknown
Ect2	epithelial cell transforming sequence 2 oncogene	12.498	cell morphogenesis
Cfl2	cofilin 2, muscle	12.211	protein binding
Sh3bgrl	SH3 domain binding glutamic acid-rich protein like	9.7435	Unknown
Bex4	brain expressed gene 4	9.4991	Unknown
Cfl1	cofilin 1, non-muscle	9.3235	cytokinesis
Cd24	CD24 molecule	9.2544	response to hypoxia
Ns5atp9	NS5A (hepatitis C virus) transactivated protein 9	9.1968	Unknown
Hnrnpab	heterogeneous nuclear ribonucleoprotein A/B	8.8712	epithelial to mesenchymal transition
Mycn	v-mycmyelocytomatosis viral related oncogene, neuroblastoma derived (avian)	8.6884	regulation of transcription, DNA-dependent
LOC294446	similar to Myristoylated alanine-rich C-kinase substrate (MARCKS) (ACAMP-81)	8.5949	actin binding
Pex13	peroxisomal biogenesis factor 13	8.4726	fatty acid alpha-oxidation
Maoa	monoamine oxidase A	7.8728	catecholamine metabolic process
Tmeff1	transmembrane protein with EGF-like and two follistatin-like domains 1	7.7994	multicellular organismal development
Ube2e3	ubiquitin-conjugating enzyme E2E 3, UBC4/5 homolog (yeast)	7.5905	modification-dependent protein catabolic process
Rab10	RAB10, member RAS oncogene family	7.5637	regulation of transcription, DNA-dependent
Arf4	ADP-ribosylation factor 4	7.3522	transport
Mapre1	microtubule-associated protein, RP/EB family, member 1	7.0496	cell cycle
Psme3	proteasome (prosome, macropain) activator subunit 3	7.0045	cell adhesion
Tmem43	transmembrane protein 43	6.9166	Unknown
March5	membrane-associated ring finger (C3HC4) 5	6.9148	zinc ion binding
Sqle	Squaleneepoxidase	6.7699	cellular aromatic compound metabolic process
Apaf1	apoptotic peptidase activating factor 1	6.6501	neural tube closure

**Table 6:** Stem-cell genes enriched in RMC

**Gene Symbol**	**Gene Title**	**Fold Changes**	**Function**
RGD1307882	similar to CG9346-PA	2.9758	RNA processing
Mll1	myeloid/lymphoid or mixed-lineage leukemia 1	2.9906	DNA repair
Fermt3	fermitin family homolog 3 (Drosophila)	3.0629	protein binding
Ppfibp2	PTPRF interacting protein, binding protein 2 (liprin beta 2)	3.2355	DNA integration
Mtf1	metal-regulatory transcription factor 1	3.2416	regulation of transcription, DNA-dependent
Egr1	early growth response 1	3.2473	negative regulation of transcription from RNA polymerase II promoter
Map7	microtubule-associated protein 7	3.263	cell morphogenesis
Sfrs11	splicing factor, arginine/serine-rich 11	3.3325	nuclear mRNA splicing, via spliceosome
Cd302	CD302 molecule	3.3742	binding
Ctnnal1	catenin (cadherin associated protein), alpha-like 1	3.4127	unknown
Cyp4f6	cytochrome P450 4 F6	3.4649	leukotriene metabolic process
Mlec	Malectin	3.5937	carbohydrate metabolic process
Smarca2	SWI/SNF related, matrix associated, actin dependent regulator of chromatin, subfamily a, member 2	3.663	negative regulation of cell proliferation
Scn1b	sodium channel, voltage-gated, type I, beta	3.6699	transport
Tmbim1	transmembrane BAX inhibitor motif containing 1	3.7938	unknown
Pck2	phosphoenolpyruvatecarboxykinase 2 (mitochondrial)	3.8945	gluconeogenesis
Tnni3	troponin I type 3 (cardiac)	4.0048	vasculogenesis
Nefh	neurofilament, heavy polypeptide	4.2066	microtubule cytoskeleton organization
Prpf38b	PRP38 pre-mRNA processing factor 38 (yeast) domain containing B	4.3721	mRNA processing
Tnnt1	troponin T type 1 (skeletal, slow)	4.4062	skeletal muscle contraction
Zranb2	zinc finger, RAN-binding domain containing 2	4.598	mRNA processing
Zrsr1	zinc finger (CCCH type), RNA binding motif and serine/arginine rich 1	5.0348	nucleotide binding
Lrrc23	leucine rich repeat containing 23	5.1395	protein binding
Akap8l	A kinase (PRKA) anchor protein 8-like	5.6932	DNA binding
Tnnc2	troponin C type 2 (fast)	8.6024	skeletal muscle contraction

### Pathway analysis and validation of differentially expressed genes in AMC and RMC

The pathway analysis (Additional file 9: Figure S2) revealed novel molecular networks involving several signaling molecules and pathways within microglia. In order to validate the results obtained from pathway analysis, we randomly selected three transcription factors which are highly expressed in the AMC. They are: Sox4 and Sox11 which are SRY-related HMG-box family of transcription factors [57] and Runt-related transcription factor 1; translocated to, 1 (cyclinD-related) (Runx1t1), a member of the ETO gene family of transcriptional co-repressors [58].

**Figure 6:**
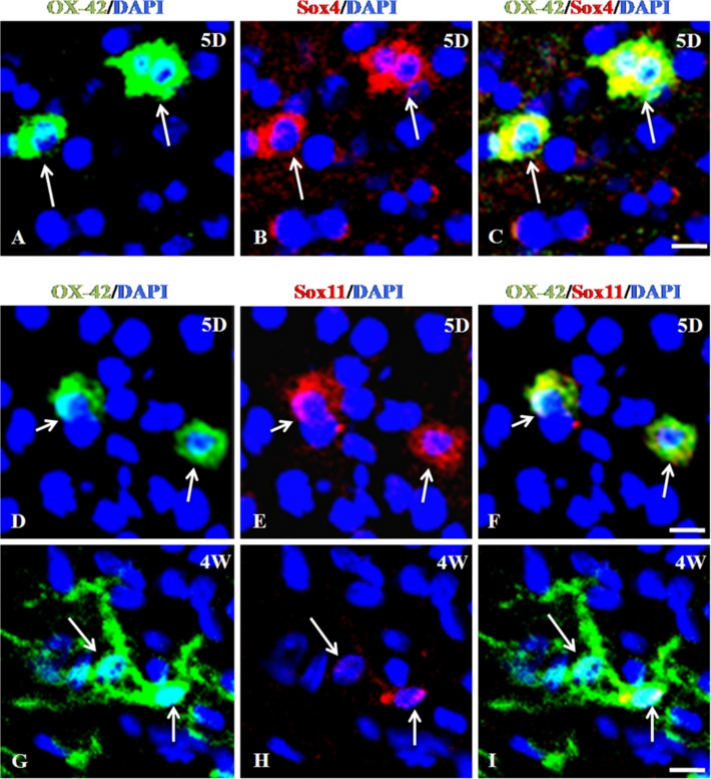
**A-I. Differential immunoexpression of Sox4 and Sox11 in AMC and RMC.** Confocal images showing a high immunoexpression of Sox4 (B; red) and its co-localization (C) in OX42 (A; green) labeled AMC. Immunoexpression of Sox11 (E, H; red) and its co-localization (F, I) in OX42 (D, G; green) labeled AMC and RMC in the CC from 5-day (5D) and 4-week (4 W) old rat brain was also observed. Sox11immuno expression is undetectable in RMC (I) compared to that in the AMC (F). (DAPI – blue).Scale bars: A-I 10 μm.

Runx1t1, by forming a fusion protein with Runx1, another member of RUNX family, leads to self-renewal of human monocytic cells thereby impairing differentiation of these cells [59]. Certain genes known to be downregulated by the Runx1-Runx1t1 transcription factor complex such as Socs1, Csf1, and Runx3 are highly expressed by RMC [60] (Additional file 5: Sheet S4). Further, a transcriptional dysregulation caused by this fusion protein was found to cause the over-expression of Sox4 in human progenitor cells [60]. In an earlier study, Sox4 deficient mice exhibited proliferation-defective pro-B cells [61]. Similar networks might function in the AMC and therefore warrants further investigation. Our immunohistochemical analysis revealed that Sox4 (Figure6A-C) is highly expressed in the nucleus and cytoplasm of AMC. Similarly, Sox11 (Figure6D-I) and Runx1t1 (Figure3D-I) are expressed in the AMC, but hardly detectable in the RMC thus validating our microarray results. Further, quantitative real time RTPCR for Runx1t1 using RNA extracted from LCM-captured AMC and RMC showed a very high expression of Runx1t1 in AMC when compared to RMC (Figure2D).

### Differential expression of septin genes in AMC and RMC

**Figure 7:**
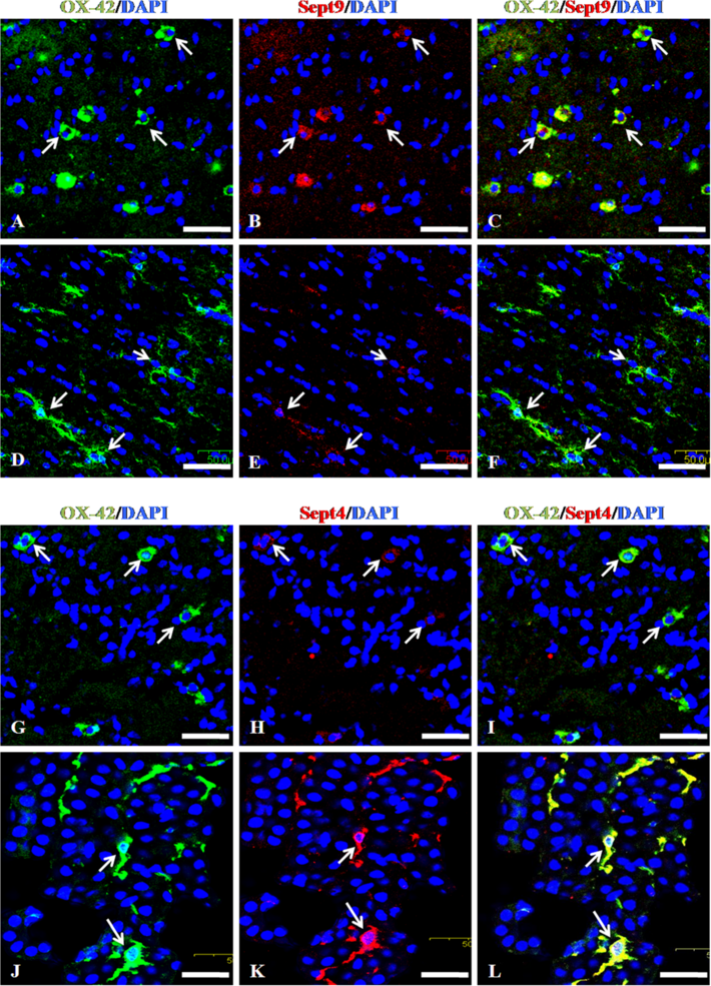
**A-L. Immunoexpression of Sept9 and Sept4 in RMC.** Confocal images showing the immunoexpression of Sept9 and Sept4 (B, E, H, K; red) and their co-localization (C, F, I, L) in OX42 (A, D, G, J; green) labeled AMC and RMC in the CC from 5-day (5D) and 4-week (4 W) old rat brain.Sept9 immunoexpression is undetectable in RMC (F) compared to that in the AMC (C) whereas Sept4 is undetectable in AMC (I) compared to that in the RMC (L). (DAPI – blue).Scale bars: A-L 50 μm.

Septins are a family of multifunctional proteins involved in cytoskeletal organisation and cell division [62-64]. They have also been implicated in tumorigenesis and neurodegeneration [65]. In the present study, Septin genes were found to be differentially expressed in AMC and RMC. Sept3, 6, 9 and 11 were expressed in AMC whereas Sept4 and 8 expressed in RMC. The expression of some of these genes was further confirmed by immunohistochemical and quantitative real time RTPCR analysis which showed Sept9 immunoexpression and mRNA expression in the AMC (Figure7A-F and Figure2D) and Sept4 expression in the RMC (Figure7G-L and Figure2D). Since the role of Septins in microglial functioning has not yet been investigated, studies on the Septin family may further our knowledge on the cytoskeletal dynamics involved in proliferation, migration and activation of AMC and RMC.

Microglia in response to trauma or neurodegenerative stimuli exhibit upregulation of proinflammatory cytokines and chemokines [66]. In the present study, both AMC and RMC exhibited relatively low expression intensities for most of the cytokines such as TNF-α and interleukins and chemokines such as Cxcl3, Cxcl12 and Ccl2 (Additional file 10: Sheet S8).

## Discussion

### Microglia in the healthy brain express low levels of cytokines and chemokines

This study is a novel attempt to examine the global gene expression profile of microglia *in situ* and to functionally distinguish the two distinct microglial phenotypes, namely, AMC and RMC. A noteworthy feature of this transcriptome profile was that the expression of cytokines and chemokines in both AMC and RMC was hardly detectable which is in agreement with previous studies [24]. It has been widely shown that the untreated microglia in culture produce some amount of proinflammatory cytokines and chemokines, indicating that culture media stimulate the microglial cells. Significantly, the low expression of cytokines and chemokines in both types of microglia in the present study appears to mimic the transcriptome status of normal microglia in healthy brain *in vivo.*

### AMC express genes involved in cell cycle process and migration whereas RMC express genes involved in synaptic integrity and neuronal maturation

AMC from the first week of postnatal rat brains have a high proliferative capacity [2]. During development, about two-thirds of AMC undergo apoptosis and the rest transform into RMC [67,68]. In accordance with this, microarray analysis in the present study revealed a high expression of cell proliferation/cell cycle-related genes such as Myc and CyclinA2, CyclinB2 and CyclinD1 (Additional file 6: Sheet S5) [69,70] and genes involved in cell death (Figure5A & Additional file 6: Sheet S5) namely, Casp2,Casp3 [71,72] and Apaf1(Glial apoptosis related gene) in the AMC [73]. It is striking that the AMC express Dcx, a protein known to be a marker for migrating neurons [74]. It may be worth investigating the role of Dcx in migration of AMC in the early postnatal brain.

On the other hand, the RMC, apart from cell homeostasis and glial development, appear to contribute to synaptic transmission as they express genes such as Grin2c, S100b and Camk2a (Additional file 5: Sheet S4) [75-77]. This is interesting and supports the recent experimental studies showing the role of microglia in the maintenance and modifications of synaptic integrity in the healthy brain [78,79]. Further, Grin2c, a subunit of NMDA receptor complex is expressed by the microglial cells in the CC and has been shown to be functionally important in microglia-mediated neuroinflammation [80,81]. S100b, a calcium ion binding protein, is also expressed by microglia and relocates around phagosomes during microglial activation and phagocytosis [82,83].

RMC express myelin basic protein (Mbp) which encodes two families of proteins i.e., classic Mbps and Golli-Mbps. Classic Mbps serve as the major protein constituent of myelin in the central and peripheral nervous system whereas, the Golli proteins are known to be broadly distributed in the brain, particularly in the microglia during normal development and inflammation [84] and involved in the interaction between microglia and oligodendrocyte precursor cells during multiple sclerosis [85]. Taken together, these results reveal that microglia are not only involved in immune response and phagocytosis but also play diverse roles in healthy brain.

### Both AMC and RMC express cytoskeleton-related genes

Regulation of cytoskeletal dynamics is important to both microglial migration and ramification [86-88]. Apart from cytoskeletal structural proteins such as tubulins and actin, we found that the AMC express cytoskeleton-associated Crmp family proteins [89] such as Crmp-1, Dpysl3 and Dpysl5 and Septin family proteins such as Sept9 and 11 [90,91]. Septins are implicated in cytoskeletal processes such as vesicular trafficking [92]. These cytoskeleton-associated proteins may therefore explain the migration and phagocytosis of AMC during normal development and pathology. In the present study, AMC express Sept9 but not Sept4 whereas, RMC express Sept4, but not Sept9, indicating differential roles of Septin family genes in AMC and RMC. Sept4 has been recently shown to be involved in cortical neuron migration [93]. Absence of Sept4 immunoexpression in the AMC and its high expression in the RMC is suggestive of an important role for this protein in microglial transformation during development.

### Expression of monocyte- and stem cell-specific genes by AMC and RMC indicates their stemness and origin

Recent studies have proven that microglia originate from the mesenchymal progenitor cells at the yolk sac [56]. However, microarray studies of various hematopoetic and non-hematopoetic cell types revealed a close relationship between the gene expression profiles of microglia and bone-marrow derived macrophages [94] which are known to differentiate from circulating monocytes. Therefore, we sought to identify the monocyte-specific genes expressed by AMC and RMC. AMC express several monocyte-specific genes including Mcl1 and Id2. Mcl1 is associated with cell viability and differentiation of myeloid cells which include monocytes and macrophages [95,96] and Id2, a negative regulator of basic helix loop helix transcription factors, is involved in the differentiation of myeloid cells [97]. A recent study demonstrated that Id2 is required for bone morphogenic protein (Bmp)-mediated differentiation of microglia into Map2^+^ neurons and Gfap^+^ astrocytes [98] suggesting that this gene may promote microglial trans-differentiation. Both Mcl1 and Id2 have been shown to be involved in cell differentiation and their high expression in AMC explains the role of these genes in promoting the maturation of AMC and its transformation into RMC

On the other hand, RMC exhibited increased expression of Lsp1, which binds to the cytoskeleton and is known to be a marker for leucocytes [99]. Overexpression of Lsp1 in neutrophils was associated to defective actin polymerization which render these cells immotile [100]. Further, overexpression of Lsp1 in a highly motile melanoma cell line led to formation of hair-like projections. Thus, upregulation of Lsp1 in the RMC, compared to the AMC may explain the role of this gene in motility and ramification of RMC [101] which are the resident population in the adult brain parenchyma.

Both AMC and RMC express stem cell-specific (ESC, NSC and HSC) genes, indicating their stemness and suggesting that microglia may undergo trans-differentiation. The RMC expressed a high percentage of HSC specific genes in comparison to ESC and NSC specific genes, and this, reinforces the monocytic nature of microglia. For example Mll1, a highly expressed HSC specific gene in the RMC, is a histone methyl transferase whose functional disruption is implicated in human leukemia [102]. Understanding the functions of these HSC specific genes may be important in comprehending the immune system related-roles of AMC and RMC.

### AMC express proliferation- and differentiation-related genes, Sox4, Sox11 and Runx1t1

In order to validate the microarray data, we have analyzed the expression patterns of SOX genes (Sox4 and Sox11) which are known to be involved in differentiation and Runx1t1, which is involved in the proliferation of hematopoietic lineage cells. These genes were highly expressed by the AMC and their expression and role have not been studied in microglia, so far. Initially, nuclear expression of the transcription factor Sox11 was shown to be associated with embryonic neurogenesis and lymphopoiesis [103,104]. However, there are no data on the role of SOX genes in microglia in which Sox11 is expressed in the cytoplasm as reported in plasma myeloma cells and other B-cell lymphomas [104]. According to previous studies, the overexpression of the fusion protein, Runx1-Runx1t1 causes the downregulation of Csf-1 (a hematopoietic cytokine known to cause activation of microglia) [105] and Runx3 (a tumour suppressor) [106]. Our expression profile showed the increased expression of Runx1t1 in the AMC and downregulation of Csf-1 and Runx3 in AMC compared to RMC. Functional analysis of these transcription factors may help in understanding microglial proliferation and differentiation.

## Conclusions

Overall, the transcriptome profiling has identified several genes, which help in elucidating morphological transformation and functions of AMC and RMC. These genes not only represent the physiological role of microglia in the developing brain but may also be useful therapeutic targets in neuropathologies in which microglia are implicated.

## Competing interests

The authors declare that they have no competing interests.

## Authors’ contributions

RP performed majority of the experiments and wrote the manuscript. BJ, BN, MJ1 and MJ2 performed some experiments and participated in discussion. JL, SST and EAL participated actively in discussion of the project and editorial work of the manuscript. STD is the Principal Investigator and was instrumental to the execution of the entire project. All of the authors have read and approved the final version of the manuscript.

## Supplementary Material

Additional file 1 **Sheet S1.** Primer sequences . Click here for file

Additional file 2 **Figure S1. Expression of Dcx and Mbp in BV-2 microglia.** PCR was done using cDNA obtained from BV-2 microglia for Dcx and Mbp. Both Dcx and Mbp were found to be expressed by BV-2 microglia as identified by the PCR products in agarose gel electrophoresis. Click here for file

Additional file 3 **Sheet S2.** AMC and RMC genes with P-value. Click here for file

Additional file 4 **Sheet S3.** AMC and RMC gene clusters. Click here for file

Additional file 5 **Sheet S4.** Genes involved in CDC42-RAC pathway for migration, Synaptic transmission and genes downregulated by the Runx1-Runx1t1 complex. Click here for file

Additional file 6 **Sheet S5.** Functions of AMC and RMC. Click here for file

Additional file 7 **Sheet S6.** Monocytic genes enriched in AMC and RMC. Click here for file

Additional file 8 **Sheet S7.** ESC, NSC and HSC genes enriched in AMC and RMC. Click here for file

Additional file 9 **Figure S2. Pathway analysis.** Novel molecular networks identified by inputting AMC and RMC expression data into Adriane Pathway Studio. Red colored shapes specify AMC genes and violet colored shapes specify RMC genes. Some AMC genes - Sox4, Sox11, Runx1t1 are highlighted in the figure. Click here for file

Additional file 10 **Sheet S8.** Expression of cytokines in AMC and RMC. Click here for file
