# Supplementary material for: Transcriptome analysis of amoeboid and ramified microglia isolated from the corpus callosum of rat brain
Source: BMC Neurosci. 2012 Jun 14;13:64. doi: 10.1186/1471-2202-13-64 (PMC3441342; doi:10.1186/1471-2202-13-64)
Supplement: Additional file 1 — Sheet S1. Primer sequences . [file 1471-2202-13-64-S1.docx]

| **Marker** | **Cell specificity** | **Forward primer** | **Reverse primer** | **Product size (bp)** |
| --- | --- | --- | --- | --- |
| Cd11b (OX42) | Microglia | AACACCAAGGACAGGCTGCG | TGCGCCTGAGTATGCCGTTC | 127 |
| Gfap | Astrocytes | GGTGTGGAGTGCCTTCGTAT | TACGATGTCCTGGGAAAAGG | 139 |
| Vim | Endothelial cells | AATGCTTCTCTGGCACGTCT | GCTCCTGGATCTCTTCATCG | 100 |
| CNPase | Oligodendrocytes | ACGGCGTGGCGACTAGACTGT | CCGGGCTTCAGCTTCTTCAGG | 86 |
| Gapdh | Positive control | TCAAGAAGGTGGTGAAGCAG | AGGTGGAAGAATGGGAGTTG | 111 |
| Mbp | NA | CAGGGAGCCATAATGGGTAG | GCTTCTTTAGCGGTGACAGG | 163 |
| Dcx | NA | CCAAGAGAGAACAGCAAACCA | TCCAGTCAGCAAAGGTAAGGA | 145 |
| B actin | Positive control | GGATTCCATACCCAAGAAGGA | GAAGAGCTATGAGCTGCCTGA | 103 |
