# Supplementary figures and images for: Transcriptome analysis of amoeboid and ramified microglia isolated from the corpus callosum of rat brain
Source: BMC Neurosci. 2012 Jun 14;13:64. doi: 10.1186/1471-2202-13-64 (PMC3441342; doi:10.1186/1471-2202-13-64)

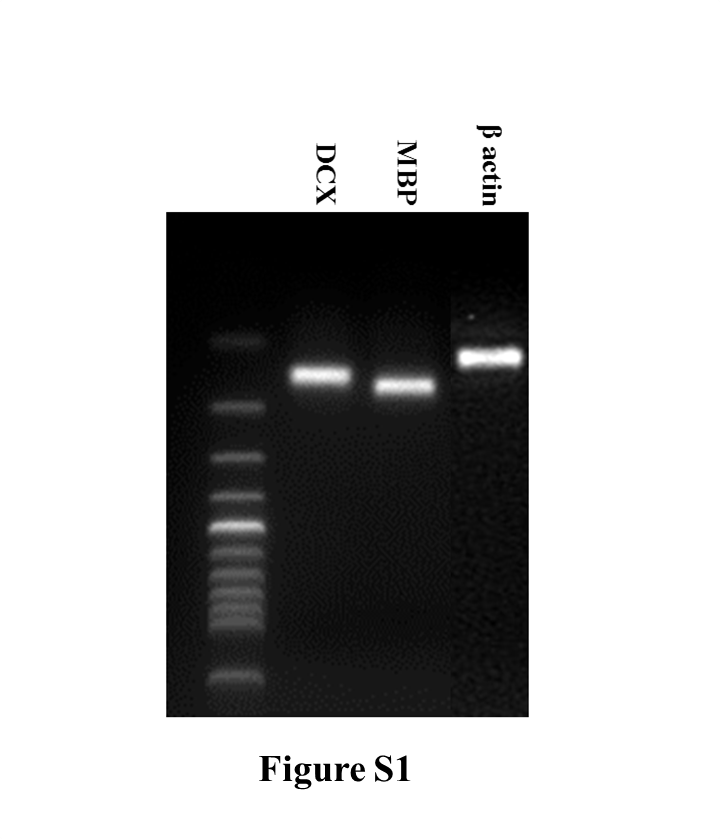

Supplement: Additional file 2 — Figure S1. Expression of Dcx and Mbp in BV-2 microglia. PCR was done using cDNA obtained from BV-2 microglia for Dcx and Mbp. Both Dcx and Mbp were found to be expressed by BV-2 microglia as identified by the PCR products in agarose gel electrophoresis. [file 1471-2202-13-64-S2.tiff]

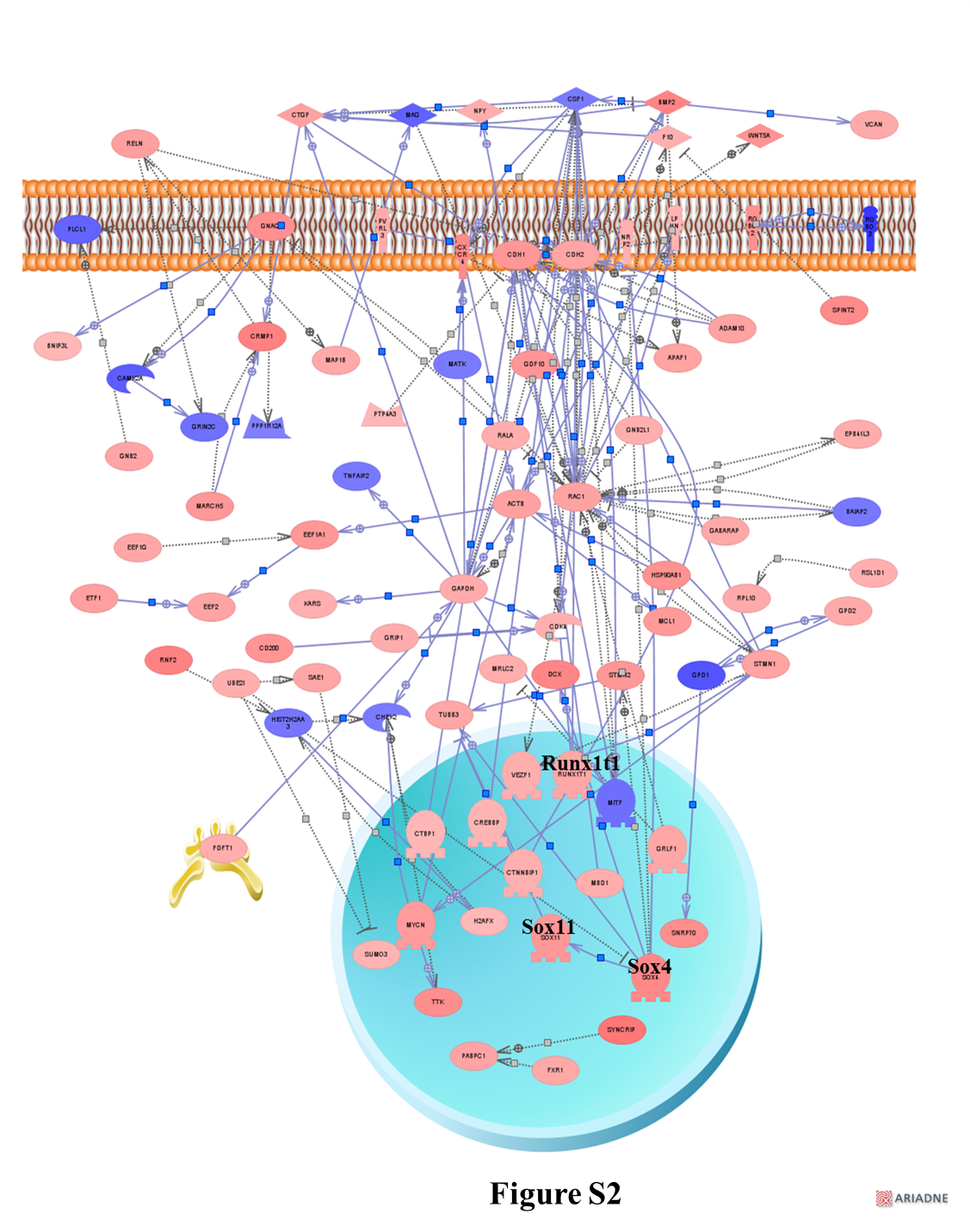

Supplement: Additional file 9 — Figure S2. Pathway analysis. Novel molecular networks identified by inputting AMC and RMC expression data into Adriane Pathway Studio. Red colored shapes specify AMC genes and violet colored shapes specify RMC genes. Some AMC genes - Sox4, Sox11, Runx1t1 are highlighted in the figure. [file 1471-2202-13-64-S9.tiff]
