# Supplementary material for: Transcriptome analysis of amoeboid and ramified microglia isolated from the corpus callosum of rat brain
Source: BMC Neurosci. 2012 Jun 14;13:64. doi: 10.1186/1471-2202-13-64 (PMC3441342; doi:10.1186/1471-2202-13-64)
Supplement: Additional file 3 — Sheet S2. AMC and RMC genes with P-value. [file 1471-2202-13-64-S3.docx]

**Top 25 AMC specific genes sorted using *p values***

| **Probe ID** | **Gene Symbol** | **Gene Title** | **Fold Change** | ***p value*** | **Function** |
| --- | --- | --- | --- | --- | --- |
| 1371533_at | Dctn6 | dynactin 6 | 2.3803 | 9.03E-06 | transferase activity |
| 1380407_at | RGD1310352 | similar to HTGN29 protein; keratinocytes associated transmembrane protein 2 | 13.419 | 3.70E-05 | --- |
| 1393082_at | Ppp1r14c | protein phosphatase 1, regulatory (inhibitor) subunit 14c | 3.7916 | 4.32E-05 | regulation of phosphorylation |
| 1371782_at | Nipsnap3a | nipsnap homolog 3A (C. elegans) | 9.0179 | 6.17E-05 | --- |
| 1392885_at | Mbd1 | methyl-CpG binding domain protein 1 | 2.1202 | 6.78E-05 |  |
| 1398803_at | Dync1h1 | dynein cytoplasmic 1 heavy chain 1 | 2.6624 | 7.74E-05 | microtubule-based movement |
| 1371779_at | Snx6 | sorting nexin 6 | 6.3942 | 9.84E-05 | cell communication |
| 1371133_a_at | Prkar2b | protein kinase, cAMP dependent regulatory, type II beta | 2.7781 | 0.000135 | regulation of protein amino acid phosphorylation |
| 1370213_at | Ybx1 | Y box binding protein 1 | 3.7191 | 0.00016 | negative regulation of transcription from RNA polymerase II promoter |
| 1388588_at | Mtvr2 | mammary tumor virus receptor 2 | 5.7502 | 0.000182 | receptor activity |
| 1387899_at | Crmp1 | collapsin response mediator protein 1 | 15.498 | 0.000184 | neuron development |
| 1384000_at | Sox4 | SRY (sex determining region Y)-box 4 | 28.328 | 0.000221 | response to hypoxia |
| 1372180_at | Sdc3 | syndecan 3 | 2.4525 | 0.000228 | cell adhesion |
| 1376658_at | Raph1 | Ras association (RalGDS/AF-6) and pleckstrin homology domains 1 | 6.045 | 0.000245 | signal transduction |
| 1371949_at | Bzw1 | basic leucine zipper and W2 domains 1 | 6.3039 | 0.000245 | transcription |
| 1380619_at | RGD1305537 | similar to RIKEN cDNA 3110001I22 | 6.8767 | 0.000258 | --- |
| 1390259_at | Ube2d1 | ubiquitin-conjugating enzyme E2D 1, UBC4/5 homolog (yeast) | 5.0533 | 0.000265 | protein polyubiquitination |
| 1377935_at | Cldnd1 | Claudin domain containing 1 | 2.2565 | 0.000271 | --- |
| 1386857_at | Stmn1 | stathmin 1 | 4.7384 | 0.000286 | microtubule depolymerization |
| 1389788_at | Morn4 | MORN repeat containing 4 | 2.3078 | 0.000298 | --- |

**Top 25 RMC specific genes sorted using *p values***

| **Probe ID** | **Gene Symbol** | **Gene Title** | **Fold Changes** | ***p value*** | **Function** |
| --- | --- | --- | --- | --- | --- |
| 1370434_a_at | Mobp | myelin-associated oligodendrocyte basic protein | 623.18 | 1.23E-05 | nervous system development |
| 1380436_at | RT1-A2 | RT1 class Ia, locus A2 | 2.1753 | 3.09E-05 | antigen processing and presentation of peptide antigen via MHC class I |
| 1377803_at | Ccdc153 | coiled-coil domain containing 153 | 4.1465 | 8.61E-05 | --- |
| 1374374_x_at | Shroom1 | shroom family member 1 | 2.2842 | 0.00011 | cell morphogenesis |
| 1378088_at | Zdhhc1 | zinc finger, DHHC-type containing 1 | 2.2872 | 0.000132 | protein palmitoylation |
| 1369607_at | Fgf6 | fibroblast growth factor 6 | 2.1594 | 0.000147 | cartilage condensation |
| 1374621_at | Taf1c | TATA box binding protein (Tbp)-associated factor, RNA polymerase I, C | 2.3723 | 0.0002 | transcription |
| 1395399_at | Zcchc17 | zinc finger, CCHC domain containing 17 | 2.0382 | 0.000203 | RNA binding |
| 1372140_at | Ccdc28a | coiled-coil domain containing 28A | 3.8307 | 0.000361 | --- |
| 1371997_at | Akr1cl2 | aldo-keto reductase family 1, member C-like 2 | 2.4959 | 0.000385 | oxidation reduction |
| 1387341_a_at | Mbp | myelin basic protein | 4.7084 | 0.000396 | myelination |
| 1370849_at | Hapln2 | hyaluronan and proteoglycan link protein 2 | 9.8354 | 0.000399 | cell adhesion |
| 1382188_at | Gpatch4 | G patch domain containing 4 | 3.2975 | 0.000466 | nucleic acid binding |
| 1390158_at | LOC304903 | similar to Pappalysin-2 precursor (Pregnancy-associated plasma protein-A2) (PAPP-A2) | 2.1139 | 0.000478 | proteolysis |
| 1391386_at | Chn1 | Chimerin (chimaerin) 1 | 5.3575 | 0.00057 | signal transduction |
| 1391884_at | F12 | coagulation factor XII (Hageman factor) | 3.1552 | 0.000593 | Factor XII activation |
| 1374349_at | Ctdspl | CTD (carboxy-terminal domain, RNA polymerase II, polypeptide A) small phosphatase-like | 2.206 | 0.000618 | phosphatase activity |
| 1372240_at | Sgca | sarcoglycan, alpha (dystrophin-associated glycoprotein) | 3.5115 | 0.000646 | calcium ion binding |
| 1388187_at | Camk2a | calcium/calmodulin-dependent protein kinase II alpha | 4.6656 | 0.000665 | calcium ion transport |
| 1384092_at | C1ql3 | Complement component 1, q subcomponent-like 3 | 2.1413 | 0.00073469 | protein binding |
