# Supplementary material for: Transcriptome analysis of amoeboid and ramified microglia isolated from the corpus callosum of rat brain
Source: BMC Neurosci. 2012 Jun 14;13:64. doi: 10.1186/1471-2202-13-64 (PMC3441342; doi:10.1186/1471-2202-13-64)
Supplement: Additional file 4 — Sheet S3. AMC and RMC gene clusters. [file 1471-2202-13-64-S4.docx]

**Functional clusters specific to AMC**

| **Annotation Cluster 1** | **Enrichment Score: 10.24** | **Function** | **Number of Genes** | ***p value*** | **Benjamini** |
| --- | --- | --- | --- | --- | --- |
|  | GOTERM_CC_FAT | non-membrane-bounded organelle | 196 | 7.10E-15 | 1.80E-12 |
|  | GOTERM_CC_FAT | intracellular non-membrane-bounded organelle | 196 | 7.10E-15 | 1.80E-12 |
|  | GOTERM_CC_FAT | microtubule cytoskeleton | 57 | 1.30E-09 | 1.70E-07 |
|  | GOTERM_CC_FAT | cytoskeleton | 105 | 1.00E-08 | 1.00E-06 |
|  | GOTERM_CC_FAT | cytoskeletal part | 78 | 9.80E-07 | 4.60E-05 |
| **Annotation Cluster 2** | **Enrichment Score: 6.87** | **Function** | **Number of Genes** | ***p value*** | **Benjamini** |
|  | GOTERM_CC_FAT | nuclear lumen | 111 | 5.30E-11 | 9.10E-09 |
|  | GOTERM_CC_FAT | intracellular organelle lumen | 124 | 1.20E-08 | 1.00E-06 |
|  | GOTERM_CC_FAT | membrane-enclosed lumen | 128 | 4.40E-08 | 3.20E-06 |
|  | GOTERM_CC_FAT | organelle lumen | 124 | 9.70E-08 | 6.20E-06 |
|  | GOTERM_CC_FAT | nucleoplasm | 78 | 9.80E-07 | 4.60E-05 |
|  | GOTERM_CC_FAT | nucleoplasm part | 44 | 2.20E-03 | 3.40E-02 |
| **Annotation Cluster 3** | **Enrichment Score: 5.73** | **Function** | **Number of Genes** | ***p value*** | **Benjamini** |
|  | GOTERM_CC_FAT | microtubule cytoskeleton | 57 | 1.30E-09 | 1.70E-07 |
|  | GOTERM_CC_FAT | microtubule | 31 | 1.30E-07 | 7.70E-06 |
|  | GOTERM_CC_FAT | cytoskeletal part | 78 | 9.80E-07 | 4.60E-05 |
|  | SP_PIR_KEYWORDS | microtubule | 23 | 2.60E-04 | 4.80E-03 |
|  | GOTERM_BP_FAT | microtubule-based process | 27 | 4.90E-04 | 1.90E-02 |
| **Annotation Cluster 4** | **Enrichment Score: 5.57** | **Function** | **Number of Genes** | ***p value*** | **Benjamini** |
|  | GOTERM_MF_FAT | cytoskeletal protein binding | 57 | 2.50E-08 | 1.20E-05 |
|  | GOTERM_MF_FAT | actin binding | 35 | 9.50E-06 | 1.10E-03 |
|  | SP_PIR_KEYWORDS | actin-binding | 24 | 8.20E-05 | 2.00E-03 |
| **Annotation Cluster 5** | **Enrichment Score: 5.56** | **Function** | **Number of Genes** | ***p value*** | **Benjamini** |
|  | SP_PIR_KEYWORDS | protein biosynthesis | 42 | 2.10E-13 | 2.10E-11 |
|  | GOTERM_BP_FAT | translational elongation | 28 | 4.90E-11 | 1.50E-07 |
|  | SP_PIR_KEYWORDS | ribonucleoprotein | 38 | 5.90E-10 | 4.80E-08 |
|  | KEGG_PATHWAY | Ribosome | 25 | 8.30E-10 | 1.40E-07 |
|  | SP_PIR_KEYWORDS | ribosome | 23 | 5.30E-08 | 3.60E-06 |
|  | GOTERM_CC_FAT | cytosolic ribosome | 15 | 2.30E-06 | 9.90E-05 |
|  | GOTERM_BP_FAT | translation | 55 | 3.20E-06 | 2.80E-04 |
|  | SP_PIR_KEYWORDS | ribosomal protein | 27 | 8.40E-06 | 3.10E-04 |
|  | GOTERM_CC_FAT | cytosolic part | 21 | 1.60E-05 | 6.20E-04 |
|  | GOTERM_CC_FAT | ribonucleoprotein complex | 56 | 7.10E-05 | 2.30E-03 |
|  | GOTERM_CC_FAT | cytosolic small ribosomal subunit | 11 | 7.60E-05 | 2.30E-03 |
|  | GOTERM_CC_FAT | small ribosomal subunit | 12 | 1.70E-04 | 4.00E-03 |
|  | GOTERM_CC_FAT | ribosomal subunit | 16 | 2.10E-04 | 4.50E-03 |
|  | GOTERM_MF_FAT | structural molecule activity | 50 | 4.00E-02 | 5.10E-01 |
|  | GOTERM_CC_FAT | ribosome | 30 | 5.20E-02 | 3.40E-01 |
|  | GOTERM_MF_FAT | structural constituent of ribosome | 27 | 8.60E-02 | 7.10E-01 |
| **Annotation Cluster 6** | **Enrichment Score: 5.46** | **Function** | **Number of Genes** | ***p value*** | **Benjamini** |
|  | GOTERM_BP_FAT | cell cycle | 70 | 8.90E-11 | 1.30E-07 |
|  | GOTERM_BP_FAT | proteasomal protein catabolic process | 24 | 1.80E-09 | 1.80E-06 |
|  | GOTERM_BP_FAT | proteasomal ubiquitin-dependent protein catabolic process | 24 | 1.80E-09 | 1.80E-06 |
|  | GOTERM_BP_FAT | mitotic cell cycle | 42 | 2.40E-09 | 1.80E-06 |
|  | GOTERM_BP_FAT | modification-dependent protein catabolic process | 47 | 1.30E-08 | 6.30E-06 |
|  | GOTERM_BP_FAT | modification-dependent macromolecule catabolic process | 47 | 1.30E-08 | 6.30E-06 |
|  | GOTERM_BP_FAT | protein catabolic process | 52 | 1.40E-08 | 6.00E-06 |
|  | GOTERM_BP_FAT | anaphase-promoting complex-dependent proteasomal ubiquitin-dependent protein catabolic process | 18 | 3.50E-08 | 1.30E-05 |
|  | GOTERM_BP_FAT | cellular protein catabolic process | 49 | 3.60E-08 | 1.20E-05 |
|  | GOTERM_BP_FAT | proteolysis involved in cellular protein catabolic process | 48 | 7.10E-08 | 2.20E-05 |
|  | GOTERM_BP_FAT | ubiquitin-dependent protein catabolic process | 32 | 1.20E-07 | 3.30E-05 |
|  | GOTERM_BP_FAT | regulation of ubiquitin-protein ligase activity during mitotic cell cycle | 18 | 1.50E-07 | 3.80E-05 |
|  | GOTERM_BP_FAT | regulation of ligase activity | 19 | 2.10E-07 | 4.60E-05 |
|  | GOTERM_BP_FAT | negative regulation of ubiquitin-protein ligase activity during mitotic cell cycle | 17 | 2.30E-07 | 4.30E-05 |
|  | GOTERM_BP_FAT | positive regulation of ligase activity | 18 | 2.60E-07 | 4.60E-05 |
|  | GOTERM_BP_FAT | negative regulation of ligase activity | 17 | 3.10E-07 | 4.80E-05 |
|  | GOTERM_BP_FAT | negative regulation of ubiquitin-protein ligase activity | 17 | 3.10E-07 | 4.80E-05 |
|  | GOTERM_BP_FAT | positive regulation of ubiquitin-protein ligase activity during mitotic cell cycle | 17 | 5.20E-07 | 7.50E-05 |
|  | GOTERM_BP_FAT | regulation of ubiquitin-protein ligase activity | 18 | 7.00E-07 | 9.20E-05 |
|  | GOTERM_BP_FAT | positive regulation of ubiquitin-protein ligase activity | 17 | 8.80E-07 | 1.10E-04 |
|  | GOTERM_BP_FAT | cellular macromolecule catabolic process | 54 | 9.70E-07 | 1.10E-04 |
|  | GOTERM_BP_FAT | macromolecule catabolic process | 59 | 1.40E-06 | 1.60E-04 |
|  | GOTERM_BP_FAT | cell cycle process | 49 | 1.80E-06 | 1.90E-04 |
|  | GOTERM_BP_FAT | negative regulation of protein ubiquitination | 17 | 1.80E-06 | 1.90E-04 |
|  | GOTERM_BP_FAT | positive regulation of protein ubiquitination | 18 | 2.20E-06 | 2.10E-04 |
|  | GOTERM_BP_FAT | regulation of protein ubiquitination | 19 | 1.50E-05 | 1.10E-03 |
|  | GOTERM_BP_FAT | negative regulation of protein modification process | 21 | 1.80E-05 | 1.20E-03 |
|  | GOTERM_CC_FAT | proteasome complex | 14 | 2.50E-05 | 8.60E-04 |
|  | SP_PIR_KEYWORDS | proteasome | 13 | 6.00E-05 | 1.80E-03 |
|  | GOTERM_BP_FAT | positive regulation of protein metabolic process | 33 | 6.60E-05 | 3.70E-03 |
|  | KEGG_PATHWAY | Proteasome | 13 | 1.20E-04 | 3.20E-03 |
|  | GOTERM_BP_FAT | positive regulation of cellular protein metabolic process | 31 | 1.50E-04 | 7.40E-03 |
|  | GOTERM_BP_FAT | positive regulation of catalytic activity | 51 | 1.50E-04 | 7.40E-03 |
|  | GOTERM_BP_FAT | positive regulation of molecular function | 56 | 1.90E-04 | 8.70E-03 |
|  | GOTERM_BP_FAT | negative regulation of cellular protein metabolic process | 25 | 2.60E-04 | 1.20E-02 |
|  | GOTERM_BP_FAT | positive regulation of protein modification process | 26 | 2.80E-04 | 1.20E-02 |
|  | GOTERM_BP_FAT | negative regulation of molecular function | 36 | 4.10E-04 | 1.60E-02 |
|  | GOTERM_BP_FAT | negative regulation of protein metabolic process | 25 | 4.10E-04 | 1.60E-02 |
|  | GOTERM_BP_FAT | regulation of cellular protein metabolic process | 46 | 7.60E-04 | 2.70E-02 |
|  | GOTERM_BP_FAT | negative regulation of catalytic activity | 29 | 1.70E-03 | 5.40E-02 |
|  | GOTERM_BP_FAT | regulation of protein modification process | 32 | 3.20E-03 | 8.80E-02 |
|  | GOTERM_BP_FAT | cell cycle phase | 26 | 1.20E-02 | 2.10E-01 |
|  | SP_PIR_KEYWORDS | ubl conjugation pathway | 21 | 4.00E-02 | 2.90E-01 |
|  | GOTERM_BP_FAT | proteolysis | 60 | 1.50E-01 | 8.00E-01 |
| **Annotation Cluster 7** | **Enrichment Score: 4.53** | **Function** | **Number of Genes** | ***p value*** | **Benjamini** |
|  | UP_SEQ_FEATURE | cross-link:Glycyl lysine isopeptide (Lys-Gly) (interchain with G-Cter in ubiquitin) | 24 | 4.10E-06 | 6.40E-03 |
|  | SP_PIR_KEYWORDS | ubl conjugation | 43 | 7.20E-05 | 1.80E-03 |
|  | SP_PIR_KEYWORDS | isopeptide bond | 28 | 8.80E-05 | 2.00E-03 |
| **Annotation Cluster 8** | **Enrichment Score: 4.44** | **Function** | **Number of Genes** | ***p value*** | **Benjamini** |
|  | GOTERM_BP_FAT | protein transport | 70 | 1.50E-07 | 3.60E-05 |
|  | GOTERM_BP_FAT | establishment of protein localization | 70 | 2.20E-07 | 4.40E-05 |
|  | GOTERM_BP_FAT | protein localization | 79 | 2.70E-07 | 4.50E-05 |
|  | GOTERM_BP_FAT | intracellular transport | 59 | 1.20E-05 | 9.00E-04 |
|  | GOTERM_BP_FAT | cellular protein localization | 39 | 3.10E-04 | 1.30E-02 |
|  | GOTERM_BP_FAT | cellular macromolecule localization | 39 | 3.50E-04 | 1.50E-02 |
|  | GOTERM_BP_FAT | intracellular protein transport | 35 | 6.50E-04 | 2.30E-02 |
|  | GOTERM_BP_FAT | protein targeting | 21 | 2.30E-03 | 6.90E-02 |
|  | SP_PIR_KEYWORDS | protein transport | 32 | 6.40E-03 | 7.70E-02 |
| **Annotation Cluster 9** | **Enrichment Score: 4.37** | **Function** | **Number of Genes** | ***p value*** | **Benjamini** |
|  | GOTERM_BP_FAT | mRNA processing | 36 | 3.50E-07 | 5.20E-05 |
|  | GOTERM_BP_FAT | mRNA metabolic process | 39 | 5.80E-07 | 8.00E-05 |
|  | GOTERM_BP_FAT | RNA splicing | 31 | 2.10E-06 | 2.10E-04 |
|  | GOTERM_BP_FAT | RNA processing | 47 | 1.30E-05 | 9.40E-04 |
|  | KEGG_PATHWAY | Spliceosome | 22 | 7.40E-05 | 2.50E-03 |
|  | SP_PIR_KEYWORDS | mrna processing | 21 | 1.30E-04 | 2.80E-03 |
|  | SP_PIR_KEYWORDS | mrna splicing | 18 | 2.00E-04 | 4.10E-03 |
|  | GOTERM_CC_FAT | spliceosome | 15 | 2.10E-04 | 4.60E-03 |
|  | SP_PIR_KEYWORDS | Spliceosome | 13 | 2.50E-04 | 5.00E-03 |
|  | GOTERM_BP_FAT | RNA splicing, via transesterification reactions | 20 | 4.00E-04 | 1.70E-02 |
|  | GOTERM_BP_FAT | nuclear mRNA splicing, via spliceosome | 20 | 4.00E-04 | 1.70E-02 |
|  | GOTERM_BP_FAT | RNA splicing, via transesterification reactions with bulged adenosine as nucleophile | 20 | 4.00E-04 | 1.70E-02 |
| **Annotation Cluster 10** | **Enrichment Score: 3.92** | **Function** | **Number of Genes** | ***p value*** | **Benjamini** |
|  | GOTERM_BP_FAT | mitotic cell cycle | 42 | 2.40E-09 | 1.80E-06 |
|  | GOTERM_BP_FAT | cell cycle process | 49 | 1.80E-06 | 1.90E-04 |
|  | GOTERM_BP_FAT | cell division | 27 | 3.60E-06 | 3.00E-04 |
|  | SP_PIR_KEYWORDS | cell cycle | 33 | 2.10E-05 | 7.30E-04 |
|  | GOTERM_BP_FAT | mitosis | 19 | 1.00E-04 | 5.40E-03 |
|  | GOTERM_BP_FAT | nuclear division | 19 | 1.00E-04 | 5.40E-03 |
|  | GOTERM_BP_FAT | M phase of mitotic cell cycle | 19 | 1.70E-04 | 7.90E-03 |
|  | GOTERM_BP_FAT | organelle fission | 19 | 2.10E-04 | 9.80E-03 |
|  | SP_PIR_KEYWORDS | cell division | 21 | 3.80E-04 | 6.50E-03 |
|  | GOTERM_BP_FAT | M phase | 22 | 5.50E-03 | 1.30E-01 |
|  | SP_PIR_KEYWORDS | mitosis | 13 | 5.80E-03 | 7.50E-02 |
|  | GOTERM_BP_FAT | cell cycle phase | 26 | 1.20E-02 | 2.10E-01 |
|  | GOTERM_BP_FAT | chromosome segregation | 8 | 5.90E-02 | 5.60E-01 |

**Functional clusters specific to RMC**

| **Annotation Cluster 1** | **Enrichment Score: 4.13** | **Function** | **Number of Genes** | ***p value*** | **Benjamini** |
| --- | --- | --- | --- | --- | --- |
|  | GOTERM_BP_FAT | regulation of membrane potential | 18 | 2.20E-05 | 8.40E-03 |
|  | GOTERM_BP_FAT | axon ensheathment | 10 | 3.30E-05 | 1.10E-02 |
|  | GOTERM_BP_FAT | ensheathment of neurons | 10 | 3.30E-05 | 1.10E-02 |
|  | GOTERM_BP_FAT | regulation of action potential in neuron | 11 | 9.50E-05 | 2.40E-02 |
|  | GOTERM_BP_FAT | regulation of action potential | 12 | 1.20E-04 | 2.60E-02 |
|  | GOTERM_BP_FAT | myelination | 9 | 1.40E-04 | 2.80E-02 |
|  | GOTERM_BP_FAT | transmission of nerve impulse | 23 | 3.50E-04 | 6.00E-02 |
| **Annotation Cluster 2** | **Enrichment Score: 3.44** | **Function** | **Number of Genes** | ***p value*** | **Benjamini** |
|  | GOTERM_CC_FAT | cell projection | 47 | 2.30E-05 | 8.80E-03 |
|  | GOTERM_CC_FAT | neuron projection | 30 | 3.60E-04 | 2.70E-02 |
|  | GOTERM_CC_FAT | dendrite | 17 | 5.70E-03 | 1.80E-01 |
| **Annotation Cluster 3** | **Enrichment Score: 3.33** | **Function** | **Number of Genes** | ***p value*** | **Benjamini** |
|  | GOTERM_BP_FAT | cellular ion homeostasis | 36 | 6.50E-08 | 1.50E-04 |
|  | GOTERM_BP_FAT | cellular chemical homeostasis | 36 | 9.10E-08 | 1.00E-04 |
|  | GOTERM_BP_FAT | ion homeostasis | 36 | 4.90E-07 | 3.70E-04 |
|  | GOTERM_BP_FAT | cellular homeostasis | 37 | 2.90E-06 | 1.70E-03 |
|  | GOTERM_BP_FAT | chemical homeostasis | 39 | 3.00E-06 | 1.40E-03 |
|  | GOTERM_BP_FAT | regulation of membrane potential | 18 | 2.20E-05 | 8.40E-03 |
|  | GOTERM_BP_FAT | homeostatic process | 45 | 1.40E-04 | 2.60E-02 |
|  | GOTERM_BP_FAT | cellular cation homeostasis | 19 | 1.30E-03 | 1.60E-01 |
|  | GOTERM_BP_FAT | cation homeostasis | 19 | 4.50E-03 | 3.10E-01 |
|  | GOTERM_BP_FAT | cellular metal ion homeostasis | 14 | 9.10E-03 | 4.10E-01 |
|  | GOTERM_BP_FAT | metal ion homeostasis | 14 | 1.20E-02 | 4.70E-01 |
|  | GOTERM_BP_FAT | cellular di-, tri-valent inorganic cation homeostasis | 15 | 1.30E-02 | 4.60E-01 |
|  | GOTERM_BP_FAT | di-, tri-valent inorganic cation homeostasis | 15 | 2.00E-02 | 4.90E-01 |
|  | GOTERM_BP_FAT | cellular calcium ion homeostasis | 11 | 5.90E-02 | 7.30E-01 |
|  | GOTERM_BP_FAT | calcium ion homeostasis | 11 | 6.70E-02 | 7.60E-01 |
|  | GOTERM_BP_FAT | cytosolic calcium ion homeostasis | 7 | 2.00E-01 | 9.30E-01 |
|  | GOTERM_BP_FAT | elevation of cytosolic calcium ion concentration | 6 | 2.80E-01 | 9.60E-01 |
| **Annotation Cluster 4** | **Enrichment Score: 2.34** | **Function** | **Number of Genes** | ***p value*** | **Benjamini** |
|  | GOTERM_BP_FAT | oligodendrocyte differentiation | 6 | 1.50E-03 | 1.70E-01 |
|  | GOTERM_BP_FAT | oligodendrocyte development | 5 | 3.00E-03 | 2.70E-01 |
|  | GOTERM_BP_FAT | glial cell development | 6 | 3.40E-03 | 2.80E-01 |
|  | GOTERM_BP_FAT | glial cell differentiation | 8 | 4.10E-03 | 3.00E-01 |
|  | GOTERM_BP_FAT | ensheathment of axons in the central nervous system | 3 | 6.90E-03 | 3.80E-01 |
|  | GOTERM_BP_FAT | myelination in the central nervous system | 3 | 6.90E-03 | 3.80E-01 |
|  | GOTERM_BP_FAT | gliogenesis | 8 | 1.40E-02 | 4.50E-01 |
| **Annotation Cluster 5** | **Enrichment Score: 2.31** | **Function** | **Number of Genes** | ***p value*** | **Benjamini** |
|  | GOTERM_MF_FAT | ion binding | 145 | 2.60E-05 | 1.60E-02 |
|  | GOTERM_MF_FAT | cation binding | 137 | 3.40E-04 | 3.00E-02 |
|  | GOTERM_MF_FAT | metal ion binding | 133 | 8.70E-04 | 6.00E-02 |
|  | GOTERM_MF_FAT | transition metal ion binding | 88 | 7.50E-03 | 2.60E-01 |
|  | GOTERM_MF_FAT | zinc ion binding | 71 | 8.30E-03 | 2.50E-01 |
|  | SP_PIR_KEYWORDS | zinc | 44 | 8.50E-02 | 7.20E-01 |
|  | SP_PIR_KEYWORDS | zinc-finger | 26 | 8.80E-02 | 7.10E-01 |
|  | SP_PIR_KEYWORDS | metal-binding | 65 | 1.00E-01 | 7.00E-01 |
| **Annotation Cluster 6** | **Enrichment Score: 2.21** | **Function** | **Number of Genes** | ***p value*** | **Benjamini** |
|  | INTERPRO | Metallothionein superfamily, eukaryotic | 3 | 5.20E-03 | 8.90E-01 |
|  | INTERPRO | Metallothionein, vertebrate, metal binding site | 3 | 5.20E-03 | 8.90E-01 |
|  | INTERPRO | Metallothionein, vertebrate | 3 | 5.20E-03 | 8.90E-01 |
|  | SP_PIR_KEYWORDS | chelation | 3 | 5.20E-03 | 2.10E-01 |
|  | SP_PIR_KEYWORDS | metal-thiolate cluster | 3 | 5.20E-03 | 2.10E-01 |
|  | PIR_SUPERFAMILY | PIRSF002564:metallothionein | 3 | 5.30E-03 | 6.60E-01 |
|  | UP_SEQ_FEATURE | region of interest:Alpha | 3 | 5.70E-03 | 9.50E-01 |
|  | UP_SEQ_FEATURE | region of interest:Beta | 3 | 5.70E-03 | 9.50E-01 |
|  | UP_SEQ_FEATURE | metal ion-binding site:Divalent metal cation; cluster A | 3 | 5.70E-03 | 9.50E-01 |
|  | UP_SEQ_FEATURE | metal ion-binding site:Divalent metal cation; cluster B | 3 | 5.70E-03 | 9.50E-01 |
|  | SP_PIR_KEYWORDS | metal binding | 3 | 2.40E-02 | 4.40E-01 |
| **Annotation Cluster 7** | **Enrichment Score: 2.12** | **Function** | **Number of Genes** | ***p value*** | **Benjamini** |
|  | GOTERM_CC_FAT | synapse part | 21 | 4.70E-04 | 2.90E-02 |
|  | GOTERM_CC_FAT | postsynaptic membrane | 14 | 7.10E-04 | 3.80E-02 |
|  | GOTERM_CC_FAT | synapse | 26 | 1.00E-03 | 4.30E-02 |
|  | GOTERM_MF_FAT | extracellular ligand-gated ion channel activity | 8 | 9.70E-03 | 2.70E-01 |
|  | GOTERM_CC_FAT | cell junction | 24 | 2.30E-02 | 3.30E-01 |
|  | SP_PIR_KEYWORDS | postsynaptic cell membrane | 10 | 3.00E-02 | 4.30E-01 |
|  | SP_PIR_KEYWORDS | synapse | 14 | 5.30E-02 | 5.70E-01 |
|  | SP_PIR_KEYWORDS | cell junction | 18 | 8.60E-02 | 7.10E-01 |
| **Annotation Cluster 8** | **Enrichment Score: 2.1** | **Function** | **Number of Genes** | ***p value*** | **Benjamini** |
|  | GOTERM_BP_FAT | regulation of synaptic plasticity | 11 | 4.70E-04 | 7.40E-02 |
|  | GOTERM_BP_FAT | regulation of neuronal synaptic plasticity | 8 | 1.40E-03 | 1.70E-01 |
|  | GOTERM_BP_FAT | regulation of synaptic transmission | 13 | 1.20E-02 | 4.60E-01 |
|  | GOTERM_BP_FAT | regulation of transmission of nerve impulse | 13 | 2.00E-02 | 5.00E-01 |
|  | GOTERM_BP_FAT | regulation of neurological system process | 13 | 3.10E-02 | 5.90E-01 |
|  | GOTERM_BP_FAT | regulation of system process | 18 | 5.20E-02 | 7.00E-01 |
| **Annotation Cluster 9** | **Enrichment Score: 2.08** | **Function** | **Number of Genes** | ***p value*** | **Benjamini** |
|  | GOTERM_MF_FAT | cytoskeletal protein binding | 27 | 2.20E-03 | 1.10E-01 |
|  | GOTERM_CC_FAT | actin cytoskeleton | 15 | 1.20E-02 | 2.30E-01 |
|  | GOTERM_MF_FAT | actin binding | 16 | 2.30E-02 | 4.00E-01 |
| **Annotation Cluster 10** | **Enrichment Score: 1.92** | **Function** | **Number of Genes** | ***p value*** | **Benjamini** |
|  | SP_PIR_KEYWORDS | ionic channel | 28 | 1.20E-05 | 4.20E-03 |
|  | GOTERM_MF_FAT | ion channel activity | 29 | 3.60E-05 | 1.20E-02 |
|  | GOTERM_MF_FAT | substrate specific channel activity | 29 | 6.10E-05 | 1.30E-02 |
|  | GOTERM_BP_FAT | ion transport | 44 | 6.30E-05 | 1.80E-02 |
|  | GOTERM_MF_FAT | channel activity | 29 | 1.20E-04 | 1.80E-02 |
|  | GOTERM_MF_FAT | passive transmembrane transporter activity | 29 | 1.20E-04 | 1.80E-02 |
|  | GOTERM_MF_FAT | gated channel activity | 24 | 1.20E-04 | 1.60E-02 |
|  | GOTERM_MF_FAT | ligand-gated ion channel activity | 14 | 1.50E-04 | 1.60E-02 |
|  | GOTERM_MF_FAT | ligand-gated channel activity | 14 | 1.50E-04 | 1.60E-02 |
|  | GOTERM_CC_FAT | ion channel complex | 16 | 1.60E-04 | 1.50E-02 |
|  | SP_PIR_KEYWORDS | ion transport | 34 | 8.80E-04 | 7.60E-02 |
|  | GOTERM_MF_FAT | cation channel activity | 20 | 1.80E-03 | 1.00E-01 |
|  | GOTERM_MF_FAT | metal ion transmembrane transporter activity | 21 | 5.50E-03 | 2.20E-01 |
|  | INTERPRO | Potassium channel, voltage dependent, Kv3 | 3 | 1.00E-02 | 9.40E-01 |
|  | PIR_SUPERFAMILY | PIRSF500973:shaw-related voltage-gated potassium channel, alpha subunit | 3 | 1.00E-02 | 5.10E-01 |
|  | GOTERM_BP_FAT | metal ion transport | 24 | 1.30E-02 | 4.50E-01 |
|  | SP_PIR_KEYWORDS | voltage-gated channel | 12 | 1.40E-02 | 3.20E-01 |
|  | UP_SEQ_FEATURE | short sequence motif:Selectivity filter | 7 | 1.80E-02 | 9.30E-01 |
|  | GOTERM_BP_FAT | cation transport | 27 | 2.20E-02 | 5.10E-01 |
|  | GOTERM_CC_FAT | cation channel complex | 8 | 3.90E-02 | 4.00E-01 |
|  | INTERPRO | Ion transport | 9 | 4.00E-02 | 9.80E-01 |
|  | GOTERM_MF_FAT | alkali metal ion binding | 13 | 4.30E-02 | 5.40E-01 |
|  | GOTERM_MF_FAT | voltage-gated cation channel activity | 10 | 4.50E-02 | 5.50E-01 |
|  | GOTERM_BP_FAT | monovalent inorganic cation transport | 17 | 4.50E-02 | 6.70E-01 |
|  | GOTERM_MF_FAT | voltage-gated ion channel activity | 12 | 4.70E-02 | 5.60E-01 |
|  | GOTERM_MF_FAT | voltage-gated channel activity | 12 | 4.70E-02 | 5.60E-01 |
|  | INTERPRO | Potassium channel, voltage dependent, Kv | 4 | 5.00E-02 | 9.80E-01 |
|  | GOTERM_CC_FAT | T-tubule | 4 | 5.60E-02 | 4.90E-01 |
|  | INTERPRO | Voltage-dependent potassium channel | 4 | 7.90E-02 | 9.80E-01 |
|  | GOTERM_MF_FAT | voltage-gated potassium channel activity | 7 | 9.20E-02 | 7.00E-01 |
|  | GOTERM_MF_FAT | potassium ion binding | 8 | 1.00E-01 | 7.20E-01 |
|  | SP_PIR_KEYWORDS | potassium transport | 8 | 1.00E-01 | 6.90E-01 |
|  | PIR_SUPERFAMILY | PIRSF002449:potassium voltage-gated channel, alpha subunit, subfamilies A/C/D/F/G/S | 3 | 1.00E-01 | 9.90E-01 |
|  | SP_PIR_KEYWORDS | potassium channel | 6 | 1.20E-01 | 6.90E-01 |
|  | INTERPRO | Potassium channel, voltage dependent, Kv, tetramerisation | 4 | 1.30E-01 | 9.90E-01 |
|  | GOTERM_MF_FAT | potassium channel activity | 8 | 1.50E-01 | 8.10E-01 |
|  | SP_PIR_KEYWORDS | potassium | 8 | 1.50E-01 | 7.40E-01 |
|  | GOTERM_BP_FAT | potassium ion transport | 8 | 2.20E-01 | 9.40E-01 |
|  | SP_PIR_KEYWORDS | transport | 50 | 2.40E-01 | 8.50E-01 |
|  | INTERPRO | BTB/POZ fold | 6 | 3.00E-01 | 1.00E+00 |
|  | GOTERM_CC_FAT | voltage-gated potassium channel complex | 4 | 3.00E-01 | 8.00E-01 |
|  | GOTERM_CC_FAT | potassium channel complex | 4 | 3.00E-01 | 8.00E-01 |
|  | INTERPRO | BTB/POZ-like | 6 | 3.10E-01 | 1.00E+00 |
|  | SMART | BTB | 6 | 4.50E-01 | 1.00E+00 |
