# Supplementary material for: Transcriptome analysis of amoeboid and ramified microglia isolated from the corpus callosum of rat brain
Source: BMC Neurosci. 2012 Jun 14;13:64. doi: 10.1186/1471-2202-13-64 (PMC3441342; doi:10.1186/1471-2202-13-64)
Supplement: Additional file 5 — Sheet S4. Genes involved in CDC42-RAC pathway for migration, Synaptic transmission and genes downregulated by the Runx1-Runx1t1 complex. [file 1471-2202-13-64-S5.docx]

|  | **Description: Genes involved in CDC42-RAC pathway for migration were found to be specific to AMC** |  |
| --- | --- | --- |
| **Gene Symbol** | **Gene Name** | **Fold Change** |
| Rac1 | ras-related C3 botulinum toxin substrate 1 (rho family, small GTP binding protein Rac1) | 5.5863 |
| Cdc42 | cell division cycle 42 (GTP binding protein, 25kDa) | 5.1257 |
| Actr2 | ARP2 actin-related protein 2 homolog (yeast) | 4.6831 |
| Actr3 | ARP3 actin-related protein 3 homolog (yeast) | 3.8936 |
| Arpc5 | actin related protein 2/3 complex, subunit 5, 16kDa | 3.4395 |
| Pik3ca | phosphoinositide-3-kinase, catalytic, alpha polypeptide | 2.4225 |
| Pdgfra | platelet-derived growth factor receptor, alpha polypeptide | 2.5694 |

|  | **Description: Genes involved in synaptic transmission were found to be specific to RMC** |  |
| --- | --- | --- |
| **Gene Symbol** | **Gene Name** | **Fold Change** |
| Agt | angiotensinogen (serpin peptidase inhibitor, clade A, member 8) | 6.3234 |
| Grin2c | glutamate receptor, ionotropic, N-methyl D-aspartate 2C | 5.9695 |
| Cpeb1 | cytoplasmic polyadenylation element binding protein 1 | 4.8865 |
| Camk2a | calcium/calmodulin-dependent protein kinase II alpha | 4.6656 |
| S100b | S100 calcium binding protein B | 3.3308 |
| Arc | activity-regulated cytoskeleton-associated protein | 3.3203 |
| Cabc1 | presenilin 2; chaperone, ABC1 activity of bc1 complex homolog (S. pombe) | 3.2619 |
| Egr1 | early growth response 1 | 3.2473 |
| Rnf39 | ring finger protein 39 | 3.2455 |
| Grm8 | glutamate receptor, metabotropic 8 | 2.386 |
| Plk3 | polo-like kinase 3 (Drosophila) | 2.079 |
| Sipa1l1 | signal-induced proliferation-associated 1 like 1 | 2.0713 |
| Pink1 | PTEN induced putative kinase 1 | 2.034 |

|  | **Description: Genes downregulated by the Runx1-Runx1t1 complex were found to be specific to RMC** |  |
| --- | --- | --- |
| **Gene Symbol** | **Gene Name** | **Fold Change** |
| Mbp | myelin basic protein | 21.571 |
| Nefh | neurofilament, heavy polypeptide 200kDa | 4.2066 |
| S100B | S100 calcium binding protein B | 3.3308 |
| Csf1 | colony stimulating factor 1 (macrophage) | 2.8628 |
| Bin2 | bridging integrator 2 | 2.5676 |
| Prkcd | protein kinase C, delta | 2.432 |
| Tnfsf13 | tumor necrosis factor (ligand) superfamily, member 13 | 2.414 |
| Egr3 | early growth response 3 | 2.1875 |
| Runx3 | runt-related transcription factor 3 | 2.1139 |
| Socs1 | suppressor of cytokine signaling 1 | 2.0005 |
