# Supplementary material for: Transcriptome analysis of amoeboid and ramified microglia isolated from the corpus callosum of rat brain
Source: BMC Neurosci. 2012 Jun 14;13:64. doi: 10.1186/1471-2202-13-64 (PMC3441342; doi:10.1186/1471-2202-13-64)
Supplement: Additional file 6 — Sheet S5. Functions of AMC and RMC. [file 1471-2202-13-64-S6.docx]

|  |  | **AMC specific genes involved in cell proliferation** |  |
| --- | --- | --- | --- |
| **Probe ID** | **Gene Symbol** | **Gene Title** | **Fold Change** |
| 1384000_at | Sox4 | SRY (sex determining region Y)-box 4 | 28.328 |
| 1369953_a_at | Cd24 | CD24 molecule | 9.2544 |
| 1376648_at | Mycn | v-myc myelocytomatosis viral related oncogene, neuroblastoma derived (avian) | 8.6884 |
| 1390386_at | Casp3 | caspase 3, apoptosis related cysteine protease | 5.3212 |
| 1369944_at | Marcksl1 | MARCKS-like 1 | 5.254 |
| 1369242_at | Pax6 | paired box 6 | 5.123 |
| 1374279_at | Scye1 | small inducible cytokine subfamily E, member 1 | 4.7907 |
| 1373200_at | Eef1e1 | eukaryotic translation elongation factor 1 epsilon 1 | 4.7568 |
| 1368214_at | Smad2 | SMAD family member 2 | 4.7514 |
| 1370478_at | Myo16 | myosin XVI | 4.4391 |
| 1371202_a_at | Nfib | nuclear factor I/B | 4.2932 |
| 1367604_at | Crip2 | cysteine-rich protein 2 | 4.1602 |
| 1368042_a_at | Hmg1l1 | high-mobility group (nonhistone chromosomal) protein 1-like 1 | 3.9596 |
| 1368308_at | Myc | myelocytomatosis oncogene | 3.835 |
| 1374776_at | Vash2 | vasohibin 2 | 3.7308 |
| 1370213_at | Ybx1 | Y box binding protein 1 | 3.7191 |
| 1368189_at | Dhcr7 | 7-dehydrocholesterol reductase | 3.6913 |
| 1375719_s_at | Cdh13 | cadherin 13 | 3.5609 |
| 1368870_at | Id2 | inhibitor of DNA binding 2 | 3.524 |
| 1377661_at | Frs2 | fibroblast growth factor receptor substrate 2 | 3.4399 |
| 1379582_a_at | Ccna2 | cyclin A2 | 3.2889 |
| 1383075_at | Ccnd1 | cyclin D1 | 3.201 |
| 1398757_at | Npm1 | nucleophosmin (nucleolar phosphoprotein B23, numatrin) | 3.1793 |
| 1369964_at | Coro1a | coronin, actin binding protein 1A | 3.146 |
| 1370909_at | Nup62 | nucleoporin 62 | 3.128 |
| 1374304_at | Xrcc4 | X-ray repair complementing defective repair in Chinese hamster cells 4 | 3.0992 |
| 1370908_at | Hdac2 | histone deacetylase 2 | 3.0713 |
| 1373369_at | Zmiz1 | zinc finger, MIZ-type containing 1 | 3.044 |
| 1389528_s_at | Jun | Jun oncogene | 3.0095 |
| 1369559_a_at | Cd47 | Cd47 molecule | 2.843 |
| 1388802_at | Bex1 | brain expressed gene 1 | 2.7963 |
| 1367734_at | Akr1b1 | aldo-keto reductase family 1, member B1 (aldose reductase) | 2.779 |
| 1393058_at | Eid2 | EP300 interacting inhibitor of differentiation 2 | 2.7084 |
| 1380168_at | Etv4 | ets variant 4 | 2.6701 |
| 1368945_at | Bmp2 | bone morphogenetic protein 2 | 2.6681 |
| 1388314_at | Hmgn1 | high-mobility group nucleosome binding domain 1 | 2.5724 |
| 1370941_at | Pdgfra | platelet derived growth factor receptor, alpha polypeptide | 2.5694 |
| 1368032_at | Nolc1 | nucleolar and coiled-body phosphoprotein 1 | 2.4885 |
| 1367609_at | Mif | macrophage migration inhibitory factor | 2.4869 |
| 1387769_a_at | Id3 | inhibitor of DNA binding 3 | 2.3788 |
| 1369950_at | Cdk4 | cyclin-dependent kinase 4 | 2.3458 |
| 1387888_at | Rps9 | ribosomal protein S9 | 2.3402 |
| 1371592_at | Csk | c-src tyrosine kinase | 2.3096 |
| 1372122_at | Tsg101 | tumor susceptibility gene 101 | 2.2867 |
| 1386881_at | Igfbp3 | insulin-like growth factor binding protein 3 | 2.2773 |
| 1395429_at | Chrna7 | Cholinergic receptor, nicotinic, alpha 7 | 2.2716 |
| 1396100_at | Aggf1 | angiogenic factor with G patch and FHA domains 1 | 2.2678 |
| 1372757_at | Stat1 | signal transducer and activator of transcription 1 | 2.2596 |
| 1367766_at | Nme2 | non-metastatic cells 2, protein (NM23B) expressed in | 2.2229 |
| 1373824_at | Cfdp1 | craniofacial development protein 1 | 2.194 |
| 1373315_at | Arnt2 | aryl hydrocarbon receptor nuclear translocator 2 | 2.1418 |
| 1367831_at | Tp53 | tumor protein p53 | 2.1165 |
| 1367776_at | Cdc2 | cell division cycle 2, G1 to S and G2 to M | 2.0803 |
| 1389213_at | Vash1 | vasohibin 1 | 2.0244 |
| 1379651_at | Foxp1 | Forkhead box P1 | 2.0145 |

|  |  | **RMC specific genes involved in cell proliferation** |  |
| --- | --- | --- | --- |
| **Probe ID** | **Gene Symbol** | **Gene Title** | **Fold Change** |
| 1387811_at | Agt | angiotensinogen (serpin peptidase inhibitor, clade A, member 8) | 6.3234 |
| 1384202_at | Tesc | tescalcin | 5.6757 |
| 1385074_at | Smarca2 | SWI/SNF related, matrix associated, actin dependent regulator of chromatin, subfamily a, member 2 | 3.663 |
| 1368451_at | Hrh3 | histamine receptor H3 | 3.5485 |
| 1395419_at | Mll1 | myeloid/lymphoid or mixed-lineage leukemia 1 | 2.9906 |
| 1393836_at | Mitf | microphthalmia-associated transcription factor | 2.9697 |
| 1387874_at | Dbp | D site of albumin promoter (albumin D-box) binding protein | 2.9682 |
| 1380582_at | Csf1 | colony stimulating factor 1 (macrophage) | 2.8628 |
| 1377288_at | Hsf4 | heat shock transcription factor 4 | 2.5857 |
| 1379677_at | Tnfsf13 | tumor necrosis factor (ligand) superfamily, member 13 | 2.414 |
| 1368553_at | Acvrl1 | activin A receptor type II-like 1 | 2.4091 |
| 1372260_at | Rogdi | rogdi homolog (Drosophila) | 2.2851 |
| 1388241_at | Insl3 | insulin-like 3 | 2.1941 |
| 1370649_at | Bdkrb2 /// RGD1308470 | bradykinin receptor B2 /// similar to RIKEN cDNA 4933433P14 gene | 2.1876 |
| 1369101_at | Rxra | retinoid X receptor alpha | 2.1608 |
| 1387479_at | Runx3 | runt-related transcription factor 3 | 2.1139 |
| 1393067_at | Tek | TEK tyrosine kinase, endothelial | 2.1025 |
| 1374741_at | Esrra | estrogen related receptor, alpha | 2.1 |
| 1368186_a_at | Syk | spleen tyrosine kinase | 2.0797 |
| 1387776_at | Tgm2 | transglutaminase 2, C polypeptide | 2.0755 |
| 1387437_at | Fbxo2 | F-box protein 2 | 2.0622 |
| 1392749_at | Cntfr | ciliary neurotrophic factor receptor | 2.0575 |
| 1396820_at | Hdac1 | histone deacetylase 1 | 2.0538 |
| 1369158_at | Casr | calcium-sensing receptor | 2.0507 |
| 1368072_at | Btg3 | B-cell translocation gene 3 | 2.0372 |
| 1369618_at | Il13 | interleukin 13 | 2.0364 |
| 1387136_at | Ptprv | protein tyrosine phosphatase, receptor type, V | 2.0303 |
| 1381012_at | Serpinf1 | serine (or cysteine) peptidase inhibitor, clade F, member 1 | 2.001 |

|  |  | **AMC specific genes involved in cell cycle** |  |
| --- | --- | --- | --- |
| **Probe ID** | **Gene Symbol** | **Gene Name** | **Fold Change** |
| 1390672_at | Rprm | reprimo, TP53 dependent G2 arrest mediator candidate | 19.125 |
| 1371074_a_at | Mcm6 | minichromosome maintenance complex component 6 | 8.1985 |
| 1381042_at | Anapc10 | anaphase promoting complex subunit 10 | 8.066 |
| 1370290_at | Tubb5 | tubulin, beta 5 | 7.3307 |
| 1387892_at | Tubb5 | tubulin, beta 5 | 7.1734 |
| 1375525_at | Mapre1 | microtubule-associated protein, RP/EB family, member 1; similar to Microtubule-associated protein RP/EB family member 1 (APC-binding protein EB1) (End-binding protein 1) (EB1) | 7.0496 |
| 1388582_at | Psme3 | similar to proteaseome (prosome, macropain) 28 subunit, 3; proteasome (prosome, macropain) activator subunit 3 | 7.0045 |
| 1383192_at | Spast | spastin | 6.988 |
| 1382385_at | Psmc6 | proteasome (prosome, macropain) 26S subunit, ATPase, 6; similar to Psmc6 protein | 6.4896 |
| 1378264_at | Nasp | nuclear autoantigenic sperm protein (histone-binding); similar to nuclear autoantigenic sperm protein | 5.2995 |
| 1390259_at | Ube2d1 | ubiquitin-conjugating enzyme E2D 1, UBC4/5 homolog (yeast) | 5.0533 |
| 1383940_at | Nuf2 | NUF2, NDC80 kinetochore complex component, homolog (S. cerevisiae) | 4.9688 |
| 1398756_at | Npm1 | nucleophosmin (nucleolar phosphoprotein B23, numatrin); similar to Nucleophosmin (NPM) (Nucleolar phosphoprotein B23) (Numatrin) (Nucleolar protein NO38) | 4.9399 |
| 1369408_at | Dbc1 | deleted in bladder cancer 1 (human) | 4.8326 |
| 1386857_at | Stmn1 | stathmin 1 | 4.7384 |
| 1373658_at | Racgap1 | Rac GTPase-activating protein 1 | 4.6191 |
| 1387026_at | Smc1a | structural maintenance of chromosomes 1A | 4.4069 |
| 1384392_at | Cyp26b1 | cytochrome P450, family 26, subfamily b, polypeptide 1 | 4.3134 |
| 1379261_at | Ska2 | hypothetical protein LOC691962; hypothetical protein LOC687897; family with sequence similarity 33, member A | 4.0094 |
| 1398773_at | Khdrbs1 | KH domain containing, RNA binding, signal transduction associated 1 | 3.9389 |
| 1371618_s_at | Tubb3 | tubulin, beta 3 | 3.8563 |
| 1367663_at | Psme1 | proteasome (prosome, macropain) activator subunit 1 | 3.6823 |
| 1381153_at | Anapc4 | anaphase promoting complex subunit 4 | 3.5894 |
| 1376065_at | Rrs1 | RRS1 ribosome biogenesis regulator homolog (S. cerevisiae) | 3.571 |
| 1372949_at | Sept11 | septin 11 | 3.5036 |
| 1371928_at | Cdca8 | cell division cycle associated 8 | 3.4068 |
| 1370250_at | Ube2i | transmembrane protein 215; similar to Ubiquitin-conjugating enzyme E2 I (Ubiquitin-protein ligase I) (Ubiquitin carrier protein I) (SUMO-1-protein ligase) (SUMO-1-conjugating enzyme) (Ubiquitin-conjugating enzyme UbcE2A); ubiquitin-conjugating enzyme E2I | 3.3128 |
| 1379582_a_at | Ccna2 | cyclin A2 | 3.2889 |
| 1367590_at | Ran | RAN, member RAS oncogene family | 3.2657 |
| 1389566_at | Ccnb2 | cyclin B2 | 3.2038 |
| 1383075_at | Ccnd1 | cyclin D1 | 3.201 |
| 1398786_at | Psmb2 | proteasome (prosome, macropain) subunit, beta type 2 | 3.1812 |
| 1398757_at | Npm1 | nucleophosmin (nucleolar phosphoprotein B23, numatrin); similar to Nucleophosmin (NPM) (Nucleolar phosphoprotein B23) (Numatrin) (Nucleolar protein NO38) | 3.1793 |
| 1383269_at | Rnf2 | ring finger protein 2 | 3.1366 |
| 1371851_at | Psmd6 | proteasome (prosome, macropain) 26S subunit, non-ATPase, 6 | 3.1318 |
| 1368002_at | Msh2 | mutS homolog 2 (E. coli) | 3.1126 |
| 1370393_at | Haus1 | coiled-coil domain containing 5 | 3.0948 |
| 1370246_at | Calm2 | calmodulin pseudogene 2; calmodulin 3; calmodulin 2; calmodulin 1 | 3.0685 |
| 1399158_a_at | Npm1 | nucleophosmin (nucleolar phosphoprotein B23, numatrin); similar to Nucleophosmin (NPM) (Nucleolar phosphoprotein B23) (Numatrin) (Nucleolar protein NO38) | 3.0648 |
| 1374565_at | Nek6 | NIMA (never in mitosis gene a)-related kinase 6 | 2.9406 |
| 1398778_at | Psma1 | proteasome (prosome, macropain) subunit, alpha type 1 | 2.9127 |
| 1399097_at | --- | similar to spindlin; spindlin 1 | 2.8794 |
| 1387772_at | Calm1 | calmodulin pseudogene 2; calmodulin 3; calmodulin 2; calmodulin 1 | 2.8775 |
| 1388514_at | Ppm1g | protein phosphatase 1G (formerly 2C), magnesium-dependent, gamma isoform | 2.8138 |
| 1385090_at | Rad17 | RAD17 homolog (S. pombe) | 2.8077 |
| 1368508_at | LOC100361067 /// Psma3 /// Psma3l | proteasome (prosome, macropain) subunit, alpha type 3; proteasome subunit alpha type 3-like; similar to Proteasome subunit alpha type 3 (Proteasome component C8) (Macropain subunit C8) (Multicatalytic endopeptidase complex subunit C8) (Proteasome subunit K) | 2.7971 |
| 1370034_at | Cdc25b | cell division cycle 25 homolog B (S. pombe) | 2.7607 |
| 1384280_at | Nusap1 | nucleolar and spindle associated protein 1 | 2.7393 |
| 1398813_at | Uba3 | ubiquitin-like modifier activating enzyme 3 | 2.7254 |
| 1371684_at | Pelo | pelota homolog (Drosophila) | 2.7203 |
| 1393041_at | Smc2 | structural maintenance of chromosomes 2 | 2.7164 |
| 1384323_at | Psmc6 | proteasome (prosome, macropain) 26S subunit, ATPase, 6; similar to Psmc6 protein | 2.6092 |
| 1370803_at | Zwint | ZW10 interactor | 2.5857 |
| 1383126_at | Akt1 | v-akt murine thymoma viral oncogene homolog 1 | 2.5848 |
| 1398831_at | Psmb4 | proteasome (prosome, macropain) subunit, beta type 4 | 2.5649 |
| 1368273_at | Mapk6 | mitogen-activated protein kinase 6 | 2.5004 |
| 1383101_at | Anapc10 | anaphase promoting complex subunit 10 | 2.4138 |
| 1370116_at | Sept3 | septin 3 | 2.3462 |
| 1369950_at | Cdk4 | cyclin-dependent kinase 4 | 2.3458 |
| 1386913_at | Pdpn | podoplanin | 2.3404 |
| 1374428_at | Kif3b | kinesin family member 3B | 2.3164 |
| 1376951_at | Mad2l1 | MAD2 (mitotic arrest deficient, homolog)-like 1 (yeast) | 2.2948 |
| 1372122_at | Tsg101 | tumor susceptibility gene 101 | 2.2867 |
| 1398405_at | Sept6 | NFKB repressing factor; septin 6 | 2.2675 |
| 1371480_at | Cks1b | similar to Cyclin-dependent kinases regulatory subunit 1 (CKS-1) (Sid 1334); RGD1561797 | 2.1804 |
| 1387801_at | Ppp6c | protein phosphatase 6, catalytic subunit | 2.1596 |
| 1398858_at | Psmd2 | proteasome (prosome, macropain) 26S subunit, non-ATPase, 2 | 2.1413 |
| 1387884_at | Psma5 | proteasome (prosome, macropain) subunit, alpha type 5 | 2.1221 |
| 1367831_at | Tp53 | tumor protein p53 | 2.1165 |
| 1367698_a_at | Sept9 | septin 9 | 2.1059 |
| 1374516_at | Chtf8 | CTF8, chromosome transmission fidelity factor 8 homolog (S. cerevisiae) | 2.1014 |
| 1367776_at | Cdc2 | cell division cycle 2, G1 to S and G2 to M | 2.0803 |
| 1367837_at | Psma4 | proteasome (prosome, macropain) subunit, alpha type 4 | 2.0768 |
| 1369992_at | Psmd1 | proteasome (prosome, macropain) 26S subunit, non-ATPase, 1 | 2.0534 |
| 1388386_at | Chmp1a | chromatin modifying protein 1A | 2.0489 |
| 1388474_at | Ube2i | transmembrane protein 215; similar to Ubiquitin-conjugating enzyme E2 I (Ubiquitin-protein ligase I) (Ubiquitin carrier protein I) (SUMO-1-protein ligase) (SUMO-1-conjugating enzyme) (Ubiquitin-conjugating enzyme UbcE2A); ubiquitin-conjugating enzyme E2I | 2.0447 |
| 1370274_at | LOC100360548 /// Ubb | similar to polyubiquitin; ubiquitin C; ubiquitin B | 2.0076 |

|  |  | **RMC specific genes involved in cell cycle** |  |
| --- | --- | --- | --- |
| **Probe ID** | **Gene Symbol** | **Gene Name** | **Fold Change** |
| 1389448_at | Sept4 | septin 4 | 6.0436 |
| 1392996_at | Cpeb1 | cytoplasmic polyadenylation element binding protein 1 | 4.8865 |
| 1388187_at | Camk2a | calcium/calmodulin-dependent protein kinase II alpha | 4.6656 |
| 1386296_at | --- | septin 8 | 3.8265 |
| 1380405_at | Sept8 | septin 8 | 3.7375 |
| 1389824_at | Camk2a | calcium/calmodulin-dependent protein kinase II alpha | 3.5972 |
| 1381637_at | Camk2a | calcium/calmodulin-dependent protein kinase II alpha | 3.524 |
| 1398297_at | Mapk12 | mitogen-activated protein kinase 12 | 2.9091 |
| 1374062_x_at | Mapre3 | microtubule-associated protein, RP/EB family, member 3 | 2.894 |
| 1380989_at | Numa1 | nuclear mitotic apparatus protein 1 | 2.7358 |
| 1393723_at | Plk5 | polo-like kinase 5 pseudogene | 2.5451 |
| 1369590_a_at | Ddit3 | DNA-damage inducible transcript 3 | 2.1394 |
| 1393155_at | Plk3 | polo-like kinase 3 (Drosophila) | 2.079 |

|  |  | **AMC specific genes involved in cell death** |  |
| --- | --- | --- | --- |
| **Probe ID** | **Gene Symbol** | **Gene Name** | **Fold Change** |
| 1369953_a_at | Cd24 | CD24 molecule | 9.2544 |
| 1369197_at | Apaf1 | apoptotic peptidase activating factor 1 | 6.6501 |
| 1387111_at | Ddah1 | B-cell CLL/lymphoma 10; dimethylarginine dimethylaminohydrolase 1 | 6.1822 |
| 1372513_at | Rac1 | ras-related C3 botulinum toxin substrate 1 | 5.5863 |
| 1390386_at | Casp3 | caspase 3, apoptosis related cysteine protease | 5.3212 |
| 1369408_at | Dbc1 | deleted in bladder cancer 1 (human) | 4.8326 |
| 1367890_at | Casp2 | caspase 2 | 4.1832 |
| 1387142_at | Polb | polymerase (DNA directed), beta | 4.0578 |
| 1371883_at | Mmd | monocyte to macrophage differentiation-associated | 3.9032 |
| 1368308_at | Myc | myelocytomatosis oncogene | 3.835 |
| 1398851_at | Ywhae | tyrosine 3-monooxygenase/tryptophan 5-monooxygenase activation protein, epsilon polypeptide | 3.2796 |
| 1370044_at | Faim | Fas apoptotic inhibitory molecule | 3.1639 |
| 1370909_at | Nup62 | nucleoporin 62 | 3.128 |
| 1368002_at | Msh2 | mutS homolog 2 (E. coli) | 3.1126 |
| 1367557_s_at | Gapdh | similar to glyceraldehyde-3-phosphate dehydrogenase (phosphorylating) (EC 1.2.1.12) | 3.0318 |
| 1385534_at | Ngfrap1 | nerve growth factor receptor (TNFRSF16) associated protein 1 | 3.0174 |
| 1389528_s_at | Jun | Jun oncogene | 3.0095 |
| 1368137_at | Mapt | microtubule-associated protein tau | 2.9792 |
| 1388332_at | Rac1 | ras-related C3 botulinum toxin substrate 1 | 2.9728 |
| 1374565_at | Nek6 | NIMA (never in mitosis gene a)-related kinase 6 | 2.9406 |
| 1372520_at | Mcl1 | myeloid cell leukemia sequence 1 | 2.8383 |
| 1388767_at | Pdcd6 | programmed cell death 6 | 2.5972 |
| 1384615_at | Luc7l3 | cisplatin resistance-associated overexpressed protein | 2.596 |
| 1383126_at | Akt1 | v-akt murine thymoma viral oncogene homolog 1 | 2.5848 |
| 1383491_at | Aen | apoptosis enhancing nuclease | 2.4961 |
| 1389325_at | Pdcd10 | programmed cell death 10 | 2.434 |
| 1369066_at | Madd | MAP-kinase activating death domain | 2.3363 |
| 1371498_at | Aimp2 | JTV1 gene | 2.3333 |
| 1388979_at | Smndc1 | survival motor neuron domain containing 1 | 2.2793 |
| 1389381_at | Sqstm1 | sequestosome 1 | 2.2461 |
| 1371876_at | Psmg2 | proteasome (prosome, macropain) assembly chaperone 2 | 2.2433 |
| 1367831_at | Tp53 | tumor protein p53 | 2.1165 |
| 1380548_at | Ift57 | intraflagellar transport 57 homolog (Chlamydomonas) | 2.0491 |
| 1388723_at | Bre | brain and reproductive organ-expressed protein | 2.0428 |
| 1368138_at | Mapt | microtubule-associated protein tau | 2.034 |
| 1398800_at | Ywhab | tyrosine 3-monooxygenase/tryptophan 5-monooxygenase activation protein, beta polypeptide | 2.0295 |
| 1370274_at | LOC100360548 /// Ubb | similar to polyubiquitin; ubiquitin C; ubiquitin B | 2.0076 |

|  |  | **RMC specific genes involved in cell death** |  |
| --- | --- | --- | --- |
| **Probe ID** | **Gene Symbol** | **Gene Name** | **Fold Change** |
| 1381922_at | Slc5a11 | solute carrier family 5 (sodium/glucose cotransporter), member 11 | 12.343 |
| 1371414_at | Gsn | gelsolin | 3.9717 |
| 1374363_at | Aifm3 | apoptosis-inducing factor, mitochondrion-associated 3 | 3.547 |
| 1372536_at | Cabc1 | presenilin 2; chaperone, ABC1 activity of bc1 complex homolog (S. pombe) | 3.2619 |
| 1382055_at | Rtkn | rhotekin | 2.6178 |
| 1382757_at | --- | forkhead box L2 | 2.4773 |
| 1379677_at | Tnfsf13 | tumor necrosis factor (ligand) superfamily, member 13 | 2.414 |
| 1372930_at | Sp110 | SP110 nuclear body protein | 2.3595 |
| 1371785_at | Tnfrsf12a | tumor necrosis factor receptor superfamily, member 12a | 2.3378 |
| 1394481_at | Ube2z | ubiquitin-conjugating enzyme E2Z | 2.3277 |
| 1385148_at | P2rx1 | purinergic receptor P2X, ligand-gated ion channel, 1 | 2.3229 |
| 1374995_at | Elmo3 | engulfment and cell motility 3 | 2.2129 |
| 1385333_at | Aatk | apoptosis-associated tyrosine kinase | 2.2009 |
| 1369889_at | Ifnb1 | interferon beta 1, fibroblast | 2.1702 |
| 1375613_at | Gata6 | GATA binding protein 6 | 2.1637 |
| 1383890_at | Hip1 | huntingtin interacting protein 1 | 2.0956 |
| 1386998_at | Aldoc | aldolase C, fructose-bisphosphate | 2.077 |

|  |  | **AMC specific genes involved in cell migration** |  |
| --- | --- | --- | --- |
| **Probe ID** | **Gene Symbol** | **Gene Name** | **Fold Change** |
| 1390838_at | --- | SATB homeobox 2 | 18.049 |
| 1374966_at | Dcx | doublecortin | 16.642 |
| 1373661_a_at | Cxcr4 | chemokine (C-X-C motif) receptor 4 | 12.759 |
| 1389244_x_at | Cxcr4 | chemokine (C-X-C motif) receptor 4 | 12.274 |
| 1387871_at | --- | cofilin 1, non-muscle; similar to Cofilin-1 (Cofilin, non-muscle isoform) | 9.3235 |
| 1369953_a_at | Cd24 | CD24 molecule | 9.2544 |
| 1374638_at | Pex13 | peroxisomal biogenesis factor 13 | 8.4726 |
| 1372513_at | Rac1 | ras-related C3 botulinum toxin substrate 1 | 5.5863 |
| 1369242_at | Pax6 | paired box 6 | 5.123 |
| 1374279_at | Aimp1 | small inducible cytokine subfamily E, member 1 | 4.7907 |
| 1373957_at | Reln | reelin | 4.568 |
| 1375968_at | Ctnna2 | catenin (cadherin associated protein), alpha 2 | 4.4094 |
| 1367631_at | Ctgf | connective tissue growth factor | 4.374 |
| 1388196_at | Nckap1 | NCK-associated protein 1 | 4.268 |
| 1373102_at | Cdh13 | cadherin 13 | 4.2509 |
| 1369103_at | Fyn | FYN oncogene related to SRC, FGR, YES | 4.249 |
| 1371043_a_at | Pou3f3 | POU class 3 homeobox 3 | 4.1031 |
| 1388643_at | Fut8 | fucosyltransferase 8 (alpha (1,6) fucosyltransferase) | 3.9358 |
| 1368642_at | Cdh2 | cadherin 2 | 3.7671 |
| 1375719_s_at | Cdh13 | cadherin 13 | 3.5609 |
| 1398851_at | Ywhae | tyrosine 3-monooxygenase/tryptophan 5-monooxygenase activation protein, epsilon polypeptide | 3.2796 |
| 1387276_at | Dclk1 | doublecortin-like kinase 1 | 3.2522 |
| 1369964_at | Coro1a | coronin, actin binding protein 1A | 3.146 |
| 1388332_at | Rac1 | ras-related C3 botulinum toxin substrate 1 | 2.9728 |
| 1369559_a_at | Cd47 | Cd47 molecule | 2.843 |
| 1387259_at | Cdh2 | cadherin 2 | 2.8205 |
| 1388963_at | Astn1 | astrotactin 1 | 2.7225 |
| 1390650_at | Nup85 | nucleoporin 85kDa | 2.718 |
| 1369686_at | Dclk1 | doublecortin-like kinase 1 | 2.6822 |
| 1370607_a_at | Nrg1 | neuregulin 1 | 2.5663 |
| 1371947_at | Ndn | necdin homolog (mouse) | 2.5112 |
| 1373577_at | Nrp1 | neuropilin 1 | 2.4484 |
| 1369895_s_at | Podxl | podocalyxin-like | 2.3518 |
| 1387571_at | Nr2f1 | nuclear receptor subfamily 2, group F, member 1 | 2.2851 |
| 1368076_at | Vhl | von Hippel-Lindau tumor suppressor | 2.216 |

|  |  | **RMC specific genes involved in cell migration** |  |
| --- | --- | --- | --- |
| **Probe ID** | **Gene Symbol** | **Gene Name** | **Fold Change** |
| 1380370_at | Robo3 | roundabout homolog 3 (Drosophila) | 17.393 |
| 1368553_at | Acvrl1 | activin A receptor type II-like 1 | 2.4091 |
| 1371785_at | Tnfrsf12a | tumor necrosis factor receptor superfamily, member 12a | 2.3378 |
| 1374697_at | Plekhg5 | pleckstrin homology domain containing, family G (with RhoGef domain) member 5 | 2.3121 |
| 1387424_at | Cntn2 | contactin 2 (axonal) | 2.1679 |
| 1390479_at | Tlx3 | T-cell leukemia, homeobox 3 | 2.0799 |
| 1368186_a_at | Syk | spleen tyrosine kinase | 2.0797 |
| 1369284_at | Barhl2 | BarH-like homeobox 2 | 2.0607 |
| 1368335_at | Apoa1 | apolipoprotein A-I | 2.0495 |

|  |  | **AMC specific genes involved in cytoskeleton** |  |
| --- | --- | --- | --- |
| **Probe ID** | **Gene Symbol** | **Gene Name** | **Fold Change** |
| 1388101_at | Dpysl3 | dihydropyrimidinase-like 3 | 31.006 |
| 1375880_at | Appbp2 | amyloid beta precursor protein (cytoplasmic tail) binding protein 2 | 13.277 |
| 1387871_at | --- | cofilin 1, non-muscle; similar to Cofilin-1 (Cofilin, non-muscle isoform) | 9.3235 |
| 1370948_a_at | Marcks | myristoylated alanine rich protein kinase C substrate | 8.5949 |
| 1370290_at | Tubb5 | tubulin, beta 5 | 7.3307 |
| 1387892_at | Tubb5 | tubulin, beta 5 | 7.1734 |
| 1375525_at | Mapre1 | microtubule-associated protein, RP/EB family, member 1; similar to Microtubule-associated protein RP/EB family member 1 (APC-binding protein EB1) (End-binding protein 1) (EB1) | 7.0496 |
| 1383192_at | Spast | spastin | 6.988 |
| 1388131_at | Tubb2b | tubulin, beta 2b | 6.9175 |
| 1367579_a_at | Tuba1a /// Tuba1b /// Tuba1c | tubulin, alpha 1C | 6.3695 |
| 1387774_at | Ywhaz | tyrosine 3-monooxygenase/tryptophan 5-monooxygenase activation protein, zeta polypeptide | 6.0917 |
| 1371327_a_at | Actg1 | actin, gamma 1; similar to put. type 5 nonmuscle actin; similar to Actin, cytoplasmic 2 (Gamma-actin); similar to actin-like | 5.8562 |
| 1379450_at | Cttnbp2nl | CTTNBP2 N-terminal like | 5.6111 |
| 1370680_at | Stau2 | staufen, RNA binding protein, homolog 2 (Drosophila) | 5.6094 |
| 1387015_at | Pfn2 | profilin 2 | 5.1856 |
| 1384150_at | Mid1 | midline 1 | 5.0901 |
| 1398756_at | Npm1 | nucleophosmin (nucleolar phosphoprotein B23, numatrin); similar to Nucleophosmin (NPM) (Nucleolar phosphoprotein B23) (Numatrin) (Nucleolar protein NO38) | 4.9399 |
| 1386882_at | Dynlt1 | dynein light chain Tctex-type 1 | 4.8568 |
| 1386857_at | Stmn1 | stathmin 1 | 4.7384 |
| 1375424_at | Actr2 | ARP2 actin-related protein 2 homolog (yeast) | 4.6831 |
| 1368869_at | Akap12 | A kinase (PRKA) anchor protein 12 | 4.5495 |
| 1371653_at | Tpm4 | tropomyosin 4 | 4.5474 |
| 1370478_at | Myo16 | myosin XVI | 4.4391 |
| 1375968_at | Ctnna2 | catenin (cadherin associated protein), alpha 2 | 4.4094 |
| 1379754_at | Stau2 | staufen, RNA binding protein, homolog 2 (Drosophila) | 4.2722 |
| 1375538_at | Vcl | vinculin | 4.2035 |
| 1384068_at | Ckap2 | similar to cytoskeleton associated protein 2; cytoskeleton associated protein 2 | 4.1187 |
| 1383156_at | Kif2a | similar to hypothetical protein; kinesin family member 2A | 4.0729 |
| 1387142_at | Polb | polymerase (DNA directed), beta | 4.0578 |
| 1379261_at | Ska2 | hypothetical protein LOC691962; hypothetical protein LOC687897; family with sequence similarity 33, member A | 4.0094 |
| 1388566_at | Lasp1 | LIM and SH3 protein 1 | 3.953 |
| 1370890_at | Actr3 | ARP3 actin-related protein 3 homolog (yeast) | 3.8936 |
| 1371618_s_at | Tubb3 | tubulin, beta 3 | 3.8563 |
| 1368308_at | Myc | myelocytomatosis oncogene | 3.835 |
| 1370949_at | --- | myristoylated alanine rich protein kinase C substrate | 3.7211 |
| 1389163_at | Trim32 | tripartite motif-containing 32 | 3.6942 |
| 1398836_s_at | Actb | actin, beta | 3.6424 |
| 1373408_at | Tbca | tubulin folding cofactor A | 3.5647 |
| 1369041_at | Nlgn1 | neuroligin 1 | 3.5456 |
| 1372949_at | Sept11 | septin 11 | 3.5036 |
| 1398978_at | Ap1g1 | adaptor-related protein complex 1, gamma 1 subunit | 3.4678 |
| 1371490_at | Hsbp1 | heat shock factor binding protein 1 | 3.4454 |
| 1390022_at | Arpc5 | actin related protein 2/3 complex, subunit 5 | 3.4395 |
| 1371928_at | Cdca8 | cell division cycle associated 8 | 3.4068 |
| 1373363_at | Map1b | microtubule-associated protein 1B | 3.2594 |
| 1385157_at | Cbx1 | chromobox homolog 1 (HP1 beta homolog Drosophila ) | 3.2536 |
| 1370804_at | Gabarap | GABA(A) receptor-associated protein | 3.2524 |
| 1368240_a_at | Prkcb | protein kinase C, beta | 3.2509 |
| 1371511_at | Arpc2 | actin related protein 2/3 complex, subunit 2 | 3.2334 |
| 1389566_at | Ccnb2 | cyclin B2 | 3.2038 |
| 1378475_at | Appbp2 | amyloid beta precursor protein (cytoplasmic tail) binding protein 2 | 3.1876 |
| 1370059_at | Nefl | neurofilament, light polypeptide | 3.1827 |
| 1398757_at | Npm1 | nucleophosmin (nucleolar phosphoprotein B23, numatrin); similar to Nucleophosmin (NPM) (Nucleolar phosphoprotein B23) (Numatrin) (Nucleolar protein NO38) | 3.1793 |
| 1369964_at | Coro1a | coronin, actin binding protein 1A | 3.146 |
| 1367759_at | H1f0 | H1 histone family, member 0 | 3.1325 |
| 1370909_at | Nup62 | nucleoporin 62 | 3.128 |
| 1387025_at | Dync1i1 | dynein cytoplasmic 1 intermediate chain 1 | 3.124 |
| 1371733_at | Arl8a | ADP-ribosylation factor-like 8A | 3.1143 |
| 1370393_at | Haus1 | coiled-coil domain containing 5 | 3.0948 |
| 1373897_at | Lmnb1 | lamin B1 | 3.0938 |
| 1368571_at | Clip2 | CAP-GLY domain containing linker protein 2 | 3.0825 |
| 1370246_at | Calm2 | calmodulin pseudogene 2; calmodulin 3; calmodulin 2; calmodulin 1 | 3.0685 |
| 1399158_a_at | Npm1 | nucleophosmin (nucleolar phosphoprotein B23, numatrin); similar to Nucleophosmin (NPM) (Nucleolar phosphoprotein B23) (Numatrin) (Nucleolar protein NO38) | 3.0648 |
| 1368137_at | Mapt | microtubule-associated protein tau | 2.9792 |
| 1387856_at | Cnn3 | calponin 3, acidic | 2.8817 |
| 1399097_at | --- | similar to spindlin; spindlin 1 | 2.8794 |
| 1387772_at | Calm1 | calmodulin pseudogene 2; calmodulin 3; calmodulin 2; calmodulin 1 | 2.8775 |
| 1371360_at | Ndrg1 | N-myc downstream regulated gene 1 | 2.8387 |
| 1392590_at | Arhgap24 | Rho GTPase activating protein 24 | 2.8244 |
| 1371741_at | Actr1a | ARP1 actin-related protein 1 homolog A, centractin alpha (yeast) | 2.8046 |
| 1368508_at | LOC100361067 /// Psma3 /// Psma3l | proteasome (prosome, macropain) subunit, alpha type 3; proteasome subunit alpha type 3-like; similar to Proteasome subunit alpha type 3 (Proteasome component C8) (Macropain subunit C8) (Multicatalytic endopeptidase complex subunit C8) (Proteasome subunit K) | 2.7971 |
| 1370034_at | Cdc25b | cell division cycle 25 homolog B (S. pombe) | 2.7607 |
| 1395357_at | Map1b | microtubule-associated protein 1B | 2.7587 |
| 1368450_at | Myo5a | myosin Va | 2.7463 |
| 1384280_at | Nusap1 | nucleolar and spindle associated protein 1 | 2.7393 |
| 1373048_at | Actr10 | actin-related protein 10 homolog (S. cerevisiae) | 2.7331 |
| 1390650_at | Nup85 | nucleoporin 85kDa | 2.718 |
| 1374595_at | Tnks2 | tankyrase, TRF1-interacting ankyrin-related ADP-ribose polymerase 2 | 2.6977 |
| 1369637_at | Kif3c | kinesin family member 3C | 2.6881 |
| 1398803_at | Dync1h1 | dynein cytoplasmic 1 heavy chain 1 | 2.6624 |
| 1370339_at | Tpm3 | tropomyosin 3, gamma | 2.6579 |
| 1367655_at | Tmsb10 | similar to thymosin, beta 10; thymosin, beta 10 | 2.6407 |
| 1394079_at | Tbl1xr1 | transducin (beta)-like 1 X-linked receptor 1 | 2.6107 |
| 1371694_at | Dpysl2 | dihydropyrimidinase-like 2 | 2.6079 |
| 1383126_at | Akt1 | v-akt murine thymoma viral oncogene homolog 1 | 2.5848 |
| 1398831_at | Psmb4 | proteasome (prosome, macropain) subunit, beta type 4 | 2.5649 |
| 1389545_at | Tbl1xr1 | transducin (beta)-like 1 X-linked receptor 1 | 2.5429 |
| 1388874_at | Mtss1 | metastasis suppressor 1 | 2.5385 |
| 1371947_at | Ndn | necdin homolog (mouse) | 2.5112 |
| 1389005_at | Ip6k2 | inositol hexakisphosphate kinase 2 | 2.4902 |
| 1370450_at | Tpm3 | tropomyosin 3, gamma | 2.4784 |
| 1368603_at | Add2 | adducin 2 (beta) | 2.4619 |
| 1367605_at | Pfn1 | profilin 1 | 2.4598 |
| 1386967_at | Rhoq | ras homolog gene family, member Q | 2.458 |
| 1388128_at | Actr3 | ARP3 actin-related protein 3 homolog (yeast) | 2.4502 |
| 1376363_at | Actr10 | actin-related protein 10 homolog (S. cerevisiae) | 2.3891 |
| 1371533_at | Dctn6 | dynactin 6 | 2.3803 |
| 1372903_at | Kif18b /// LOC100363850 | kinesin family member 18B | 2.3528 |
| 1370116_at | Sept3 | septin 3 | 2.3462 |
| 1374428_at | Kif3b | kinesin family member 3B | 2.3164 |
| 1371148_s_at | Ina | internexin neuronal intermediate filament protein, alpha | 2.3027 |
| 1376951_at | Mad2l1 | MAD2 (mitotic arrest deficient, homolog)-like 1 (yeast) | 2.2948 |
| 1389520_at | Wdr1 | WD repeat domain 1 | 2.2916 |
| 1395429_at | Chrna7 | cholinergic receptor, nicotinic, alpha 7 | 2.2716 |
| 1398405_at | Sept6 | NFKB repressing factor; septin 6 | 2.2675 |
| 1398900_at | Dctn3 | similar to dynactin 3; dynactin 3 | 2.2652 |
| 1367766_at | Nme2 | non-metastatic cells 2, protein (NM23B) expressed in; non-metastatic cells 2, protein (NM23B) expressed in, pseudogene 1 | 2.2229 |
| 1371419_at | Sptbn1 | spectrin, beta, non-erythrocytic 1 | 2.1942 |
| 1378279_at | Gabarap | GABA(A) receptor-associated protein | 2.177 |
| 1370287_a_at | Tpm1 | tropomyosin 1, alpha | 2.1674 |
| 1388596_at | Cotl1 | coactosin-like 1 (Dictyostelium) | 2.1611 |
| 1382873_at | Cttnbp2nl | CTTNBP2 N-terminal like | 2.1313 |
| 1382143_at | Farp1 | FERM, RhoGEF (Arhgef) and pleckstrin domain protein 1 (chondrocyte-derived) | 2.1199 |
| 1371125_at | Kif2a | similar to hypothetical protein; kinesin family member 2A | 2.1086 |
| 1367698_a_at | Sept9 | septin 9 | 2.1059 |
| 1392532_at | Kif2a | similar to hypothetical protein; kinesin family member 2A | 2.0971 |
| 1367776_at | Cdc2 | cell division cycle 2, G1 to S and G2 to M | 2.0803 |
| 1380548_at | Ift57 | intraflagellar transport 57 homolog (Chlamydomonas) | 2.0491 |
| 1388386_at | Chmp1a | chromatin modifying protein 1A | 2.0489 |
| 1368138_at | Mapt | microtubule-associated protein tau | 2.034 |
| 1368157_at | Stmn3 | stathmin-like 3 | 2.0278 |
| 1389605_at | Jakmip1 | janus kinase and microtubule interacting protein 1 | 2.0262 |

|  |  | **RMC specific genes involved in cytoskeleton** |  |
| --- | --- | --- | --- |
| **Probe ID** | **Gene Symbol** | **Gene Name** | **Fold Change** |
| 1391330_at | Asb14 | dynein, axonemal, heavy polypeptide 12 | 12.772 |
| 1385547_at | Ermn | ermin, ERM-like protein | 8.8241 |
| 1372195_at | Tnnc2 | troponin C type 2 (fast) | 8.6024 |
| 1389448_at | Sept4 | septin 4 | 6.0436 |
| 1368306_at | Grin2c | glutamate receptor, ionotropic, N-methyl D-aspartate 2C | 5.9695 |
| 1389770_at | Ttll9 | tubulin tyrosine ligase-like family, member 9 | 5.7674 |
| 1370896_a_at | Myh11 | myosin, heavy chain 11, smooth muscle | 5.2547 |
| 1376564_at | Sptbn4 | spectrin, beta, non-erythrocytic 4 | 5.1284 |
| 1370668_a_at | Cnksr2 | connector enhancer of kinase suppressor of Ras 2 | 5.1124 |
| 1376124_at | Iqub | IQ motif and ubiquitin domain containing; Wiskott-Aldrich syndrome-like | 5.0687 |
| 1392996_at | Cpeb1 | cytoplasmic polyadenylation element binding protein 1 | 4.8865 |
| 1370412_at | Tnnt1 | troponin T type 1 (skeletal, slow) | 4.4062 |
| 1370815_at | Nefh | neurofilament, heavy polypeptide | 4.2066 |
| 1394609_at | Ablim2 | actin binding LIM protein family, member 2 | 4.1983 |
| 1386931_at | Tnni3 | troponin I type 3 (cardiac) | 4.0048 |
| 1383943_at | Dnah7 | dynein, axonemal, heavy polypeptide 7 | 3.9824 |
| 1371414_at | Gsn | gelsolin | 3.9717 |
| 1381039_at | Dnah1 | dynein, axonemal, heavy chain 1 | 3.858 |
| 1368740_at | P2rx6 | purinergic receptor P2X, ligand-gated ion channel, 6 | 3.8483 |
| 1386296_at | --- | septin 8 | 3.8265 |
| 1389876_at | Camk2n1 | calcium/calmodulin-dependent protein kinase II inhibitor 1 | 3.7875 |
| 1380405_at | Sept8 | septin 8 | 3.7375 |
| 1387068_at | Arc | activity-regulated cytoskeleton-associated protein | 3.3203 |
| 1387337_at | Cort | cortistatin; kinesin family member 1B | 3.2903 |
| 1388718_at | Tmod1 | tropomodulin 1 | 3.2862 |
| 1388740_at | Fermt3 | fermitin family homolog 3 (Drosophila) | 3.0629 |
| 1389157_at | Cdc42ep1 | CDC42 effector protein (Rho GTPase binding) 1 | 3.0373 |
| 1392279_at | Dnai1 | dynein, axonemal, intermediate chain 1 | 3.0337 |
| 1374735_at | Arhgap4 | Rho GTPase activating protein 4 | 2.9998 |
| 1391625_at | Wasl | IQ motif and ubiquitin domain containing; Wiskott-Aldrich syndrome-like | 2.9348 |
| 1383138_at | Ssh3 | slingshot homolog 3 (Drosophila) | 2.9245 |
| 1374062_x_at | Mapre3 | microtubule-associated protein, RP/EB family, member 3 | 2.894 |
| 1377384_at | Plekhh3 | pleckstrin homology domain containing, family H (with MyTH4 domain) member 3 | 2.8935 |
| 1380989_at | Numa1 | nuclear mitotic apparatus protein 1 | 2.7358 |
| 1378384_at | Ints6 | integrator complex subunit 6 | 2.6003 |
| 1387907_at | Itpr1 | inositol 1,4,5-triphosphate receptor, type 1 | 2.5019 |
| 1384182_at | Fermt2 | fermitin family homolog 2 (Drosophila) | 2.3256 |
| 1386699_at | Dnali1 | dynein, axonemal, light intermediate chain 1 | 2.3126 |
| 1388539_at | Pkp2 | plakophilin 2 | 2.3106 |
| 1374374_x_at | Shroom1 | shroom family member 1 | 2.2842 |
| 1383426_at | Pstpip1 | proline-serine-threonine phosphatase-interacting protein 1 | 2.2798 |
| 1395447_at | Ermn | ermin, ERM-like protein | 2.2732 |
| 1389002_at | Tln1 | talin 1 | 2.2627 |
| 1368054_at | Lmna | lamin A | 2.2565 |
| 1371075_at | Myh13 | myosin, heavy chain 13, skeletal muscle | 2.2529 |
| 1379025_at | Ubr4 | ZUBR1 | 2.2474 |
| 1371664_at | Pxn | paxillin | 2.2373 |
| 1380492_at | Freq | frequenin homolog (Drosophila) | 2.2311 |
| 1374995_at | Elmo3 | engulfment and cell motility 3 | 2.2129 |
| 1377798_at | Tchp | trichoplein, keratin filament binding | 2.1749 |
| 1384095_at | Myrip | myosin VIIA and Rab interacting protein | 2.1568 |
| 1375833_at | Ptpn4 | protein tyrosine phosphatase, non-receptor type 4 | 2.086 |
| 1368081_at | Abca2 | ATP-binding cassette, sub-family A (ABC1), member 2 | 2.0815 |

|  |  | **AMC specific genes involved in cell morphogenesis** |  |
| --- | --- | --- | --- |
| **Probe ID** | **Gene Symbol** | **Gene Name** | **Fold Change** |
| 1388101_at | Dpysl3 | dihydropyrimidinase-like 3 | 31.006 |
| 1374966_at | Dcx | doublecortin | 16.642 |
| 1373661_a_at | Cxcr4 | chemokine (C-X-C motif) receptor 4 | 12.759 |
| 1383747_at | Ect2 | epithelial cell transforming sequence 2 oncogene | 12.498 |
| 1389244_x_at | Cxcr4 | chemokine (C-X-C motif) receptor 4 | 12.274 |
| 1387871_at | --- | cofilin 1, non-muscle; similar to Cofilin-1 (Cofilin, non-muscle isoform) | 9.3235 |
| 1370309_a_at | Hnrnpab | heterogeneous nuclear ribonucleoprotein A/B | 8.8712 |
| 1371412_a_at | Nrep | neuronal regeneration related protein | 7.4128 |
| 1375896_at | Stradb | amyotrophic lateral sclerosis 2 (juvenile) chromosome region, candidate 2 (human) | 5.8947 |
| 1372513_at | Rac1 | ras-related C3 botulinum toxin substrate 1 | 5.5863 |
| 1384497_at | Klf7 | Kruppel-like factor 7 (ubiquitous) | 5.4363 |
| 1391127_at | Cdc42 | cell division cycle 42 (GTP binding protein) | 5.1257 |
| 1369242_at | Pax6 | paired box 6 | 5.123 |
| 1371232_a_at | Vcan | versican | 4.9595 |
| 1386857_at | Stmn1 | stathmin 1 | 4.7384 |
| 1398270_at | Bmp2 | bone morphogenetic protein 2 | 4.5698 |
| 1373957_at | Reln | reelin | 4.568 |
| 1375968_at | Ctnna2 | catenin (cadherin associated protein), alpha 2 | 4.4094 |
| 1367654_at | Fat1 | FAT tumor suppressor homolog 1 (Drosophila) | 4.2863 |
| 1368042_a_at | Hmg1l1 | similar to high mobility group protein 1 (HMG-1) | 3.9596 |
| 1367930_at | Gap43 | growth associated protein 43 | 3.9493 |
| 1398836_s_at | Actb | actin, beta | 3.6424 |
| 1373363_at | Map1b | microtubule-associated protein 1B | 3.2594 |
| 1387276_at | Dclk1 | doublecortin-like kinase 1 | 3.2522 |
| 1370059_at | Nefl | neurofilament, light polypeptide | 3.1827 |
| 1382630_at | RGD1311558 | similar to 4930506M07Rik protein | 3.0291 |
| 1388332_at | Rac1 | ras-related C3 botulinum toxin substrate 1 | 2.9728 |
| 1375729_at | Epha4 | Eph receptor A4 | 2.7895 |
| 1388054_a_at | Vcan | versican | 2.7777 |
| 1395357_at | Map1b | microtubule-associated protein 1B | 2.7587 |
| 1376139_at | Plxna3 | plexin A3 | 2.7048 |
| 1369686_at | Dclk1 | doublecortin-like kinase 1 | 2.6822 |
| 1380168_at | Etv4 | ets variant 4 | 2.6701 |
| 1368945_at | Bmp2 | bone morphogenetic protein 2 | 2.6681 |
| 1382632_at | Robo2 | roundabout, axon guidance receptor, homolog 2 (Drosophila) | 2.6021 |
| 1369213_at | L1cam | L1 cell adhesion molecule | 2.5785 |
| 1370607_a_at | Nrg1 | neuregulin 1 | 2.5663 |
| 1371947_at | Ndn | necdin homolog (mouse) | 2.5112 |
| 1373577_at | Nrp1 | neuropilin 1 | 2.4484 |
| 1379750_at | Robo2 | roundabout, axon guidance receptor, homolog 2 (Drosophila) | 2.3881 |
| 1386913_at | Pdpn | podoplanin | 2.3404 |
| 1388783_at | Hmgb1 | similar to high mobility group protein; Rattus norvegicus high mobility group box 1, pseudogene 3; similar to Hmgb1 protein; similar to High mobility group protein 1 (HMG-1) | 2.1736 |
| 1372155_at | Trim28 | tripartite motif-containing 28 | 2.1465 |
| 1370166_at | Sdc2 | syndecan 2 | 2.0821 |

|  |  | **RMC specific genes involved in cell morphogenesis** | |
| --- | --- | --- | --- |
| **Probe ID** | **Gene Symbol** | **Gene Name** | **Fold Change** |
| 1380370_at | Robo3 | roundabout homolog 3 (Drosophila) | 17.393 |
| 1376564_at | Sptbn4 | spectrin, beta, non-erythrocytic 4 | 5.1284 |
| 1392916_at | Map7 | microtubule-associated protein 7 | 3.263 |
| 1374374_x_at | Shroom1 | shroom family member 1 | 2.2842 |
| 1387424_at | Cntn2 | contactin 2 (axonal) | 2.1679 |
| 1369101_at | Rxra | retinoid X receptor alpha | 2.1608 |
| 1387479_at | Runx3 | runt-related transcription factor 3 | 2.1139 |
| 1369582_at | Vax2 | ventral anterior homeobox 2 | 2.0916 |
| 1368335_at | Apoa1 | apolipoprotein A-I | 2.0495 |
| 1387760_a_at | Onecut1 | one cut homeobox 1 | 2.002 |

|  |  | **AMC specific genes involved in Neuron differentiation** |  |
| --- | --- | --- | --- |
| **Probe ID** | **Gene Symbol** | **Gene Name** | **Fold Change** |
| 1388101_at | Dpysl3 | dihydropyrimidinase-like 3 | 31.006 |
| 1374966_at | Dcx | doublecortin | 16.642 |
| 1387899_at | Crmp1 | collapsin response mediator protein 1 | 15.498 |
| 1373661_a_at | Cxcr4 | chemokine (C-X-C motif) receptor 4 | 12.759 |
| 1389244_x_at | Cxcr4 | chemokine (C-X-C motif) receptor 4 | 12.274 |
| 1384437_at | Smarca1 | SWI/SNF related, matrix associated, actin dependent regulator of chromatin, subfamily a, member 1 | 10.724 |
| 1369953_a_at | Cd24 | CD24 molecule | 9.2544 |
| 1387141_at | Dpysl5 | dihydropyrimidinase-like 5 | 8.7932 |
| 1371412_a_at | Nrep | neuronal regeneration related protein | 7.4128 |
| 1368879_a_at | Gnao1 | guanine nucleotide binding protein (G protein), alpha activating activity polypeptide O | 7.3973 |
| 1370448_at | Gpc2 | glypican 2 | 6.8796 |
| 1372513_at | Rac1 | ras-related C3 botulinum toxin substrate 1 | 5.5863 |
| 1384497_at | Klf7 | Kruppel-like factor 7 (ubiquitous) | 5.4363 |
| 1390386_at | Casp3 | caspase 3, apoptosis related cysteine protease | 5.3212 |
| 1391127_at | Cdc42 | cell division cycle 42 (GTP binding protein) | 5.1257 |
| 1369242_at | Pax6 | paired box 6 | 5.123 |
| 1371232_a_at | Vcan | versican | 4.9595 |
| 1386857_at | Stmn1 | stathmin 1 | 4.7384 |
| 1373957_at | Reln | reelin | 4.568 |
| 1393708_at | Bhlhe22 | basic helix-loop-helix family, member e22 | 4.5657 |
| 1375968_at | Ctnna2 | catenin (cadherin associated protein), alpha 2 | 4.4094 |
| 1375050_at | Gprin1 | G protein-regulated inducer of neurite outgrowth 1 | 4.1506 |
| 1367930_at | Gap43 | growth associated protein 43 | 3.9493 |
| 1371618_s_at | Tubb3 | tubulin, beta 3 | 3.8563 |
| 1398874_at | Atxn10 | ataxin 10 | 3.8086 |
| 1385387_at | Nkx2-2 | NK2 homeobox 2 | 3.7815 |
| 1375119_at | Nedd4 | neural precursor cell expressed, developmentally down-regulated gene 4 | 3.6588 |
| 1398836_s_at | Actb | actin, beta | 3.6424 |
| 1369041_at | Nlgn1 | neuroligin 1 | 3.5456 |
| 1373363_at | Map1b | microtubule-associated protein 1B | 3.2594 |
| 1387276_at | Dclk1 | doublecortin-like kinase 1 | 3.2522 |
| 1370059_at | Nefl | neurofilament, light polypeptide | 3.1827 |
| 1389791_at | Cln8 | ceroid-lipofuscinosis, neuronal 8 | 3.1431 |
| 1370908_at | Hdac2 | similar to Histone deacetylase 2 (HD2); histone deacetylase 2 | 3.0713 |
| 1382630_at | RGD1311558 | similar to 4930506M07Rik protein | 3.0291 |
| 1368137_at | Mapt | microtubule-associated protein tau | 2.9792 |
| 1388332_at | Rac1 | ras-related C3 botulinum toxin substrate 1 | 2.9728 |
| 1381533_at | Rnd1 | Rho family GTPase 1 | 2.9597 |
| 1375729_at | Epha4 | Eph receptor A4 | 2.7895 |
| 1388054_a_at | Vcan | versican | 2.7777 |
| 1395357_at | Map1b | microtubule-associated protein 1B | 2.7587 |
| 1376139_at | Plxna3 | plexin A3 | 2.7048 |
| 1369686_at | Dclk1 | doublecortin-like kinase 1 | 2.6822 |
| 1380168_at | Etv4 | ets variant 4 | 2.6701 |
| 1382632_at | Robo2 | roundabout, axon guidance receptor, homolog 2 (Drosophila) | 2.6021 |
| 1369213_at | L1cam | L1 cell adhesion molecule | 2.5785 |
| 1370607_a_at | Nrg1 | neuregulin 1 | 2.5663 |
| 1371947_at | Ndn | necdin homolog (mouse) | 2.5112 |
| 1373577_at | Nrp1 | neuropilin 1 | 2.4484 |
| 1373378_at | Agtpbp1 | ATP/GTP binding protein 1 | 2.401 |
| 1379750_at | Robo2 | roundabout, axon guidance receptor, homolog 2 (Drosophila) | 2.3881 |
| 1387769_a_at | Id3 | inhibitor of DNA binding 3 | 2.3788 |
| 1390447_at | Stx3 | syntaxin 3 | 2.2896 |
| 1383071_at | Mtpn | myotrophin | 2.2567 |
| 1370166_at | Sdc2 | syndecan 2 | 2.0821 |
| 1384227_at | Ptprk | protein tyrosine phosphatase, receptor type, K, extracellular region | 2.0808 |
| 1373907_at | Trappc4 | trafficking protein particle complex 4 | 2.0419 |
| 1368138_at | Mapt | microtubule-associated protein tau | 2.034 |
| 1368157_at | Stmn3 | stathmin-like 3 | 2.0278 |

|  |  | **RMC specific genes involved in Neuron differentiation** |  |
| --- | --- | --- | --- |
| **Probe ID** | **Gene Symbol** | **Gene Name** | **Fold Change** |
| 1380370_at | Robo3 | roundabout homolog 3 (Drosophila) | 17.393 |
| 1381374_at | Lgi4 | leucine-rich repeat LGI family, member 4 | 8.1016 |
| 1376564_at | Sptbn4 | spectrin, beta, non-erythrocytic 4 | 5.1284 |
| 1375032_at | --- | kinase non-catalytic C-lobe domain (KIND) containing 1 | 4.2146 |
| 1378803_at | Nkx6-2 | NK6 homeobox 2 | 3.0395 |
| 1390936_at | Lgi4 | leucine-rich repeat LGI family, member 4 | 2.9917 |
| 1375615_at | Ntrk1 | neurotrophic tyrosine kinase, receptor, type 1 | 2.3371 |
| 1393968_at | Scarf1 | scavenger receptor class F, member 1 | 2.2997 |
| 1387424_at | Cntn2 | contactin 2 (axonal) | 2.1679 |
| 1369101_at | Rxra | retinoid X receptor alpha | 2.1608 |
| 1387479_at | Runx3 | runt-related transcription factor 3 | 2.1139 |
| 1369582_at | Vax2 | ventral anterior homeobox 2 | 2.0916 |
| 1390479_at | Tlx3 | T-cell leukemia, homeobox 3 | 2.0799 |
| 1386995_at | Btg2 | B-cell translocation gene 2, anti-proliferative | 2.0761 |
| 1369284_at | Barhl2 | BarH-like homeobox 2 | 2.0607 |
| 1396820_at | Hdac1 | hypothetical gene supported by AF321129; similar to histone deacetylase 1; histone deacetylase 1; histone deacetylase 1-like | 2.0538 |
| 1368335_at | Apoa1 | apolipoprotein A-I | 2.0495 |
| 1398212_at | Olig2 | oligodendrocyte lineage transcription factor 2 | 2.046 |
| 1369329_at | Notch3 | Notch homolog 3 (Drosophila) | 2.0324 |
| 1381801_at | Fscn2 | fascin homolog 2, actin-bundling protein, retinal (Strongylocentrotus purpuratus) | 2.0267 |

|  |  | **AMC specific genes involved in Glial differentiation** |  |
| --- | --- | --- | --- |
| **Probe ID** | **Gene Symbol** | **Gene Name** | **Fold Change** |
| 1369242_at | Pax6 | paired box 6 | 5.123 |
| 1373957_at | Reln | reelin | 4.568 |
| 1370432_at | Pou3f1 | POU class 3 homeobox 1 | 4.5113 |
| 1387275_at | Sox11 | SRY (sex determining region Y)-box 11 | 4.5022 |
| 1367930_at | Gap43 | growth associated protein 43 | 3.9493 |
| 1385387_at | Nkx2-2 | NK2 homeobox 2 | 3.7815 |
| 1370607_a_at | Nrg1 | neuregulin 1 | 2.5663 |

|  |  | **RMC specific genes involved in Glial differentiation** |  |
| --- | --- | --- | --- |
| **Probe ID** | **Gene Symbol** | **Gene Name** | **Fold Change** |
| 1387112_at | Plp1 | proteolipid protein 1 | 8.9996 |
| 1387811_at | Agt | angiotensinogen (serpin peptidase inhibitor, clade A, member 8) | 6.3234 |
| 1368563_at | Aspa | aspartoacylase | 4.4382 |
| 1371414_at | Gsn | gelsolin | 3.9717 |
| 1374954_at | Hdac11 | histone deacetylase 11 | 3.1063 |
| 1378803_at | Nkx6-2 | NK6 homeobox 2 | 3.0395 |
| 1398212_at | Olig2 | oligodendrocyte lineage transcription factor 2 | 2.046 |
| 1376263_at | Metrn | meteorin, glial cell differentiation regulator | 2.0417 |

|  |  | **AMC specific genes involved in Myeloid differentiation** |  |
| --- | --- | --- | --- |
| **Probe ID** | **Gene Symbol** | **Gene Name** | **Fold Change** |
| 1371887_at | Hmgb3 | high mobility group box 3; similar to High mobility group protein 4 (HMG-4) (High mobility group protein 2a) (HMG-2a) | 12.466 |
| 1368042_a_at | Hmg1l1 | similar to high mobility group protein | 3.9596 |
| 1368870_at | Id2 | inhibitor of DNA binding 2 | 3.524 |
| 1389528_s_at | Jun | Jun oncogene | 3.0095 |
| 1369897_s_at | Gnas | GNAS complex locus | 2.473 |
| 1367766_at | Nme2 | non-metastatic cells 2, protein (NM23B) expressed in; non-metastatic cells 2, protein (NM23B) expressed in, pseudogene 1 | 2.2229 |
| 1388783_at | Hmgb1 | similar to high mobility group protein | 2.1736 |

|  |  | **RMC specific genes involved in Myeloid differentiation** |  |
| --- | --- | --- | --- |
| **Probe ID** | **Gene Symbol** | **Gene Name** | **Fold Change** |
| 1384202_at | Tesc | tescalcin | 5.6757 |
| 1367733_at | Car2 | carbonic anhydrase II | 3.7983 |
| 1393836_at | Mitf | microphthalmia-associated transcription factor | 2.9697 |
| 1380582_at | Csf1 | colony stimulating factor 1 (macrophage) | 2.8628 |
| 1387107_at | Zbtb7a | zinc finger and BTB domain containing 7a | 2.1625 |
| 1381997_at | Adipoq | adiponectin, C1Q and collagen domain containing | 2.1101 |
| 1374741_at | Esrra | estrogen related receptor, alpha | 2.1 |
| 1386922_at | Car2 | carbonic anhydrase II | 2.0443 |

|  |  | **AMC specific genes involved in Leukocyte differentiation** |  |
| --- | --- | --- | --- |
| **Probe ID** | **Gene Symbol** | **Gene Name** | **Fold Change** |
| 1384000_at | Sox4 | SRY (sex determining region Y)-box 4 | 28.328 |
| 1369953_a_at | Cd24 | CD24 molecule | 9.2544 |
| 1383137_at | Sox4 | SRY (sex determining region Y)-box 4 | 6.6886 |
| 1369770_at | Sstr1 | somatostatin receptor 1 | 6.1777 |
| 1399033_at | Cbfb | core-binding factor, beta subunit | 6.0758 |
| 1373860_at | Sox4 | SRY (sex determining region Y)-box 4 | 4.6399 |
| 1383506_at | Bcl11a | B-cell CLL/lymphoma 11A (zinc finger protein) | 3.5046 |
| 1369964_at | Coro1a | coronin, actin binding protein 1A | 3.146 |
| 1368002_at | Msh2 | mutS homolog 2 (E. coli) | 3.1126 |
| 1374304_at | Xrcc4 | X-ray repair complementing defective repair in Chinese hamster cells 4 | 3.0992 |
| 1367831_at | Tp53 | tumor protein p53 | 2.1165 |
| 1379651_at | Foxp1 | forkhead box P1 | 2.0145 |

|  |  | **RMC specific genes involved in Leukocyte differentiation** |  |
| --- | --- | --- | --- |
| **Probe ID** | **Gene Symbol** | **Gene Name** | **Fold Change** |
| 1367733_at | Car2 | carbonic anhydrase II | 3.7983 |
| 1372536_at | Cabc1 | presenilin 2; chaperone, ABC1 activity of bc1 complex homolog (S. pombe) | 3.2619 |
| 1368321_at | Egr1 | early growth response 1 | 3.2473 |
| 1393836_at | Mitf | microphthalmia-associated transcription factor | 2.9697 |
| 1380582_at | Csf1 | colony stimulating factor 1 (macrophage) | 2.8628 |
| 1372740_at | LOC687705 /// Mink1 | similar to misshapen-like kinase 1 isoform 1; similar to Map4k6-pending protein | 2.7585 |
| 1375615_at | Ntrk1 | neurotrophic tyrosine kinase, receptor, type 1 | 2.3371 |
| 1367679_at | Cd74 | Cd74 molecule, major histocompatibility complex, class II invariant chain | 2.1378 |
| 1368186_a_at | Syk | spleen tyrosine kinase | 2.0797 |
| 1386922_at | Car2 | carbonic anhydrase II | 2.0443 |
| 1387760_a_at | Onecut1 | one cut homeobox 1 | 2.002 |

|  |  | **AMC specific genes involved in Lymphocyte differentiation** |  |
| --- | --- | --- | --- |
| **Probe ID** | **Gene Symbol** | **Gene Name** | **Fold Change** |
| 1384000_at | Sox4 | SRY (sex determining region Y)-box 4 | 28.328 |
| 1369953_a_at | Cd24 | CD24 molecule | 9.2544 |
| 1383137_at | Sox4 | SRY (sex determining region Y)-box 4 | 6.6886 |
| 1369770_at | Sstr1 | somatostatin receptor 1 | 6.1777 |
| 1399033_at | Cbfb | core-binding factor, beta subunit | 6.0758 |
| 1373860_at | Sox4 | SRY (sex determining region Y)-box 4 | 4.6399 |
| 1383506_at | Bcl11a | B-cell CLL/lymphoma 11A (zinc finger protein) | 3.5046 |
| 1369964_at | Coro1a | coronin, actin binding protein 1A | 3.146 |
| 1368002_at | Msh2 | mutS homolog 2 (E. coli) | 3.1126 |
| 1374304_at | Xrcc4 | X-ray repair complementing defective repair in Chinese hamster cells 4 | 3.0992 |
| 1367831_at | Tp53 | tumor protein p53 | 2.1165 |
| 1379651_at | Foxp1 | forkhead box P1 | 2.0145 |

|  |  | **RMC specific genes involved in Lymphocyte differentiation** |  |
| --- | --- | --- | --- |
| **Probe ID** | **Gene Symbol** | **Gene Name** | **Fold Change** |
| 1368321_at | Egr1 | early growth response 1 | 3.2473 |
| 1372740_at | LOC687705 /// Mink1 | similar to misshapen-like kinase 1 isoform 1; similar to Map4k6-pending protein | 2.7585 |
| 1375615_at | Ntrk1 | neurotrophic tyrosine kinase, receptor, type 1 | 2.3371 |
| 1367679_at | Cd74 | Cd74 molecule, major histocompatibility complex, class II invariant chain | 2.1378 |
| 1368186_a_at | Syk | spleen tyrosine kinase | 2.0797 |
| 1387760_a_at | Onecut1 | one cut homeobox 1 | 2.002 |

|  |  | **AMC specific genes involved in Muscle cell differentiation** |  |
| --- | --- | --- | --- |
| **Probe ID** | **Gene Symbol** | **Gene Name** | **Fold Change** |
| 1371327_a_at | Actg1 | actin, gamma 1; similar to put. type 5 nonmuscle actin; similar to Actin, cytoplasmic 2 (Gamma-actin); similar to actin-like | 5.8562 |
| 1392770_at | Neo1 | neogenin homolog 1 (chicken) | 3.0202 |
| 1370282_at | Csrp2 | cysteine and glycine-rich protein 2 | 2.641 |
| 1370607_a_at | Nrg1 | neuregulin 1 | 2.5663 |
| 1383071_at | Mtpn | myotrophin | 2.2567 |
| 1370307_at | Agrn | agrin | 2.1852 |
| 1388686_at | Rcan1 | regulator of calcineurin 1 | 2.0934 |
| 1379651_at | Foxp1 | forkhead box P1 | 2.0145 |

|  |  | **RMC specific genes involved in Muscle cell differentiation** |  |
| --- | --- | --- | --- |
| **Probe ID** | **Gene Symbol** | **Gene Name** | **Fold Change** |
| 1387811_at | Agt | angiotensinogen (serpin peptidase inhibitor, clade A, member 8) | 6.3234 |
| 1370896_a_at | Myh11 | myosin, heavy chain 11, smooth muscle | 5.2547 |
| 1388718_at | Tmod1 | tropomodulin 1 | 3.2862 |
| 1398297_at | Mapk12 | mitogen-activated protein kinase 12 | 2.9091 |
| 1367779_at | Bin1 | bridging integrator 1 | 2.4971 |
| 1375613_at | Gata6 | GATA binding protein 6 | 2.1637 |
| 1369101_at | Rxra | retinoid X receptor alpha | 2.1608 |
| 1369607_at | Fgf6 | fibroblast growth factor 6 | 2.1594 |
| 1391557_at | Sox15 | SRY (sex determining region Y)-box 15 | 2.1253 |
| 1370264_at | Syne1 | spectrin repeat containing, nuclear envelope 1 | 2.0433 |

|  |  | **AMC specific genes involved in Leydig cell differentiation** |  |
| --- | --- | --- | --- |
| **Probe ID** | **Gene Symbol** | **Gene Name** | **Fold Change** |
| 1383075_at | Ccnd1 | cyclin D1 | 3.201 |
| 1367662_at | Hsd17b10 | hydroxysteroid (17-beta) dehydrogenase 10 | 2.144 |

|  |  | **RMC specific genes involved in Leydig cell differentiation** |  |
| --- | --- | --- | --- |
| **Probe ID** | **Gene Symbol** | **Gene Name** | **Fold Change** |
| 1392916_at | Map7 | microtubule-associated protein 7 | 3.263 |
| 1367612_at | Mgst1 | microsomal glutathione S-transferase 1 | 2.1862 |

|  |  | **AMC specific genes involved in Cell Adhesion** |  |
| --- | --- | --- | --- |
| **Probe ID** | **Gene Symbol** | **Gene Name** | **Fold Change** |
| 1369953_a_at | Cd24 | CD24 molecule | 9.2544 |
| 1384824_at | Pcdh18 | protocadherin 18 | 6.9746 |
| 1374643_at | Fat4 | FAT tumor suppressor homolog 4 (Drosophila); similar to FAT tumor suppressor homolog 4 | 6.4558 |
| 1372513_at | Rac1 | ras-related C3 botulinum toxin substrate 1 | 5.5863 |
| 1393149_at | Pcdha1 | protocadherin alpha 4 | 5.4411 |
| 1375337_at | Adam9 | ADAM metallopeptidase domain 9 (meltrin gamma) | 5.0154 |
| 1371232_a_at | Vcan | versican | 4.9595 |
| 1387126_at | Atp2c1 | ATPase, Ca++ transporting, type 2C, member 1 | 4.6029 |
| 1373957_at | Reln | reelin | 4.568 |
| 1382192_at | Lyve1 | lymphatic vessel endothelial hyaluronan receptor 1 | 4.5102 |
| 1375968_at | Ctnna2 | catenin (cadherin associated protein), alpha 2 | 4.4094 |
| 1367631_at | Ctgf | connective tissue growth factor | 4.374 |
| 1370959_at | Col3a1 | collagen, type III, alpha 1 | 4.3557 |
| 1367888_at | Pcdh21 | protocadherin 21 | 4.3513 |
| 1367654_at | Fat1 | FAT tumor suppressor homolog 1 (Drosophila) | 4.2863 |
| 1373102_at | Cdh13 | cadherin 13 | 4.2509 |
| 1375538_at | Vcl | vinculin | 4.2035 |
| 1370016_at | Nell2 | NEL-like 2 (chicken); similar to protein kinase C-binding protein NELL2 | 4.1415 |
| 1387204_at | Negr1 | neuronal growth regulator 1 | 3.9103 |
| 1368642_at | Cdh2 | cadherin 2 | 3.7671 |
| 1375719_s_at | Cdh13 | cadherin 13 | 3.5609 |
| 1369041_at | Nlgn1 | neuroligin 1 | 3.5456 |
| 1376105_at | Col14a1 | collagen, type XIV, alpha 1 | 3.2817 |
| 1384509_s_at | Pcdh17 | protocadherin 17 | 3.1805 |
| 1369964_at | Coro1a | coronin, actin binding protein 1A | 3.146 |
| 1392770_at | Neo1 | neogenin homolog 1 (chicken) | 3.0202 |
| 1388332_at | Rac1 | ras-related C3 botulinum toxin substrate 1 | 2.9728 |
| 1394316_a_at | Tspan5 | tetraspanin 5 | 2.9458 |
| 1371518_at | Nid1 | nidogen 1 | 2.8619 |
| 1369559_a_at | Cd47 | Cd47 molecule | 2.843 |
| 1387259_at | Cdh2 | cadherin 2 | 2.8205 |
| 1388054_a_at | Vcan | versican | 2.7777 |
| 1381206_at | Plcxd2 | phosphatidylinositol-specific phospholipase C, X domain containing 2; CD96 molecule | 2.777 |
| 1388963_at | Astn1 | astrotactin 1 | 2.7225 |
| 1393173_at | Rasa1 | RAS p21 protein activator (GTPase activating protein) 1 | 2.6875 |
| 1377089_a_at | Tspan5 | tetraspanin 5 | 2.6418 |
| 1382632_at | Robo2 | roundabout, axon guidance receptor, homolog 2 (Drosophila) | 2.6021 |
| 1369213_at | L1cam | L1 cell adhesion molecule | 2.5785 |
| 1383575_at | Ctnnd2 | catenin (cadherin-associated protein), delta 2 (neural plakophilin-related arm-repeat protein) | 2.5246 |
| 1372180_at | Sdc3 | syndecan 3 | 2.4525 |
| 1373577_at | Nrp1 | neuropilin 1 | 2.4484 |
| 1367569_at | Rpsa | similar to 40S ribosomal protein SA (p40) (34/67 kDa laminin receptor); ribosomal protein SA | 2.3925 |
| 1379750_at | Robo2 | roundabout, axon guidance receptor, homolog 2 (Drosophila) | 2.3881 |
| 1386913_at | Pdpn | podoplanin | 2.3404 |
| 1367582_at | Rpl29 | ribosomal protein L29; similar to 60S ribosomal protein L29 (P23); ribosomal protein L29, pseudogene 1; similar to 60S ribosomal protein L29 | 2.2795 |
| 1396100_at | Aggf1 | angiogenic factor with G patch and FHA domains 1 | 2.2678 |
| 1371696_at | Gpr56 | G protein-coupled receptor 56 | 2.2564 |
| 1391146_at | Cdh11 | cadherin 11 | 2.2357 |
| 1373824_at | Cfdp1 | similar to craniofacial development protein 1; craniofacial development protein 1 | 2.194 |
| 1384227_at | Ptprk | protein tyrosine phosphatase, receptor type, K, extracellular region | 2.0808 |
| 1374589_at | Vezt | vezatin, adherens junctions transmembrane protein | 2.0539 |

|  |  | **RMC specific genes involved in Cell Adhesion** |  |
| --- | --- | --- | --- |
| **Probe ID** | **Gene Symbol** | **Gene Name** | **Fold Change** |
| 1370849_at | Hapln2 | hyaluronan and proteoglycan link protein 2 | 9.8354 |
| 1368861_a_at | Mag | myelin-associated glycoprotein | 8.1186 |
| 1387811_at | Agt | angiotensinogen (serpin peptidase inhibitor, clade A, member 8) | 6.3234 |
| 1389891_at | Col11a2 | collagen, type XI, alpha 2 | 5.6344 |
| 1376711_at | Cldn11 | claudin 11 | 5.1628 |
| 1375708_at | Col27a1 | collagen, type XXVII, alpha 1 | 4.2993 |
| 1370185_at | Cntnap1 | contactin associated protein 1 | 3.3452 |
| 1392754_at | Adam8 | ADAM metallopeptidase domain 8 | 3.3285 |
| 1391575_at | Hapln4 | hyaluronan and proteoglycan link protein 4 | 3.0551 |
| 1388145_at | Tnxb | tenascin XA | 2.4498 |
| 1368115_at | Cldn3 | claudin 3 | 2.4098 |
| 1371785_at | Tnfrsf12a | tumor necrosis factor receptor superfamily, member 12a | 2.3378 |
| 1388539_at | Pkp2 | plakophilin 2 | 2.3106 |
| 1389002_at | Tln1 | talin 1 | 2.2627 |
| 1371664_at | Pxn | paxillin | 2.2373 |
| 1380190_at | F8 | coagulation factor VIII, procoagulant component | 2.2221 |
| 1397511_at | Ttyh1 | tweety homolog 1 (Drosophila) | 2.2184 |
| 1387424_at | Cntn2 | contactin 2 (axonal) | 2.1679 |
| 1369607_at | Fgf6 | fibroblast growth factor 6 | 2.1594 |
| 1395015_at | F8 | coagulation factor VIII, procoagulant component | 2.1392 |
| 1393067_at | Tek | TEK tyrosine kinase, endothelial | 2.1025 |
| 1368186_a_at | Syk | spleen tyrosine kinase | 2.0797 |
