# Supplementary material for: Transcriptome analysis of amoeboid and ramified microglia isolated from the corpus callosum of rat brain
Source: BMC Neurosci. 2012 Jun 14;13:64. doi: 10.1186/1471-2202-13-64 (PMC3441342; doi:10.1186/1471-2202-13-64)
Supplement: Additional file 7 — Sheet S6. Monocytic genes enriched in AMC and RMC. [file 1471-2202-13-64-S7.docx]

**Monocytic genes specific to AMC**

| **Gene Symbol** | **Gene Name** | **Signal intensity in monocytes** |
| --- | --- | --- |
| AA809056 | actin, beta | 26674.44 |
| ACTB | actin, beta | 26156.96 |
| RPS2 | ribosomal protein S2 pseudogene 8; ribosomal protein S2 pseudogene 11; ribosomal protein S2 pseudogene 5; ribosomal protein S2 pseudogene 12; ribosomal protein S2 pseudogene 51; ribosomal protein S2 pseudogene 17; ribosomal protein S2 pseudogene 55; ribosomal protein S2 pseudogene 20; ribosomal protein S2 | 23821.66 |
| RPS18 | ribosomal protein S18 pseudogene 12; ribosomal protein S18 pseudogene 5; ribosomal protein S18 | 21936.04 |
| RPS3A | ribosomal protein S3A pseudogene 5; ribosomal protein S3a pseudogene 47; ribosomal protein S3a pseudogene 49; ribosomal protein S3A; hypothetical LOC100131699; hypothetical LOC100130107 | 21572.45 |
| BE561479 | glyceraldehyde-3-phosphate dehydrogenase-like 6; hypothetical protein LOC100133042; glyceraldehyde-3-phosphate dehydrogenase | 21161.19 |
| RPL10 | ribosomal protein L10; ribosomal protein L10 pseudogene 15; ribosomal protein L10 pseudogene 6; ribosomal protein L10 pseudogene 16; ribosomal protein L10 pseudogene 9 | 20836.91 |
| AI925635 | ribosomal protein S3A pseudogene 5; ribosomal protein S3a pseudogene 47; ribosomal protein S3a pseudogene 49; ribosomal protein S3A; hypothetical LOC100131699; hypothetical LOC100130107 | 20563.33 |
| RPL7 | ribosomal protein L7 pseudogene 26; ribosomal protein L7 pseudogene 16; ribosomal protein L7; ribosomal protein L7 pseudogene 32; ribosomal protein L7 pseudogene 23; ribosomal protein L7 pseudogene 24; ribosomal protein L7 pseudogene 20 | 20396.63 |
| RPL7A | ribosomal protein L7a pseudogene 70; ribosomal protein L7a; ribosomal protein L7a pseudogene 30; ribosomal protein L7a pseudogene 66; ribosomal protein L7a pseudogene 27; ribosomal protein L7a pseudogene 11; ribosomal protein L7a pseudogene 62 | 19475 |
| CFL1 | cofilin 1 (non-muscle) | 18148.69 |
| AI799007 | ribosomal protein S12; ribosomal protein S12 pseudogene 4; ribosomal protein S12 pseudogene 11; ribosomal protein S12 pseudogene 9 | 18098.26 |
| GAPDH | glyceraldehyde-3-phosphate dehydrogenase-like 6; hypothetical protein LOC100133042; glyceraldehyde-3-phosphate dehydrogenase | 17827.03 |
| RPS13 | ribosomal protein S13 pseudogene 8; ribosomal protein S13; ribosomal protein S13 pseudogene 2 | 17429.59 |
| RPL3 | ribosomal protein L3; similar to 60S ribosomal protein L3 (L4) | 17000.91 |
| RPL28 | ribosomal protein L28 | 16962.88 |
| GAPDH | glyceraldehyde-3-phosphate dehydrogenase-like 6; hypothetical protein LOC100133042; glyceraldehyde-3-phosphate dehydrogenase | 16618.35 |
| BE963164 | eukaryotic translation elongation factor 1 gamma | 16423.07 |
| RPL3 | ribosomal protein L3; similar to 60S ribosomal protein L3 (L4) | 16266.81 |
| RPL3 | ribosomal protein L3; similar to 60S ribosomal protein L3 (L4) | 16137.43 |
| PABPC1 | poly(A) binding protein, cytoplasmic pseudogene 5; poly(A) binding protein, cytoplasmic 1 | 16065.81 |
| ACTG1 | actin, gamma 1 | 15824.22 |
| OSTM1 | ribosomal protein L3 pseudogene 7 | 15770.26 |
| ACTG1 | actin, gamma 1 | 15586.13 |
| AF119850 | eukaryotic translation elongation factor 1 gamma | 15083.36 |
| RPS20 | ribosomal protein S20 | 15057.91 |
| ACTG1 | actin, gamma 1 | 15053.39 |
| CXCR4 | chemokine (C-X-C motif) receptor 4 | 14974.42 |
| ACTG1 | actin, gamma 1 | 14956.24 |
| ACTG1 | actin, gamma 1 | 14606.57 |
| MCL1 | myeloid cell leukemia sequence 1 (BCL2-related) | 14521.93 |
| ACTG1 | actin, gamma 1 | 14428.58 |
| CXCR4 | chemokine (C-X-C motif) receptor 4 | 14359.47 |
| EEF1G | eukaryotic translation elongation factor 1 gamma | 13914.51 |
| ACTG1 | actin, gamma 1 | 13796.08 |
| RPS2 | ribosomal protein S2 pseudogene 8; ribosomal protein S2 pseudogene 11; ribosomal protein S2 pseudogene 5; ribosomal protein S2 pseudogene 12; ribosomal protein S2 pseudogene 51; ribosomal protein S2 pseudogene 17; ribosomal protein S2 pseudogene 55; ribosomal protein S2 pseudogene 20; ribosomal protein S2 | 13598.05 |
| RPL18 | ribosomal protein L18 | 13035 |
| RPL4 | ribosomal protein L4; ribosomal protein L4 pseudogene 5; ribosomal protein L4 pseudogene 4 | 13001.9 |
| RPL4 | ribosomal protein L4; ribosomal protein L4 pseudogene 5; ribosomal protein L4 pseudogene 4 | 12225.11 |
| KIAA1128 | ribosomal protein S3A pseudogene 5; ribosomal protein S3a pseudogene 47; ribosomal protein S3a pseudogene 49; ribosomal protein S3A; hypothetical LOC100131699; hypothetical LOC100130107 | 12214.8 |
| ACTG1 | actin, gamma 1 | 12179.47 |
| GABARAP | GABA(A) receptor-associated protein | 11561.88 |
| RPS20 | ribosomal protein S20 | 11473.08 |
| RPL3 | ribosomal protein L3; similar to 60S ribosomal protein L3 (L4) | 11343.31 |
| GNB2L1 | guanine nucleotide binding protein (G protein), beta polypeptide 2-like 1 | 11316.29 |
| YBX1 | Y box binding protein 1 | 10586.31 |
| EEF1B2 | eukaryotic translation elongation factor 1 beta 2; eukaryotic translation elongation factor 1 beta 2-like | 10557.02 |
| RPL15 | ribosomal protein L15 pseudogene 22; ribosomal protein L15 pseudogene 18; ribosomal protein L15 pseudogene 17; ribosomal protein L15 pseudogene 3; ribosomal protein L15 pseudogene 7; ribosomal protein L15 | 10553.65 |
| RPS5 | ribosomal protein S5 | 10547.09 |
| RPS11 | ribosomal protein S11 pseudogene 5; ribosomal protein S11 | 10481.08 |
| PFN1 | profilin 1 | 10347.09 |
| RPS9 | ribosomal protein S9; ribosomal protein S9 pseudogene 4 | 10251.65 |
| RPL18A | ribosomal protein L18a pseudogene 3 | 10043.28 |
| RPS9 | ribosomal protein S9; ribosomal protein S9 pseudogene 4 | 9790.077 |
| CHST6 | coactosin-like 1 (Dictyostelium) | 9766.338 |
| Sept9 | septin 9 | 9595.756 |
| RPL15 | ribosomal protein L15 pseudogene 22; ribosomal protein L15 pseudogene 18; ribosomal protein L15 pseudogene 17; ribosomal protein L15 pseudogene 3; ribosomal protein L15 pseudogene 7; ribosomal protein L15 | 9537.651 |
| BE966374 | Y box binding protein 1 | 9447.42 |
| TUBA1C | tubulin, alpha 1c | 9418.413 |
| SH3BGRL3 | SH3 domain binding glutamic acid-rich protein like 3 | 9395.202 |
| PCBP1 | poly(rC) binding protein 1 | 9238.988 |
| CXCR4 | chemokine (C-X-C motif) receptor 4 | 9131.201 |
| TUBA1C | tubulin, alpha 1c | 9116.709 |
| CMTM6 | CKLF-like MARVEL transmembrane domain containing 6 | 8878.223 |
| VCAN | versican | 8753.031 |
| ARPC2 | actin related protein 2/3 complex, subunit 2, 34kDa | 8399.619 |
| VCAN | versican | 8325.302 |
| RPL7 | ribosomal protein L7 pseudogene 26; ribosomal protein L7 pseudogene 16; ribosomal protein L7; ribosomal protein L7 pseudogene 32; ribosomal protein L7 pseudogene 23; ribosomal protein L7 pseudogene 24; ribosomal protein L7 pseudogene 20 | 8101.48 |
| MCL1 | myeloid cell leukemia sequence 1 (BCL2-related) | 7821.734 |
| PABPCP3 | poly(A) binding protein, cytoplasmic pseudogene 5; poly(A) binding protein, cytoplasmic 1 | 7745.039 |
| RAB1A | RAB1A, member RAS oncogene family | 7629.17 |
| ID2 | inhibitor of DNA binding 2, dominant negative helix-loop-helix protein | 7595.235 |
| MAPRE1 | microtubule-associated protein, RP/EB family, member 1 | 7481.985 |
| PSME1 | proteasome (prosome, macropain) activator subunit 1 (PA28 alpha) | 7435.124 |
| EIF3F | eukaryotic translation initiation factor 3, subunit F; similar to hCG2040283 | 7171.419 |
| LDHA | lactate dehydrogenase A | 7058.81 |
| JTB | jumping translocation breakpoint | 6980.99 |
| EIF3H | eukaryotic translation initiation factor 3, subunit H | 6954.922 |
| RPL4 | ribosomal protein L4; ribosomal protein L4 pseudogene 5; ribosomal protein L4 pseudogene 4 | 6903.76 |
| EEF2 | eukaryotic translation elongation factor 2 | 6818.024 |
| TOMM7 | hypothetical LOC100129478; chromosome 4 open reading frame 46 | 6795.628 |
| CAPNS1 | calpain, small subunit 1 | 6501.847 |
| NAP1L1 | nucleosome assembly protein 1-like 1 | 6253.808 |
| BASP1 | brain abundant, membrane attached signal protein 1 | 6212.216 |
| ZDHHC7 | zinc finger, DHHC-type containing 7 | 6192.996 |
| SUMO2 | SMT3 suppressor of mif two 3 homolog 2 (S. cerevisiae) pseudogene; SMT3 suppressor of mif two 3 homolog 2 (S. cerevisiae); SMT3 suppressor of mif two 3 homolog 3 (S. cerevisiae) | 6156.485 |
| JTB | jumping translocation breakpoint | 6126.577 |
| TALDO1 | transaldolase 1 | 6059.018 |
| ARPC2 | actin related protein 2/3 complex, subunit 2, 34kDa | 6037.919 |
| YWHAZ | tyrosine 3-monooxygenase/tryptophan 5-monooxygenase activation protein, zeta polypeptide | 5992.925 |
| BG292367 | ras-related C3 botulinum toxin substrate 1 (rho family, small GTP binding protein Rac1) | 5950.331 |
| NAP1L1 | nucleosome assembly protein 1-like 1 | 5790.004 |
| TUBA1A | tubulin, alpha 1a | 5765.715 |
| NAP1L1 | nucleosome assembly protein 1-like 1 | 5675.685 |
| EIF4G2 | eukaryotic translation initiation factor 4 gamma, 2 | 5663.222 |
| HINT1 | histidine triad nucleotide binding protein 1 | 5393.879 |
| BG435404 | ADP-ribosylation factor-like 4C | 5388.096 |
| ACTR3 | ARP3 actin-related protein 3 homolog (yeast) | 5376.732 |
| HINT1 | histidine triad nucleotide binding protein 1 | 5218.545 |
| HNRNPD | heterogeneous nuclear ribonucleoprotein D (AU-rich element RNA binding protein 1, 37kDa) | 5147.527 |
| HNRNPK | heterogeneous nuclear ribonucleoprotein K; similar to heterogeneous nuclear ribonucleoprotein K | 5123.188 |
| JTB | jumping translocation breakpoint | 5084.224 |
| HNRNPK | heterogeneous nuclear ribonucleoprotein K; similar to heterogeneous nuclear ribonucleoprotein K | 5059.466 |
| WDR1 | WD repeat domain 1 | 5044.459 |
| EEF2 | eukaryotic translation elongation factor 2 | 4982.02 |
| MAPK6 | mitogen-activated protein kinase 6 | 4971.424 |
| HSPA8 | heat shock 70kDa protein 8 | 4933.54 |
| KARS | lysyl-tRNA synthetase | 4802.778 |
| YWHAB | tyrosine 3-monooxygenase/tryptophan 5-monooxygenase activation protein, beta polypeptide | 4798.768 |
| EIF3C | eukaryotic translation initiation factor 3, subunit C-like | 4759.659 |
| SUMO3 | SMT3 suppressor of mif two 3 homolog 2 (S. cerevisiae) pseudogene; SMT3 suppressor of mif two 3 homolog 2 (S. cerevisiae); SMT3 suppressor of mif two 3 homolog 3 (S. cerevisiae) | 4748.275 |
| L48784 | ribosomal protein S2 pseudogene 8; ribosomal protein S2 pseudogene 11; ribosomal protein S2 pseudogene 5; ribosomal protein S2 pseudogene 12; ribosomal protein S2 pseudogene 51; ribosomal protein S2 pseudogene 17; ribosomal protein S2 pseudogene 55; ribosomal protein S2 pseudogene 20; ribosomal protein S2 | 4730 |
| RPS11 | ribosomal protein S11 pseudogene 5; ribosomal protein S11 | 4725.569 |
| NONO | non-POU domain containing, octamer-binding | 4612.957 |
| HECA | headcase homolog (Drosophila) | 4526.448 |
| C14orf166 | chromosome 14 open reading frame 166 | 4394.988 |
| ARL4C | ADP-ribosylation factor-like 4C | 4376.133 |
| ATP6V0D1 | ATPase, H+ transporting, lysosomal 38kDa, V0 subunit d1 | 4370.573 |
| RAB31 | RAB31, member RAS oncogene family | 4339.323 |
| ENO1 | enolase 1, (alpha) | 4320.887 |
| HSP90AB1 | heat shock protein 90kDa alpha (cytosolic), class B member 1 | 4315.772 |
| CORO1A | coronin, actin binding protein, 1A | 4284.676 |
| AP3S1 | adaptor-related protein complex 3, sigma 1 subunit | 4260.407 |
| EIF4H | eukaryotic translation initiation factor 4H | 4233.903 |
| BE880245 | glucosamine (N-acetyl)-6-sulfatase | 4161.114 |
| SNORD119 | small nuclear ribonucleoprotein polypeptides B and B1 | 4142.913 |
| EIF3C | eukaryotic translation initiation factor 3, subunit C-like | 4142.016 |
| SSR1 | signal sequence receptor, alpha | 4125.66 |
| RAP1B | RAP1B, member of RAS oncogene family | 4120.803 |
| USP3 | ubiquitin specific peptidase 3 | 4111.313 |
| ARPC5 | actin related protein 2/3 complex, subunit 5, 16kDa | 4018.905 |
| YWHAZ | tyrosine 3-monooxygenase/tryptophan 5-monooxygenase activation protein, zeta polypeptide | 4011.752 |
| CUGBP2 | CUG triplet repeat, RNA binding protein 2 | 4006.99 |
| NPM1 | nucleophosmin 1 (nucleolar phosphoprotein B23, numatrin) pseudogene 21; hypothetical LOC100131044; similar to nucleophosmin 1; nucleophosmin (nucleolar phosphoprotein B23, numatrin) | 4006.986 |
| YWHAZ | tyrosine 3-monooxygenase/tryptophan 5-monooxygenase activation protein, zeta polypeptide | 3986.961 |
| LASP1 | LIM and SH3 protein 1 | 3985.366 |
| RAC1 | ras-related C3 botulinum toxin substrate 1 (rho family, small GTP binding protein Rac1) | 3980.139 |
| MARCKSL1 | MARCKS-like 1 | 3906.913 |
| NME2 | non-metastatic cells 1, protein (NM23A) expressed in; NME1-NME2 readthrough transcript; non-metastatic cells 2, protein (NM23B) expressed in | 3895.271 |
| AL121934 | ribosomal protein L10; ribosomal protein L10 pseudogene 15; ribosomal protein L10 pseudogene 6; ribosomal protein L10 pseudogene 16; ribosomal protein L10 pseudogene 9 | 3876.875 |
| PPA1 | pyrophosphatase (inorganic) 1 | 3873.736 |
| ZMIZ1 | zinc finger, MIZ-type containing 1 | 3873.629 |
| ARPC2 | actin related protein 2/3 complex, subunit 2, 34kDa | 3842.325 |
| ECOP | similar to EGFR-coamplified and overexpressed protein; EGFR-coamplified and overexpressed protein | 3818.708 |
| ZNF207 | zinc finger protein 207 | 3773.038 |
| HSPA8 | heat shock 70kDa protein 8 | 3749.782 |
| NXT1 | NTF2-like export factor 1 | 3749.544 |
| EIF3D | eukaryotic translation initiation factor 3, subunit D | 3748.552 |
| GNB1 | guanine nucleotide binding protein (G protein), beta polypeptide 1 | 3691.842 |
| SH3BGRL | SH3 domain binding glutamic acid-rich protein like | 3680.698 |
| SET | SET nuclear oncogene; similar to SET translocation | 3649.544 |
| NPM1 | nucleophosmin 1 (nucleolar phosphoprotein B23, numatrin) pseudogene 21; hypothetical LOC100131044; similar to nucleophosmin 1; nucleophosmin (nucleolar phosphoprotein B23, numatrin) | 3623.448 |
| SH3BGRL | SH3 domain binding glutamic acid-rich protein like | 3570.84 |
| IFNGR2 | interferon gamma receptor 2 (interferon gamma transducer 1) | 3553.107 |
| VCAN | versican | 3541.604 |
| PSMA1 | proteasome (prosome, macropain) subunit, alpha type, 1 | 3536.719 |
| SLA | Src-like-adaptor | 3523.921 |
| HSPA8 | heat shock 70kDa protein 8 | 3515.278 |
| EIF3CL | eukaryotic translation initiation factor 3, subunit C-like | 3498.145 |
| AI278616 | SET nuclear oncogene; similar to SET translocation | 3486.426 |
| DDOST | dolichyl-diphosphooligosaccharide-protein glycosyltransferase | 3484.155 |
| EHD1 | EH-domain containing 1 | 3444.771 |
| STARD7 | StAR-related lipid transfer (START) domain containing 7 | 3426.091 |
| SSR4 | signal sequence receptor, delta (translocon-associated protein delta) | 3400.772 |
| PSMG2 | proteasome (prosome, macropain) assembly chaperone 2 | 3333.182 |
| SLC7A5 | solute carrier family 7 (cationic amino acid transporter, y+ system), member 5 | 3327.208 |
| PPP6C | protein phosphatase 6, catalytic subunit | 3326.048 |
| AV702810 | SET nuclear oncogene; similar to SET translocation | 3293.195 |
| MARCKS | myristoylated alanine-rich protein kinase C substrate | 3244.314 |
| UBE2L3 | ubiquitin-conjugating enzyme E2L 3 | 3228.823 |
| SUMO2 | SMT3 suppressor of mif two 3 homolog 4 (S. cerevisiae) | 3212.3 |
| RHOQ | ras homolog gene family, member Q; similar to small GTP binding protein TC10 | 3192.798 |
| TNRC6B | ribosomal protein L7 pseudogene 26; ribosomal protein L7 pseudogene 16; ribosomal protein L7; ribosomal protein L7 pseudogene 32; ribosomal protein L7 pseudogene 23; ribosomal protein L7 pseudogene 24; ribosomal protein L7 pseudogene 20 | 3175.454 |
| PSMA1 | proteasome (prosome, macropain) subunit, alpha type, 1 | 3166.373 |
| PPP6C | protein phosphatase 6, catalytic subunit | 3141.932 |
| LOC130074 | family with sequence similarity 168, member B | 3116.281 |
| UBE2D2 | ubiquitin-conjugating enzyme E2D 2 (UBC4/5 homolog, yeast) | 3099.57 |
| REEP5 | receptor accessory protein 5 | 3072.975 |
| M30448 | fibrillarin | 3071.665 |
| EHD1 | EH-domain containing 1 | 3059.046 |
| SFPQ | splicing factor proline/glutamine-rich (polypyrimidine tract binding protein associated) | 3054.054 |
| BE789881 | RAB31, member RAS oncogene family | 3042.162 |
| ARL4C | ADP-ribosylation factor-like 4C | 3016.425 |
| VCAN | versican | 3012.893 |
| CLTA | clathrin, light chain (Lca) | 2994.388 |
| CSDE1 | cold shock domain containing E1, RNA-binding | 2965.034 |
| PABPC4 | poly(A) binding protein, cytoplasmic 4 (inducible form) | 2895.137 |
| ETF1 | eukaryotic translation termination factor 1 | 2867.999 |
| TUBB | tubulin, beta; similar to tubulin, beta 5; tubulin, beta pseudogene 2; tubulin, beta pseudogene 1 | 2856.972 |
| SQSTM1 | sequestosome 1 | 2856.58 |
| ADFP | adipose differentiation-related protein | 2850.109 |
| CD47 | CD47 molecule | 2842.594 |
| ZNF207 | zinc finger protein 207 | 2833.241 |
| NPM1 | nucleophosmin 1 (nucleolar phosphoprotein B23, numatrin) pseudogene 21; hypothetical LOC100131044; similar to nucleophosmin 1; nucleophosmin (nucleolar phosphoprotein B23, numatrin) | 2822.519 |
| PSMA1 | proteasome (prosome, macropain) subunit, alpha type, 1 | 2796.824 |
| RHOQ | ras homolog gene family, member Q; similar to small GTP binding protein TC10 | 2790.333 |
| YWHAZ | tyrosine 3-monooxygenase/tryptophan 5-monooxygenase activation protein, zeta polypeptide | 2788.695 |
| C19orf56 | chromosome 19 open reading frame 56 | 2782.527 |
| KHDRBS1 | KH domain containing, RNA binding, signal transduction associated 1 | 2762.355 |
| SNN | stannin | 2750.509 |
| WDR1 | WD repeat domain 1 | 2726.627 |
| ETF1 | eukaryotic translation termination factor 1 | 2719.74 |
| PSMD6 | proteasome (prosome, macropain) 26S subunit, non-ATPase, 6 | 2716.741 |
| ARF4 | ADP-ribosylation factor 4 | 2715.679 |
| PAPOLA | poly(A) polymerase alpha | 2674.225 |
| AP2S1 | adaptor-related protein complex 2, sigma 1 subunit | 2664.566 |
| NM_030673 | SEC13 homolog (S. cerevisiae) | 2625.997 |
| PLEKHB2 | pleckstrin homology domain containing, family B (evectins) member 2 | 2625.008 |
| RHOQ | ras homolog gene family, member Q; similar to small GTP binding protein TC10 | 2621.294 |
| KLF6 | Kruppel-like factor 6 | 2612.352 |
| MIF | hypothetical protein LOC284889 | 2608.373 |
| PRKCB1 | protein kinase C, beta | 2578.584 |
| RNF138 | ring finger protein 138 | 2575.77 |
| SRP72 | signal recognition particle 72kDa | 2534.669 |
| RAB31 | RAB31, member RAS oncogene family | 2528.851 |
| IVNS1ABP | influenza virus NS1A binding protein | 2524.492 |
| CSK | c-src tyrosine kinase | 2521.708 |
| ID2 | inhibitor of DNA binding 2, dominant negative helix-loop-helix protein | 2515.583 |
| SFRS10 | transformer 2 beta homolog (Drosophila) | 2491.699 |
| MGST3 | microsomal glutathione S-transferase 3 | 2471.359 |
| PSMB7 | proteasome (prosome, macropain) subunit, beta type, 7 | 2464.129 |
| TUBB | tubulin, beta; similar to tubulin, beta 5; tubulin, beta pseudogene 2; tubulin, beta pseudogene 1 | 2459.719 |
| HMGN3 | high mobility group nucleosomal binding domain 3 | 2435.617 |
| GNB1 | guanine nucleotide binding protein (G protein), beta polypeptide 1 | 2431.901 |
| PQLC1 | PQ loop repeat containing 1 | 2418.268 |
| UBE2I | ubiquitin-conjugating enzyme E2I (UBC9 homolog, yeast) | 2394.217 |
| DCN | signal sequence receptor, alpha | 2375.507 |
| CSDE1 | cold shock domain containing E1, RNA-binding | 2370.885 |
| AP2S1 | adaptor-related protein complex 2, sigma 1 subunit | 2366.723 |
| U88968 | enolase 1, (alpha) | 2359.383 |
| FAM49B | family with sequence similarity 49, member B | 2354.564 |
| TBCA | tubulin folding cofactor A | 2335.587 |
| HINT1 | histidine triad nucleotide binding protein 1 | 2331.447 |
| GNAI3 | guanine nucleotide binding protein (G protein), alpha inhibiting activity polypeptide 3 | 2329.34 |
| RAB11A | RAB11A, member RAS oncogene family | 2300.161 |
| ATXN10 | ataxin 10 | 2296.552 |
| DYNLT1 | dynein, light chain, Tctex-type 1 | 2288.789 |
| SET | SET nuclear oncogene; similar to SET translocation | 2284.233 |
| BE566290 | ARP2 actin-related protein 2 homolog (yeast) | 2275.271 |
| GYG1 | glycogenin 1 | 2260.633 |
| KARS | lysyl-tRNA synthetase | 2255.732 |
| NDRG1 | N-myc downstream regulated 1 | 2245.925 |
| TCEA1 | transcription elongation factor A (SII), 1 pseudogene 2; transcription elongation factor A (SII), 1 | 2231.356 |
| GYG1 | glycogenin 1 | 2229.725 |
| ESD | esterase D/formylglutathione hydrolase | 2218.588 |
| EHD1 | EH-domain containing 1 | 2207.484 |
| SURF1 | surfeit 1 | 2186.13 |
| MAX | MYC associated factor X | 2181.307 |
| CCT8 | similar to chaperonin containing TCP1, subunit 8 (theta); chaperonin containing TCP1, subunit 8 (theta) | 2175.243 |
| SFRS10 | transformer 2 beta homolog (Drosophila) | 2174.901 |
| TSG101 | tumor susceptibility gene 101 | 2168.325 |
| ZC3H15 | zinc finger CCCH-type containing 15 | 2165.516 |
| RBMX | similar to RNA binding motif protein, X-linked; similar to hCG2011544; RNA binding motif protein, X-linked | 2154.911 |
| X81636 | clathrin, light chain (Lca) | 2139.53 |
| CLTA | clathrin, light chain (Lca) | 2138.658 |
| OAT | ornithine aminotransferase (gyrate atrophy) | 2125.939 |
| CCT4 | chaperonin containing TCP1, subunit 4 (delta) | 2112.819 |
| CMPK1 | cytidine monophosphate (UMP-CMP) kinase 1, cytosolic | 2109.585 |
| TUFM | Tu translation elongation factor, mitochondrial | 2106.645 |
| YWHAB | tyrosine 3-monooxygenase/tryptophan 5-monooxygenase activation protein, beta polypeptide | 2096.643 |
| EIF4EBP2 | eukaryotic translation initiation factor 4E binding protein 2 | 2077.143 |
| AA149594 | Kruppel-like factor 11 | 2076.009 |
| JUN | jun oncogene | 2075.837 |
| ERH | enhancer of rudimentary homolog (Drosophila) | 2071.242 |
| SNRPB2 | small nuclear ribonucleoprotein polypeptide B'' | 2028.278 |
| BG505670 | heterogeneous nuclear ribonucleoprotein A3 pseudogene 1 | 2027.407 |
| SUMO2 | SMT3 suppressor of mif two 3 homolog 2 (S. cerevisiae) pseudogene; SMT3 suppressor of mif two 3 homolog 2 (S. cerevisiae); SMT3 suppressor of mif two 3 homolog 3 (S. cerevisiae) | 2025.05 |
| HSD17B10 | hydroxysteroid (17-beta) dehydrogenase 10 | 2022.508 |
| HSBP1 | heat shock factor binding protein 1 | 2009.502 |
| DPYSL2 | dihydropyrimidinase-like 2 | 2006.907 |
| CORO1C | coronin, actin binding protein, 1C | 2005.832 |
| KLF6 | Kruppel-like factor 6 | 1995.601 |
| TXN | thioredoxin | 1995.246 |
| FYN | FYN oncogene related to SRC, FGR, YES | 1974.83 |
| DCTN3 | dynactin 3 (p22) | 1959.877 |
| NM_017698 | hypothetical protein LOC100130886 | 1956.961 |
| GLRX | glutaredoxin (thioltransferase) | 1954.508 |
| SNX6 | sorting nexin 6 | 1946.261 |
| KHDRBS1 | KH domain containing, RNA binding, signal transduction associated 1 | 1937.571 |
| SLA | Src-like-adaptor | 1927.366 |
| SET | SET nuclear oncogene; similar to SET translocation | 1925.908 |
| HNRNPD | heterogeneous nuclear ribonucleoprotein D (AU-rich element RNA binding protein 1, 37kDa) | 1925.548 |
| PRDX1 | peroxiredoxin 1 | 1921.001 |
| AP2S1 | adaptor-related protein complex 2, sigma 1 subunit | 1907.287 |
| CBFB | core-binding factor, beta subunit | 1891.444 |
| MDH2 | malate dehydrogenase 2, NAD (mitochondrial) | 1881.701 |
| WBP11 | WW domain binding protein 11 | 1870.098 |
| RNF10 | ring finger protein 10 | 1866.699 |
| BF691447 | UDP-Gal:betaGlcNAc beta 1,4- galactosyltransferase, polypeptide 5 | 1855.122 |
| IDI1 | isopentenyl-diphosphate delta isomerase 1 | 1850.28 |
| TUBB | tubulin, beta; similar to tubulin, beta 5; tubulin, beta pseudogene 2; tubulin, beta pseudogene 1 | 1848.798 |
| ZC3H15 | zinc finger CCCH-type containing 15 | 1844.077 |
| ACTR3 | ARP3 actin-related protein 3 homolog (yeast) | 1831.356 |
| PSMB4 | proteasome (prosome, macropain) subunit, beta type, 4 | 1822.771 |
| MCL1 | myeloid cell leukemia sequence 1 (BCL2-related) | 1814.955 |
| SNRPB | small nuclear ribonucleoprotein polypeptides B and B1 | 1805.696 |
| TMEM183A | transmembrane protein 183A; transmembrane protein 183B | 1795.76 |
| B4GALT5 | UDP-Gal:betaGlcNAc beta 1,4- galactosyltransferase, polypeptide 5 | 1781.589 |
| ENY2 | enhancer of yellow 2 homolog (Drosophila) | 1776.902 |
| PAPOLA | poly(A) polymerase alpha | 1765.878 |
| NAP1L1 | nucleosome assembly protein 1-like 1 | 1763.194 |
| GNB2 | guanine nucleotide binding protein (G protein), beta polypeptide 2 | 1759.676 |
| KLF2 | Kruppel-like factor 2 (lung) | 1745.669 |
| PDIA6 | protein disulfide isomerase family A, member 6 | 1725.604 |
| GNS | glucosamine (N-acetyl)-6-sulfatase | 1719.68 |
| C20orf11 | chromosome 20 open reading frame 11 | 1708.788 |
| SH3BP5 | SH3-domain binding protein 5 (BTK-associated) | 1678.868 |
| FAM89B | family with sequence similarity 89, member B | 1677.804 |
| SLBP | stem-loop binding protein | 1669.704 |
| AFTPH | aftiphilin | 1652.223 |
| ARF5 | ADP-ribosylation factor 5 | 1642.669 |
| CD47 | CD47 molecule | 1642.394 |
| IDI1 | isopentenyl-diphosphate delta isomerase 1 | 1631.216 |
| PSMA3 | proteasome (prosome, macropain) subunit, alpha type, 3 | 1622.023 |
| LYPLA1 | lysophospholipase I | 1615.377 |
| PNO1 | partner of NOB1 homolog (S. cerevisiae) | 1614.235 |
| STAT1 | signal transducer and activator of transcription 1, 91kDa | 1610.192 |
| C11orf58 | chromosome 11 open reading frame 58 pseudogene; chromosome 11 open reading frame 58 | 1605.916 |
| MSL-1 | male-specific lethal 1 homolog (Drosophila) | 1596.424 |
| GTF2A2 | general transcription factor IIA, 2, 12kDa | 1592.707 |
| NONO | non-POU domain containing, octamer-binding | 1584.256 |
| DNAJA1 | DnaJ (Hsp40) homolog, subfamily A, member 1 | 1579.719 |
| EIF3A | eukaryotic translation initiation factor 3, subunit A | 1576.629 |
| OXSR1 | oxidative-stress responsive 1 | 1571.342 |
| DUSP2 | dual specificity phosphatase 2 | 1549.481 |
| ALG5 | asparagine-linked glycosylation 5, dolichyl-phosphate beta-glucosyltransferase homolog (S. cerevisiae) | 1516.229 |
| UBE2E3 | ubiquitin-conjugating enzyme E2E 3 (UBC4/5 homolog, yeast) | 1503.146 |
| PDIA6 | protein disulfide isomerase family A, member 6 | 1500.976 |
| PDCD10 | programmed cell death 10 | 1497.014 |
| SRP72 | signal recognition particle 72kDa | 1480.268 |
| PSMA4 | proteasome (prosome, macropain) subunit, alpha type, 4 | 1479.612 |
| RAB7A | RAB7A, member RAS oncogene family | 1477.53 |
| AKR1A1 | aldo-keto reductase family 1, member A1 (aldehyde reductase) | 1474.28 |
| PSMD2 | proteasome (prosome, macropain) 26S subunit, non-ATPase, 2 | 1470.241 |
| LRRC59 | leucine rich repeat containing 59 | 1467.46 |
| HNRNPA3 | heterogeneous nuclear ribonucleoprotein A3 pseudogene 1 | 1463.841 |
| RAN | RAN, member RAS oncogene family | 1463.369 |
| GNB1 | guanine nucleotide binding protein (G protein), beta polypeptide 1 | 1457.819 |
| PIK3CD | phosphoinositide-3-kinase, catalytic, delta polypeptide | 1454.635 |
| TUBB3 | tubulin, beta 3; melanocortin 1 receptor (alpha melanocyte stimulating hormone receptor) | 1446.299 |
| MCL1 | myeloid cell leukemia sequence 1 (BCL2-related) | 1423.622 |
| LY6G6C | dimethylarginine dimethylaminohydrolase 2 | 1422.766 |
| MAP3K7IP2 | mitogen-activated protein kinase kinase kinase 7 interacting protein 2 | 1422.655 |
| C11orf58 | chromosome 11 open reading frame 58 pseudogene; chromosome 11 open reading frame 58 | 1417.27 |
| RAB14 | RAB14, member RAS oncogene family | 1417.121 |
| RAB1A | RAB1A, member RAS oncogene family | 1412.816 |
| ZNF580 | zinc finger protein 580 | 1409.401 |
| MAP3K7IP2 | mitogen-activated protein kinase kinase kinase 7 interacting protein 2 | 1395.6 |
| DDAH2 | dimethylarginine dimethylaminohydrolase 2 | 1380.085 |
| FDFT1 | farnesyl-diphosphate farnesyltransferase 1 | 1377.669 |
| DCTN6 | dynactin 6 | 1376.562 |
| UBE2D2 | ubiquitin-conjugating enzyme E2D 2 (UBC4/5 homolog, yeast) | 1375.713 |
| AKR1B1 | aldo-keto reductase family 1, member B1 (aldose reductase) | 1374.322 |
| SUMO3 | SMT3 suppressor of mif two 3 homolog 2 (S. cerevisiae) pseudogene; SMT3 suppressor of mif two 3 homolog 2 (S. cerevisiae); SMT3 suppressor of mif two 3 homolog 3 (S. cerevisiae) | 1373.268 |
| YTHDF2 | YTH domain family, member 2 | 1368.058 |
| SYNCRIP | synaptotagmin binding, cytoplasmic RNA interacting protein | 1366.052 |
| RBBP7 | retinoblastoma binding protein 7 | 1364.396 |
| RABAC1 | Rab acceptor 1 (prenylated) | 1360.831 |
| PSMB2 | proteasome (prosome, macropain) subunit, beta type, 2 | 1359.73 |
| DNAJA1 | DnaJ (Hsp40) homolog, subfamily A, member 1 | 1356.636 |
| YWHAB | tyrosine 3-monooxygenase/tryptophan 5-monooxygenase activation protein, beta polypeptide | 1355.786 |
| RAB7A | RAB7A, member RAS oncogene family | 1353.565 |
| FLJ22536 | SRY (sex determining region Y)-box 4 | 1353.348 |
| SEC63 | SEC63 homolog (S. cerevisiae) | 1345.338 |
| AKT1 | v-akt murine thymoma viral oncogene homolog 1 | 1345.301 |
| HNRNPD | heterogeneous nuclear ribonucleoprotein D (AU-rich element RNA binding protein 1, 37kDa) | 1339.206 |
| HN1 | hematological and neurological expressed 1 | 1330.797 |
| ENC1 | ectodermal-neural cortex (with BTB-like domain) | 1329.147 |
| MARCKS | myristoylated alanine-rich protein kinase C substrate | 1325.439 |
| VEZF1 | vascular endothelial zinc finger 1 | 1322.008 |
| UBE2D1 | ubiquitin-conjugating enzyme E2D 1 (UBC4/5 homolog, yeast) | 1320.135 |
| CGGBP1 | CGG triplet repeat binding protein 1 | 1308.657 |
| HSBP1 | heat shock factor binding protein 1 | 1299.568 |
| DSTN | destrin (actin depolymerizing factor) | 1295.179 |
| CAPRIN1 | cell cycle associated protein 1 | 1292.348 |
| ACTR1A | ARP1 actin-related protein 1 homolog A, centractin alpha (yeast) | 1290.358 |
| CCT2 | chaperonin containing TCP1, subunit 2 (beta) | 1285.316 |
| GRB2 | growth factor receptor-bound protein 2 | 1266.871 |
| PSMB4 | proteasome (prosome, macropain) subunit, beta type, 4 | 1260.303 |
| LSM1 | LSM1 homolog, U6 small nuclear RNA associated (S. cerevisiae) | 1242.694 |
| SCYE1 | aminoacyl tRNA synthetase complex-interacting multifunctional protein 1 | 1242.46 |
| PTPN12 | protein tyrosine phosphatase, non-receptor type 12 | 1237.653 |
| KPNA2 | karyopherin alpha 2 (RAG cohort 1, importin alpha 1); karyopherin alpha-2 subunit like | 1236.271 |
| ACTR3 | ARP3 actin-related protein 3 homolog (yeast) | 1232.981 |
| LOC645139 | poly(A) binding protein interacting protein 1; similar to poly(A) binding protein interacting protein 1 | 1228.495 |
| HSP90AB1 | heat shock protein 90kDa alpha (cytosolic), class B member 1 | 1226.287 |
| PAPOLA | poly(A) polymerase alpha | 1224.83 |
| PSME3 | proteasome (prosome, macropain) activator subunit 3 (PA28 gamma; Ki) | 1222.407 |
| UBA1 | ubiquitin-like modifier activating enzyme 1 | 1219.419 |
| RNF103 | vacuolar protein sorting 24 homolog (S. cerevisiae); ring finger protein 103 | 1210.301 |
| DDOST | dolichyl-diphosphooligosaccharide-protein glycosyltransferase | 1203.563 |
| FAM89B | family with sequence similarity 89, member B | 1202.337 |
| LYPLA1 | lysophospholipase I | 1196.574 |
| PCMT1 | protein-L-isoaspartate (D-aspartate) O-methyltransferase | 1194.415 |
| TES | testis derived transcript (3 LIM domains) | 1179.874 |
| BRE | brain and reproductive organ-expressed (TNFRSF1A modulator) | 1172.738 |
| EIF3A | eukaryotic translation initiation factor 3, subunit A | 1171.517 |
| TSN | translin | 1168.778 |
| NONO | non-POU domain containing, octamer-binding | 1156.181 |
| ARF4 | ADP-ribosylation factor 4 | 1155.659 |
| ACTR2 | ARP2 actin-related protein 2 homolog (yeast) | 1155.277 |
| WBP11 | WW domain binding protein 11 | 1145.553 |
| MEMO1 | mediator of cell motility 1; similar to mediator of cell motility 1 | 1141.218 |
| GLRX | glutaredoxin (thioltransferase) | 1139.815 |
| Sept9 | septin 9 | 1136.102 |
| C19orf2 | chromosome 19 open reading frame 2 | 1125.152 |
| VPS26A | vacuolar protein sorting 26 homolog A (S. pombe) | 1123.507 |
| GARS | glycyl-tRNA synthetase | 1120.241 |
| BRE | brain and reproductive organ-expressed (TNFRSF1A modulator) | 1118.528 |
| PTS | 6-pyruvoyltetrahydropterin synthase | 1113.325 |
| NAP1L1 | nucleosome assembly protein 1-like 1 | 1107.323 |
| MAPRE1 | microtubule-associated protein, RP/EB family, member 1 | 1106.747 |
| CCNG2 | cyclin G2 | 1095.887 |
| SEC24B | SEC24 family, member B (S. cerevisiae) | 1093.195 |
| SQLE | squalene epoxidase | 1090.121 |
| PRMT1 | protein arginine methyltransferase 1 | 1088.615 |
| MARCKS | myristoylated alanine-rich protein kinase C substrate | 1084.987 |
| GAS6 | similar to growth arrest-specific 6; growth arrest-specific 6 | 1082.303 |
| HNRNPAB | heterogeneous nuclear ribonucleoprotein A/B | 1081.373 |
| Sept6 | septin 6 | 1074.064 |
| PSMB2 | proteasome (prosome, macropain) subunit, beta type, 2 | 1065.543 |
| CNOT7 | CCR4-NOT transcription complex, subunit 7 | 1061.299 |
| AK026926 | protein disulfide isomerase family A, member 6 | 1052.486 |
| KPNA2 | karyopherin alpha 2 (RAG cohort 1, importin alpha 1); karyopherin alpha-2 subunit like | 1049.691 |
| NCOA1 | nuclear receptor coactivator 1 | 1042.585 |
| PDIA6 | protein disulfide isomerase family A, member 6 | 1040.393 |
| BTBD1 | BTB (POZ) domain containing 1 | 1033.899 |
| FABP5 | fatty acid binding protein 5-like 2; fatty acid binding protein 5 (psoriasis-associated); fatty acid binding protein 5-like 8; fatty acid binding protein 5-like 7; fatty acid binding protein 5-like 9 | 1030.686 |
| MTF2 | metal response element binding transcription factor 2 | 1027.15 |
| FDFT1 | farnesyl-diphosphate farnesyltransferase 1 | 1021.052 |
| RPL28 | ribosomal protein L28 | 1020.957 |
| POLB | polymerase (DNA directed), beta | 1020.957 |
| WSB2 | WD repeat and SOCS box-containing 2 | 1019.473 |
| U19178 | brain and reproductive organ-expressed (TNFRSF1A modulator) | 1014.655 |
| SFPQ | splicing factor proline/glutamine-rich (polypyrimidine tract binding protein associated) | 1011.12 |
| YWHAE | similar to 14-3-3 protein epsilon (14-3-3E) (Mitochondrial import stimulation factor L subunit) (MSF L); tyrosine 3-monooxygenase/tryptophan 5-monooxygenase activation protein, epsilon polypeptide | 1011.085 |
| VKORC1 | vitamin K epoxide reductase complex, subunit 1 | 996.416 |
| CAMSAP1 | calmodulin regulated spectrin-associated protein 1 | 996.315 |
| AL562152 | SH3-domain binding protein 5 (BTK-associated) | 995.969 |
| NCOA1 | nuclear receptor coactivator 1 | 988.3606 |
| TMEM43 | transmembrane protein 43 | 983.101 |
| NAP1L1 | nucleosome assembly protein 1-like 1 | 979.9712 |
| MYST2 | MYST histone acetyltransferase 2; similar to MYST histone acetyltransferase 2 | 979.8738 |
| RHOQ | ras homolog gene family, member Q; similar to small GTP binding protein TC10 | 976.9925 |
| FOXJ3 | forkhead box J3 | 972.5254 |
| NCOA6 | nuclear receptor coactivator 6 | 972.0386 |
| MYO3B | high-mobility group box 1; high-mobility group box 1-like 10 | 968.081 |
| SMARCE1 | SWI/SNF related, matrix associated, actin dependent regulator of chromatin, subfamily e, member 1 | 967.5484 |
| MBD1 | methyl-CpG binding domain protein 1 | 961.5543 |
| U52111 | ribosomal protein L18a pseudogene 6; ribosomal protein L18a | 959.5079 |
| PSMD1 | proteasome (prosome, macropain) 26S subunit, non-ATPase, 1 | 959.1077 |
| PRKCB1 | protein kinase C, beta | 954.8183 |
| PRKRA | protein kinase, interferon-inducible double stranded RNA dependent activator | 954.0647 |
| EHD1 | EH-domain containing 1 | 952.5746 |
| KIAA1598 | KIAA1598 | 952.4092 |
| AL520908 | synaptotagmin binding, cytoplasmic RNA interacting protein | 946.8016 |
| AL157493 | DNA segment on chromosome 4 (unique) 234 expressed sequence | 945.1628 |
| CUL4B | cullin 4B | 939.7693 |
| UGP2 | UDP-glucose pyrophosphorylase 2 | 938.7985 |
| TES | testis derived transcript (3 LIM domains) | 936.6486 |
| SMAD2 | SMAD family member 2 | 936.4612 |
| LRRC47 | leucine rich repeat containing 47 | 917.1098 |
| N34842 | bromodomain containing 3 | 911.9559 |
| NEDD9 | neural precursor cell expressed, developmentally down-regulated 9 | 911.7709 |
| SFPQ | splicing factor proline/glutamine-rich (polypyrimidine tract binding protein associated) | 908.986 |
| RB1CC1 | RB1-inducible coiled-coil 1 | 906.936 |
| TRIM28 | tripartite motif-containing 28 | 902.8414 |
| PPP2R1A | protein phosphatase 2 (formerly 2A), regulatory subunit A, alpha isoform | 902.6226 |
| VPS24 | vacuolar protein sorting 24 homolog (S. cerevisiae); ring finger protein 103 | 902.2485 |
| MCL1 | myeloid cell leukemia sequence 1 (BCL2-related) | 901.0635 |
| RNF10 | ring finger protein 10 | 898.4643 |
| RAB9A | RAB9A, member RAS oncogene family | 896.9443 |
| NM_005758 | heterogeneous nuclear ribonucleoprotein A3 pseudogene 1 | 894.8933 |
| HNRNPD | heterogeneous nuclear ribonucleoprotein D (AU-rich element RNA binding protein 1, 37kDa) | 894.2578 |
| PAIP1 | poly(A) binding protein interacting protein 1; similar to poly(A) binding protein interacting protein 1 | 892.6746 |
| RAD23B | RAD23 homolog B (S. cerevisiae) | 891.0613 |
| DSTN | destrin (actin depolymerizing factor) | 883.3348 |
| DDAH2 | dimethylarginine dimethylaminohydrolase 2 | 877.4353 |
| GARNL4 | GTPase activating Rap/RanGAP domain-like 4 | 876.177 |
| ID2 | inhibitor of DNA binding 2, dominant negative helix-loop-helix protein | 873.6817 |
| REEP5 | receptor accessory protein 5 | 871.8281 |
| SMNDC1 | survival motor neuron domain containing 1 | 871.5332 |
| TRAPPC4 | trafficking protein particle complex 4 | 867.5539 |
| CAMSAP1 | calmodulin regulated spectrin-associated protein 1 | 859.2242 |
| MAX | MYC associated factor X | 856.0297 |
| VCL | vinculin | 853.2411 |
| VCAN | versican | 853.2326 |
| CPSF6 | cleavage and polyadenylation specific factor 6, 68kDa | 853.1845 |
| ACTR10 | actin-related protein 10 homolog (S. cerevisiae) | 852.7524 |
| AL579035 | EH-domain containing 1 | 852.4238 |
| PSMA5 | proteasome (prosome, macropain) subunit, alpha type, 5 | 851.9775 |
| PLXNC1 | plexin C1 | 846.2898 |
| NRG1 | neuregulin 1 | 844.1585 |
| PPIH | peptidylprolyl isomerase H (cyclophilin H) | 843.3856 |
| NKIRAS2 | NFKB inhibitor interacting Ras-like 2 | 835.9072 |
| TSC22D2 | TSC22 domain family, member 2 | 835.3667 |
| AGTPBP1 | ATP/GTP binding protein 1 | 833.7431 |
| AP1G1 | adaptor-related protein complex 1, gamma 1 subunit | 831.9607 |
| CHMP1A | chromatin modifying protein 1A | 829.7743 |
| CCNG2 | cyclin G2 | 829.608 |
| C1QBP | complement component 1, q subcomponent binding protein | 829.1772 |
| RBM15B | RNA binding motif protein 15B | 828.9075 |
| FYN | FYN oncogene related to SRC, FGR, YES | 825.4947 |
| JTV1 | aminoacyl tRNA synthetase complex-interacting multifunctional protein 2; stromal antigen 3-like 3 | 823.5545 |
| Sept6 | septin 6 | 822.87 |
| TRIAP1 | TP53 regulated inhibitor of apoptosis 1 | 821.1352 |
| NCOA1 | nuclear receptor coactivator 1 | 815.3719 |
| March5 | membrane-associated ring finger (C3HC4) 5 | 810.458 |
| KCTD5 | potassium channel tetramerisation domain containing 5 | 808.9076 |
| HMG2L1 | HMG box domain containing 4 | 807.2308 |
| EIF2C1 | eukaryotic translation initiation factor 2C, 1 | 805.7487 |
| AW151617 | SMAD family member 2 | 802.4178 |
| METAP2 | methionyl aminopeptidase 2 | 799.9843 |
| AMZ2 | archaelysin family metallopeptidase 2 | 794.8294 |
| VPS37B | vacuolar protein sorting 37 homolog B (S. cerevisiae) | 790.9242 |
| GNAI3 | guanine nucleotide binding protein (G protein), alpha inhibiting activity polypeptide 3 | 790.5834 |
| LOC729496 | SMT3 suppressor of mif two 3 homolog 4 (S. cerevisiae) | 790.1122 |
| CDC42EP4 | CDC42 effector protein (Rho GTPase binding) 4 | 789.6624 |
| MMD | monocyte to macrophage differentiation-associated | 789.0299 |
| JTV1 | aminoacyl tRNA synthetase complex-interacting multifunctional protein 2; stromal antigen 3-like 3 | 788.5352 |
| SLC9A6 | solute carrier family 9 (sodium/hydrogen exchanger), member 6 | 787.0889 |
| RAD23B | RAD23 homolog B (S. cerevisiae) | 786.4073 |
| CCNG2 | cyclin G2 | 783.1428 |
| C10orf56 | zinc finger, CCHC domain containing 24 | 782.944 |
| ELAVL1 | ELAV (embryonic lethal, abnormal vision, Drosophila)-like 1 (Hu antigen R) | 779.1595 |
| LPCAT1 | lysophosphatidylcholine acyltransferase 1 | 778.5419 |
| TSPAN14 | tetraspanin 14 | 776.0762 |
| MTCH2 | mitochondrial carrier homolog 2 (C. elegans) | 775.7394 |
| BZW2 | basic leucine zipper and W2 domains 2 | 774.4939 |
| Z97353 | ribosomal protein L15 pseudogene 22; ribosomal protein L15 pseudogene 18; ribosomal protein L15 pseudogene 17; ribosomal protein L15 pseudogene 3; ribosomal protein L15 pseudogene 7; ribosomal protein L15 | 772.442 |
| CCT3 | chaperonin containing TCP1, subunit 3 (gamma) | 772.364 |
| VEZF1 | vascular endothelial zinc finger 1 | 771.7542 |
| GRINL1A | glutamate receptor, ionotropic, N-methyl D-aspartate-like 1B; glutamate receptor, ionotropic, N-methyl D-aspartate-like 1A; GRINL1A complex locus | 769.9966 |
| RASGRP2 | RAS guanyl releasing protein 2 (calcium and DAG-regulated) | 768.8804 |
| MADD | MAP-kinase activating death domain | 768.1133 |
| LSM6 | LSM6 homolog, U6 small nuclear RNA associated (S. cerevisiae) | 766.7759 |
| CAMK2G | signal recognition particle 72kDa | 765.9601 |
| BEX4 | brain expressed, X-linked 4 | 761.6606 |
| MTSS1 | metastasis suppressor 1 | 759.8111 |
| IHPK2 | inositol hexakisphosphate kinase 2 | 752.2408 |
| DPH5 | DPH5 homolog (S. cerevisiae) | 752.2408 |
| NM_005825 | RAS guanyl releasing protein 2 (calcium and DAG-regulated) | 751.849 |
| ZC3H14 | zinc finger CCCH-type containing 14 | 745.9402 |
| NUP62 | nucleoporin 62kDa | 741.5857 |
| RAB8B | RAB8B, member RAS oncogene family | 737.8776 |
| NKIRAS2 | NFKB inhibitor interacting Ras-like 2 | 736.4346 |
| BCL11A | B-cell CLL/lymphoma 11A (zinc finger protein) | 734.1778 |
| ADNP | activity-dependent neuroprotector homeobox | 733.4071 |
| C10orf56 | zinc finger, CCHC domain containing 24 | 732.3256 |
| SMS | spermine synthase; similar to spermine synthase | 727.9075 |
| HERV-FRD | neural precursor cell expressed, developmentally down-regulated 9 | 713.9061 |
| HNRNPA3 | heterogeneous nuclear ribonucleoprotein A3 | 710.8502 |
| GALNT2 | UDP-N-acetyl-alpha-D-galactosamine:polypeptide N-acetylgalactosaminyltransferase 2 (GalNAc-T2) | 709.6782 |
| ZNHIT3 | zinc finger, HIT type 3 | 709.3902 |
| ANKHD1 | ankyrin repeat and KH domain containing 1; ANKHD1-EIF4EBP3 readthrough transcript; eukaryotic translation initiation factor 4E binding protein 3 | 708.0413 |
| CDC25B | cell division cycle 25 homolog B (S. pombe) | 704.6545 |
| CUGBP2 | CUG triplet repeat, RNA binding protein 2 | 703.4553 |
| RAB14 | RAB14, member RAS oncogene family | 703.0768 |
| CSNK1E | casein kinase 1, epsilon | 702.0438 |
| UGCG | UDP-glucose ceramide glucosyltransferase | 699.9589 |
| GRINL1A | glutamate receptor, ionotropic, N-methyl D-aspartate-like 1B; glutamate receptor, ionotropic, N-methyl D-aspartate-like 1A; GRINL1A complex locus | 698.5743 |
| GMPS | guanine monphosphate synthetase | 695.5272 |
| EDEM1 | ER degradation enhancer, mannosidase alpha-like 1 | 687.9177 |
| IPO5 | importin 5 | 682.7299 |
| MYC | v-myc myelocytomatosis viral oncogene homolog (avian) | 680.5384 |
| H1F0 | H1 histone family, member 0 | 672.239 |
| SOX4 | SRY (sex determining region Y)-box 4 | 670.1555 |
| SNN | stannin | 670.1171 |
| THAP11 | THAP domain containing 11 | 669.638 |
| ESD | esterase D/formylglutathione hydrolase | 669.4439 |
| NUDT4 | nudix (nucleoside diphosphate linked moiety X)-type motif 4; nudix (nucleoside diphosphate linked moiety X)-type motif 4 pseudogene 1 | 669.3079 |
| CCT6A | chaperonin containing TCP1, subunit 6A (zeta 1) | 667.6746 |
| X81636 | clathrin, light chain (Lca) | 665.6743 |
| CAPRIN1 | cell cycle associated protein 1 | 664.799 |
| DCUN1D1 | DCN1, defective in cullin neddylation 1, domain containing 1 (S. cerevisiae) | 663.3405 |
| HMG2L1 | HMG box domain containing 4 | 662.0522 |
| EPM2AIP1 | EPM2A (laforin) interacting protein 1 | 661.2412 |
| NUDT4 | nudix (nucleoside diphosphate linked moiety X)-type motif 4; nudix (nucleoside diphosphate linked moiety X)-type motif 4 pseudogene 1 | 659.224 |
| BRD3 | bromodomain containing 3 | 657.6555 |
| RPP30 | ribonuclease P/MRP 30kDa subunit | 657.1826 |
| ADAM9 | ADAM metallopeptidase domain 9 (meltrin gamma) | 651.4065 |
| NUCKS1 | nuclear casein kinase and cyclin-dependent kinase substrate 1 | 649.7182 |
| DDEF1 | ArfGAP with SH3 domain, ankyrin repeat and PH domain 1 | 644.819 |
| Sept6 | septin 6 | 643.5196 |
| TSN | translin | 642.6102 |
| CITED2 | Cbp/p300-interacting transactivator, with Glu/Asp-rich carboxy-terminal domain, 2 | 637.6089 |
| KLF7 | Kruppel-like factor 7 (ubiquitous) | 635.5126 |
| FXYD6 | FXYD domain containing ion transport regulator 6 | 633.9049 |
| PWP1 | PWP1 homolog (S. cerevisiae) | 633.7849 |
| LRRC42 | Yip1 domain family, member 1 | 630.1218 |
| IPO5 | importin 5 | 628.4774 |
| NOLC1 | nucleolar and coiled-body phosphoprotein 1 | 619.9152 |
| MAX | MYC associated factor X | 612.5352 |
| RAD17 | RAD17 homolog (S. pombe) | 610.9166 |
| NUDT4 | nudix (nucleoside diphosphate linked moiety X)-type motif 4; nudix (nucleoside diphosphate linked moiety X)-type motif 4 pseudogene 1 | 610.6527 |
| RAD21 | RAD21 homolog (S. pombe) | 609.7137 |
| SMARCE1 | SWI/SNF related, matrix associated, actin dependent regulator of chromatin, subfamily e, member 1 | 608.6113 |
| CAMSAP1 | calmodulin regulated spectrin-associated protein 1 | 608.4474 |
| TM2D1 | TM2 domain containing 1 | 595.3918 |
| MPZL1 | myelin protein zero-like 1 | 595.241 |
| PRKAR2B | protein kinase, cAMP-dependent, regulatory, type II, beta | 593.0621 |
| STX3 | syntaxin 3 | 591.629 |
| ACTR1A | ARP1 actin-related protein 1 homolog A, centractin alpha (yeast) | 590.536 |
| MBD1 | methyl-CpG binding domain protein 1 | 589.5077 |
| TRIM33 | tripartite motif-containing 33 | 588.2073 |
| UAP1 | UDP-N-acteylglucosamine pyrophosphorylase 1 | 587.3236 |
| KIAA0101 | KIAA0101 | 587.1028 |
| ZNF287 | zinc finger protein 287 | 584.7278 |
| DYNC1H1 | dynein, cytoplasmic 1, heavy chain 1 | 583.1778 |
| AI738802 | cell division cycle 2-like 6 (CDK8-like) | 581.5144 |
| RAB6A | RAB6B, member RAS oncogene family | 580.6366 |
| C9orf46 | chromosome 9 open reading frame 46 | 578.6367 |
| KIAA1033 | KIAA1033 | 577.1397 |
| LOC729148 | YDD19 protein | 572.8069 |
| CDK4 | cyclin-dependent kinase 4 | 572.7132 |
| PNO1 | partner of NOB1 homolog (S. cerevisiae) | 572.0213 |
| MGAT2 | mannosyl (alpha-1,6-)-glycoprotein beta-1,2-N-acetylglucosaminyltransferase | 571.4643 |
| HECW1 | HECT, C2 and WW domain containing E3 ubiquitin protein ligase 1 | 570.6743 |
| ACTR2 | ARP2 actin-related protein 2 homolog (yeast) | 568.7472 |
| PRPS1 | phosphoribosyl pyrophosphate synthetase 1; phosphoribosyl pyrophosphate synthetase 1-like 1 | 568.0387 |
| CCDC49 | coiled-coil domain containing 49 | 567.5846 |
| SSR1 | signal sequence receptor, alpha | 566.8251 |
| MTSS1 | metastasis suppressor 1 | 564.2412 |
| RAP1GDS1 | RAP1, GTP-GDP dissociation stimulator 1 | 564.1312 |
| AI672428 | nuclear receptor coactivator 1 | 558.2397 |
| TIMM23 | translocase of inner mitochondrial membrane 23 homolog (yeast); translocase of inner mitochondrial membrane 23 homolog B (yeast) | 556.154 |
| CITED2 | Cbp/p300-interacting transactivator, with Glu/Asp-rich carboxy-terminal domain, 2 | 554.7367 |
| GNS | glucosamine (N-acetyl)-6-sulfatase | 550.869 |
| UBE2S | ubiquitin-conjugating enzyme E2S | 549.0401 |
| GTF3C4 | general transcription factor IIIC, polypeptide 4, 90kDa | 543.0444 |
| UBE2K | ubiquitin-conjugating enzyme E2K (UBC1 homolog, yeast) | 540.5624 |
| MTF2 | metal response element binding transcription factor 2 | 540.4538 |
| DECR2 | 2,4-dienoyl CoA reductase 2, peroxisomal | 537.0314 |
| SRP72 | signal recognition particle 72kDa | 536.1745 |
| TRIM33 | tripartite motif-containing 33 | 534.7378 |
| ZBTB5 | zinc finger and BTB domain containing 5 | 534.1568 |
| IGF2R | insulin-like growth factor 2 receptor | 531.5024 |
| PLEKHB2 | pleckstrin homology domain containing, family B (evectins) member 2 | 531.2224 |
| C5orf13 | chromosome 5 open reading frame 13 | 521.944 |
| TUBB3 | tubulin, beta 3; melanocortin 1 receptor (alpha melanocyte stimulating hormone receptor) | 519.2697 |
| NUDT4 | nudix (nucleoside diphosphate linked moiety X)-type motif 4; nudix (nucleoside diphosphate linked moiety X)-type motif 4 pseudogene 1 | 518.0523 |
| NTAN1 | N-terminal asparagine amidase | 516.915 |
| PPM1B | protein phosphatase 1B (formerly 2C), magnesium-dependent, beta isoform | 516.8114 |
| ISG20L1 | apoptosis enhancing nuclease | 513.1812 |
| PREP | prolyl endopeptidase | 512.3409 |
| AGRN | agrin | 511.5879 |
| MCM2 | minichromosome maintenance complex component 2 | 511.5721 |
| POLR2I | polymerase (RNA) II (DNA directed) polypeptide I, 14.5kDa | 510.0325 |
| PPM1G | protein phosphatase 1G (formerly 2C), magnesium-dependent, gamma isoform | 509.7398 |
| ARCN1 | archain 1 | 507.7339 |
| PWP1 | PWP1 homolog (S. cerevisiae) | 505.9052 |
| SALL2 | sal-like 2 (Drosophila) | 505.1743 |
| PCGF3 | polycomb group ring finger 3 | 504.7812 |
| BF340123 | riboflavin kinase | 504.0912 |
| NDUFA7 | NADH dehydrogenase (ubiquinone) 1 alpha subcomplex, 7, 14.5kDa | 503.9885 |
| FN3KRP | fructosamine 3 kinase related protein | 502.2412 |
| C16orf33 | small nuclear ribonucleoprotein 25kDa (U11/U12) | 499.8236 |
| SDC2 | syndecan 2 | 497.0692 |
| PAIP1 | poly(A) binding protein interacting protein 1; similar to poly(A) binding protein interacting protein 1 | 496.8617 |
| MADD | MAP-kinase activating death domain | 494.7909 |
| APAF1 | apoptotic peptidase activating factor 1 | 492.0745 |
| FOXK2 | forkhead box K2 | 491.1743 |
| UBE2L3 | ubiquitin-conjugating enzyme E2L 3 | 488.5784 |
| ELAVL1 | ELAV (embryonic lethal, abnormal vision, Drosophila)-like 1 (Hu antigen R) | 488.5341 |
| MAX | MYC associated factor X | 488.5095 |
| RPL10 | ribosomal protein L10; ribosomal protein L10 pseudogene 15; ribosomal protein L10 pseudogene 6; ribosomal protein L10 pseudogene 16; ribosomal protein L10 pseudogene 9 | 486.8869 |
| AW006345 | signal sequence receptor, alpha | 486.6461 |
| PPP2R5C | protein phosphatase 2, regulatory subunit B', gamma isoform | 486.5002 |
| TSC22D1 | TSC22 domain family, member 1 | 482.163 |
| CLN8 | ceroid-lipofuscinosis, neuronal 8 (epilepsy, progressive with mental retardation) | 481.2339 |
| IVNS1ABP | influenza virus NS1A binding protein | 476.8965 |
| RAB22A | RAB22A, member RAS oncogene family | 475.4259 |
| SLC7A1 | solute carrier family 7 (cationic amino acid transporter, y+ system), member 1 | 474.0188 |
| HMGCR | 3-hydroxy-3-methylglutaryl-Coenzyme A reductase | 473.4569 |
| ACAT1 | acetyl-Coenzyme A acetyltransferase 1 | 472.1655 |
| ZDHHC6 | zinc finger, DHHC-type containing 6 | 472.043 |
| RNMT | RNA (guanine-7-) methyltransferase | 471.4209 |
| PDCD6 | aryl-hydrocarbon receptor repressor; programmed cell death 6 | 470.7987 |
| SYNCRIP | synaptotagmin binding, cytoplasmic RNA interacting protein | 470.6505 |
| UBE2D1 | ubiquitin-conjugating enzyme E2D 1 (UBC4/5 homolog, yeast) | 469.5105 |
| BG289914 | RCD1 required for cell differentiation1 homolog (S. pombe) | 468.641 |
| C5orf15 | chromosome 5 open reading frame 15 | 465.4014 |
| NGFRAP1 | nerve growth factor receptor (TNFRSF16) associated protein 1 | 463.8282 |
| DDX54 | DEAD (Asp-Glu-Ala-Asp) box polypeptide 54 | 463.2411 |
| HSPA4 | heat shock 70kDa protein 4 | 463.1365 |
| C1QBP | complement component 1, q subcomponent binding protein | 462.7069 |
| LARGE | like-glycosyltransferase | 460.9078 |
| HARS | histidyl-tRNA synthetase | 458.7018 |
| CDC42EP4 | CDC42 effector protein (Rho GTPase binding) 4 | 456.3857 |
| CBFB | core-binding factor, beta subunit | 456.368 |
| MYO5A | myosin VA (heavy chain 12, myoxin) | 456.3175 |
| CIB2 | calcium and integrin binding family member 2 | 454.6456 |
| PRAF2 | PRA1 domain family, member 2 | 453.4603 |
| BCAT1 | branched chain aminotransferase 1, cytosolic | 451.2426 |
| MAX | MYC associated factor X | 449.1109 |
| XPOT | exportin, tRNA (nuclear export receptor for tRNAs); similar to Exportin-T (tRNA exportin) (Exportin(tRNA)) | 448.7744 |
| RAD21 | RAD21 homolog (S. pombe) | 448.2109 |
| DIMT1L | DIM1 dimethyladenosine transferase 1-like (S. cerevisiae) | 446.945 |
| MPZL1 | myelin protein zero-like 1 | 445.1216 |
| POLR2H | polymerase (RNA) II (DNA directed) polypeptide H | 442.6883 |
| GSTM5 | glutathione S-transferase mu 5 | 441.8277 |
| Gcom1 | glutamate receptor, ionotropic, N-methyl D-aspartate-like 1B; glutamate receptor, ionotropic, N-methyl D-aspartate-like 1A; GRINL1A complex locus | 441.7496 |
| TNKS2 | tankyrase, TRF1-interacting ankyrin-related ADP-ribose polymerase 2 | 441.6744 |
| NM_014778 | nucleoporin like 1 | 440.0049 |
| SYNCRIP | synaptotagmin binding, cytoplasmic RNA interacting protein | 439.9828 |
| IFT20 | transmembrane protein 97 | 438.4078 |
| MTF2 | metal response element binding transcription factor 2 | 438.2092 |
| UTX | lysine (K)-specific demethylase 6A | 438.0429 |
| TMEM183A | transmembrane protein 183A; transmembrane protein 183B | 435.6817 |
| PUM2 | pumilio homolog 2 (Drosophila) | 434.9542 |
| PUM2 | pumilio homolog 2 (Drosophila) | 434.0999 |
| CD47 | CD47 molecule | 432.9113 |
| DYRK2 | dual-specificity tyrosine-(Y)-phosphorylation regulated kinase 2 | 432.6088 |
| NID1 | nidogen 1 | 431.7521 |
| SRM | spermidine synthase | 431.2574 |
| ZNF22 | zinc finger protein 22 (KOX 15) | 431.0769 |
| POGZ | pogo transposable element with ZNF domain | 430.2578 |
| SQLE | squalene epoxidase | 428.0786 |
| RUNX1T1 | runt-related transcription factor 1; translocated to, 1 (cyclin D-related) | 427.7103 |
| RPL18 | ribosomal protein L18 | 423.964 |
| NM_016402 | SUMO1 activating enzyme subunit 1 | 423.1909 |
| PSME3 | proteasome (prosome, macropain) activator subunit 3 (PA28 gamma; Ki) | 422.9843 |
| EPRS | glutamyl-prolyl-tRNA synthetase | 422.7324 |
| COPS5 | COP9 constitutive photomorphogenic homolog subunit 5 (Arabidopsis) | 422.4179 |
| IVNS1ABP | influenza virus NS1A binding protein | 420.3089 |
| GNAO1 | guanine nucleotide binding protein (G protein), alpha activating activity polypeptide O | 419.5078 |
| RAB1A | RAB1A, member RAS oncogene family | 417.0975 |
| ADD2 | adducin 2 (beta) | 416.5742 |
| RTN1 | reticulon 1 | 415.9159 |
| EEF1E1 | eukaryotic translation elongation factor 1 epsilon 1 | 415.5263 |
| RARS | arginyl-tRNA synthetase | 414.8411 |
| SCAMP1 | secretory carrier membrane protein 1 | 414.0313 |
| ZNF318 | zinc finger protein 318 | 412.5376 |
| SFPQ | splicing factor proline/glutamine-rich (polypyrimidine tract binding protein associated) | 411.0727 |
| KIF3C | kinesin family member 3C | 410.8412 |
| BE222709 | microfibrillar-associated protein 3 | 410.5387 |
| RALA | v-ral simian leukemia viral oncogene homolog A (ras related) | 410.3187 |
| CROP | cisplatin resistance-associated overexpressed protein | 410.2791 |
| TRAPPC4 | trafficking protein particle complex 4 | 409.4334 |
| VASH1 | vasohibin 1 | 407.5903 |
| C7orf43 | chromosome 7 open reading frame 43 | 406.5523 |
| CCT2 | chaperonin containing TCP1, subunit 2 (beta) | 406.2519 |
| WDR1 | WD repeat domain 1 | 405.5655 |
| N-PAC | cytokine-like nuclear factor n-pac | 405.1513 |
| PPM1B | protein phosphatase 1B (formerly 2C), magnesium-dependent, beta isoform | 404.437 |
| KIDINS220 | kinase D-interacting substrate, 220kDa | 403.229 |
| AW500473 | eukaryotic translation initiation factor 4E binding protein 2 | 403.2001 |
| CCT6A | chaperonin containing TCP1, subunit 6A (zeta 1) | 401.0182 |
| PRKACB | protein kinase, cAMP-dependent, catalytic, beta | 400.9084 |
| WDR41 | WD repeat domain 41 | 399.8111 |
| RAN | RAN, member RAS oncogene family | 399.2361 |
| PPP2R5C | protein phosphatase 2, regulatory subunit B', gamma isoform | 398.7528 |
| NM_017936 | SMEK homolog 1, suppressor of mek1 (Dictyostelium) | 397.3214 |
| ATRX | alpha thalassemia/mental retardation syndrome X-linked (RAD54 homolog, S. cerevisiae) | 395.6641 |
| ATXN10 | ataxin 10 | 395.2108 |
| H2AFY2 | H2A histone family, member Y2 | 394.5743 |
| EIF2S1 | eukaryotic translation initiation factor 2, subunit 1 alpha, 35kDa | 394.4856 |
| CKS1B | CDC28 protein kinase regulatory subunit 1B | 393.8337 |
| MYCL1 | v-myc myelocytomatosis viral oncogene homolog 1, lung carcinoma derived (avian) | 391.2352 |
| SMURF2 | SMAD specific E3 ubiquitin protein ligase 2 | 388.4486 |
| DIMT1L | DIM1 dimethyladenosine transferase 1-like (S. cerevisiae) | 386.8161 |
| PPP2R3A | protein phosphatase 2 (formerly 2A), regulatory subunit B'', alpha | 385.5746 |
| RAB11FIP3 | RAB11 family interacting protein 3 (class II) | 384.7743 |
| STX7 | syntaxin 7 | 384.3325 |
| ANKHD1 | ankyrin repeat and KH domain containing 1; ANKHD1-EIF4EBP3 readthrough transcript; eukaryotic translation initiation factor 4E binding protein 3 | 384.1198 |
| EML5 | zinc finger CCCH-type containing 14 | 383.3409 |
| PCDHB6 | protocadherin beta 6 | 383.0912 |
| KIAA1166 | zinc finger, C4H2 domain containing | 380.3783 |
| PRDX2 | peroxiredoxin 2 | 380.0953 |
| RAD54L2 | RAD54-like 2 (S. cerevisiae) | 379.7251 |
| CUGBP2 | CUG triplet repeat, RNA binding protein 2 | 378.2404 |
| NELL2 | NEL-like 2 (chicken) | 377.4155 |
| PYCR1 | pyrroline-5-carboxylate reductase 1 | 376.9686 |
| MOSPD2 | motile sperm domain containing 2 | 374.9361 |
| MDH2 | malate dehydrogenase 2, NAD (mitochondrial) | 374.2771 |
| PAFAH1B3 | platelet-activating factor acetylhydrolase, isoform Ib, subunit 3 (29kDa) | 370.9678 |
| SPTBN1 | spectrin, beta, non-erythrocytic 1 | 370.9678 |
| ZBED4 | zinc finger, BED-type containing 4 | 370.8968 |
| NTAN1 | N-terminal asparagine amidase | 369.23 |
| NT5DC1 | TSPY-like 4 | 368.9986 |
| CDC2L6 | cell division cycle 2-like 6 (CDK8-like) | 365.7621 |
| GLRA2 | glycine receptor, alpha 2 | 363.5307 |
| AL574194 | lymphatic vessel endothelial hyaluronan receptor 1 | 363.0133 |
| PLAA | phospholipase A2-activating protein | 359.6234 |
| NASP | nuclear autoantigenic sperm protein (histone-binding) | 358.9228 |
| MARCKS | myristoylated alanine-rich protein kinase C substrate | 358.3856 |
| AU147942 | peroxiredoxin 2 | 358.2182 |
| RBM4B | RNA binding motif protein 4B | 357.241 |
| BEX1 | brain expressed, X-linked 1 | 356.3708 |
| CCDC47 | coiled-coil domain containing 47 | 354.9697 |
| TIMM23 | translocase of inner mitochondrial membrane 23 homolog (yeast); translocase of inner mitochondrial membrane 23 homolog B (yeast) | 353.0508 |
| AV681579 | amyloid beta precursor protein (cytoplasmic tail) binding protein 2 | 352.4693 |
| OSBPL11 | oxysterol binding protein-like 11 | 350.8355 |
| HDAC2 | histone deacetylase 2 | 350.0134 |
| KHDRBS1 | KH domain containing, RNA binding, signal transduction associated 1 | 348.6377 |
| RFC5 | WD repeat and SOCS box-containing 2 | 346.2098 |
| PIGO | phosphatidylinositol glycan anchor biosynthesis, class O | 345.6039 |
| GNAI3 | guanine nucleotide binding protein (G protein), alpha inhibiting activity polypeptide 3 | 345.0792 |
| IPO5 | importin 5 | 344.8333 |
| KLF6 | Kruppel-like factor 6 | 342.9289 |
| TLK1 | tousled-like kinase 1 | 342.8935 |
| STAG1 | stromal antigen 1 | 341.6539 |
| NOLA2 | required for meiotic nuclear division 5 homolog B (S. cerevisiae) | 340.3412 |
| SQSTM1 | sequestosome 1 | 339.6666 |
| FBXL14 | F-box and leucine-rich repeat protein 14 | 339.5482 |
| ABCE1 | similar to ATP-binding cassette, sub-family E, member 1; ATP-binding cassette, sub-family E (OABP), member 1 | 339.4828 |
| RAP1GDS1 | RAP1, GTP-GDP dissociation stimulator 1 | 339.469 |
| SOCS5 | suppressor of cytokine signaling 5 | 339.2517 |
| KLHL20 | kelch-like 20 (Drosophila) | 338.0393 |
| ANKRD12 | ankyrin repeat domain 12 | 337.5827 |
| INA | internexin neuronal intermediate filament protein, alpha | 336.7206 |
| FBXW2 | F-box and WD repeat domain containing 2 | 335.7908 |
| HMGB3 | similar to high mobility group box 3; high-mobility group box 3 | 333.4668 |
| TIA1 | TIA1 cytotoxic granule-associated RNA binding protein | 332.4077 |
| DIP2A | DIP2 disco-interacting protein 2 homolog A (Drosophila) | 332.1136 |
| ACO1 | aconitase 1, soluble | 331.386 |
| RIOK2 | RIO kinase 2 (yeast) | 330.0077 |
| U35622 | ets variant 4 | 329.6301 |
| SRR | serine racemase | 329.4806 |
| PRPS2 | phosphoribosyl pyrophosphate synthetase 2 | 329.3658 |
| UBE2I | ubiquitin-conjugating enzyme E2I (UBC9 homolog, yeast) | 328.5475 |
| MPHOSPH10 | M-phase phosphoprotein 10 (U3 small nucleolar ribonucleoprotein) | 328.2772 |
| MPZL1 | myelin protein zero-like 1 | 327.3913 |
| NASP | nuclear autoantigenic sperm protein (histone-binding) | 327.2411 |
| ZNF821 | zinc finger protein 821 | 326.7788 |
| PSMD1 | proteasome (prosome, macropain) 26S subunit, non-ATPase, 1 | 325.9406 |
| L1CAM | L1 cell adhesion molecule | 324.4077 |
| CFDP1 | craniofacial development protein 1 | 321.2115 |
| ID3 | inhibitor of DNA binding 3, dominant negative helix-loop-helix protein | 320.7574 |
| TRIM33 | tripartite motif-containing 33 | 319.7458 |
| FBXO21 | F-box protein 21 | 318.9277 |
| RNMT | RNA (guanine-7-) methyltransferase | 318.2205 |
| FEM1C | fem-1 homolog c (C. elegans) | 317.7481 |
| TBC1D9 | TBC1 domain family, member 9 (with GRAM domain) | 316.4847 |
| AA527502 | heterogeneous nuclear ribonucleoprotein A3 | 316.2084 |
| CRMP1 | collapsin response mediator protein 1 | 314.8412 |
| JUN | jun oncogene | 314.0496 |
| NUP205 | nucleoporin 205kDa | 314.0298 |
| PTRH2 | peptidyl-tRNA hydrolase 2 | 312.5743 |
| KIAA0649 | KIAA0649 | 312.206 |
| FBXO21 | F-box protein 21 | 311.1333 |
| TSN | translin | 310.6915 |
| PAIP1 | poly(A) binding protein interacting protein 1; similar to poly(A) binding protein interacting protein 1 | 310.4507 |
| PAPOLA | poly(A) polymerase alpha | 309.855 |
| UBA3 | ubiquitin-like modifier activating enzyme 3 | 308.3133 |
| FARP1 | FERM, RhoGEF (ARHGEF) and pleckstrin domain protein 1 (chondrocyte-derived) | 307.3411 |
| PEX13 | peroxisomal biogenesis factor 13 | 305.9565 |
| CDCA8 | cell division cycle associated 8 | 305.5768 |
| EFR3A | EFR3 homolog A (S. cerevisiae) | 304.2338 |
| GPR56 | G protein-coupled receptor 56 | 302.8411 |
| TIA1 | TIA1 cytotoxic granule-associated RNA binding protein | 302.2503 |
| BF589679 | aminoacyl tRNA synthetase complex-interacting multifunctional protein 1 | 301.9661 |
| TMEM97 | transmembrane protein 97 | 300.3397 |
| M19267 | tropomyosin 1 (alpha) | 300.2394 |
| PELO | pelota homolog (Drosophila) | 299.751 |
| PHTF2 | putative homeodomain transcription factor 2 | 298.6635 |
| COQ2 | coenzyme Q2 homolog, prenyltransferase (yeast) | 298.5744 |
| RRS1 | RRS1 ribosome biogenesis regulator homolog (S. cerevisiae) | 297.7129 |
| AB006572 | chromosome 19 open reading frame 2 | 297.6492 |
| ARL4C | ADP-ribosylation factor-like 4C | 296.7412 |
| CBL | Cas-Br-M (murine) ecotropic retroviral transforming sequence | 296.3584 |
| TOPBP1 | topoisomerase (DNA) II binding protein 1 | 295.2259 |
| TBC1D9 | TBC1 domain family, member 9 (with GRAM domain) | 295.0098 |
| BE968833 | spectrin, beta, non-erythrocytic 1 | 294.8932 |
| PSMC6 | proteasome (prosome, macropain) 26S subunit, ATPase, 6 | 294.888 |
| CUL2 | cullin 2 | 294.7434 |
| ZNF292 | zinc finger protein 292 | 293.2561 |
| TGS1 | trimethylguanosine synthase homolog (S. cerevisiae) | 292.5361 |
| VEZF1 | vascular endothelial zinc finger 1 | 292.5164 |
| PSME3 | proteasome (prosome, macropain) activator subunit 3 (PA28 gamma; Ki) | 290.8555 |
| BE048514 | syndecan 2 | 290.7412 |
| PPP2R5C | protein phosphatase 2, regulatory subunit B', gamma isoform | 290.7408 |
| CUL4B | cullin 4B | 290.2052 |
| C5orf13 | chromosome 5 open reading frame 13 | 288.9231 |
| REEP1 | receptor accessory protein 1 | 288.3734 |
| TDG | similar to G/T mismatch-specific thymine DNA glycosylase; thymine-DNA glycosylase | 288.3239 |
| NID1 | nidogen 1 | 288.1518 |
| ABCE1 | similar to ATP-binding cassette, sub-family E, member 1; ATP-binding cassette, sub-family E (OABP), member 1 | 288.1387 |
| SERPINH1 | serpin peptidase inhibitor, clade H (heat shock protein 47), member 1, (collagen binding protein 1) | 288.0078 |
| AKAP12 | A kinase (PRKA) anchor protein 12 | 286.8062 |
| MOGAT2 | monoacylglycerol O-acyltransferase 2 | 284.7174 |
| SEC63 | SEC63 homolog (S. cerevisiae) | 284.3117 |
| SRP72 | signal recognition particle 72kDa | 283.884 |
| DYNC1I1 | dynein, cytoplasmic 1, intermediate chain 1 | 283.6585 |
| AW080932 | heterogeneous nuclear ribonucleoprotein A3 | 283.353 |
| NUP37 | nucleoporin 37kDa | 283.2476 |
| RPL10 | ribosomal protein L10; ribosomal protein L10 pseudogene 15; ribosomal protein L10 pseudogene 6; ribosomal protein L10 pseudogene 16; ribosomal protein L10 pseudogene 9 | 281.8512 |
| ZNF821 | zinc finger protein 821 | 281.2435 |
| WDR57 | small nuclear ribonucleoprotein 40kDa (U5) | 280.6448 |
| TM2D1 | TM2 domain containing 1 | 279.9849 |
| ANKHD1 | ankyrin repeat and KH domain containing 1; ANKHD1-EIF4EBP3 readthrough transcript; eukaryotic translation initiation factor 4E binding protein 3 | 278.8353 |
| FAM98A | family with sequence similarity 98, member A | 278.8286 |
| NUP85 | nucleoporin 85kDa | 278.8076 |
| SOX4 | SRY (sex determining region Y)-box 4 | 278.6518 |
| LANCL2 | LanC lantibiotic synthetase component C-like 2 (bacterial) | 276.8376 |
| NUP155 | nucleoporin 155kDa | 275.7333 |
| CHORDC1 | cysteine and histidine-rich domain (CHORD)-containing 1; cysteine and histidine-rich domain (CHORD)-containing 1 pseudogene | 275.7319 |
| AI123320 | eukaryotic translation initiation factor 3, subunit A | 274.3948 |
| MPPED1 | metallophosphoesterase domain containing 1 | 274.3542 |
| TMEM97 | transmembrane protein 97 | 273.9614 |
| NUSAP1 | nucleolar and spindle associated protein 1 | 272.2285 |
| DHCR7 | 7-dehydrocholesterol reductase | 271.2544 |
| AA811923 | La ribonucleoprotein domain family, member 4B | 270.4375 |
| GTDC1 | glycosyltransferase-like domain containing 1 | 268.441 |
| STAT1 | signal transducer and activator of transcription 1, 91kDa | 267.3749 |
| RTN1 | reticulon 1 | 267.2639 |
| MAP3K7 | mitogen-activated protein kinase kinase kinase 7 | 267.0708 |
| SCFD1 | sec1 family domain containing 1 | 266.9097 |
| TRIB2 | tribbles homolog 2 (Drosophila) | 266.841 |
| ZNF318 | zinc finger protein 318 | 266.3499 |
| RAB11A | RAB11A, member RAS oncogene family | 266.1048 |
| MAOA | monoamine oxidase A | 266.0744 |
| ZNF609 | zinc finger protein 609 | 265.2411 |
| CPSF6 | cleavage and polyadenylation specific factor 6, 68kDa | 265.1156 |
| SEC23A | Sec23 homolog A (S. cerevisiae) | 264.1432 |
| PCMT1 | protein-L-isoaspartate (D-aspartate) O-methyltransferase | 264.1064 |
| Sept11 | septin 11 | 263.4547 |
| RASA1 | RAS p21 protein activator (GTPase activating protein) 1 | 263.3272 |
| SLC7A1 | solute carrier family 7 (cationic amino acid transporter, y+ system), member 1 | 263.1743 |
| GRWD1 | glutamate-rich WD repeat containing 1 | 263.1743 |
| NRG1 | neuregulin 1 | 262.8547 |
| AGRN | agrin | 261.3069 |
| CHRNA7 | CHRNA7 (cholinergic receptor, nicotinic, alpha 7, exons 5-10) and FAM7A (family with sequence similarity 7A, exons A-E) fusion; cholinergic receptor, nicotinic, alpha 7 | 261.2713 |
| AGRN | agrin | 260.9009 |
| SEMA6A | sema domain, transmembrane domain (TM), and cytoplasmic domain, (semaphorin) 6A | 259.1744 |
| IDH1 | isocitrate dehydrogenase 1 (NADP+), soluble | 258.7879 |
| MGAT2 | mannosyl (alpha-1,6-)-glycoprotein beta-1,2-N-acetylglucosaminyltransferase | 258.4498 |
| MAP3K7 | mitogen-activated protein kinase kinase kinase 7 | 258.433 |
| FNDC3B | fibronectin type III domain containing 3B | 258.1195 |
| EIF2S1 | eukaryotic translation initiation factor 2, subunit 1 alpha, 35kDa | 257.0442 |
| SYNCRIP | synaptotagmin binding, cytoplasmic RNA interacting protein | 256.191 |
| RLBP1 | retinaldehyde binding protein 1 | 256.0076 |
| HS3ST2 | heparan sulfate (glucosamine) 3-O-sulfotransferase 2 | 255.8376 |
| RMND5B | required for meiotic nuclear division 5 homolog B (S. cerevisiae) | 255.5414 |
| C6orf134 | chromosome 6 open reading frame 134 | 255.1933 |
| SPTBN1 | spectrin, beta, non-erythrocytic 1 | 254.4023 |
| CASP3 | caspase 3, apoptosis-related cysteine peptidase | 253.4197 |
| NPY | neuropeptide Y | 253.3842 |
| PAICS | phosphoribosylaminoimidazole carboxylase, phosphoribosylaminoimidazole succinocarboxamide synthetase | 252.9711 |
| Z95126 | SET nuclear oncogene; similar to SET translocation | 252.3208 |
| WDR1 | WD repeat domain 1 | 252.2411 |
| TRIB2 | tribbles homolog 2 (Drosophila) | 252.1922 |
| SCYL2 | SCY1-like 2 (S. cerevisiae) | 250.9387 |
| RPRM | reprimo, TP53 dependent G2 arrest mediator candidate | 249.8543 |
| MPZL1 | myelin protein zero-like 1 | 249.2411 |
| LARP5 | La ribonucleoprotein domain family, member 4B | 248.5078 |
| NCALD | neurocalcin delta | 248.417 |
| TSC22D2 | TSC22 domain family, member 2 | 247.4078 |
| WHSC1 | Wolf-Hirschhorn syndrome candidate 1 | 247.3802 |
| MPZL1 | myelin protein zero-like 1 | 247.0947 |
| RAD17 | RAD17 homolog (S. pombe) | 245.931 |
| PCDHA3 | protocadherin alpha 3 | 245.5745 |
| SMC1A | structural maintenance of chromosomes 1A | 245.5105 |
| NSMCE4A | non-SMC element 4 homolog A (S. cerevisiae) | 245.4224 |
| CIB2 | calcium and integrin binding family member 2 | 245.3411 |
| PPP2R3A | protein phosphatase 2 (formerly 2A), regulatory subunit B'', alpha | 244.6463 |
| IFT57 | intraflagellar transport 57 homolog (Chlamydomonas) | 244.5926 |
| RNGTT | RNA guanylyltransferase and 5'-phosphatase | 244.1087 |
| RASGRP2 | RAS guanyl releasing protein 2 (calcium and DAG-regulated) | 243.1561 |
| WHSC1 | Wolf-Hirschhorn syndrome candidate 1 | 241.2695 |
| BG292065 | protein kinase N2 | 241.1515 |
| MRPS2 | mitochondrial ribosomal protein S2 | 240.9668 |
| SDC2 | syndecan 2 | 240.1807 |
| EFR3A | EFR3 homolog A (S. cerevisiae) | 239.6792 |
| CROP | cisplatin resistance-associated overexpressed protein | 239.6758 |
| TP53 | tumor protein p53 | 239.0309 |
| SFRS10 | transformer 2 beta homolog (Drosophila) | 238.5919 |
| AKAP7 | A kinase (PRKA) anchor protein 7 | 238.1859 |
| AKAP7 | A kinase (PRKA) anchor protein 7 | 237.7602 |
| LARP5 | La ribonucleoprotein domain family, member 4B | 237.2412 |
| PCGF3 | polycomb group ring finger 3 | 236.9156 |
| AW003989 | chromosome 19 open reading frame 2 | 236.6468 |
| PPP2R2B | protein phosphatase 2 (formerly 2A), regulatory subunit B, beta isoform | 236.5936 |
| KLHL20 | kelch-like 20 (Drosophila) | 235.8906 |
| MYO16 | myosin XVI | 235.5744 |
| NDN | necdin homolog (mouse) | 235.53 |
| X81636 | clathrin, light chain (Lca) | 234.4081 |
| PRPS1 | phosphoribosyl pyrophosphate synthetase 1; phosphoribosyl pyrophosphate synthetase 1-like 1 | 233.2314 |
| ATP2C1 | ATPase, Ca++ transporting, type 2C, member 1 | 232.9698 |
| UBE2L3 | ubiquitin-conjugating enzyme E2L 3 | 232.7411 |
| N-PAC | cytokine-like nuclear factor n-pac | 232.3047 |
| CRIP2 | cysteine-rich protein 2 | 232.209 |
| BMP5 | bone morphogenetic protein 5 | 231.338 |
| MAP3K7 | mitogen-activated protein kinase kinase kinase 7 | 231.2054 |
| POU3F1 | POU class 3 homeobox 1 | 230.6023 |
| KIAA1648 | chromosome 6 open reading frame 35; hCG1820764; tetratricopeptide repeat domain 28 | 230.5745 |
| UBE2V2 | ubiquitin-conjugating enzyme E2 variant 2 | 230.2402 |
| PDGFRA | platelet-derived growth factor receptor, alpha polypeptide | 230.2249 |
| RAB11FIP3 | RAB11 family interacting protein 3 (class II) | 229.1657 |
| NRG1 | neuregulin 1 | 229.0366 |
| ZNF354A | zinc finger protein 354A | 228.9559 |
| GMDS | GDP-mannose 4,6-dehydratase | 228.0072 |
| AGTPBP1 | ATP/GTP binding protein 1 | 227.1738 |
| PKN2 | protein kinase N2 | 226.3788 |
| MTF2 | metal response element binding transcription factor 2 | 226.2731 |
| CCDC90B | coiled-coil domain containing 90B | 225.5572 |
| NSMCE4A | non-SMC element 4 homolog A (S. cerevisiae) | 224.9079 |
| STAG1 | stromal antigen 1 | 224.6414 |
| KLHL25 | kelch-like 25 (Drosophila) | 224.6317 |
| BCL11A | B-cell CLL/lymphoma 11A (zinc finger protein) | 224.3463 |
| RASGRP2 | RAS guanyl releasing protein 2 (calcium and DAG-regulated) | 223.7522 |
| CASP2 | caspase 2, apoptosis-related cysteine peptidase | 223.3283 |
| UBE2I | ubiquitin-conjugating enzyme E2I (UBC9 homolog, yeast) | 223.1277 |
| Sept6 | septin 6 | 222.9295 |
| NM_014459 | protocadherin 17 | 222.6746 |
| HMGCR | 3-hydroxy-3-methylglutaryl-Coenzyme A reductase | 222.1025 |
| NUCKS1 | nuclear casein kinase and cyclin-dependent kinase substrate 1 | 222.0118 |
| MCM6 | minichromosome maintenance complex component 6 | 219.5551 |
| ZNF32 | zinc finger protein 32 | 219.4391 |
| RB1CC1 | RB1-inducible coiled-coil 1 | 217.8306 |
| PIK3CD | phosphoinositide-3-kinase, catalytic, delta polypeptide | 217.6182 |
| NEUROD6 | neurogenic differentiation 6 | 217.5747 |
| PFN2 | profilin 2 | 217.3224 |
| D4S234E | DNA segment on chromosome 4 (unique) 234 expressed sequence | 216.1031 |
| TMEM132A | solute carrier family 25, member 30 | 215.8136 |
| ATP2C1 | ATPase, Ca++ transporting, type 2C, member 1 | 215.7151 |
| ATP2C1 | ATPase, Ca++ transporting, type 2C, member 1 | 215.4316 |
| ZNF688 | zinc finger protein 688; zinc finger protein 785 | 215.0979 |
| GTDC1 | glycosyltransferase-like domain containing 1 | 214.423 |
| SCAPER | S-phase cyclin A-associated protein in the ER | 213.7416 |
| MOSPD1 | motile sperm domain containing 1 | 213.2476 |
| SMC6 | structural maintenance of chromosomes 6 | 212.6416 |
| ZNF609 | zinc finger protein 609 | 211.575 |
| NOLC1 | nucleolar and coiled-body phosphoprotein 1 | 210.9417 |
| DBC1 | deleted in bladder cancer 1 | 210.0718 |
| CUL4B | cullin 4B | 210.006 |
| UBE2I | ubiquitin-conjugating enzyme E2I (UBC9 homolog, yeast) | 209.8522 |
| RNGTT | RNA guanylyltransferase and 5'-phosphatase | 209.8419 |
| FAM155A | family with sequence similarity 155, member A | 209.7052 |
| PAFAH1B2 | platelet-activating factor acetylhydrolase, isoform Ib, subunit 2 (30kDa) | 209.575 |
| UBE2K | ubiquitin-conjugating enzyme E2K (UBC1 homolog, yeast) | 209.3814 |
| AGRN | agrin | 207.8339 |
| BE793789 | Wolf-Hirschhorn syndrome candidate 1 | 207.1608 |
| SMU1 | smu-1 suppressor of mec-8 and unc-52 homolog (C. elegans) | 206.4438 |
| RNF111 | ring finger protein 111 | 206.3229 |
| TM2D1 | TM2 domain containing 1 | 206.0554 |
| TNRC6B | trinucleotide repeat containing 6B | 205.5993 |
| AI216690 | dual-specificity tyrosine-(Y)-phosphorylation regulated kinase 2 | 204.7843 |
| PIGO | phosphatidylinositol glycan anchor biosynthesis, class O | 204.1658 |
| YIPF4 | Yip1 domain family, member 4 | 203.7422 |
| NARS2 | asparaginyl-tRNA synthetase 2, mitochondrial (putative) | 203.5516 |
| TXN | thioredoxin | 203.5062 |
| PWP1 | PWP1 homolog (S. cerevisiae) | 202.7076 |
| PDAP1 | PDGFA associated protein 1; similar to PDGFA associated protein 1 | 201.8655 |
| HDGFRP3 | hepatoma-derived growth factor, related protein 3 | 201.4301 |
| MTSS1 | metastasis suppressor 1 | 201.1539 |
| CCNB2 | cyclin B2 | 201.1538 |
| KHDRBS2 | KH domain containing, RNA binding, signal transduction associated 2 | 200.6624 |
| CLIP2 | CAP-GLY domain containing linker protein 2 | 200.4093 |
| SRGAP2 | SLIT-ROBO Rho GTPase activating protein 2 | 200.0615 |
| AI273812 | v-myc myelocytomatosis viral oncogene homolog 1, lung carcinoma derived (avian) | 199.9577 |
| SEMA6D | sema domain, transmembrane domain (TM), and cytoplasmic domain, (semaphorin) 6D | 199.5203 |
| Sept9 | septin 9 | 199.3167 |
| D4S234E | DNA segment on chromosome 4 (unique) 234 expressed sequence | 198.7984 |
| ZNF654 | zinc finger protein 654 | 198.5086 |
| TUBB2A | tubulin, beta 2B | 197.963 |
| FAM118A | family with sequence similarity 118, member A | 197.263 |
| NM_001224 | caspase 2, apoptosis-related cysteine peptidase | 196.6876 |
| GTF2E1 | general transcription factor IIE, polypeptide 1, alpha 56kDa | 196.4654 |
| NRG1 | neuregulin 1 | 196.2019 |
| TSPAN6 | tetraspanin 6 | 196.1652 |
| JUN | jun oncogene | 196.0641 |
| OXSM | 3-oxoacyl-ACP synthase, mitochondrial | 195.8433 |
| SPTBN1 | spectrin, beta, non-erythrocytic 1 | 195.6767 |
| ADD2 | adducin 2 (beta) | 195.4599 |
| ZSWIM1 | zinc finger, SWIM-type containing 1 | 195.39 |
| SAMD14 | sterile alpha motif domain containing 14 | 194.3279 |
| IGSF3 | immunoglobulin superfamily, member 3 | 194.1036 |
| ZBED4 | zinc finger, BED-type containing 4 | 193.9854 |
| NM_018627 | calmodulin regulated spectrin-associated protein 1 | 193.8196 |
| TSC22D2 | TSC22 domain family, member 2 | 193.6579 |
| HMP19 | HMP19 protein | 193.1053 |
| MOSPD2 | motile sperm domain containing 2 | 192.9375 |
| RAB22A | RAB22A, member RAS oncogene family | 192.7063 |
| CTTNBP2NL | CTTNBP2 N-terminal like | 192.5982 |
| POLR3H | cold shock domain containing C2, RNA binding | 192.3473 |
| NFU1 | NFU1 iron-sulfur cluster scaffold homolog (S. cerevisiae) | 192.3149 |
| NRG1 | neuregulin 1 | 191.471 |
| TCF4 | transcription factor 4 | 191.4088 |
| RQCD1 | RCD1 required for cell differentiation1 homolog (S. pombe) | 191.3564 |
| PAX6 | paired box 6 | 191.1778 |
| KIF2A | kinesin heavy chain member 2A | 190.2448 |
| CDH2 | cadherin 2, type 1, N-cadherin (neuronal) | 190.2448 |
| MYCN | v-myc myelocytomatosis viral related oncogene, neuroblastoma derived (avian) | 190.1778 |
| UGDH | UDP-glucose dehydrogenase | 190.0765 |
| RQCD1 | RCD1 required for cell differentiation1 homolog (S. pombe) | 189.9596 |
| KIF3C | kinesin family member 3C | 189.5069 |
| APC2 | adenomatosis polyposis coli 2 | 188.9096 |
| PHKA1 | phosphorylase kinase, alpha 1 pseudogene 1; phosphorylase kinase, alpha 1 (muscle) | 188.5787 |
| DPYSL3 | dihydropyrimidinase-like 3 | 188.512 |
| CASP2 | caspase 2, apoptosis-related cysteine peptidase | 186.746 |
| SOCS5 | suppressor of cytokine signaling 5 | 186.2392 |
| N-PAC | cytokine-like nuclear factor n-pac | 184.9351 |
| RAMP2 | receptor (G protein-coupled) activity modifying protein 2 | 184.6492 |
| CDH11 | cadherin 11, type 2, OB-cadherin (osteoblast) | 184.2471 |
| PLXNA3 | plexin A3 | 184.2465 |
| HDGFRP3 | hepatoma-derived growth factor, related protein 3 | 183.6573 |
| NM_017936 | SMEK homolog 1, suppressor of mek1 (Dictyostelium) | 183.2341 |
| DZIP3 | DAZ interacting protein 3, zinc finger | 182.8501 |
| SEC23A | Sec23 homolog A (S. cerevisiae) | 182.7677 |
| PBRM1 | polybromo 1 | 182.4147 |
| LMNB1 | lamin B1 | 180.9158 |
| HIVEP2 | human immunodeficiency virus type I enhancer binding protein 2 | 180.9158 |
| KIDINS220 | kinase D-interacting substrate, 220kDa | 180.7284 |
| CDC2 | cell division cycle 2, G1 to S and G2 to M | 180.6178 |
| GALK1 | galactokinase 1 | 180.5467 |
| ARMC1 | armadillo repeat containing 1 | 179.2239 |
| SDC3 | syndecan 3 | 179.0466 |
| PFTK1 | PFTAIRE protein kinase 1 | 178.9724 |
| PSME3 | proteasome (prosome, macropain) activator subunit 3 (PA28 gamma; Ki) | 177.9304 |
| HSPA4 | heat shock 70kDa protein 4 | 177.4194 |
| NM_013303 | angiogenic factor with G patch and FHA domains 1 | 177.4184 |
| MLLT11 | myeloid/lymphoid or mixed-lineage leukemia (trithorax homolog, Drosophila); translocated to, 11 | 176.7617 |
| HDGFRP3 | hepatoma-derived growth factor, related protein 3 | 176.633 |
| PSPC1 | paraspeckle component 1; paraspeckle protein 1 pseudogene | 176.4604 |
| EXOC2 | exocyst complex component 2 | 176.2863 |
| STMN1 | stathmin 1 | 176.042 |
| DBR1 | debranching enzyme homolog 1 (S. cerevisiae) | 175.2425 |
| NOLC1 | nucleolar and coiled-body phosphoprotein 1 | 175.2095 |
| C11orf24 | chromosome 11 open reading frame 24 | 174.9413 |
| IGF2R | insulin-like growth factor 2 receptor | 174.7673 |
| GABRA5 | gamma-aminobutyric acid (GABA) A receptor, alpha 5 | 174.4841 |
| TPBG | trophoblast glycoprotein | 174.3958 |
| AK002179 | heparan sulfate 2-O-sulfotransferase 1 | 174.1905 |
| FRS2 | fibroblast growth factor receptor substrate 2 | 174.1743 |
| KIAA0644 | KIAA0644 gene product | 174.1122 |
| RPS6KA6 | ribosomal protein S6 kinase, 90kDa, polypeptide 6 | 174.0305 |
| SQSTM1 | sequestosome 1 | 173.4648 |
| FBXW2 | F-box and WD repeat domain containing 2 | 173.4342 |
| JTV1 | aminoacyl tRNA synthetase complex-interacting multifunctional protein 2; stromal antigen 3-like 3 | 173.3782 |
| ANKRD57 | ankyrin repeat domain 57 | 172.5784 |
| BCL11A | B-cell CLL/lymphoma 11A (zinc finger protein) | 171.6403 |
| C10orf97 | chromosome 10 open reading frame 97 | 171.5617 |
| TCF4 | transcription factor 4 | 170.2586 |
| EHBP1 | EH domain binding protein 1 | 169.5458 |
| COQ3 | coenzyme Q3 homolog, methyltransferase (S. cerevisiae) | 169.2618 |
| AGGF1 | angiogenic factor with G patch and FHA domains 1 | 168.2965 |
| RAB6B | RAB6B, member RAS oncogene family | 167.7459 |
| CASP2 | caspase 2, apoptosis-related cysteine peptidase | 167.6354 |
| SYT1 | synaptotagmin I | 167.4314 |
| L1CAM | L1 cell adhesion molecule | 167.3948 |
| BAZ1A | bromodomain adjacent to zinc finger domain, 1A | 167.3682 |
| GMDS | GDP-mannose 4,6-dehydratase | 166.4115 |
| EPHA4 | EPH receptor A4 | 166.1674 |
| CLDND1 | claudin domain containing 1 | 166.147 |
| SLC16A7 | solute carrier family 16, member 7 (monocarboxylic acid transporter 2) | 166.1434 |
| HSPA4 | heat shock 70kDa protein 4 | 165.7818 |
| DOK4 | docking protein 4 | 165.2872 |
| BF214329 | mitochondrial fission regulator 1 | 163.9384 |
| ZNF609 | zinc finger protein 609 | 163.207 |
| STX7 | syntaxin 7 | 162.9213 |
| EBI3 | Epstein-Barr virus induced 3 | 162.4585 |
| LMTK2 | lemur tyrosine kinase 2 | 162.0193 |
| RFK | riboflavin kinase | 161.709 |
| MEX3D | mex-3 homolog D (C. elegans) | 161.2357 |
| IGFBP3 | insulin-like growth factor binding protein 3 | 160.6901 |
| KLHL20 | kelch-like 20 (Drosophila) | 160.5198 |
| COL14A1 | collagen, type XIV, alpha 1 | 160.1152 |
| ZNF287 | zinc finger protein 287 | 160.0783 |
| TPM1 | tropomyosin 1 (alpha) | 160.0174 |
| PRDX2 | peroxiredoxin 2 | 159.4838 |
| STMN1 | stathmin 1 | 158.6602 |
| PODXL | podocalyxin-like | 158.6376 |
| CCNA2 | cyclin A2 | 158.4889 |
| ARHGAP24 | Rho GTPase activating protein 24 | 158.4705 |
| BAZ1A | bromodomain adjacent to zinc finger domain, 1A | 158.3138 |
| SNAPC1 | small nuclear RNA activating complex, polypeptide 1, 43kDa | 157.5673 |
| MAP3K7 | mitogen-activated protein kinase kinase kinase 7 | 157.4995 |
| KIAA0101 | KIAA0101 | 157.0187 |
| HS6ST1 | heparan sulfate 6-O-sulfotransferase 1 | 156.9563 |
| CKAP2 | cytoskeleton associated protein 2 | 156.6293 |
| GAS6 | similar to growth arrest-specific 6; growth arrest-specific 6 | 156.3795 |
| HDGFRP3 | hepatoma-derived growth factor, related protein 3 | 155.6027 |
| ANKRD12 | ankyrin repeat domain 12 | 155.077 |
| UBE2L3 | ubiquitin-conjugating enzyme E2L 3 | 154.8555 |
| PHTF2 | putative homeodomain transcription factor 2 | 154.6694 |
| MAGEH1 | melanoma antigen family H, 1 | 154.4223 |
| RNF146 | ring finger protein 146 | 153.972 |
| AFF4 | AF4/FMR2 family, member 4 | 153.4469 |
| RTN2 | reticulon 2 | 152.9258 |
| PAIP1 | poly(A) binding protein interacting protein 1; similar to poly(A) binding protein interacting protein 1 | 152.7779 |
| PLXNC1 | plexin C1 | 151.2545 |
| ZNF688 | zinc finger protein 688; zinc finger protein 785 | 149.7279 |
| ARHGAP12 | Rho GTPase activating protein 12 | 149.3361 |
| NEO1 | neogenin homolog 1 (chicken) | 149.1246 |
| NEFL | neurofilament, light polypeptide | 149.0977 |
| DHCR7 | 7-dehydrocholesterol reductase | 148.3973 |
| RTN2 | reticulon 2 | 148.1655 |
| STX7 | syntaxin 7 | 147.8903 |
| NUSAP1 | nucleolar and spindle associated protein 1 | 147.8902 |
| DOK4 | docking protein 4 | 147.771 |
| AL567227 | TIA1 cytotoxic granule-associated RNA binding protein | 147.6371 |
| CDC2L6 | cell division cycle 2-like 6 (CDK8-like) | 147.3589 |
| N20923 | FYN oncogene related to SRC, FGR, YES | 147.2938 |
| PRDX2 | peroxiredoxin 2 | 146.6907 |
| GPR56 | G protein-coupled receptor 56 | 146.6154 |
| SFXN1 | sideroflexin 1 | 146.4883 |
| HSPA4 | heat shock 70kDa protein 4 | 146.4232 |
| SLC7A1 | solute carrier family 7 (cationic amino acid transporter, y+ system), member 1 | 146.1519 |
| LRRC8B | leucine rich repeat containing 8 family, member B | 145.5263 |
| TCF4 | transcription factor 4 | 145.5251 |
| ZC3H14 | zinc finger CCCH-type containing 14 | 145.3507 |
| PKIA | protein kinase (cAMP-dependent, catalytic) inhibitor alpha | 145.3086 |
| FAM49B | family with sequence similarity 49, member B | 145.0607 |
| WBP5 | WW domain binding protein 5 | 144.2398 |
| SMC1A | structural maintenance of chromosomes 1A | 143.8874 |
| TTC35 | tetratricopeptide repeat domain 35 | 143.8262 |
| STAU2 | staufen, RNA binding protein, homolog 2 (Drosophila) | 143.7991 |
| CSNK1E | casein kinase 1, epsilon | 143.7418 |
| GSTM3 | glutathione S-transferase mu 3 (brain) | 143.7038 |
| AI907083 | aryl-hydrocarbon receptor repressor; programmed cell death 6 | 143.7038 |
| ACAT2 | acetyl-Coenzyme A acetyltransferase 2 | 143.2871 |
| AI922605 | collagen, type IV, alpha 1 | 142.8862 |
| TSPAN5 | tetraspanin 5 | 142.8838 |
| TASP1 | taspase, threonine aspartase, 1 | 142.1749 |
| SLC7A1 | solute carrier family 7 (cationic amino acid transporter, y+ system), member 1 | 142.0522 |
| RELN | reelin | 141.3933 |
| EPRS | glutamyl-prolyl-tRNA synthetase | 141.1689 |
| KIAA1128 | ribosomal protein S3A pseudogene 5; ribosomal protein S3a pseudogene 47; ribosomal protein S3a pseudogene 49; ribosomal protein S3A; hypothetical LOC100131699; hypothetical LOC100130107 | 140.1878 |
| NUP62 | nucleoporin 62kDa | 140.1402 |
| NRP1 | neuropilin 1 | 139.744 |
| DEPDC1 | DEP domain containing 1 | 139.3846 |
| GNB2L1 | guanine nucleotide binding protein (G protein), beta polypeptide 2-like 1 | 138.957 |
| KIAA1033 | KIAA1033 | 138.5495 |
| SSTR2 | somatostatin receptor 2 | 138.5375 |
| HMMR | hyaluronan-mediated motility receptor (RHAMM) | 138.3882 |
| ABLIM3 | actin binding LIM protein family, member 3 | 138.3343 |
| SLC1A1 | solute carrier family 1 (neuronal/epithelial high affinity glutamate transporter, system Xag), member 1 | 138.3275 |
| POU3F1 | POU class 3 homeobox 1 | 138.3013 |
| NR2F1 | nuclear receptor subfamily 2, group F, member 1 | 137.7677 |
| TPM1 | tropomyosin 1 (alpha) | 137.6417 |
| PDCD6 | aryl-hydrocarbon receptor repressor; programmed cell death 6 | 137.1934 |
| IGFBP3 | insulin-like growth factor binding protein 3 | 136.6349 |
| HS2ST1 | heparan sulfate 2-O-sulfotransferase 1 | 136.0432 |
| CLN8 | ceroid-lipofuscinosis, neuronal 8 (epilepsy, progressive with mental retardation) | 135.7767 |
| ATRX | alpha thalassemia/mental retardation syndrome X-linked (RAD54 homolog, S. cerevisiae) | 135.7491 |
| PAICS | phosphoribosylaminoimidazole carboxylase, phosphoribosylaminoimidazole succinocarboxamide synthetase | 135.4184 |
| SSR3 | signal sequence receptor, gamma (translocon-associated protein gamma) | 135.313 |
| C7orf25 | chromosome 7 open reading frame 25 | 135.106 |
| BMP2 | bone morphogenetic protein 2 | 135.0506 |
| GIPR | gastric inhibitory polypeptide receptor | 133.8777 |
| RPS6KA6 | ribosomal protein S6 kinase, 90kDa, polypeptide 6 | 133.4747 |
| STX3 | syntaxin 3 | 133.3633 |
| GNAO1 | guanine nucleotide binding protein (G protein), alpha activating activity polypeptide O | 132.7331 |
| WRB | tryptophan rich basic protein | 132.6726 |
| PARD6B | par-6 partitioning defective 6 homolog beta (C. elegans) | 132.117 |
| XRCC4 | X-ray repair complementing defective repair in Chinese hamster cells 4 | 131.3142 |
| NM_015936 | tyrosyl-tRNA synthetase 2, mitochondrial | 130.9167 |
| ASCC3 | activating signal cointegrator 1 complex subunit 3 | 130.8957 |
| CTPS | CTP synthase | 130.4042 |
| PIGO | phosphatidylinositol glycan anchor biosynthesis, class O | 130.1664 |
| ARNT2 | aryl-hydrocarbon receptor nuclear translocator 2 | 129.8208 |
| UTX | lysine (K)-specific demethylase 6A | 129.7212 |
| KIAA1033 | KIAA1033 | 129.6372 |
| PTPN12 | protein tyrosine phosphatase, non-receptor type 12 | 129.1878 |
| CD200 | CD200 molecule | 128.5182 |
| VCL | vinculin | 128.4231 |
| COL4A1 | collagen, type IV, alpha 1 | 128.3303 |
| RHOQ | ras homolog gene family, member Q; similar to small GTP binding protein TC10 | 127.8498 |
| PLXNC1 | plexin C1 | 127.8089 |
| RP5-1000E10.4 | suppressor of IKK epsilon | 127.4993 |
| SBNO1 | strawberry notch homolog 1 (Drosophila) | 126.2612 |
| DYRK2 | dual-specificity tyrosine-(Y)-phosphorylation regulated kinase 2 | 126.0995 |
| CDC42EP4 | CDC42 effector protein (Rho GTPase binding) 4 | 125.9598 |
| EPRS | glutamyl-prolyl-tRNA synthetase | 125.908 |
| C2orf34 | chromosome 2 open reading frame 34 | 125.6336 |
| SMAD2 | SMAD family member 2 | 125.0621 |
| RBP1 | retinol binding protein 1, cellular | 124.6758 |
| SERF1B | small EDRK-rich factor 1A (telomeric); small EDRK-rich factor 1B (centromeric) | 124.4735 |
| COL14A1 | collagen, type XIV, alpha 1 | 124.2007 |
| CCNA2 | cyclin A2 | 123.3431 |
| MTSS1 | metastasis suppressor 1 | 122.6648 |
| GPSM2 | G-protein signaling modulator 2 (AGS3-like, C. elegans) | 122.0804 |
| PCDHA10 | protocadherin alpha 13; protocadherin alpha 10; protocadherin alpha subfamily C, 1; protocadherin alpha subfamily C, 2 | 121.648 |
| TERF1 | similar to telomeric repeat binding factor (NIMA-interacting) 1; telomeric repeat binding factor (NIMA-interacting) 1; telomeric repeat binding factor (NIMA-interacting) 1 pseudogene | 120.8596 |
| CCT8 | similar to chaperonin containing TCP1, subunit 8 (theta); chaperonin containing TCP1, subunit 8 (theta) | 120.6806 |
| ANKRD17 | ankyrin repeat domain 17 | 120.6384 |
| D4S234E | DNA segment on chromosome 4 (unique) 234 expressed sequence | 119.9034 |
| PCMT1 | protein-L-isoaspartate (D-aspartate) O-methyltransferase | 119.6916 |
| AW103265 | mannosyl (alpha-1,6-)-glycoprotein beta-1,2-N-acetylglucosaminyltransferase | 119.5988 |
| CIB2 | calcium and integrin binding family member 2 | 118.3699 |
| DIMT1L | DIM1 dimethyladenosine transferase 1-like (S. cerevisiae) | 118.096 |
| FAM164A | family with sequence similarity 164, member A | 116.543 |
| AMOTL2 | angiomotin like 2 | 116.4979 |
| TWISTNB | TWIST neighbor | 116.243 |
| AZI2 | 5-azacytidine induced 2 | 115.9723 |
| TCF4 | transcription factor 4 | 115.8823 |
| CTNND2 | catenin (cadherin-associated protein), delta 2 (neural plakophilin-related arm-repeat protein) | 115.5802 |
| CRNKL1 | crooked neck pre-mRNA splicing factor-like 1 (Drosophila) | 115.5327 |
| HNRNPD | heterogeneous nuclear ribonucleoprotein D (AU-rich element RNA binding protein 1, 37kDa) | 115.424 |
| AI907083 | aryl-hydrocarbon receptor repressor; programmed cell death 6 | 114.8828 |
| RAD17 | RAD17 homolog (S. pombe) | 114.6202 |
| CSRP2 | cysteine and glycine-rich protein 2 | 113.8868 |
| CBLN1 | cerebellin 1 precursor | 113.4496 |
| HECW1 | HECT, C2 and WW domain containing E3 ubiquitin protein ligase 1 | 112.6404 |
| NETO2 | neuropilin (NRP) and tolloid (TLL)-like 2 | 112.3315 |
| FGF13 | fibroblast growth factor 13 | 111.9464 |
| APC2 | adenomatosis polyposis coli 2 | 111.8245 |
| B4GALT6 | UDP-Gal:betaGlcNAc beta 1,4- galactosyltransferase, polypeptide 6 | 111.5018 |
| TSPAN6 | tetraspanin 6 | 111.5018 |
| DACT1 | dapper, antagonist of beta-catenin, homolog 1 (Xenopus laevis) | 111.4278 |
| DPYSL3 | dihydropyrimidinase-like 3 | 110.8098 |
| PDPN | podoplanin | 110.2298 |
| MID1 | midline 1 (Opitz/BBB syndrome) | 109.6804 |
| M88163 | SWI/SNF related, matrix associated, actin dependent regulator of chromatin, subfamily a, member 1 | 108.7909 |
| GAP43 | growth associated protein 43 | 108.4719 |
| PTPN12 | protein tyrosine phosphatase, non-receptor type 12 | 108.4694 |
| YWHAE | similar to 14-3-3 protein epsilon (14-3-3E) (Mitochondrial import stimulation factor L subunit) (MSF L); tyrosine 3-monooxygenase/tryptophan 5-monooxygenase activation protein, epsilon polypeptide | 108.3518 |
| SGCB | sarcoglycan, beta (43kDa dystrophin-associated glycoprotein) | 107.405 |
| TMEM97 | transmembrane protein 97 | 107.405 |
| INHBB | inhibin, beta B | 107.1693 |
| LOC284393 | ribosomal protein L10; ribosomal protein L10 pseudogene 15; ribosomal protein L10 pseudogene 6; ribosomal protein L10 pseudogene 16; ribosomal protein L10 pseudogene 9 | 106.0199 |
| PREP | prolyl endopeptidase | 105.7658 |
| PCDHA5 | protocadherin alpha 5 | 105.33 |
| SATB2 | SATB homeobox 2 | 105.1775 |
| XRCC4 | X-ray repair complementing defective repair in Chinese hamster cells 4 | 104.6774 |
| AI767210 | major facilitator superfamily domain containing 9 | 104.2826 |
| HMMR | hyaluronan-mediated motility receptor (RHAMM) | 103.8272 |
| GPD2 | glycerol-3-phosphate dehydrogenase 2 (mitochondrial) | 103.4144 |
| C11orf24 | chromosome 11 open reading frame 24 | 103.1931 |
| SCAMP1 | secretory carrier membrane protein 1 | 102.4403 |
| SOX11 | SRY (sex determining region Y)-box 11 | 102.1226 |
| CCND1 | cyclin D1 | 102.1226 |
| POU3F3 | POU class 3 homeobox 3 | 101.9676 |
| RND1 | Rho family GTPase 1 | 101.7605 |
| FUT8 | fucosyltransferase 8 (alpha (1,6) fucosyltransferase) | 101.5024 |
| CD200 | CD200 molecule | 101.4476 |
| NM_018517 | kinesin heavy chain member 2A | 101.257 |
| RUNX1T1 | runt-related transcription factor 1; translocated to, 1 (cyclin D-related) | 101.0256 |
| KIF18B | kinesin family member 18B | 100.4305 |
| PFTK1 | PFTAIRE protein kinase 1 | 100.077 |
| KIF2A | kinesin heavy chain member 2A | 99.91643 |
| MTFR1 | mitochondrial fission regulator 1 | 99.85397 |
| SNAP91 | synaptosomal-associated protein, 91kDa homolog (mouse) | 99.85397 |
| ISOC1 | isochorismatase domain containing 1 | 99.64881 |
| ZNF22 | zinc finger protein 22 (KOX 15) | 99.5199 |
| LARP5 | La ribonucleoprotein domain family, member 4B | 99.46029 |
| CUL4B | cullin 4B | 99.20445 |
| METAP2 | methionyl aminopeptidase 2 | 98.4883 |
| PPFIA4 | protein tyrosine phosphatase, receptor type, f polypeptide (PTPRF), interacting protein (liprin), alpha 4 | 98.24064 |
| MYCN | v-myc myelocytomatosis viral related oncogene, neuroblastoma derived (avian) | 97.98869 |
| AL551971 | protein arginine methyltransferase 3 | 97.8906 |
| MEX3D | mex-3 homolog D (C. elegans) | 97.26864 |
| HIVEP2 | human immunodeficiency virus type I enhancer binding protein 2 | 97.23077 |
| PCDH21 | protocadherin 21 | 96.79498 |
| MAP1B | microtubule-associated protein 1B | 95.82813 |
| MAGI2 | putative homeodomain transcription factor 2 | 95.79565 |
| RP5-1000E10.4 | suppressor of IKK epsilon | 95.51313 |
| EHBP1 | EH domain binding protein 1 | 95.28242 |
| TERF1 | similar to telomeric repeat binding factor (NIMA-interacting) 1; telomeric repeat binding factor (NIMA-interacting) 1; telomeric repeat binding factor (NIMA-interacting) 1 pseudogene | 95.2044 |
| Sept6 | septin 6 | 95.20424 |
| SSTR1 | somatostatin receptor 1 | 94.83183 |
| EEF1E1 | eukaryotic translation elongation factor 1 epsilon 1 | 94.68025 |
| CTPS2 | CTP synthase II | 94.60903 |
| MELK | maternal embryonic leucine zipper kinase | 94.36483 |
| ATP2C1 | ATPase, Ca++ transporting, type 2C, member 1 | 94.34169 |
| PARD6B | par-6 partitioning defective 6 homolog beta (C. elegans) | 93.39113 |
| FARP1 | FERM, RhoGEF (ARHGEF) and pleckstrin domain protein 1 (chondrocyte-derived) | 93.28045 |
| SOX4 | SRY (sex determining region Y)-box 4 | 92.93634 |
| ANKRD12 | ankyrin repeat domain 12 | 92.8934 |
| DCX | doublecortin | 92.15305 |
| NM_030781 | collectin sub-family member 12 | 91.163 |
| SEC63 | SEC63 homolog (S. cerevisiae) | 91.06231 |
| AGGF1 | angiogenic factor with G patch and FHA domains 1 | 90.98009 |
| TCF4 | transcription factor 4 | 90.69104 |
| EIF2S1 | eukaryotic translation initiation factor 2, subunit 1 alpha, 35kDa | 90.34248 |
| CDC2 | cell division cycle 2, G1 to S and G2 to M | 90.24561 |
| MYCNOS | v-myc myelocytomatosis viral related oncogene, neuroblastoma derived (avian) | 90.0046 |
| LOC728969 | heparan sulfate 6-O-sulfotransferase 1 | 89.99775 |
| PCDHA2 | protocadherin alpha 2 | 89.8372 |
| VASH2 | vasohibin 2 | 89.65549 |
| SQLE | squalene epoxidase | 89.01941 |
| TPM1 | tropomyosin 1 (alpha) | 88.92763 |
| LARGE | like-glycosyltransferase | 88.08524 |
| SPTBN1 | spectrin, beta, non-erythrocytic 1 | 87.83156 |
| HNRNPD | heterogeneous nuclear ribonucleoprotein D (AU-rich element RNA binding protein 1, 37kDa) | 87.28604 |
| FAM3C | family with sequence similarity 3, member C | 87.12971 |
| CYP26B1 | cytochrome P450, family 26, subfamily B, polypeptide 1 | 86.65873 |
| MAP1B | microtubule-associated protein 1B | 85.92283 |
| FBXO21 | F-box protein 21 | 84.42634 |
| COL3A1 | collagen, type III, alpha 1 | 84.23593 |
| SCAPER | S-phase cyclin A-associated protein in the ER | 83.94685 |
| SYNGR3 | synaptogyrin 3 | 83.931 |
| LXN | latexin | 83.931 |
| MAP4K3 | mitogen-activated protein kinase kinase kinase kinase 3 | 83.70524 |
| C10orf88 | chromosome 10 open reading frame 88 | 83.26361 |
| PCDHA2 | protocadherin alpha 2 | 82.96143 |
| TRIM32 | tripartite motif-containing 32 | 82.80739 |
| ORC6L | origin recognition complex, subunit 6 like (yeast) | 82.80556 |
| TCF4 | transcription factor 4 | 82.76931 |
| APAF1 | apoptotic peptidase activating factor 1 | 82.61526 |
| RSBN1 | round spermatid basic protein 1 | 82.4837 |
| SCAPER | S-phase cyclin A-associated protein in the ER | 82.26445 |
| FAM49B | family with sequence similarity 49, member B | 81.61386 |
| PSCD3 | cytohesin 3 | 81.48849 |
| RNF219 | ring finger protein 219 | 81.07037 |
| SLC16A7 | solute carrier family 16, member 7 (monocarboxylic acid transporter 2) | 81.00433 |
| CFDP1 | craniofacial development protein 1 | 80.98488 |
| RNF2 | ring finger protein 2 | 80.63201 |
| CROP | cisplatin resistance-associated overexpressed protein | 80.1728 |
| AW157202 | SRY (sex determining region Y)-box 11 | 79.44274 |
| DDAH1 | dimethylarginine dimethylaminohydrolase 1 | 78.91865 |
| REEP1 | receptor accessory protein 1 | 78.69795 |
| CASP2 | caspase 2, apoptosis-related cysteine peptidase | 78.4772 |
| GOLIM4 | golgi integral membrane protein 4 | 77.08001 |
| SOX4 | SRY (sex determining region Y)-box 4 | 76.81377 |
| RRP15 | ribosomal RNA processing 15 homolog (S. cerevisiae) | 76.77826 |
| CTNNA2 | catenin (cadherin-associated protein), alpha 2 | 76.70667 |
| LDB2 | LIM domain binding 2 | 76.4411 |
| MTF2 | metal response element binding transcription factor 2 | 76.39253 |
| SCAMP1 | secretory carrier membrane protein 1 | 76.22903 |
| BAALC | brain and acute leukemia, cytoplasmic | 76.20893 |
| R38475 | microfibrillar-associated protein 3 | 75.75175 |
| SUCLA2 | succinate-CoA ligase, ADP-forming, beta subunit | 75.33878 |
| YBX1 | Y box binding protein 1 | 74.97156 |
| RND3 | Rho family GTPase 3 | 74.96232 |
| USP27X | ubiquitin specific peptidase 27, X-linked | 74.56855 |
| VEZT | vezatin, adherens junctions transmembrane protein | 74.33475 |
| SBNO1 | strawberry notch homolog 1 (Drosophila) | 74.09355 |
| FOXJ3 | forkhead box J3 | 72.89409 |
| GOLSYN | Golgi-localized protein | 72.64215 |
| UBE2D2 | ubiquitin-conjugating enzyme E2D 2 (UBC4/5 homolog, yeast) | 71.83991 |
| NRP1 | neuropilin 1 | 71.49611 |
| SMARCA1 | SWI/SNF related, matrix associated, actin dependent regulator of chromatin, subfamily a, member 1 | 70.7376 |
| SBNO1 | strawberry notch homolog 1 (Drosophila) | 70.7376 |
| PRKAA2 | protein kinase, AMP-activated, alpha 2 catalytic subunit | 70.14468 |
| ENC1 | ectodermal-neural cortex (with BTB-like domain) | 70.09309 |
| ELAVL2 | ELAV (embryonic lethal, abnormal vision, Drosophila)-like 2 (Hu antigen B) | 69.80861 |
| DIAPH3 | diaphanous homolog 3 (Drosophila) | 68.83012 |
| Sept11 | septin 11 | 68.44804 |
| RWDD2A | RWD domain containing 2A | 68.38605 |
| SLC2A11 | solute carrier family 2 (facilitated glucose transporter), member 11 | 68.24764 |
| RPL5 | family with sequence similarity 69, member A | 67.81431 |
| COL3A1 | collagen, type III, alpha 1 | 67.6619 |
| SMC2 | structural maintenance of chromosomes 2 | 67.52274 |
| MID1 | midline 1 (Opitz/BBB syndrome) | 67.37238 |
| FAT | FAT tumor suppressor homolog 1 (Drosophila) | 66.87374 |
| GNAO1 | guanine nucleotide binding protein (G protein), alpha activating activity polypeptide O | 66.78962 |
| GPD2 | glycerol-3-phosphate dehydrogenase 2 (mitochondrial) | 66.57959 |
| SOCS5 | suppressor of cytokine signaling 5 | 66.45128 |
| ZNF180 | zinc finger protein 180 | 66.33657 |
| DOK4 | docking protein 4 | 65.90905 |
| XRCC4 | X-ray repair complementing defective repair in Chinese hamster cells 4 | 65.81233 |
| PPP2R2B | protein phosphatase 2 (formerly 2A), regulatory subunit B, beta isoform | 65.571 |
| NEFL | neurofilament, light polypeptide | 65.03506 |
| POLR3F | polymerase (RNA) III (DNA directed) polypeptide F, 39 kDa | 64.69505 |
| ANAPC10 | anaphase promoting complex subunit 10; anaphase promoting complex subunit 10 pseudogene | 64.19504 |
| PPP4R2 | hypothetical LOC100129064 | 63.92514 |
| BF967657 | stathmin-like 2 | 63.43761 |
| ZWILCH | Zwilch, kinetochore associated, homolog (Drosophila) | 63.41517 |
| MINPP1 | multiple inositol polyphosphate histidine phosphatase, 1 | 63.21217 |
| BE794962 | nucleoporin 62kDa | 63.0475 |
| PODXL2 | podocalyxin-like 2 | 62.61933 |
| RASA1 | RAS p21 protein activator (GTPase activating protein) 1 | 62.57022 |
| MPPED2 | metallophosphoesterase domain containing 2 | 62.45671 |
| ANKRD46 | ankyrin repeat domain 46 | 62.12708 |
| DDX6 | DEAD (Asp-Glu-Ala-Asp) box polypeptide 6 | 61.33255 |
| TBL1XR1 | transducin (beta)-like 1 X-linked receptor 1 | 60.5497 |
| NRG1 | neuregulin 1 | 60.38922 |
| GALNT2 | UDP-N-acetyl-alpha-D-galactosamine:polypeptide N-acetylgalactosaminyltransferase 2 (GalNAc-T2) | 60.31554 |
| CCND1 | cyclin D1 | 60.14583 |
| GREM2 | gremlin 2, cysteine knot superfamily, homolog (Xenopus laevis) | 60.12417 |
| CDO1 | cysteine dioxygenase, type I | 59.45702 |
| TIA1 | TIA1 cytotoxic granule-associated RNA binding protein | 59.28335 |
| STMN2 | stathmin-like 2 | 59.06498 |
| SSTR2 | somatostatin receptor 2 | 58.56541 |
| PDAP1 | PDGFA associated protein 1; similar to PDGFA associated protein 1 | 58.14566 |
| KIAA0644 | KIAA0644 gene product | 58.12563 |
| SQSTM1 | sequestosome 1 | 57.99355 |
| PVRL3 | poliovirus receptor-related 3 | 57.85201 |
| WDHD1 | WD repeat and HMG-box DNA binding protein 1 | 57.33254 |
| DZIP3 | DAZ interacting protein 3, zinc finger | 56.91803 |
| PTPRG | protein tyrosine phosphatase, receptor type, G | 56.64194 |
| ZFPM2 | zinc finger protein, multitype 2 | 56.00087 |
| TIA1 | TIA1 cytotoxic granule-associated RNA binding protein | 55.98131 |
| RNGTT | RNA guanylyltransferase and 5'-phosphatase | 55.59481 |
| SCAMP1 | secretory carrier membrane protein 1 | 55.30848 |
| ARPP-21 | cyclic AMP-regulated phosphoprotein, 21 kD | 55.30848 |
| ADFP | adipose differentiation-related protein | 55.26107 |
| DYM | dymeclin | 54.75745 |
| SLC30A4 | solute carrier family 30 (zinc transporter), member 4 | 54.55513 |
| IPO5 | importin 5 | 54.3622 |
| AF041209 | midline 1 (Opitz/BBB syndrome) | 53.87441 |
| LARP5 | La ribonucleoprotein domain family, member 4B | 53.62907 |
| B4GALT6 | UDP-Gal:betaGlcNAc beta 1,4- galactosyltransferase, polypeptide 6 | 53.31522 |
| SGCB | sarcoglycan, beta (43kDa dystrophin-associated glycoprotein) | 52.56683 |
| KLHL20 | kelch-like 20 (Drosophila) | 52.25021 |
| RNGTT | RNA guanylyltransferase and 5'-phosphatase | 51.29087 |
| CDH11 | cadherin 11, type 2, OB-cadherin (osteoblast) | 50.91054 |
| NARG1 | NMDA receptor regulated 1 | 50.90529 |
| HS2ST1 | heparan sulfate 2-O-sulfotransferase 1 | 50.78349 |
| SLC7A1 | solute carrier family 7 (cationic amino acid transporter, y+ system), member 1 | 50.44084 |
| MSH2 | mutS homolog 2, colon cancer, nonpolyposis type 1 (E. coli) | 50.32224 |
| TLK1 | tousled-like kinase 1 | 50.30868 |
| SLC35F5 | solute carrier family 35, member F5 | 50.29599 |
| EXOC5 | exocyst complex component 5 | 50.2267 |
| TCF4 | transcription factor 4 | 50.14594 |
| APAF1 | apoptotic peptidase activating factor 1 | 50.10685 |
| HS2ST1 | heparan sulfate 2-O-sulfotransferase 1 | 49.87847 |
| ARL15 | ADP-ribosylation factor-like 15 | 49.70666 |
| NEFL | neurofilament, light polypeptide | 49.60147 |
| ABCA5 | ATP-binding cassette, sub-family A (ABC1), member 5 | 49.42963 |
| GPSM2 | G-protein signaling modulator 2 (AGS3-like, C. elegans) | 49.42963 |
| BF674842 | thymine-DNA glycosylase pseudogene | 49.1804 |
| PDGFRA | platelet-derived growth factor receptor, alpha polypeptide | 49.16096 |
| MYO5A | myosin VA (heavy chain 12, myoxin) | 48.9873 |
| SCAMP1 | secretory carrier membrane protein 1 | 48.8889 |
| PCDHA6 | protocadherin alpha 8; protocadherin alpha 6 | 48.18628 |
| AU154455 | podoplanin | 48.1791 |
| BMP2 | bone morphogenetic protein 2 | 48.13411 |
| SPTBN1 | spectrin, beta, non-erythrocytic 1 | 47.79672 |
| ZNF287 | zinc finger protein 287 | 47.65071 |
| NM_018104 | odz, odd Oz/ten-m homolog 3 (Drosophila) | 47.08846 |
| PAICS | phosphoribosylaminoimidazole carboxylase, phosphoribosylaminoimidazole succinocarboxamide synthetase | 47.07565 |
| PLAA | phospholipase A2-activating protein | 46.93454 |
| DAAM1 | dishevelled associated activator of morphogenesis 1 | 46.57808 |
| RUNX1T1 | runt-related transcription factor 1; translocated to, 1 (cyclin D-related) | 46.48499 |
| UTX | lysine (K)-specific demethylase 6A | 46.4568 |
| FAM69A | family with sequence similarity 69, member A | 46.16043 |
| SYT1 | synaptotagmin I | 45.68858 |
| SLC1A1 | solute carrier family 1 (neuronal/epithelial high affinity glutamate transporter, system Xag), member 1 | 45.26164 |
| HDGFRP3 | hepatoma-derived growth factor, related protein 3 | 45.26164 |
| C1orf176 | defects in morphology 1 homolog (S. cerevisiae) | 44.65409 |
| BF223370 | major facilitator superfamily domain containing 9 | 44.44125 |
| K03199 | tumor protein p53 | 43.79167 |
| ZNF292 | zinc finger protein 292 | 43.7471 |
| BPNT1 | 3'(2'), 5'-bisphosphate nucleotidase 1 | 43.74538 |
| MAOA | monoamine oxidase A | 43.66131 |
| SOX11 | SRY (sex determining region Y)-box 11 | 43.65713 |
| BCAT1 | branched chain aminotransferase 1, cytosolic | 43.40817 |
| SENP7 | SUMO1/sentrin specific peptidase 7 | 43.25835 |
| SMC2 | structural maintenance of chromosomes 2 | 42.73666 |
| P15RS | regulation of nuclear pre-mRNA domain containing 1A | 42.66115 |
| APPBP2 | amyloid beta precursor protein (cytoplasmic tail) binding protein 2 | 42.1792 |
| NM_018581 | signal recognition particle 72kDa | 41.96384 |
| POGZ | pogo transposable element with ZNF domain | 41.91286 |
| APPBP2 | amyloid beta precursor protein (cytoplasmic tail) binding protein 2 | 41.58303 |
| TSC22D2 | TSC22 domain family, member 2 | 41.53561 |
| SRR | serine racemase | 41.45745 |
| EIF4G2 | eukaryotic translation initiation factor 4 gamma, 2 | 41.42048 |
| CSRP2 | cysteine and glycine-rich protein 2 | 41.35201 |
| PURG | purine-rich element binding protein G | 41.35201 |
| PDGFRA | platelet-derived growth factor receptor, alpha polypeptide | 41.1791 |
| NAP1L3 | nucleosome assembly protein 1-like 3 | 41.16154 |
| PRKACB | protein kinase, cAMP-dependent, catalytic, beta | 40.99029 |
| DCX | doublecortin | 40.78236 |
| ATRX | alpha thalassemia/mental retardation syndrome X-linked (RAD54 homolog, S. cerevisiae) | 40.21005 |
| TUBB2B | tubulin, beta 2B | 40.20084 |
| NEDD4 | neural precursor cell expressed, developmentally down-regulated 4 | 40.14717 |
| NM_018627 | calmodulin regulated spectrin-associated protein 1 | 39.98181 |
| NR2F1 | nuclear receptor subfamily 2, group F, member 1 | 39.87782 |
| GABRA5 | gamma-aminobutyric acid (GABA) A receptor, alpha 5 | 39.59397 |
| CNN3 | calponin 3, acidic | 39.15628 |
| PCDHA12 | protocadherin alpha 13; protocadherin alpha 10; protocadherin alpha subfamily C, 1; protocadherin alpha subfamily C, 2 | 39.13364 |
| RFXAP | regulatory factor X-associated protein | 39.12625 |
| CDH2 | cadherin 2, type 1, N-cadherin (neuronal) | 38.61316 |
| WIF1 | WNT inhibitory factor 1 | 38.61316 |
| BC002836 | EF-hand calcium binding domain 2 | 38.05615 |
| PDPN | podoplanin | 37.63936 |
| RUNX1T1 | runt-related transcription factor 1; translocated to, 1 (cyclin D-related) | 37.36808 |
| APPBP2 | amyloid beta precursor protein (cytoplasmic tail) binding protein 2 | 37.29612 |
| NUDT4 | nudix (nucleoside diphosphate linked moiety X)-type motif 4; nudix (nucleoside diphosphate linked moiety X)-type motif 4 pseudogene 1 | 37.20246 |
| NCKAP1 | NCK-associated protein 1 | 37.13481 |
| MTHFD2L | methylenetetrahydrofolate dehydrogenase (NADP+ dependent) 2-like | 37.02023 |
| CTNND2 | catenin (cadherin-associated protein), delta 2 (neural plakophilin-related arm-repeat protein) | 36.95315 |
| FAT4 | FAT tumor suppressor homolog 4 (Drosophila) | 36.89838 |
| C19orf2 | chromosome 19 open reading frame 2 | 36.8289 |
| CDH13 | cadherin 13, H-cadherin (heart) | 36.76882 |
| LGI1 | leucine-rich, glioma inactivated 1 | 36.74039 |
| AL049545 | ribosomal protein L7 pseudogene 26; ribosomal protein L7 pseudogene 16; ribosomal protein L7; ribosomal protein L7 pseudogene 32; ribosomal protein L7 pseudogene 23; ribosomal protein L7 pseudogene 24; ribosomal protein L7 pseudogene 20 | 36.59771 |
| NIP7 | nuclear import 7 homolog (S. cerevisiae) | 36.16726 |
| DZIP3 | DAZ interacting protein 3, zinc finger | 35.75115 |
| SNX16 | sorting nexin 16 | 35.52851 |
| RAB14 | RAB14, member RAS oncogene family | 35.12222 |
| TRIM33 | tripartite motif-containing 33 | 34.6973 |
| RAB7A | RAB7A, member RAS oncogene family | 34.547 |
| DIMT1L | DIM1 dimethyladenosine transferase 1-like (S. cerevisiae) | 34.50265 |
| SMARCA1 | SWI/SNF related, matrix associated, actin dependent regulator of chromatin, subfamily a, member 1 | 34.47022 |
| TMEM132A | solute carrier family 25, member 30 | 34.4168 |
| GABRA5 | gamma-aminobutyric acid (GABA) A receptor, alpha 5 | 34.3836 |
| PAPOLA | poly(A) polymerase alpha | 34.25167 |
| ZNF268 | zinc finger protein 268 | 34.1492 |
| NKX2-2 | NK2 homeobox 2 | 34.03408 |
| GPR85 | G protein-coupled receptor 85 | 33.93071 |
| KIDINS220 | kinase D-interacting substrate, 220kDa | 33.70662 |
| TMEFF1 | transmembrane protein with EGF-like and two follistatin-like domains 1; chromosome 9 open reading frame 30; hypothetical LOC729538 | 33.45547 |
| TLK1 | tousled-like kinase 1 | 33.2054 |
| COL14A1 | collagen, type XIV, alpha 1 | 33.20062 |
| STMN3 | stathmin-like 3 | 33.19436 |
| NCKAP1 | NCK-associated protein 1 | 33.14171 |
| EIF3F | eukaryotic translation initiation factor 3, subunit F; similar to hCG2040283 | 33.11707 |
| CUL2 | cullin 2 | 32.67528 |
| UGCG | UDP-glucose ceramide glucosyltransferase | 32.66368 |
| VHL | von Hippel-Lindau tumor suppressor | 32.43727 |
| DYRK2 | dual-specificity tyrosine-(Y)-phosphorylation regulated kinase 2 | 32.4243 |
| RP5-1000E10.4 | suppressor of IKK epsilon | 32.3385 |
| CTGF | connective tissue growth factor | 32.22049 |
| PCYOX1 | prenylcysteine oxidase 1 | 31.87229 |
| BMP5 | bone morphogenetic protein 5 | 31.69478 |
| LYVE1 | lymphatic vessel endothelial hyaluronan receptor 1 | 31.53641 |
| MPPED2 | metallophosphoesterase domain containing 2 | 31.51598 |
| C15orf29 | chromosome 15 open reading frame 29 | 31.31647 |
| DPH5 | DPH5 homolog (S. cerevisiae) | 31.01528 |
| TM2D1 | TM2 domain containing 1 | 30.98915 |
| MAD2L1 | MAD2 mitotic arrest deficient-like 1 (yeast) | 30.83231 |
| BE620457 | neuropilin 1 | 30.51664 |
| WDHD1 | WD repeat and HMG-box DNA binding protein 1 | 30.33234 |
| MAOA | monoamine oxidase A | 30.2878 |
| RNF128 | ring finger protein 128 | 30.2878 |
| IFT81 | intraflagellar transport 81 homolog (Chlamydomonas) | 29.99235 |
| RACGAP1 | Rac GTPase activating protein 1 pseudogene; Rac GTPase activating protein 1 | 29.51302 |
| RRP15 | ribosomal RNA processing 15 homolog (S. cerevisiae) | 29.33077 |
| XRCC4 | X-ray repair complementing defective repair in Chinese hamster cells 4 | 29.17171 |
| LOC100101247 | ribosomal protein L7 pseudogene 26; ribosomal protein L7 pseudogene 16; ribosomal protein L7; ribosomal protein L7 pseudogene 32; ribosomal protein L7 pseudogene 23; ribosomal protein L7 pseudogene 24; ribosomal protein L7 pseudogene 20 | 28.42337 |
| YIPF4 | Yip1 domain family, member 4 | 27.69681 |
| SEMA6A | sema domain, transmembrane domain (TM), and cytoplasmic domain, (semaphorin) 6A | 27.66494 |
| ANKRD12 | ankyrin repeat domain 12 | 27.52772 |
| GAP43 | growth associated protein 43 | 27.4937 |
| CD47 | CD47 molecule | 27.38767 |
| NUP205 | nucleoporin 205kDa | 27.31781 |
| OGN | osteoglycin | 27.20541 |
| Sept11 | septin 11 | 27.1435 |
| AF130082 | collagen, type III, alpha 1 | 26.56351 |
| ATRX | alpha thalassemia/mental retardation syndrome X-linked (RAD54 homolog, S. cerevisiae) | 26.44428 |
| RAD23B | similar to UV excision repair protein RAD23 homolog B | 26.13442 |
| NLGN1 | neuroligin 1 | 26.09495 |
| TTC28 | chromosome 6 open reading frame 35; hCG1820764; tetratricopeptide repeat domain 28 | 26.00413 |
| PPP2R3A | protein phosphatase 2 (formerly 2A), regulatory subunit B'', alpha | 25.85954 |
| RALGPS2 | Ral GEF with PH domain and SH3 binding motif 2 | 25.85954 |
| LARGE | like-glycosyltransferase | 25.85501 |
| SCN9A | sodium channel, voltage-gated, type IX, alpha subunit | 25.62018 |
| R41498 | leucine rich repeat containing 8 family, member B | 25.31762 |
| FAIM | Fas apoptotic inhibitory molecule | 25.31083 |
| RFXDC2 | regulatory factor X, 7 | 25.21104 |
| CDC2 | cell division cycle 2, G1 to S and G2 to M | 25.10248 |
| SATB2 | SATB homeobox 2 | 24.61007 |
| SMARCA1 | SWI/SNF related, matrix associated, actin dependent regulator of chromatin, subfamily a, member 1 | 24.49294 |
| C7orf25 | chromosome 7 open reading frame 25 | 24.44468 |
| PKN2 | protein kinase N2 | 24.37882 |
| WDHD1 | WD repeat and HMG-box DNA binding protein 1 | 24.04769 |
| ECT2 | epithelial cell transforming sequence 2 oncogene | 24.02899 |
| PBRM1 | polybromo 1 | 23.66776 |
| UBE2D1 | ubiquitin-conjugating enzyme E2D 1 (UBC4/5 homolog, yeast) | 23.43243 |
| PHF14 | PHD finger protein 14 | 22.89809 |
| RAB7A | RAB7A, member RAS oncogene family | 22.12462 |
| LUM | lumican | 21.49656 |
| TMEFF1 | transmembrane protein with EGF-like and two follistatin-like domains 1; chromosome 9 open reading frame 30; hypothetical LOC729538 | 21.06825 |
| ITGA1 | pelota homolog (Drosophila) | 19.00766 |

**Monocytic genes specific to RMC**

| **Probe Set ID** | **Gene Symbol** | **Gene Name** | **Signal intensity in monocytes** |
| --- | --- | --- | --- |
| 1294_at | UBA7 | ubiquitin-like modifier activating enzyme 7 | 612.8697 |
| 1487_at | ESRRA | estrogen-related receptor alpha | 598.0486 |
| 200616_s_at | KIAA0152 | malectin | 234.5525 |
| 200617_at | KIAA0152 | malectin | 437.8586 |
| 200621_at | CSRP1 | cysteine and glycine-rich protein 1 | 970.9595 |
| 200685_at | SFRS11 | splicing factor, arginine/serine-rich 11 | 53.67013 |
| 200686_s_at | SFRS11 | splicing factor, arginine/serine-rich 11 | 1933.024 |
| 200694_s_at | DDX24 | DEAD (Asp-Glu-Ala-Asp) box polypeptide 24 | 1669.933 |
| 200696_s_at | GSN | gelsolin (amyloidosis, Finnish type) | 1208.741 |
| 200702_s_at | DDX24 | DEAD (Asp-Glu-Ala-Asp) box polypeptide 24 | 239.1787 |
| 200747_s_at | NUMA1 | nuclear mitotic apparatus protein 1 | 563.0861 |
| 200904_at | HLA-E | major histocompatibility complex, class I, E | 4933.363 |
| 200905_x_at | HLA-E | major histocompatibility complex, class I, E | 8901.316 |
| 201024_x_at | BG261322 | eukaryotic translation initiation factor 5B | 306.4077 |
| 201025_at | EIF5B | eukaryotic translation initiation factor 5B | 136.5852 |
| 201026_at | EIF5B | eukaryotic translation initiation factor 5B | 74.18983 |
| 201027_s_at | EIF5B | eukaryotic translation initiation factor 5B | 90.06409 |
| 201042_at | KIAA1755 | transglutaminase 2 (C polypeptide, protein-glutamine-gamma-glutamyltransferase) | 162.5347 |
| 201056_at | GOLGB1 | golgin B1, golgi integral membrane protein | 150.9202 |
| 201057_s_at | GOLGB1 | golgin B1, golgi integral membrane protein | 438.9077 |
| 201085_s_at | SON | SON DNA binding protein | 226.9825 |
| 201086_x_at | NM_003103 | SON DNA binding protein | 653.7834 |
| 201087_at | PXN | paxillin | 1328.593 |
| 201182_s_at | CHD4 | chromodomain helicase DNA binding protein 4 | 361.4079 |
| 201183_s_at | CHD4 | chromodomain helicase DNA binding protein 4 | 468.8627 |
| 201184_s_at | CHD4 | chromodomain helicase DNA binding protein 4 | 1084.803 |
| 201209_at | HDAC1 | histone deacetylase 1 | 877.9851 |
| 201221_s_at | SNRP70 | small nuclear ribonucleoprotein 70kDa (U1) | 551.2117 |
| 201235_s_at | BTG2 | BTG family, member 2 | 406.693 |
| 201236_s_at | BTG2 | BTG family, member 2 | 4087.162 |
| 201473_at | JUNB | jun B proto-oncogene | 3550.39 |
| 201495_x_at | MYH11 | myosin, heavy chain 11, smooth muscle | 75.09352 |
| 201496_x_at | MYH11 | myosin, heavy chain 11, smooth muscle | 56.92149 |
| 201497_x_at | MYH11 | myosin, heavy chain 11, smooth muscle | 576.9076 |
| 201602_s_at | PPP1R12A | protein phosphatase 1, regulatory (inhibitor) subunit 12A | 43.95693 |
| 201603_at | AI817061 | protein phosphatase 1, regulatory (inhibitor) subunit 12A | 324.2758 |
| 201604_s_at | PPP1R12A | protein phosphatase 1, regulatory (inhibitor) subunit 12A | 226.6304 |
| 201651_s_at | PACSIN2 | protein kinase C and casein kinase substrate in neurons 2 | 1822.203 |
| 201693_s_at | EGR1 | early growth response 1 | 404.5944 |
| 201694_s_at | EGR1 | early growth response 1 | 1290.416 |
| 201765_s_at | HEXA | hexosaminidase A (alpha polypeptide) | 1325.896 |
| 201904_s_at | CTDSPL | CTD (carboxy-terminal domain, RNA polymerase II, polypeptide A) small phosphatase-like | 184.2053 |
| 201905_s_at | CTDSPL | CTD (carboxy-terminal domain, RNA polymerase II, polypeptide A) small phosphatase-like | 178.5843 |
| 201906_s_at | CTDSPL | CTD (carboxy-terminal domain, RNA polymerase II, polypeptide A) small phosphatase-like | 145.3569 |
| 202022_at | ALDOC | aldolase C, fructose-bisphosphate | 353.2282 |
| 202032_s_at | MAN2A2 | mannosidase, alpha, class 2A, member 2 | 1371.133 |
| 202082_s_at | SEC14L1 | SEC14-like 1 (S. cerevisiae); SEC14-like 1 pseudogene | 487.2963 |
| 202083_s_at | SEC14L1 | SEC14-like 1 (S. cerevisiae); SEC14-like 1 pseudogene | 501.8144 |
| 202084_s_at | SEC14L1 | SEC14-like 1 (S. cerevisiae); SEC14-like 1 pseudogene | 2940.183 |
| 202090_s_at | UQCR | ubiquinol-cytochrome c reductase, 6.4kDa subunit | 2924.765 |
| 202140_s_at | CLK3 | CDC-like kinase 3 | 758.0971 |
| 202190_at | CSTF1 | cleavage stimulation factor, 3' pre-RNA, subunit 1, 50kDa | 184.9433 |
| 202254_at | SIPA1L1 | signal-induced proliferation-associated 1 like 1 | 174.4807 |
| 202255_s_at | SIPA1L1 | signal-induced proliferation-associated 1 like 1 | 521.2867 |
| 202283_at | SERPINF1 | serpin peptidase inhibitor, clade F (alpha-2 antiplasmin, pigment epithelium derived factor), member 1 | 516.0627 |
| 202308_at | SREBF1 | sterol regulatory element binding transcription factor 1 | 438.9077 |
| 202339_at | SYMPK | symplekin | 220.472 |
| 202340_x_at | NR4A1 | nuclear receptor subfamily 4, group A, member 1 | 1467.845 |
| 202350_s_at | MATN2 | matrilin 2 | 28.92392 |
| 202426_s_at | RXRA | retinoid X receptor, alpha | 1112.737 |
| 202449_s_at | RXRA | retinoid X receptor, alpha | 952.6228 |
| 202468_s_at | CTNNAL1 | catenin (cadherin-associated protein), alpha-like 1 | 228.4033 |
| 202545_at | PRKCD | protein kinase C, delta | 686.858 |
| 202590_s_at | AL574319 | pyruvate dehydrogenase kinase, isozyme 2 | 198.5099 |
| 202621_at | IRF3 | interferon regulatory factor 3 | 298.8935 |
| 202623_at | EAPP | E2F-associated phosphoprotein | 1013.823 |
| 202758_s_at | RFXANK | regulatory factor X-associated ankyrin-containing protein | 392.9915 |
| 202773_s_at | SFRS8 | splicing factor, arginine/serine-rich 8 (suppressor-of-white-apricot homolog, Drosophila) | 310.2625 |
| 202774_s_at | SFRS8 | splicing factor, arginine/serine-rich 8 (suppressor-of-white-apricot homolog, Drosophila) | 651.8364 |
| 202775_s_at | SFRS8 | splicing factor, arginine/serine-rich 8 (suppressor-of-white-apricot homolog, Drosophila) | 403.6149 |
| 202818_s_at | TCEB3 | transcription elongation factor B (SIII), polypeptide 3 (110kDa, elongin A) | 647.4008 |
| 202819_s_at | TCEB3 | transcription elongation factor B (SIII), polypeptide 3 (110kDa, elongin A) | 635.9604 |
| 202834_at | AGT | angiotensinogen (serpin peptidase inhibitor, clade A, member 8) | 298.0745 |
| 202847_at | PCK2 | phosphoenolpyruvate carboxykinase 2 (mitochondrial) | 270.5883 |
| 202861_at | PER1 | period homolog 1 (Drosophila) | 689.6743 |
| 202862_at | FAH | fumarylacetoacetate hydrolase (fumarylacetoacetase) | 185.1356 |
| 202889_x_at | MAP7 | microtubule-associated protein 7 | 271.1744 |
| 202890_at | AW242297 | microtubule-associated protein 7 | 42.97602 |
| 202901_x_at | CTSS | cathepsin S | 1096.933 |
| 202902_s_at | CTSS | cathepsin S | 16330.29 |
| 202908_at | WFS1 | Wolfram syndrome 1 (wolframin) | 274.8506 |
| 202931_x_at | BIN1 | bridging integrator 1 | 726.718 |
| 202997_s_at | LOXL2 | lysyl oxidase-like 2 | 110.8125 |
| 202998_s_at | LOXL2 | lysyl oxidase-like 2 | 194.4814 |
| 202999_s_at | LOXL2 | lysyl oxidase-like 2 | 176.6374 |
| 203020_at | RABGAP1L | RAB GTPase activating protein 1-like | 401.6672 |
| 203074_at | ANXA8L2 | annexin A8-like 2 | 449.1783 |
| 203080_s_at | BAZ2B | bromodomain adjacent to zinc finger domain, 2B | 73.82624 |
| 203193_at | ESRRA | estrogen-related receptor alpha | 316.0748 |
| 203237_s_at | NOTCH3 | Notch homolog 3 (Drosophila) | 455.9323 |
| 203238_s_at | NOTCH3 | Notch homolog 3 (Drosophila) | 195.4101 |
| 203254_s_at | TLN1 | talin 1 | 334.6765 |
| 203281_s_at | UBA7 | ubiquitin-like modifier activating enzyme 7 | 780.9959 |
| 203385_at | DGKA | diacylglycerol kinase, alpha 80kDa | 634.1401 |
| 203391_at | FKBP2 | FK506 binding protein 2, 13kDa | 540.2725 |
| 203411_s_at | LMNA | lamin A/C | 968.9667 |
| 203523_at | LSP1 | lymphocyte-specific protein 1 | 3342.203 |
| 203541_s_at | KLF9 | Kruppel-like factor 9 | 162.2758 |
| 203542_s_at | KLF9 | Kruppel-like factor 9 | 464.8678 |
| 203543_s_at | KLF9 | Kruppel-like factor 9 | 435.7351 |
| 203551_s_at | COX11 | COX11 homolog, cytochrome c oxidase assembly protein (yeast) | 125.0985 |
| 203600_s_at | C4orf8 | chromosome 4 open reading frame 8 | 521.9997 |
| 203631_s_at | GPRC5B | G protein-coupled receptor, family C, group 5, member B | 133.9237 |
| 203632_s_at | GPRC5B | G protein-coupled receptor, family C, group 5, member B | 146.4492 |
| 203661_s_at | TMOD1 | tropomodulin 1 | 248.742 |
| 203662_s_at | TMOD1 | tropomodulin 1 | 308.9078 |
| 203670_at | NM_015644 | tubulin tyrosine ligase-like family, member 3; actin related protein 2/3 complex, subunit 4, 20kDa | 148.99 |
| 203704_s_at | RREB1 | ras responsive element binding protein 1 | 1068.574 |
| 203710_at | ITPR1 | inositol 1,4,5-triphosphate receptor, type 1 | 169.1406 |
| 203799_at | CD302 | CD302 molecule; lymphocyte antigen 75 | 4356.911 |
| 203827_at | WIPI1 | WD repeat domain, phosphoinositide interacting 1 | 451.4318 |
| 203841_x_at | MAPRE3 | microtubule-associated protein, RP/EB family, member 3 | 141.8211 |
| 203842_s_at | MAPRE3 | microtubule-associated protein, RP/EB family, member 3 | 240.9377 |
| 203880_at | COX17 | COX17 cytochrome c oxidase assembly homolog (S. cerevisiae) | 811.1779 |
| 203924_at | GSTA2 | glutathione S-transferase alpha 1 | 100.0687 |
| 203937_s_at | TAF1C | TATA box binding protein (TBP)-associated factor, RNA polymerase I, C, 110kDa | 228.9078 |
| 203938_s_at | TAF1C | TATA box binding protein (TBP)-associated factor, RNA polymerase I, C, 110kDa | 466.2703 |
| 203953_s_at | BE791251 | claudin 3 | 40.90498 |
| 203954_x_at | CLDN3 | claudin 3 | 186.1797 |
| 203963_at | CA12 | carbonic anhydrase XII | 175.4249 |
| 203979_at | CYP27A1 | cytochrome P450, family 27, subfamily A, polypeptide 1 | 1522.852 |
| 204036_at | LPAR1 | lysophosphatidic acid receptor 1 | 47.23265 |
| 204037_at | LPAR1 | lysophosphatidic acid receptor 1 | 74.86021 |
| 204038_s_at | LPAR1 | lysophosphatidic acid receptor 1 | 593.7743 |
| 204138_s_at | MZF1 | myeloid zinc finger 1 | 449.3412 |
| 204139_x_at | MZF1 | myeloid zinc finger 1 | 239.4556 |
| 204158_s_at | TCIRG1 | T-cell, immune regulator 1, ATPase, H+ transporting, lysosomal V0 subunit A3 | 1910.556 |
| 204197_s_at | RUNX3 | runt-related transcription factor 3 | 3507.965 |
| 204198_s_at | RUNX3 | runt-related transcription factor 3 | 4770.579 |
| 204215_at | C7orf23 | chromosome 7 open reading frame 23 | 577.4103 |
| 204412_s_at | NEFH | neurofilament, heavy polypeptide | 126.2358 |
| 204416_x_at | APOC1 | apolipoprotein C-I | 184.1585 |
| 204425_at | ARHGAP4 | Rho GTPase activating protein 4 | 537.2983 |
| 204443_at | ARSA | arylsulfatase A | 423.1563 |
| 204508_s_at | CA12 | carbonic anhydrase XII | 107.821 |
| 204509_at | CA12 | carbonic anhydrase XII | 163.7576 |
| 204519_s_at | PLLP | plasma membrane proteolipid (plasmolipin) | 259.0966 |
| 204520_x_at | BRD1 | bromodomain containing 1 | 824.4104 |
| 204537_s_at | GABRE | gamma-aminobutyric acid (GABA) A receptor, epsilon | 136.2859 |
| 204541_at | SEC14L2 | SEC14-like 2 (S. cerevisiae) | 116.6702 |
| 204578_at | HISPPD2A | histidine acid phosphatase domain containing 2A | 120.3298 |
| 204648_at | NPR1 | natriuretic peptide receptor A/guanylate cyclase A (atrionatriuretic peptide receptor A) | 317.0544 |
| 204674_at | LRMP | lymphoid-restricted membrane protein | 1188.221 |
| 204692_at | LRCH4 | leucine-rich repeats and calponin homology (CH) domain containing 4 | 781.8241 |
| 204693_at | CDC42EP1 | CDC42 effector protein (Rho GTPase binding) 1 | 313.2627 |
| 204740_at | CNKSR1 | connector enhancer of kinase suppressor of Ras 1 | 156.9585 |
| 204777_s_at | MAL | mal, T-cell differentiation protein | 183.0501 |
| 204854_at | LEPREL2 | leprecan-like 2 | 89.21308 |
| 204936_at | MAP4K2 | mitogen-activated protein kinase kinase kinase kinase 2 | 186.949 |
| 204958_at | PLK3 | polo-like kinase 3 (Drosophila) | 1226.945 |
| 204985_s_at | TRAPPC6A | trafficking protein particle complex 6A | 760.4963 |
| 204997_at | GPD1 | glycerol-3-phosphate dehydrogenase 1 (soluble) | 207.2419 |
| 205062_x_at | ARID4A | AT rich interactive domain 4A (RBP1-like) | 583.62 |
| 205158_at | RNASE4 | ribonuclease, RNase A family, 4 | 386.0016 |
| 205171_at | PTPN4 | protein tyrosine phosphatase, non-receptor type 4 (megakaryocyte) | 182.2298 |
| 205179_s_at | ADAM8 | ADAM metallopeptidase domain 8 | 132.1168 |
| 205180_s_at | ADAM8 | ADAM metallopeptidase domain 8 | 1479.181 |
| 205186_at | DNALI1 | dynein, axonemal, light intermediate chain 1 | 58.63685 |
| 205251_at | PER2 | period homolog 2 (Drosophila) | 997.259 |
| 205293_x_at | BAIAP2 | BAI1-associated protein 2 | 295.352 |
| 205294_at | BAIAP2 | BAI1-associated protein 2 | 418.2411 |
| 205322_s_at | MTF1 | metal-regulatory transcription factor 1 | 383.7395 |
| 205323_s_at | MTF1 | metal-regulatory transcription factor 1 | 529.5745 |
| 205325_at | PHYHIP | phytanoyl-CoA 2-hydroxylase interacting protein | 114.8451 |
| 205336_at | PVALB | parvalbumin | 202.8795 |
| 205354_at | GAMT | guanidinoacetate N-methyltransferase | 143.9179 |
| 205384_at | FXYD1 | FXYD domain containing ion transport regulator 1 | 232.7579 |
| 205388_at | TNNC2 | troponin C type 2 (fast) | 128.1688 |
| 205406_s_at | SPA17 | sperm autoantigenic protein 17 | 70.62321 |
| 205424_at | TBKBP1 | TBK1 binding protein 1 | 173.9706 |
| 205425_at | HIP1 | huntingtin interacting protein 1 | 109.7056 |
| 205426_s_at | HIP1 | huntingtin interacting protein 1 | 259.35 |
| 205477_s_at | AMBP | alpha-1-microglobulin/bikunin precursor | 113.1087 |
| 205508_at | SCN1B | sodium channel, voltage-gated, type I, beta | 260.7077 |
| 205531_s_at | GLS2 | glutaminase 2 (liver, mitochondrial) | 75.05349 |
| 205547_s_at | TAGLN | transgelin | 283.1432 |
| 205548_s_at | BTG3 | BTG family, member 3 | 1073.087 |
| 205640_at | ALDH3B1 | aldehyde dehydrogenase 3 family, member B1 | 1047.224 |
| 205695_at | SDS | serine dehydratase | 380.9548 |
| 205711_x_at | ATP5C1 | ATP synthase, H+ transporting, mitochondrial F1 complex, gamma polypeptide 1 | 3259.654 |
| 205714_s_at | ZMYND10 | zinc finger, MYND-type containing 10 | 164.3632 |
| 205723_at | CNTFR | ciliary neurotrophic factor receptor | 356.3745 |
| 205742_at | TNNI3 | troponin I type 3 (cardiac) | 199.099 |
| 205755_at | ITIH3 | inter-alpha (globulin) inhibitor H3 | 82.69316 |
| 205756_s_at | F8 | coagulation factor VIII, procoagulant component | 271.3784 |
| 205774_at | F12 | coagulation factor XII (Hageman factor) | 353.6237 |
| 205787_x_at | LOC727977 | zinc finger CCCH-type containing 11A | 430.4298 |
| 205788_s_at | ZC3H11A | zinc finger CCCH-type containing 11A | 2367.174 |
| 205847_at | PRSS22 | protease, serine, 22 | 243.2411 |
| 205859_at | LY86 | lymphocyte antigen 86 | 5218.314 |
| 205870_at | BDKRB2 | bradykinin receptor B2 | 177.5849 |
| 205918_at | SLC4A3 | solute carrier family 4, anion exchanger, member 3 | 221.886 |
| 205939_at | CYP3A7 | cytochrome P450, family 3, subfamily A, polypeptide 7 | 173.4043 |
| 205970_at | MT3 | metallothionein 3 | 232.1089 |
| 205985_x_at | CLCNKB | chloride channel Kb | 203.4089 |
| 205986_at | NM_004920 | apoptosis-associated tyrosine kinase | 682.5077 |
| 205989_s_at | MOG | myelin oligodendrocyte glycoprotein | 25.21153 |
| 206017_at | KIAA0319 | KIAA0319 | 59.29397 |
| 206024_at | HPD | 4-hydroxyphenylpyruvate dioxygenase | 66.75634 |
| 206030_at | ASPA | aspartoacylase (Canavan disease) | 133.0014 |
| 206076_at | LRRC23 | leucine rich repeat containing 23 | 249.2779 |
| 206083_at | BAI1 | brain-specific angiogenesis inhibitor 1 | 182.2871 |
| 206106_at | AL022328 | mitogen-activated protein kinase 12 | 201.0223 |
| 206115_at | EGR3 | early growth response 3 | 299.3629 |
| 206122_at | SOX15 | SRY (sex determining region Y)-box 15 | 198.5761 |
| 206136_at | FZD5 | frizzled homolog 5 (Drosophila) | 156.055 |
| 206200_s_at | ANXA11 | annexin A11 | 3927.669 |
| 206216_at | SRPK3 | SFRS protein kinase 3 | 294.5743 |
| 206258_at | ST8SIA5 | ST8 alpha-N-acetyl-neuraminide alpha-2,8-sialyltransferase 5 | 156.8497 |
| 206264_at | GPLD1 | glycosylphosphatidylinositol specific phospholipase D1 | 151.2241 |
| 206265_s_at | GPLD1 | glycosylphosphatidylinositol specific phospholipase D1 | 34.65381 |
| 206266_at | GPLD1 | glycosylphosphatidylinositol specific phospholipase D1 | 132.5488 |
| 206267_s_at | MATK | megakaryocyte-associated tyrosine kinase | 152.5021 |
| 206297_at | CTRC | chymotrypsin C (caldecrin) | 192.3934 |
| 206298_at | ARHGAP22 | Rho GTPase activating protein 22 | 89.79229 |
| 206315_at | CRLF1 | cytokine receptor-like factor 1 | 145.3569 |
| 206329_at | EXTL1 | exostoses (multiple)-like 1 | 203.309 |
| 206350_at | APCS | amyloid P component, serum | 109.1352 |
| 206380_s_at | CFP | complement factor properdin | 4182.763 |
| 206516_at | AMH | anti-Mullerian hormone | 177.1169 |
| 206518_s_at | RGS9 | regulator of G-protein signaling 9 | 40.34113 |
| 206525_at | GABRR1 | gamma-aminobutyric acid (GABA) receptor, rho 1 | 40.58551 |
| 206542_s_at | SMARCA2 | SWI/SNF related, matrix associated, actin dependent regulator of chromatin, subfamily a, member 2 | 188.4157 |
| 206543_at | SMARCA2 | SWI/SNF related, matrix associated, actin dependent regulator of chromatin, subfamily a, member 2 | 36.37033 |
| 206544_x_at | SMARCA2 | SWI/SNF related, matrix associated, actin dependent regulator of chromatin, subfamily a, member 2 | 194.7805 |
| 206634_at | SIX3 | SIX homeobox 3 | 84.27635 |
| 206678_at | GABRA1 | gamma-aminobutyric acid (GABA) A receptor, alpha 1 | 159.9261 |
| 206702_at | TEK | TEK tyrosine kinase, endothelial | 45.51731 |
| 206709_x_at | GPT | glutamic-pyruvate transaminase (alanine aminotransferase) | 185.1936 |
| 206728_at | ECE2 | endothelin converting enzyme 2 | 357.1924 |
| 206731_at | CNKSR2 | connector enhancer of kinase suppressor of Ras 2 | 75.83916 |
| 206868_at | STARD8 | StAR-related lipid transfer (START) domain containing 8 | 325.0745 |
| 206879_s_at | NRG2 | neuregulin 2 | 44.73169 |
| 206880_at | P2RX6 | purinergic receptor P2X, ligand-gated ion channel, 6 | 286.0077 |
| 206908_s_at | CLDN11 | claudin 11 | 286.5842 |
| 206929_s_at | NFIC | nuclear factor I/C (CCAAT-binding transcription factor) | 243.1791 |
| 206970_at | CNTN2 | contactin 2 (axonal) | 289.0107 |
| 206995_x_at | SCARF1 | scavenger receptor class F, member 1 | 26.6704 |
| 206996_x_at | CACNB1 | calcium channel, voltage-dependent, beta 1 subunit | 180.9158 |
| 207026_s_at | ATP2B3 | ATPase, Ca++ transporting, plasma membrane 3 | 187.1182 |
| 207047_s_at | CLCNKA | chloride channel Ka | 127.8809 |
| 207080_s_at | PYY | peptide YY | 52.08557 |
| 207082_at | CSF1 | colony stimulating factor 1 (macrophage) | 339.1984 |
| 207100_s_at | VAMP1 | vesicle-associated membrane protein 1 (synaptobrevin 1) | 268.6062 |
| 207101_at | VAMP1 | vesicle-associated membrane protein 1 (synaptobrevin 1) | 79.69501 |
| 207106_s_at | LTK | leukocyte receptor tyrosine kinase | 172.132 |
| 207130_at | NM_018634 | zinc finger, MYND-type containing 8 | 26.62284 |
| 207141_s_at | U39196 | potassium inwardly-rectifying channel, subfamily J, member 3 | 131.2588 |
| 207142_at | KCNJ3 | potassium inwardly-rectifying channel, subfamily J, member 3 | 40.70596 |
| 207175_at | ADIPOQ | adiponectin, C1Q and collagen domain containing | 36.37033 |
| 207204_at | FSCN2 | fascin homolog 2, actin-bundling protein, retinal (Strongylocentrotus purpuratus) | 40.91978 |
| 207233_s_at | MITF | microphthalmia-associated transcription factor | 171.5915 |
| 207308_at | SLCO1A2 | solute carrier organic anion transporter family, member 1A2 | 66.53728 |
| 207366_at | KCNS1 | potassium voltage-gated channel, delayed-rectifier, subfamily S, member 1 | 329.3312 |
| 207413_s_at | SCN5A | sodium channel, voltage-gated, type V, alpha subunit | 199.0545 |
| 207498_s_at | CYP2D6 | cytochrome P450, family 2, subfamily D, polypeptide 6 | 249.2411 |
| 207540_s_at | SYK | spleen tyrosine kinase | 783.9957 |
| 207556_s_at | DGKZ | diacylglycerol kinase, zeta 104kDa | 506.6213 |
| 207600_at | KCNC3 | potassium voltage-gated channel, Shaw-related subfamily, member 3 | 186.985 |
| 207613_s_at | CAMK2A | calcium/calmodulin-dependent protein kinase II alpha | 123.27 |
| 207655_s_at | BLNK | B-cell linker | 53.80666 |
| 207659_s_at | NM_006501 | myelin-associated oligodendrocyte basic protein | 155.3257 |
| 207678_s_at | SOX30 | SRY (sex determining region Y)-box 30 | 43.26195 |
| 207699_at | NM_014894 | zinc finger homeobox 2 | 250.9078 |
| 207717_s_at | PKP2 | plakophilin 2 | 26.81046 |
| 207761_s_at | METTL7A | methyltransferase like 7A | 1360.328 |
| 207767_s_at | EGR4 | early growth response 4 | 308.1555 |
| 207768_at | EGR4 | early growth response 4 | 33.10098 |
| 207777_s_at | SP140 | SP140 nuclear body protein | 90.78864 |
| 207832_at | BAIAP2 | BAI1-associated protein 2 | 188.896 |
| 207844_at | IL13 | interleukin 13 | 276.1394 |
| 207847_s_at | MUC1 | mucin 1, cell surface associated | 286.3414 |
| 207880_at | ADAM11 | ADAM metallopeptidase domain 11 | 396.6743 |
| 207961_x_at | MYH11 | myosin, heavy chain 11, smooth muscle | 207.1308 |
| 208012_x_at | SP110 | SP110 nuclear body protein | 1105.902 |
| 208031_s_at | RFX2 | regulatory factor X, 2 (influences HLA class II expression) | 323.8394 |
| 208062_s_at | NM_013984 | neuregulin 2 | 78.92231 |
| 208173_at | IFNB1 | interferon, beta 1, fibroblast | 138.1577 |
| 208174_x_at | ZRSR2 | zinc finger (CCCH type), RNA-binding motif and serine/arginine rich 2 | 509.8995 |
| 208208_at | MYH13 | myosin, heavy chain 13, skeletal muscle | 100.6896 |
| 208299_at | CACNA1I | calcium channel, voltage-dependent, T type, alpha 1I subunit | 245.5506 |
| 208354_s_at | SLC12A3 | solute carrier family 12 (sodium/chloride transporters), member 3 | 270.5077 |
| 208371_s_at | RING1 | ring finger protein 1 | 728.7746 |
| 208392_x_at | SP110 | SP110 nuclear body protein | 334.7421 |
| 208417_at | FGF6 | fibroblast growth factor 6 | 299.815 |
| 208457_at | GABRD | gamma-aminobutyric acid (GABA) A receptor, delta | 238.1745 |
| 208477_at | KCNC1 | potassium voltage-gated channel, Shaw-related subfamily, member 1 | 212.7752 |
| 208491_s_at | PGM5 | phosphoglucomutase 5 | 267.5743 |
| 208495_at | TLX3 | T-cell leukemia homeobox 3 | 126.3781 |
| 208518_s_at | NM_003894 | period homolog 2 (Drosophila) | 241.2446 |
| 208552_at | GRIK4 | glutamate receptor, ionotropic, kainate 4 | 86.04691 |
| 208605_s_at | NTRK1 | neurotrophic tyrosine kinase, receptor, type 1 | 76.43745 |
| 208812_x_at | HLA-C | major histocompatibility complex, class I, C; major histocompatibility complex, class I, B | 21045.38 |
| 208870_x_at | ATP5C1 | ATP synthase, H+ transporting, mitochondrial F1 complex, gamma polypeptide 1 | 4021.435 |
| 208906_at | BSCL2 | Bernardinelli-Seip congenital lipodystrophy 2 (seipin) | 473.042 |
| 208999_at | Sept8 | septin 8 | 107.405 |
| 209000_s_at | Sept8 | septin 8 | 160.7568 |
| 209018_s_at | PINK1 | PTEN induced putative kinase 1 | 806.7411 |
| 209019_s_at | PINK1 | PTEN induced putative kinase 1 | 366.8121 |
| 209048_s_at | ZMYND8 | zinc finger, MYND-type containing 8 | 122.8901 |
| 209049_s_at | BC001004 | zinc finger, MYND-type containing 8 | 72.31922 |
| 209113_s_at | HMG20B | high-mobility group 20B | 274.2936 |
| 209117_at | U79458 | WW domain binding protein 2 | 887.6486 |
| 209166_s_at | MAN2B1 | mannosidase, alpha, class 2B, member 1 | 3105.516 |
| 209209_s_at | FERMT2 | fermitin family homolog 2 (Drosophila) | 33.69906 |
| 209210_s_at | FERMT2 | fermitin family homolog 2 (Drosophila) | 44.14287 |
| 209241_x_at | MINK1 | misshapen-like kinase 1 (zebrafish) | 590.5412 |
| 209269_s_at | AW450910 | spleen tyrosine kinase | 310.4417 |
| 209275_s_at | CLN3 | ceroid-lipofuscinosis, neuronal 3 | 1250.029 |
| 209301_at | CA2 | carbonic anhydrase II | 271.5133 |
| 209383_at | DDIT3 | DNA-damage-inducible transcript 3 | 1174.598 |
| 209392_at | ENPP2 | ectonucleotide pyrophosphatase/phosphodiesterase 2 | 69.29631 |
| 209395_at | CHI3L1 | chitinase 3-like 1 (cartilage glycoprotein-39) | 183.1434 |
| 209396_s_at | CHI3L1 | chitinase 3-like 1 (cartilage glycoprotein-39) | 208.449 |
| 209447_at | SYNE1 | spectrin repeat containing, nuclear envelope 1 | 175.2754 |
| 209479_at | CCDC28A | coiled-coil domain containing 28A | 655.479 |
| 209499_x_at | TNFSF13 | TNFSF12-TNFSF13 readthrough transcript; tumor necrosis factor (ligand) superfamily, member 12; tumor necrosis factor (ligand) superfamily, member 13 | 2009.624 |
| 209500_x_at | TNFSF13 | TNFSF12-TNFSF13 readthrough transcript; tumor necrosis factor (ligand) superfamily, member 12; tumor necrosis factor (ligand) superfamily, member 13 | 4012.218 |
| 209502_s_at | BAIAP2 | BAI1-associated protein 2 | 363.7283 |
| 209528_s_at | TELO2 | TEL2, telomere maintenance 2, homolog (S. cerevisiae) | 145.853 |
| 209536_s_at | AF320070 | EH-domain containing 4 | 2795.188 |
| 209619_at | CD74 | CD74 molecule, major histocompatibility complex, class II invariant chain | 19329.63 |
| 209686_at | S100B | S100 calcium binding protein B | 77.58037 |
| 209703_x_at | METTL7A | methyltransferase like 7A | 198.9193 |
| 209716_at | CSF1 | colony stimulating factor 1 (macrophage) | 318.8484 |
| 209721_s_at | IFFO | intermediate filament family orphan 1 | 925.7957 |
| 209726_at | CA11 | carbonic anhydrase XI | 222.2746 |
| 209735_at | ABCG2 | ATP-binding cassette, sub-family G (WHITE), member 2 | 171.2076 |
| 209761_s_at | SP110 | SP110 nuclear body protein | 521.9575 |
| 209762_x_at | SP110 | SP110 nuclear body protein | 1145.031 |
| 209782_s_at | DBP | D site of albumin promoter (albumin D-box) binding protein | 330.1432 |
| 209783_at | DBP | D site of albumin promoter (albumin D-box) binding protein | 226.6079 |
| 209851_at | ZC3H13 | zinc finger CCCH-type containing 13 | 302.3786 |
| 209898_x_at | ITSN2 | intersectin 2 | 894.3523 |
| 209907_s_at | ITSN2 | intersectin 2 | 542.4441 |
| 209999_x_at | SOCS1 | suppressor of cytokine signaling 1 | 111.691 |
| 210000_s_at | SOCS1 | suppressor of cytokine signaling 1 | 33.61308 |
| 210001_s_at | SOCS1 | suppressor of cytokine signaling 1 | 126.7253 |
| 210002_at | GATA6 | GATA binding protein 6 | 31.18379 |
| 210051_at | RAPGEF3 | Rap guanine nucleotide exchange factor (GEF) 3 | 236.1864 |
| 210090_at | ARC | activity-regulated cytoskeleton-associated protein | 132.282 |
| 210099_at | ABCA2 | ATP-binding cassette, sub-family A (ABC1), member 2 | 96.9347 |
| 210100_s_at | ABCA2 | ATP-binding cassette, sub-family A (ABC1), member 2 | 171.158 |
| 210114_at | INVS | inversin | 120.7799 |
| 210129_s_at | TTLL3 | tubulin tyrosine ligase-like family, member 3; actin related protein 2/3 complex, subunit 4, 20kDa | 175.8064 |
| 210182_at | CORT | cortistatin; apoptosis-inducing, TAF9-like domain 1 | 302.9078 |
| 210185_at | CACNB1 | calcium channel, voltage-dependent, beta 1 subunit | 228.5472 |
| 210193_at | MOBP | myelin-associated oligodendrocyte basic protein | 39.7817 |
| 210197_at | ITPK1 | inositol 1,3,4-triphosphate 5/6 kinase | 212.5974 |
| 210198_s_at | PLP1 | proteolipid protein 1 | 60.95965 |
| 210201_x_at | BIN1 | bridging integrator 1 | 852.6572 |
| 210202_s_at | BIN1 | bridging integrator 1 | 271.8122 |
| 210226_at | NR4A1 | nuclear receptor subfamily 4, group A, member 1 | 755.7301 |
| 210280_at | MPZ | myelin protein zero | 334.4078 |
| 210314_x_at | TNFSF13 | TNFSF12-TNFSF13 readthrough transcript; tumor necrosis factor (ligand) superfamily, member 12; tumor necrosis factor (ligand) superfamily, member 13 | 3870.858 |
| 210336_x_at | MZF1 | myeloid zinc finger 1 | 294.5666 |
| 210357_s_at | SMOX | spermine oxidase | 1508.893 |
| 210400_at | GRIN2C | glutamate receptor, ionotropic, N-methyl D-aspartate 2C | 258.5744 |
| 210401_at | P2RX1 | purinergic receptor P2X, ligand-gated ion channel, 1 | 861.0468 |
| 210501_x_at | AF119846 | eukaryotic translation initiation factor 3, subunit K | 7585.011 |
| 210545_at | ITSN2 | intersectin 2 | 264.2202 |
| 210557_x_at | CSF1 | colony stimulating factor 1 (macrophage) | 261.2855 |
| 210577_at | CASR | calcium-sensing receptor | 232.9241 |
| 210593_at | SAT1 | KIAA0913 | 70.05613 |
| 210614_at | TTPA | tocopherol (alpha) transfer protein | 128.0292 |
| 210632_s_at | SGCA | sarcoglycan, alpha (50kDa dystrophin-associated glycoprotein) | 64.51871 |
| 210719_s_at | HMG20B | high-mobility group 20B | 558.2614 |
| 210735_s_at | CA12 | carbonic anhydrase XII | 217.9514 |
| 210740_s_at | ITPK1 | inositol 1,3,4-triphosphate 5/6 kinase | 2110.004 |
| 210745_at | ONECUT1 | one cut homeobox 1 | 133.617 |
| 210828_s_at | ARNT | aryl hydrocarbon receptor nuclear translocator | 153.0395 |
| 210838_s_at | ACVRL1 | activin A receptor type II-like 1 | 217.2414 |
| 210839_s_at | ENPP2 | ectonucleotide pyrophosphatase/phosphodiesterase 2 | 159.4838 |
| 210852_s_at | AASS | aminoadipate-semialdehyde synthase | 59.03951 |
| 210859_x_at | CLN3 | ceroid-lipofuscinosis, neuronal 3 | 782.5459 |
| 210942_s_at | ST3GAL6 | ST3 beta-galactoside alpha-2,3-sialyltransferase 6 | 71.87261 |
| 210967_x_at | CACNB1 | calcium channel, voltage-dependent, beta 1 subunit | 160.6761 |
| 210977_s_at | HSF4 | heat shock transcription factor 4 | 334.372 |
| 211003_x_at | TGM2 | transglutaminase 2 (C polypeptide, protein-glutamine-gamma-glutamyltransferase) | 160.9349 |
| 211004_s_at | ALDH3B1 | aldehyde dehydrogenase 3 family, member B1 | 470.4542 |
| 211054_at | INVS | inversin | 26.23239 |
| 211055_s_at | INVS | inversin | 33.18355 |
| 211143_x_at | NR4A1 | nuclear receptor subfamily 4, group A, member 1 | 419.0184 |
| 211146_at | U96291 | major histocompatibility complex, class I, C; major histocompatibility complex, class I, B | 98.94535 |
| 211147_s_at | P2RX6 | purinergic receptor P2X, ligand-gated ion channel, 6 | 182.5814 |
| 211178_s_at | PSTPIP1 | proline-serine-threonine phosphatase interacting protein 1 | 748.9413 |
| 211253_x_at | PYY | peptide YY | 197.9096 |
| 211272_s_at | DGKA | diacylglycerol kinase, alpha 80kDa | 147.9015 |
| 211323_s_at | ITPR1 | inositol 1,4,5-triphosphate receptor, type 1 | 198.6539 |
| 211384_s_at | CASR | calcium-sensing receptor | 148.1532 |
| 211390_at | N4BP2L1 | NEDD4 binding protein 2-like 1 | 69.36003 |
| 211424_x_at | METTL7A | methyltransferase like 7A | 46.47058 |
| 211480_s_at | SLCO1A2 | solute carrier organic anion transporter family, member 1A2 | 51.92316 |
| 211481_at | SLCO1A2 | solute carrier organic anion transporter family, member 1A2 | 320.835 |
| 211495_x_at | TNFSF13 | TNFSF12-TNFSF13 readthrough transcript; tumor necrosis factor (ligand) superfamily, member 12; tumor necrosis factor (ligand) superfamily, member 13 | 1737.374 |
| 211573_x_at | TGM2 | transglutaminase 2 (C polypeptide, protein-glutamine-gamma-glutamyltransferase) | 132.2841 |
| 211672_s_at | ARPC4 | tubulin tyrosine ligase-like family, member 3; actin related protein 2/3 complex, subunit 4, 20kDa | 474.3238 |
| 211695_x_at | MUC1 | mucin 1, cell surface associated | 160.962 |
| 211727_s_at | COX11 | COX11 homolog, cytochrome c oxidase assembly protein (yeast) | 302.1884 |
| 211799_x_at | HLA-C | major histocompatibility complex, class I, C; major histocompatibility complex, class I, B | 10286.95 |
| 211823_s_at | PXN | paxillin | 153.2999 |
| 211830_s_at | CACNA1I | calcium channel, voltage-dependent, T type, alpha 1I subunit | 228.0196 |
| 211836_s_at | MOG | myelin oligodendrocyte glycoprotein | 57.33292 |
| 211839_s_at | CSF1 | colony stimulating factor 1 (macrophage) | 194.0104 |
| 211843_x_at | CYP3A7 | cytochrome P450, family 3, subfamily A, polypeptide 7 | 71.90734 |
| 211950_at | UBR4 | ubiquitin protein ligase E3 component n-recognin 4 | 1051.738 |
| 212058_at | SR140 | U2-associated SR140 protein | 993.5567 |
| 212060_at | SR140 | U2-associated SR140 protein | 317.8403 |
| 212061_at | SR140 | U2-associated SR140 protein | 367.5147 |
| 212076_at | MLL | myeloid/lymphoid or mixed-lineage leukemia (trithorax homolog, Drosophila) | 226.1339 |
| 212078_s_at | MLL | myeloid/lymphoid or mixed-lineage leukemia (trithorax homolog, Drosophila) | 314.4078 |
| 212079_s_at | AA715041 | myeloid/lymphoid or mixed-lineage leukemia (trithorax homolog, Drosophila) | 108.8078 |
| 212080_at | MLL | myeloid/lymphoid or mixed-lineage leukemia (trithorax homolog, Drosophila) | 304.0945 |
| 212086_x_at | LMNA | lamin A/C | 1533.709 |
| 212089_at | LMNA | lamin A/C | 1045.281 |
| 212248_at | AI886796 | metadherin | 645.1724 |
| 212250_at | MTDH | metadherin | 1150.858 |
| 212251_at | MTDH | metadherin | 1306.36 |
| 212257_s_at | SMARCA2 | SWI/SNF related, matrix associated, actin dependent regulator of chromatin, subfamily a, member 2 | 161.3948 |
| 212258_s_at | SMARCA2 | SWI/SNF related, matrix associated, actin dependent regulator of chromatin, subfamily a, member 2 | 231.4559 |
| 212350_at | TBC1D1 | TBC1 (tre-2/USP6, BUB2, cdc16) domain family, member 1 | 1019.593 |
| 212359_s_at | KIAA0913 | KIAA0913 | 598.7093 |
| 212402_at | ZC3H13 | zinc finger CCCH-type containing 13 | 142.7459 |
| 212485_at | GPATCH8 | G patch domain containing 8 | 910.908 |
| 212487_at | GPATCH8 | G patch domain containing 8 | 375.2117 |
| 212527_at | FAM152B | PPPDE peptidase domain containing 2 | 611.8462 |
| 212591_at | RBM34 | RNA binding motif protein 34 | 735.7037 |
| 212716_s_at | EIF3K | eukaryotic translation initiation factor 3, subunit K | 3950.128 |
| 212772_s_at | ABCA2 | ATP-binding cassette, sub-family A (ABC1), member 2 | 308.6718 |
| 212821_at | SPTB | pleckstrin homology domain containing, family G (with RhoGef domain) member 3 | 136.1874 |
| 212823_s_at | PLEKHG3 | pleckstrin homology domain containing, family G (with RhoGef domain) member 3 | 476.7527 |
| 212841_s_at | PPFIBP2 | PTPRF interacting protein, binding protein 2 (liprin beta 2) | 230.9403 |
| 212845_at | SAMD4A | sterile alpha motif domain containing 4A | 204.2266 |
| 212859_x_at | MT1E | metallothionein 1L (gene/pseudogene); metallothionein 1E; metallothionein 1 pseudogene 3; metallothionein 1J (pseudogene) | 859.8886 |
| 213009_s_at | TRIM37 | tripartite motif-containing 37 | 315.8504 |
| 213015_at | BF448315 | bobby sox homolog (Drosophila) | 246.3534 |
| 213016_at | AA573805 | bobby sox homolog (Drosophila) | 110.053 |
| 213017_at | AL534702 | abhydrolase domain containing 3 | 277.7739 |
| 213024_at | TMF1 | TATA element modulatory factor 1 | 224.5978 |
| 213035_at | ANKRD28 | ankyrin repeat domain 28 | 353.6436 |
| 213045_at | MAST3 | microtubule associated serine/threonine kinase 3 | 247.9611 |
| 213049_at | GARNL1 | GTPase activating Rap/RanGAP domain-like 1 | 158.4813 |
| 213108_at | CAMK2A | calcium/calmodulin-dependent protein kinase II alpha | 410.6623 |
| 213121_at | SNRP70 | small nuclear ribonucleoprotein 70kDa (U1) | 182.211 |
| 213134_x_at | BTG3 | BTG family, member 3 | 1149.45 |
| 213155_at | WSCD1 | WSC domain containing 1 | 276.771 |
| 213157_s_at | WSCD1 | WSC domain containing 1 | 231.4996 |
| 213201_s_at | TNNT1 | troponin T type 1 (skeletal, slow) | 344.909 |
| 213267_at | DOPEY1 | dopey family member 1 | 27.80304 |
| 213271_s_at | DOPEY1 | dopey family member 1 | 75.32857 |
| 213298_at | NFIC | nuclear factor I/C (CCAAT-binding transcription factor) | 121.4202 |
| 213299_at | ZBTB7A | zinc finger and BTB domain containing 7A | 76.06312 |
| 213303_x_at | ZBTB7A | zinc finger and BTB domain containing 7A | 73.29352 |
| 213326_at | VAMP1 | vesicle-associated membrane protein 1 (synaptobrevin 1) | 463.2349 |
| 213335_s_at | ST3GAL6 | ST3 beta-galactoside alpha-2,3-sialyltransferase 6 | 45.93985 |
| 213337_s_at | AA877218 | suppressor of cytokine signaling 1 | 220.4263 |
| 213355_at | ST3GAL6 | ST3 beta-galactoside alpha-2,3-sialyltransferase 6 | 63.45264 |
| 213366_x_at | ATP5C1 | ATP synthase, H+ transporting, mitochondrial F1 complex, gamma polypeptide 1 | 2954.06 |
| 213375_s_at | N4BP2L1 | NEDD4 binding protein 2-like 1 | 370.0106 |
| 213397_x_at | ANG | ribonuclease, RNase A family, 4 | 167.0747 |
| 213438_at | NFASC | neurofascin homolog (chicken) | 86.67507 |
| 213471_at | NPHP4 | nephronophthisis 4 | 157.3792 |
| 213538_at | SON | SON DNA binding protein | 2117.54 |
| 213553_x_at | APOC1 | apolipoprotein C-I | 523.5743 |
| 213597_s_at | CTDSPL | CTD (carboxy-terminal domain, RNA polymerase II, polypeptide A) small phosphatase-like | 54.30268 |
| 213604_at | AW451236 | transcription elongation factor B (SIII), polypeptide 3 (110kDa, elongin A) | 541.3098 |
| 213629_x_at | MT1F | metallothionein 1F | 1069.243 |
| 213633_at | SH3BP1 | SH3-domain binding protein 1 | 599.771 |
| 213693_s_at | MUC1 | mucin 1, cell surface associated | 184.302 |
| 213706_at | GPD1 | glycerol-3-phosphate dehydrogenase 1 (soluble) | 279.0734 |
| 213724_s_at | PDK2 | pyruvate dehydrogenase kinase, isozyme 2 | 328.4773 |
| 213742_at | SFRS11 | splicing factor, arginine/serine-rich 11 | 88.92763 |
| 213824_at | OLIG2 | oligodendrocyte lineage transcription factor 2 | 104.3162 |
| 213825_at | OLIG2 | oligodendrocyte lineage transcription factor 2 | 94.40284 |
| 213836_s_at | WIPI1 | WD repeat domain, phosphoinositide interacting 1 | 436.4778 |
| 213870_at | HLA-DPA3 | collagen, type XI, alpha 2 | 312.8437 |
| 213876_x_at | ZRSR2 | zinc finger (CCCH type), RNA-binding motif and serine/arginine rich 2 | 526.7953 |
| 213909_at | LRRC15 | leucine rich repeat containing 15 | 262.9273 |
| 213934_s_at | ZNF23 | zinc finger protein 23 (KOX 16) | 44.48272 |
| 213966_at | HMG20B | high-mobility group 20B | 75.19169 |
| 213982_s_at | RABGAP1L | RAB GTPase activating protein 1-like | 443.1639 |
| 214013_s_at | TBC1D1 | TBC1 (tre-2/USP6, BUB2, cdc16) domain family, member 1 | 127.5394 |
| 214040_s_at | GSN | gelsolin (amyloidosis, Finnish type) | 145.1066 |
| 214132_at | ATP5C1 | ATP synthase, H+ transporting, mitochondrial F1 complex, gamma polypeptide 1 | 148.4888 |
| 214147_at | C1orf175 | chromosome 1 open reading frame 175 | 294.3522 |
| 214151_s_at | PIGB | cell cycle progression 1 | 311.4233 |
| 214152_at | PIGB | cell cycle progression 1 | 203.4767 |
| 214154_s_at | PKP2 | plakophilin 2 | 94.16176 |
| 214156_at | MYRIP | myosin VIIA and Rab interacting protein | 21.42654 |
| 214164_x_at | CA12 | carbonic anhydrase XII | 167.841 |
| 214187_x_at | CTDSPL | CTD (carboxy-terminal domain, RNA polymerase II, polypeptide A) small phosphatase-like | 143.9689 |
| 214203_s_at | PRODH | proline dehydrogenase (oxidase) 1 | 319.2205 |
| 214212_x_at | FERMT2 | fermitin family homolog 2 (Drosophila) | 45.5044 |
| 214213_x_at | LMNA | lamin A/C | 852.211 |
| 214242_at | MAN1A2 | mannosidase, alpha, class 1A, member 2 | 209.2446 |
| 214246_x_at | AI859060 | misshapen-like kinase 1 (zebrafish) | 2139.314 |
| 214250_at | NUMA1 | nuclear mitotic apparatus protein 1 | 42.62734 |
| 214251_s_at | NUMA1 | nuclear mitotic apparatus protein 1 | 114.634 |
| 214253_s_at | DTNB | dystrobrevin, beta | 153.1141 |
| 214270_s_at | MAPRE3 | microtubule-associated protein, RP/EB family, member 3 | 89.45937 |
| 214277_at | COX11 | COX11 homolog, cytochrome c oxidase assembly protein (yeast) | 20.3728 |
| 214290_s_at | HIST2H2AA3 | histone cluster 2, H2aa3; histone cluster 2, H2aa4 | 3522.975 |
| 214313_s_at | BE138647 | eukaryotic translation initiation factor 5B | 31.31647 |
| 214314_s_at | BE138647 | eukaryotic translation initiation factor 5B | 24.02899 |
| 214400_at | INSL3 | insulin-like 3 (Leydig cell) | 92.3269 |
| 214425_at | AMBP | alpha-1-microglobulin/bikunin precursor | 117.8657 |
| 214439_x_at | BIN1 | bridging integrator 1 | 773.0986 |
| 214459_x_at | HLA-C | major histocompatibility complex, class I, C; major histocompatibility complex, class I, B | 19339.29 |
| 214491_at | SSTR3 | somatostatin receptor 3 | 290.3133 |
| 214493_s_at | NM_005799 | InaD-like (Drosophila) | 43.88594 |
| 214572_s_at | INSL3 | insulin-like 3 (Leydig cell) | 191.5775 |
| 214625_s_at | MINK1 | misshapen-like kinase 1 (zebrafish) | 419.8391 |
| 214643_x_at | BIN1 | bridging integrator 1 | 544.9803 |
| 214650_x_at | MOG | myelin oligodendrocyte glycoprotein | 84.95395 |
| 214692_s_at | JRK | jerky homolog (mouse) | 387.4293 |
| 214705_at | INADL | InaD-like (Drosophila) | 21.69996 |
| 214783_s_at | ANXA11 | annexin A11 | 574.3424 |
| 214791_at | LOC93349 | SP140 nuclear body protein-like | 255.8736 |
| 214795_at | ZMYND8 | zinc finger, MYND-type containing 8 | 253.1007 |
| 214799_at | NFASC | neurofascin homolog (chicken) | 174.4549 |
| 214817_at | BE783668 | unc-13 homolog A (C. elegans) | 119.4212 |
| 214829_at | AASS | aminoadipate-semialdehyde synthase | 22.75876 |
| 214855_s_at | GARNL1 | GTPase activating Rap/RanGAP domain-like 1 | 146.9264 |
| 214859_at | FSTL4 | follistatin-like 4 | 218.8414 |
| 214886_s_at | N4BP2L1 | NEDD4 binding protein 2-like 1 | 115.6685 |
| 214887_at | N4BP2L1 | NEDD4 binding protein 2-like 1 | 28.71123 |
| 214906_x_at | N4BP2L1 | NEDD4 binding protein 2-like 1 | 574.1591 |
| 214915_at | ZNF362 | zinc finger protein 362 | 53.31522 |
| 214942_at | RBM34 | RNA binding motif protein 34 | 68.68157 |
| 214943_s_at | RBM34 | RNA binding motif protein 34 | 58.10618 |
| 214948_s_at | AL050136 | TATA element modulatory factor 1 | 449.3668 |
| 214988_s_at | SON | SON DNA binding protein | 554.753 |
| 214993_at | ASPHD1 | aspartate beta-hydroxylase domain containing 1 | 142.5335 |
| 215027_at | HSU79275 | Rap guanine nucleotide exchange factor (GEF) 3 | 430.4386 |
| 215032_at | RREB1 | ras responsive element binding protein 1 | 115.5708 |
| 215120_s_at | SAMD4A | sterile alpha motif domain containing 4A | 63.58134 |
| 215153_at | NOS1AP | nitric oxide synthase 1 (neuronal) adaptor protein | 526.2435 |
| 215155_at | HEXA | hexosaminidase A (alpha polypeptide) | 179.7883 |
| 215162_at | GARNL1 | GTPase activating Rap/RanGAP domain-like 1 | 87.85708 |
| 215231_at | AU144309 | protein kinase, AMP-activated, gamma 2 non-catalytic subunit | 24.5111 |
| 215274_at | SLC12A3 | solute carrier family 12 (sodium/chloride transporters), member 3 | 257.2776 |
| 215295_at | DTNB | dystrobrevin, beta | 109.6543 |
| 215342_s_at | RABGAP1L | RAB GTPase activating protein 1-like | 67.27667 |
| 215350_at | SYNE1 | spectrin repeat containing, nuclear envelope 1 | 37.02168 |
| 215425_at | BTG3 | BTG family, member 3 | 45.35685 |
| 215460_x_at | BRD1 | bromodomain containing 1 | 695.5542 |
| 215462_at | LOC149478 | polo-like kinase 3 (Drosophila) | 399.214 |
| 215471_s_at | MAP7 | microtubule-associated protein 7 | 240.1856 |
| 215495_s_at | SAMD4A | sterile alpha motif domain containing 4A | 184.0068 |
| 215496_at | SAMD4A | sterile alpha motif domain containing 4A | 186.4885 |
| 215554_at | GPLD1 | glycosylphosphatidylinositol specific phospholipase D1 | 29.68365 |
| 215620_at | AU147182 | ras responsive element binding protein 1 | 196.8084 |
| 215636_at | UBR4 | ubiquitin protein ligase E3 component n-recognin 4 | 51.46965 |
| 215689_s_at | SHBG | sex hormone-binding globulin | 281.9083 |
| 215741_x_at | AKAP8L | A kinase (PRKA) anchor protein 8-like | 192.1731 |
| 215766_at | GSTA1 | glutathione S-transferase alpha 1 | 322.0629 |
| 215809_at | CYP2D6 | cytochrome P450, family 2, subfamily D, polypeptide 6 | 376.241 |
| 215855_s_at | TMF1 | TATA element modulatory factor 1 | 114.9992 |
| 215867_x_at | AL050025 | carbonic anhydrase XII | 167.5979 |
| 215909_x_at | MINK1 | misshapen-like kinase 1 (zebrafish) | 551.2607 |
| 215911_x_at | ATP2B3 | ATPase, Ca++ transporting, plasma membrane 3 | 30.44404 |
| 215959_at | PPFIBP2 | PTPRF interacting protein, binding protein 2 (liprin beta 2) | 295.9177 |
| 215961_at | GRK6 | coagulation factor XII (Hageman factor) | 166.8599 |
| 216183_at | TGM2 | transglutaminase 2 (C polypeptide, protein-glutamine-gamma-glutamyltransferase) | 369.3426 |
| 216255_s_at | GRM8 | glutamate receptor, metabotropic 8 | 43.62615 |
| 216256_at | GRM8 | glutamate receptor, metabotropic 8 | 139.744 |
| 216309_x_at | JRK | jerky homolog (mouse) | 584.6078 |
| 216312_at | ATP2B3 | ATPase, Ca++ transporting, plasma membrane 3 | 36.85122 |
| 216336_x_at | RNF19B | metallothionein 1L (gene/pseudogene); metallothionein 1E; metallothionein 1 pseudogene 3; metallothionein 1J (pseudogene) | 439.8301 |
| 216344_at | NPHP4 | nephronophthisis 4 | 248.8778 |
| 216345_at | KIAA0913 | KIAA0913 | 134.3151 |
| 216526_x_at | HLA-C | major histocompatibility complex, class I, C; major histocompatibility complex, class I, B | 20450.09 |
| 216546_s_at | CHI3L1 | chitinase 3-like 1 (cartilage glycoprotein-39) | 319.0744 |
| 216617_s_at | MAG | myelin associated glycoprotein | 82.89206 |
| 216624_s_at | MLL | myeloid/lymphoid or mixed-lineage leukemia (trithorax homolog, Drosophila) | 127.2592 |
| 216663_s_at | ZMYND10 | zinc finger, MYND-type containing 10 | 137.8297 |
| 216724_at | DCLK2 | doublecortin-like kinase 2 | 117.8606 |
| 216725_at | DCLK2 | doublecortin-like kinase 2 | 91.35625 |
| 216944_s_at | ITPR1 | inositol 1,4,5-triphosphate receptor, type 1 | 107.4924 |
| 216992_s_at | GRM8 | glutamate receptor, metabotropic 8 | 42.08955 |
| 216993_s_at | COL11A2 | collagen, type XI, alpha 2 | 305.2702 |
| 217016_x_at | FLJ23172 | transmembrane protein 212 | 98.60168 |
| 217032_at | AL078621 | forkhead box D4-like 1 | 85.86955 |
| 217040_x_at | SOX15 | SRY (sex determining region Y)-box 15 | 368.8932 |
| 217044_s_at | PLEKHG3 | pleckstrin homology domain containing, family G (with RhoGef domain) member 3 | 330.8466 |
| 217074_at | SMOX | spermine oxidase | 138.7424 |
| 217146_at | JRK | jerky homolog (mouse) | 110.1667 |
| 217165_x_at | MT1F | metallothionein 1F | 1535.826 |
| 217184_s_at | LTK | leukocyte receptor tyrosine kinase | 230.5909 |
| 217197_x_at | N4BP2L1 | NEDD4 binding protein 2-like 1 | 464.7233 |
| 217265_at | LOC139061 | plasma membrane proteolipid (plasmolipin) | 84.71729 |
| 217282_at | MAN1A2 | mannosidase, alpha, class 1A, member 2 | 38.5425 |
| 217392_at | CAPZA1P | capping protein (actin filament) muscle Z-line, alpha 1 | 66.22082 |
| 217405_x_at | KIAA0319 | glycosylphosphatidylinositol specific phospholipase D1 | 169.9271 |
| 217411_s_at | RREB1 | ras responsive element binding protein 1 | 194.7124 |
| 217447_at | MAG | myelin associated glycoprotein | 88.92763 |
| 217456_x_at | HLA-E | major histocompatibility complex, class I, E | 6205.475 |
| 217468_at | CYP2D6 | cytochrome P450, family 2, subfamily D, polypeptide 6 | 197.5856 |
| 217573_at | GRIN2C | glutamate receptor, ionotropic, N-methyl D-aspartate 2C | 191.9716 |
| 217609_at | LRRC23 | leucine rich repeat containing 23 | 90.47443 |
| 217707_x_at | AI535683 | SWI/SNF related, matrix associated, actin dependent regulator of chromatin, subfamily a, member 2 | 352.0172 |
| 217710_x_at | ITPK1 | inositol 1,3,4-triphosphate 5/6 kinase | 214.429 |
| 217711_at | TEK | TEK tyrosine kinase, endothelial | 975.9078 |
| 217730_at | TMBIM1 | transmembrane BAX inhibitor motif containing 1 | 1510.205 |
| 217818_s_at | ARPC4 | tubulin tyrosine ligase-like family, member 3; actin related protein 2/3 complex, subunit 4, 20kDa | 523.5158 |
| 217858_s_at | ARMCX3 | armadillo repeat containing, X-linked 3 | 43.62602 |
| 217891_at | C16orf58 | chromosome 16 open reading frame 58 | 113.4313 |
| 217908_s_at | IQWD1 | IQ motif and WD repeats 1 | 181.7955 |
| 217920_at | MAN1A2 | mannosidase, alpha, class 1A, member 2 | 172.4063 |
| 217921_at | MAN1A2 | mannosidase, alpha, class 1A, member 2 | 60.15281 |
| 218028_at | ELOVL1 | elongation of very long chain fatty acids (FEN1/Elo2, SUR4/Elo3, yeast)-like 1 | 508.4736 |
| 218040_at | PRPF38B | PRP38 pre-mRNA processing factor 38 (yeast) domain containing B | 250.612 |
| 218064_s_at | AKAP8L | A kinase (PRKA) anchor protein 8-like | 250.1232 |
| 218067_s_at | ARGLU1 | arginine and glutamate rich 1 | 1519.923 |
| 218168_s_at | CABC1 | chaperone, ABC1 activity of bc1 complex homolog (S. pombe) | 1389.482 |
| 218216_x_at | ARL6IP4 | ADP-ribosylation-like factor 6 interacting protein 4 | 1296.99 |
| 218221_at | AL042842 | aryl hydrocarbon receptor nuclear translocator | 121.3707 |
| 218222_x_at | ARNT | aryl hydrocarbon receptor nuclear translocator | 363.5745 |
| 218260_at | DDA1 | DET1 and DDB1 associated 1 | 684.8677 |
| 218266_s_at | FREQ | frequenin homolog (Drosophila) | 149.9886 |
| 218269_at | RNASEN | ribonuclease type III, nuclear | 159.9129 |
| 218279_s_at | HIST2H2AA4 | histone cluster 2, H2aa3; histone cluster 2, H2aa4 | 119.3641 |
| 218280_x_at | HIST2H2AA3 | histone cluster 2, H2aa3; histone cluster 2, H2aa4 | 1554.253 |
| 218290_at | PLEKHJ1 | pleckstrin homology domain containing, family J member 1 | 779.6388 |
| 218292_s_at | PRKAG2 | protein kinase, AMP-activated, gamma 2 non-catalytic subunit | 492.9824 |
| 218309_at | CAMK2N1 | calcium/calmodulin-dependent protein kinase II inhibitor 1 | 132.6932 |
| 218317_x_at | GIYD2 | GIY-YIG domain containing 2; GIY-YIG domain containing 1 | 893.5059 |
| 218359_at | NRSN2 | neurensin 2 | 207.2419 |
| 218368_s_at | TNFRSF12A | tumor necrosis factor receptor superfamily, member 12A | 525.2932 |
| 218391_at | SNF8 | SNF8, ESCRT-II complex subunit, homolog (S. cerevisiae) | 960.3256 |
| 218394_at | ROGDI | rogdi homolog (Drosophila) | 980.8257 |
| 218396_at | VPS13C | vacuolar protein sorting 13 homolog C (S. cerevisiae) | 930.9155 |
| 218458_at | NM_022471 | germ cell-less homolog 1 (Drosophila)-like; germ cell-less homolog 1 (Drosophila) | 111.0079 |
| 218492_s_at | THAP7 | THAP domain containing 7 | 181.967 |
| 218676_s_at | PCTP | phosphatidylcholine transfer protein | 331.8411 |
| 218688_at | DAK | dihydroxyacetone kinase 2 homolog (S. cerevisiae) | 131.0106 |
| 218804_at | TMEM16A | anoctamin 1, calcium activated chloride channel | 22.89809 |
| 218819_at | INTS6 | integrator complex subunit 6 | 155.7327 |
| 218836_at | RPP21 | TRIM39-like protein | 455.24 |
| 218863_s_at | TNS1 | tensin 1 | 46.19594 |
| 218864_at | TNS1 | tensin 1 | 165.1544 |
| 218872_at | TESC | tescalcin | 1037.87 |
| 218928_s_at | SLC37A1 | solute carrier family 37 (glycerol-3-phosphate transporter), member 1 | 237.6745 |
| 218966_at | MYO5C | myosin VC | 269.5966 |
| 219051_x_at | METRN | meteorin, glial cell differentiation regulator | 215.9829 |
| 219066_at | PPCDC | phosphopantothenoylcysteine decarboxylase | 339.2411 |
| 219075_at | YIPF2 | Yip1 domain family, member 2 | 111.8013 |
| 219186_at | NM_020224 | zinc finger and BTB domain containing 7A | 230.5745 |
| 219191_s_at | BIN2 | bridging integrator 2 | 2086.734 |
| 219236_at | PAQR6 | progestin and adipoQ receptor family member VI | 398.9593 |
| 219241_x_at | SSH3 | slingshot homolog 3 (Drosophila) | 335.5743 |
| 219290_x_at | DAPP1 | dual adaptor of phosphotyrosine and 3-phosphoinositides | 234.3808 |
| 219305_x_at | FBXO2 | F-box protein 2 | 168.8307 |
| 219325_s_at | ELAC1 | elaC homolog 1 (E. coli) | 33.19436 |
| 219344_at | SLC29A3 | solute carrier family 29 (nucleoside transporters), member 3; Sp9 transcription factor homolog (mouse) | 536.0746 |
| 219352_at | HERC6 | hect domain and RLD 6 | 188.7092 |
| 219358_s_at | CENTA2 | ArfGAP with dual PH domains 2 | 898.2854 |
| 219400_at | CNTNAP1 | contactin associated protein 1 | 471.463 |
| 219401_at | XYLT2 | xylosyltransferase II | 146.2836 |
| 219411_at | ELMO3 | engulfment and cell motility 3 | 80.46659 |
| 219413_at | ACBD4 | acyl-Coenzyme A binding domain containing 4 | 316.5305 |
| 219415_at | TTYH1 | tweety homolog 1 (Drosophila) | 52.43624 |
| 219429_at | FA2H | fatty acid 2-hydroxylase | 58.5066 |
| 219431_at | ARHGAP10 | Rho GTPase activating protein 10 | 262.2857 |
| 219437_s_at | ANKRD11 | ankyrin repeat domain 11; hypothetical protein LOC100128265 | 122.8428 |
| 219455_at | NM_024788 | chromosome 7 open reading frame 63 | 189.0392 |
| 219550_at | ROBO3 | roundabout, axon guidance receptor, homolog 3 (Drosophila) | 230.7281 |
| 219564_at | KCNJ16 | potassium inwardly-rectifying channel, subfamily J, member 16 | 42.30695 |
| 219577_s_at | ABCA7 | ATP-binding cassette, sub-family A (ABC1), member 7 | 327.9078 |
| 219578_s_at | CPEB1 | cytoplasmic polyadenylation element binding protein 1 | 320.9052 |
| 219642_s_at | PEX5L | peroxisomal biogenesis factor 5-like | 105.8211 |
| 219644_at | CCDC41 | coiled-coil domain containing 41 | 39.03637 |
| 219707_at | CPNE7 | copine VII | 295.6985 |
| 219744_at | FN3K | fructosamine 3 kinase | 66.52458 |
| 219818_s_at | GPATCH1 | G patch domain containing 1 | 59.39184 |
| 219828_at | C9orf86 | chromosome 9 open reading frame 86 | 281.4935 |
| 219847_at | HDAC11 | histone deacetylase 11 | 499.6102 |
| 219878_s_at | KLF13 | Kruppel-like factor 13 | 215.3378 |
| 219882_at | TTLL7 | tubulin tyrosine ligase-like family, member 7 | 82.34719 |
| 219916_s_at | RNF39 | ring finger protein 39 | 534.0526 |
| 219919_s_at | NM_018276 | slingshot homolog 3 (Drosophila) | 273.0078 |
| 219999_at | MAN2A2 | mannosidase, alpha, class 2A, member 2 | 506.8785 |
| 220003_at | LRRC36 | leucine rich repeat containing 36 | 254.1422 |
| 220061_at | ACSM5 | acyl-CoA synthetase medium-chain family member 5 | 314.3412 |
| 220072_at | CSPP1 | centrosome and spindle pole associated protein 1 | 71.04288 |
| 220102_at | FOXL2 | forkhead box L2 | 80.40653 |
| 220125_at | DNAI1 | dynein, axonemal, intermediate chain 1 | 201.7709 |
| 220142_at | HAPLN2 | hyaluronan and proteoglycan link protein 2 | 96.36272 |
| 220168_at | CASC1 | cancer susceptibility candidate 1 | 29.80551 |
| 220182_at | SLC25A23 | solute carrier family 25 (mitochondrial carrier; phosphate carrier), member 23 | 108.1901 |
| 220185_at | SPTBN4 | spectrin, beta, non-erythrocytic 4 | 269.1744 |
| 220230_s_at | CYB5R2 | cytochrome b5 reductase 2 | 223.8367 |
| 220259_at | PLEKHH3 | pleckstrin homology domain containing, family H (with MyTH4 domain) member 3 | 42.77019 |
| 220305_at | NM_024030 | mitochondrial antiviral signaling protein | 120.3016 |
| 220331_at | CYP46A1 | cytochrome P450, family 46, subfamily A, polypeptide 1 | 203.8422 |
| 220351_at | CCRL1 | chemokine (C-C motif) receptor-like 1 | 37.27412 |
| 220434_at | ADCK4 | aarF domain containing kinase 4 | 84.47099 |
| 220443_s_at | VAX2 | ventral anterior homeobox 2 | 166.6328 |
| 220447_at | HRH3 | histamine receptor H3 | 167.2279 |
| 220488_s_at | BCAS3 | breast carcinoma amplified sequence 3 | 278.341 |
| 220517_at | VPS13C | vacuolar protein sorting 13 homolog C (S. cerevisiae) | 101.6377 |
| 220546_at | NM_024891 | myeloid/lymphoid or mixed-lineage leukemia (trithorax homolog, Drosophila) | 52.99879 |
| 220589_s_at | ITFG2 | integrin alpha FG-GAP repeat containing 2 | 138.472 |
| 220590_at | ITFG2 | integrin alpha FG-GAP repeat containing 2 | 93.07399 |
| 220596_at | GPATCH4 | G patch domain containing 4 | 187.391 |
| 220597_s_at | ARL6IP4 | ADP-ribosylation-like factor 6 interacting protein 4 | 598.5323 |
| 220761_s_at | TAOK3 | TAO kinase 3 | 173.6057 |
| 220806_x_at | GNG13 | guanine nucleotide binding protein (G protein), gamma 13 | 123.0223 |
| 220808_at | THEG | Theg homolog (mouse) | 567.5075 |
| 220841_s_at | AHI1 | Abelson helper integration site 1 | 67.24672 |
| 220842_at | AHI1 | Abelson helper integration site 1 | 37.12322 |
| 220936_s_at | NM_018267 | H2A histone family, member J | 39.9433 |
| 220964_s_at | RAB1B | RAB1B, member RAS oncogene family | 1458.08 |
| 220980_s_at | ADPGK | ADP-dependent glucokinase | 3507.659 |
| 221026_s_at | SCRT1 | scratch homolog 1, zinc finger protein (Drosophila) | 100.5229 |
| 221045_s_at | PER3 | period homolog 3 (Drosophila) | 67.83674 |
| 221156_x_at | CCPG1 | cell cycle progression 1 | 198.8273 |
| 221189_s_at | TARS2 | threonyl-tRNA synthetase 2, mitochondrial (putative) | 281.5162 |
| 221226_s_at | ACCN4 | amiloride-sensitive cation channel 4, pituitary | 270.5077 |
| 221237_s_at | OSBP2 | oxysterol binding protein 2 | 277.9841 |
| 221245_s_at | NM_030804 | frizzled homolog 5 (Drosophila) | 33.43821 |
| 221246_x_at | NM_018274 | tensin 1 | 213.9706 |
| 221252_s_at | GSG1 | germ cell associated 1 | 59.91266 |
| 221335_x_at | C19orf61 | chromosome 19 open reading frame 61 | 283.9842 |
| 221417_x_at | S1PR5 | sphingosine-1-phosphate receptor 5 | 119.8222 |
| 221424_s_at | OR51E2 | olfactory receptor, family 51, subfamily E, member 2 | 154.6873 |
| 221443_x_at | PRLH | prolactin releasing hormone | 142.4797 |
| 221447_s_at | GLT8D2 | glycosyltransferase 8 domain containing 2 | 214.1749 |
| 221494_x_at | EIF3K | eukaryotic translation initiation factor 3, subunit K | 7739.84 |
| 221499_s_at | STX16 | syntaxin 16 | 1004.486 |
| 221500_s_at | STX16 | syntaxin 16 | 1798.708 |
| 221508_at | TAOK3 | TAO kinase 3 | 219.5746 |
| 221511_x_at | CCPG1 | cell cycle progression 1 | 445.8681 |
| 221569_at | AHI1 | Abelson helper integration site 1 | 145.5964 |
| 221610_s_at | STAP2 | signal transducing adaptor family member 2 | 216.1051 |
| 221631_at | CACNA1I | calcium channel, voltage-dependent, T type, alpha 1I subunit | 180.887 |
| 221638_s_at | STX16 | syntaxin 16 | 84.97053 |
| 221663_x_at | HRH3 | histamine receptor H3 | 249.2569 |
| 221733_s_at | GPATCH4 | G patch domain containing 4 | 343.9017 |
| 221747_at | TNS1 | tensin 1 | 293.152 |
| 221748_s_at | TNS1 | tensin 1 | 219.5982 |
| 221790_s_at | LDLRAP1 | low density lipoprotein receptor adaptor protein 1 | 125.942 |
| 221857_s_at | TJAP1 | tight junction associated protein 1 (peripheral) | 321.38 |
| 221866_at | TFEB | transcription factor EB | 498.0004 |
| 221925_s_at | CSPP1 | centrosome and spindle pole associated protein 1 | 97.21185 |
| 221939_at | CARM1 | Yip1 domain family, member 2 | 605.4962 |
| 221956_at | AI631881 | leucine-rich repeats and calponin homology (CH) domain containing 4 | 475.6701 |
| 222017_x_at | LRCH4 | leucine-rich repeats and calponin homology (CH) domain containing 4 | 569.5976 |
| 222025_s_at | OPLAH | 5-oxoprolinase (ATP-hydrolysing) | 82.37481 |
| 222082_at | ZBTB7A | zinc finger and BTB domain containing 7A | 450.4524 |
| 222089_s_at | C16orf71 | chromosome 16 open reading frame 71 | 143.7038 |
| 222156_x_at | CCPG1 | cell cycle progression 1 | 50.2875 |
| 222160_at | AKAP8L | A kinase (PRKA) anchor protein 8-like | 294.7874 |
| 222190_s_at | C16orf58 | chromosome 16 open reading frame 58 | 194.2899 |
| 222239_s_at | INTS6 | integrator complex subunit 6 | 25.86303 |
| 222289_at | KCNC2 | potassium voltage-gated channel, Shaw-related subfamily, member 2 | 111.3979 |
| 32402_s_at | SYMPK | symplekin | 145.094 |
| 32625_at | NPR1 | natriuretic peptide receptor A/guanylate cyclase A (atrionatriuretic peptide receptor A) | 165.0882 |
| 32723_at | CSTF1 | cleavage stimulation factor, 3' pre-RNA, subunit 1, 50kDa | 31.50787 |
| 33767_at | NEFH | neurofilament, heavy polypeptide | 38.52116 |
| 34260_at | TELO2 | TEL2, telomere maintenance 2, homolog (S. cerevisiae) | 240.5844 |
| 35685_at | HLA-DPA3 | ring finger protein 1 | 1793.801 |
| 35974_at | LRMP | lymphoid-restricted membrane protein | 608.5603 |
| 36030_at | IFFO | intermediate filament family orphan 1 | 771.0838 |
| 36829_at | PER1 | period homolog 1 (Drosophila) | 963.7121 |
| 37796_at | AF053356 | leucine-rich repeats and calponin homology (CH) domain containing 4 | 828.1312 |
| 37872_at | JRK | jerky homolog (mouse) | 246.2338 |
| 40569_at | MZF1 | myeloid zinc finger 1 | 318.237 |
| 40612_at | DOPEY1 | dopey family member 1 | 58.60622 |
| 47608_at | TJAP1 | tight junction associated protein 1 (peripheral) | 604.2094 |
| 50221_at | TFEB | transcription factor EB | 662.9111 |
| 51192_at | SSH3 | slingshot homolog 3 (Drosophila) | 505.5201 |
| 57082_at | LDLRAP1 | low density lipoprotein receptor adaptor protein 1 | 335.0559 |
| 57163_at | ELOVL1 | elongation of very long chain fatty acids (FEN1/Elo2, SUR4/Elo3, yeast)-like 1 | 542.4002 |
| 65086_at | YIPF2 | Yip1 domain family, member 2 | 505.9261 |
| 89977_at | ACSM5 | acyl-CoA synthetase medium-chain family member 5 | 145.8516 |
| 90610_at | LRCH4 | leucine-rich repeats and calponin homology (CH) domain containing 4 | 2499.437 |
