# Supplementary material for: Transcriptome analysis of amoeboid and ramified microglia isolated from the corpus callosum of rat brain
Source: BMC Neurosci. 2012 Jun 14;13:64. doi: 10.1186/1471-2202-13-64 (PMC3441342; doi:10.1186/1471-2202-13-64)
Supplement: Additional file 8 — Sheet S7. ESC, NSC and HSC genes enriched in AMC and RMC. [file 1471-2202-13-64-S8.docx]

|  |  | **ESC genes specific to AMC** |  |
| --- | --- | --- | --- |
| **Probe Set ID** | **Gene Symbol** | **Gene Title** | **Fold Change** |
| 1373784_at | Cct8 | chaperonin containing Tcp1, subunit 8 (theta) | 13.525 |
| 1383747_at | Ect2 | epithelial cell transforming sequence 2 oncogene | 12.498 |
| 1388928_at | Cfl2 | cofilin 2, muscle | 12.211 |
| 1373458_at | Bex4 | brain expressed gene 4 | 9.4991 |
| 1370309_a_at | Hnrnpab | heterogeneous nuclear ribonucleoprotein A/B | 8.8712 |
| 1376648_at | Mycn | v-myc myelocytomatosis viral related oncogene, neuroblastoma derived (avian) | 8.6884 |
| 1374638_at | Pex13 | peroxisomal biogenesis factor 13 | 8.4726 |
| 1370678_s_at | Maoa | monoamine oxidase A | 7.8728 |
| 1377772_at | Tmeff1 | transmembrane protein with EGF-like and two follistatin-like domains 1 | 7.7994 |
| 1390485_at | March5 | membrane-associated ring finger (C3HC4) 5 | 6.9148 |
| 1387017_at | Sqle | squalene epoxidase | 6.7699 |
| 1382385_at | Psmc6 | proteasome (prosome, macropain) 26S subunit, ATPase, 6 | 6.4896 |
| 1371779_at | Snx6 | sorting nexin 6 | 6.3942 |
| 1373022_at | Rab1 | RAB1, member RAS oncogene family | 6.3274 |
| 1371949_at | Bzw1 | basic leucine zipper and W2 domains 1 | 6.3039 |
| 1370899_at | Sfpq | splicing factor proline/glutamine rich (polypyrimidine tract binding protein associated) | 6.2932 |
| 1373917_at | Etf1 | Eukaryotic translation termination factor 1 | 5.692 |
| 1372209_at | Cops5 | COP9 constitutive photomorphogenic homolog subunit 5 (Arabidopsis) | 5.5306 |
| 1378264_at | Nasp | Nuclear autoantigenic sperm protein (histone-binding) | 5.2995 |
| 1369954_at | Idh1 | isocitrate dehydrogenase 1 (NADP+), soluble | 5.2005 |
| 1369971_a_at | Hnrpd | heterogeneous nuclear ribonucleoprotein D | 5.094 |
| 1372462_at | Acat2 | acetyl-Coenzyme A acetyltransferase 2 | 5.0498 |
| 1375337_at | Adam9 | ADAM metallopeptidase domain 9 (meltrin gamma) | 5.0154 |
| 1384465_at | Pspc1 | paraspeckle component 1 | 5.0087 |
| 1398756_at | LOC300303 /// Npm1 | similar to Nucleophosmin (NPM) (Nucleolar phosphoprotein B23) (Numatrin) (Nucleolar protein NO38) /// nucleophosmin (nucleolar phosphoprotein B23, numatrin) | 4.9399 |
| 1390489_at | Syncrip | Synaptotagmin binding, cytoplasmic RNA interacting protein | 4.8936 |
| 1372575_at | Wbp11 | WW domain binding protein 11 | 4.7604 |
| 1368214_at | Smad2 | SMAD family member 2 | 4.7514 |
| 1388712_at | Cyth3 | cytohesin 3 | 4.7187 |
| 1389226_at | Stag1 | stromal antigen 1 | 4.6871 |
| 1388755_at | Sec23a | Sec23 homolog A (S. cerevisiae) | 4.6661 |
| 1372116_at | Mrps2 | mitochondrial ribosomal protein S2 | 4.6279 |
| 1398903_at | Esd | esterase D/formylglutathione hydrolase | 4.616 |
| 1368869_at | Akap12 | A kinase (PRKA) anchor protein 12 | 4.5495 |
| 1371653_at | Tpm4 | Tropomyosin 4 | 4.5474 |
| 1372566_at | Mtch2 | mitochondrial carrier homolog 2 (C. elegans) | 4.4249 |
| 1367631_at | Ctgf | connective tissue growth factor | 4.374 |
| 1367613_at | Prdx1 | peroxiredoxin 1 | 4.3624 |
| 1370959_at | Col3a1 | collagen, type III, alpha 1 | 4.3557 |
| 1389480_at | Rwdd4a | RWD domain containing 4A | 4.3289 |
| 1367485_at | Tcea1 | transcription elongation factor A (SII) 1 | 4.3035 |
| 1373846_at | Btf3l4 | basic transcription factor 3-like 4 | 4.2585 |
| 1367731_at | Gnb1 | guanine nucleotide binding protein (G protein), beta polypeptide 1 | 4.2345 |
| 1367488_at | Ankrd17 | ankyrin repeat domain 17 | 4.2225 |
| 1367683_at | Kpna2 | karyopherin (importin) alpha 2 | 4.216 |
| 1375538_at | Vcl | Vinculin | 4.2035 |
| 1377676_at | Nucks1 | nuclear casein kinase and cyclin-dependent kinase substrate 1 | 4.1448 |
| 1368852_at | Dnaja1 | DnaJ (Hsp40) homolog, subfamily A, member 1 | 4.1353 |
| 1384068_at | Ckap2 | cytoskeleton associated protein 2 | 4.1187 |
| 1371445_at | Lrrc59 | leucine rich repeat containing 59 | 4.0964 |
| 1383156_at | Kif2a | kinesin family member 2A | 4.0729 |
| 1370869_at | Bcat1 | branched chain aminotransferase 1, cytosolic | 4.0145 |
| 1386683_at | Atrx | alpha thalassemia/mental retardation syndrome X-linked (RAD54 homolog, S. cerevisiae) | 3.9841 |
| 1386394_at | Prps1 | phosphoribosyl pyrophosphate synthetase 1 | 3.9782 |
| 1398773_at | Khdrbs1 | KH domain containing, RNA binding, signal transduction associated 1 | 3.9389 |
| 1370524_at | Ube2d2 | Ubiquitin-conjugating enzyme E2D 2 | 3.9077 |
| 1399160_a_at | Ube2d3 | ubiquitin-conjugating enzyme E2D 3 (UBC4/5 homolog, yeast) | 3.8946 |
| 1367834_at | Srm | spermidine synthase | 3.8919 |
| 1385248_a_at | Ogn | osteoglycin | 3.8731 |
| 1368878_at | Idi1 | isopentenyl-diphosphate delta isomerase 1 | 3.8687 |
| 1398874_at | Atxn10 | ataxin 10 | 3.8086 |
| 1368582_at | Slc7a3 | solute carrier family 7 (cationic amino acid transporter, y+ system), member 3 | 3.7468 |
| 1391415_at | Pno1 | partner of NOB1 homolog (S. cerevisiae) | 3.7362 |
| 1382099_at | Vps26a | vacuolar protein sorting 26 homolog A (S. pombe) | 3.7143 |
| 1375889_at | Sms | spermine synthase | 3.7039 |
| 1385874_at | Usp42 | Ubiquitin specific peptidase 42 | 3.6708 |
| 1376065_at | Rrs1 | RRS1 ribosome biogenesis regulator homolog (S. cerevisiae) | 3.571 |
| 1389968_at | Eif3s10 | eukaryotic translation initiation factor 3, subunit 10 (theta) | 3.5688 |
| 1373408_at | Tbca | tubulin folding cofactor A | 3.5647 |
| 1393242_at | RGD1310922 | similar to chromosome 16 open reading frame 33; minus -99 protein | 3.5563 |
| 1371962_at | Tufm | Tu translation elongation factor, mitochondrial | 3.546 |
| 1367832_at | Lypla1 | lysophospholipase 1 | 3.5409 |
| 1390272_at | Dph5 | DPH5 homolog (S. cerevisiae) | 3.5354 |
| 1377163_at | Inhbb | Inhibin beta-B | 3.4699 |
| 1373418_at | Eprs | glutamyl-prolyl-tRNA synthetase | 3.4617 |
| 1398904_at | Nono | non-POU domain containing, octamer-binding | 3.4584 |
| 1398797_at | Hnrnpk | heterogeneous nuclear ribonucleoprotein K | 3.4439 |
| 1398439_a_at | Orc6l | origin recognition complex, subunit 6 like (yeast) | 3.4312 |
| 1370188_at | Sfrs10 | splicing factor, arginine/serine-rich 10 (transformer 2 homolog, Drosophila) | 3.3884 |
| 1380443_at | Pwp1 | PWP1 homolog (S. cerevisiae) | 3.3871 |
| 1386096_at | Mtf2 | metal response element binding transcription factor 2 | 3.3769 |
| 1367469_at | Eif4g2 /// Eif4g2-ps1 /// LOC678831 | eukaryotic translation initiation factor 4, gamma 2 /// eukaryotic translation initiation factor 4, gamma 2, pseudogene 1 /// similar to eukaryotic translation initiation factor 4 gamma, 2 | 3.3536 |
| 1367586_at | Ldha | lactate dehydrogenase A | 3.3412 |
| 1390692_at | Ctps | CTP synthase | 3.3248 |
| 1387921_at | Zc3h14 | zinc finger CCCH type containing 14 | 3.3198 |
| 1374571_at | Pigx | phosphatidylinositol glycan anchor biosynthesis, class X | 3.3109 |
| 1383339_at | RGD1310358 | similar to NNX3 | 3.2941 |
| 1389292_at | Rab18 | RAB18, member RAS oncogene family | 3.273 |
| 1394591_at | Zfp207 | zinc finger protein 207 | 3.2727 |
| 1367935_at | Smu1 | smu-1 suppressor of mec-8 and unc-52 homolog (C. elegans) | 3.267 |
| 1367590_at | Ran | RAN, member RAS oncogene family | 3.2657 |
| 1385157_at | Cbx1 | chromobox homolog 1 (HP1 beta homolog Drosophila ) | 3.2536 |
| 1378127_at | Cul2 | cullin 2 | 3.2253 |
| 1371375_at | Dstn | destrin | 3.2147 |
| 1383075_at | Ccnd1 | cyclin D1 | 3.201 |
| 1371960_at | Ythdf2 | YTH domain family, member 2 | 3.1958 |
| 1368405_at | Rala | v-ral simian leukemia viral oncogene homolog A (ras related) | 3.1901 |
| 1393247_at | Zfp26 | zinc finger protein 26 | 3.1901 |
| 1398786_at | Psmb2 | proteasome (prosome, macropain) subunit, beta type 2 | 3.1812 |
| 1398757_at | Npm1 | nucleophosmin (nucleolar phosphoprotein B23, numatrin) | 3.1793 |
| 1389857_at | Wbp5 | WW domain binding protein 5 | 3.1767 |
| 1393407_at | Fbxw2 | F-box and WD repeat domain containing 2 | 3.176 |
| 1374840_at | RGD1564921 | similar to peptidyl prolyl isomerase H | 3.1651 |
| 1383269_at | Rnf2 | ring finger protein 2 | 3.1366 |
| 1371851_at | Psmd6 | proteasome (prosome, macropain) 26S subunit, non-ATPase, 6 | 3.1318 |
| 1388865_at | Ppp4r2 | protein phosphatase 4, regulatory subunit 2 | 3.1299 |
| 1370909_at | Nup62 | nucleoporin 62 | 3.128 |
| 1368002_at | Msh2 | mutS homolog 2 (E. coli) | 3.1126 |
| 1388483_at | Cfl2 | cofilin 2, muscle | 3.0851 |
| 1370908_at | Hdac2 | histone deacetylase 2 | 3.0713 |
| 1367713_at | Eif2s1 | eukaryotic translation initiation factor 2, subunit 1 alpha | 3.0664 |
| 1388126_at | Minpp1 | multiple inositol polyphosphate histidine phosphatase 1 | 3.063 |
| 1377850_at | Znrf1 | zinc and ring finger 1 | 3.0433 |
| 1367515_at | Cnot7 | CCR4-NOT transcription complex, subunit 7 | 3.0397 |
| 1389296_at | Ttc35 | tetratricopeptide repeat domain 35 | 3.0273 |
| 1398784_at | C1qbp | complement component 1, q subcomponent binding protein | 3.012 |
| 1394854_at | Terf1 | telomeric repeat binding factor (NIMA-interacting) 1 | 2.9664 |
| 1371982_at | Dpy30 | dpy-30 homolog (C. elegans) | 2.9468 |
| 1371544_at | Erh | enhancer of rudimentary homolog (Drosophila) | 2.937 |
| 1389162_at | Nfu1 | NFU1 iron-sulfur cluster scaffold homolog (S. cerevisiae) | 2.9234 |
| 1374067_at | RGD1308127 | similar to 2700078E11Rik protein | 2.9173 |
| 1372460_at | LOC502130 /// RGD1562760 /// Set | similar to SET protein (Phosphatase 2A inhibitor I2PP2A) (I-2PP2A) (Template-activating factor I) (TAF-I) (Liver regeneration-related protein LRRGR00002) /// RGD1562760 /// SET nuclear oncogene | 2.9153 |
| 1398778_at | Psma1 | proteasome (prosome, macropain) subunit, alpha type 1 | 2.9127 |
| 1387060_at | Klf6 | Kruppel-like factor 6 | 2.8977 |
| 1373267_at | Sh3yl1 | SH3 domain containing, Ysc84-like 1 (S. cerevisiae) | 2.8858 |
| 1389655_at | Ptrh2 | peptidyl-tRNA hydrolase 2 | 2.8778 |
| 1397363_at | Pvrl3 | Poliovirus receptor-related 3 | 2.8757 |
| 1371518_at | Nid1 | Nidogen 1 | 2.8619 |
| 1388810_at | Abce1 | ATP-binding cassette, sub-family E (OABP), member 1 | 2.8519 |
| 1373273_at | Prpf38a | PRP38 pre-mRNA processing factor 38 (yeast) domain containing A | 2.85 |
| 1388428_at | Dtd1 | D-tyrosyl-tRNA deacylase 1 homolog (S. cerevisiae) | 2.8436 |
| 1372558_at | Narg1 | NMDA receptor regulated 1 | 2.8345 |
| 1367656_at | Psmb7 | proteasome (prosome, macropain) subunit, beta type 7 | 2.8327 |
| 1372173_at | RGD1309198 | similar to U5 snRNP-specific protein (Prp8-binding) | 2.8262 |
| 1398896_at | Arcn1 | archain 1 | 2.8136 |
| 1385090_at | Rad17 | RAD17 homolog (S. pombe) | 2.8077 |
| 1373380_at | Zc3h15 | zinc finger CCCH-type containing 15 | 2.7981 |
| 1368508_at | Psma3 /// Psma3l | proteasome (prosome, macropain) subunit, alpha type 3 /// proteasome subunit alpha type 3-like | 2.7971 |
| 1373063_at | Ube2k | ubiquitin-conjugating enzyme E2K (UBC1 homolog, yeast) | 2.797 |
| 1388802_at | Bex1 | brain expressed gene 1 | 2.7963 |
| 1389575_at | RGD1311703 | similar to sid2057p | 2.7805 |
| 1367734_at | Akr1b1 | aldo-keto reductase family 1, member B1 (aldose reductase) | 2.779 |
| 1372763_at | Nus1 | nuclear undecaprenyl pyrophosphate synthase 1 homolog (S. cerevisiae) | 2.7777 |
| 1398768_at | Rbbp7 | retinoblastoma binding protein 7 | 2.7533 |
| 1393412_at | Fam3c | family with sequence similarity 3, member C | 2.7499 |
| 1368450_at | Myo5a | myosin Va | 2.7463 |
| 1384280_at | Nusap1 | nucleolar and spindle associated protein 1 | 2.7393 |
| 1373048_at | Actr10 | actin-related protein 10 homolog (S. cerevisiae) | 2.7331 |
| 1398813_at | Uba3 | ubiquitin-like modifier activating enzyme 3 | 2.7254 |
| 1390420_at | Cpxm1 | carboxypeptidase X (M14 family), member 1 | 2.7249 |
| 1388975_at | Sucla2 | succinate-CoA ligase, ADP-forming, beta subunit | 2.7181 |
| 1390650_at | Nup85 | nucleoporin 85kDa | 2.718 |
| 1393041_at | Smc2 | structural maintenance of chromosomes 2 | 2.7164 |
| 1388715_at | Gars | glycyl-tRNA synthetase | 2.7116 |
| 1374595_at | Tnks2 | tankyrase, TRF1-interacting ankyrin-related ADP-ribose polymerase 2 | 2.6977 |
| 1393173_at | Rasa1 | RAS p21 protein activator (GTPase activating protein) 1 | 2.6875 |
| 1389546_at | Amotl2 | angiomotin like 2 | 2.6859 |
| 1398919_at | RGD1304704 | similar to Hypothetical protein CGI-99 | 2.6844 |
| 1380168_at | Etv4 | ets variant 4 | 2.6701 |
| 1372143_at | Ube2v2 | ubiquitin-conjugating enzyme E2 variant 2 | 2.6433 |
| 1370282_at | Csrp2 | cysteine and glycine-rich protein 2 | 2.641 |
| 1371818_at | Xpot | Exportin, tRNA (nuclear export receptor for tRNAs) | 2.633 |
| 1388960_at | Ppa1 | pyrophosphatase (inorganic) 1 | 2.6264 |
| 1383160_at | Chordc1 | cysteine and histidine-rich domain (CHORD)-containing 1 | 2.6046 |
| 1384615_at | Crop | Cisplatin resistance-associated overexpressed protein | 2.596 |
| 1371553_at | Mrpl36 | mitochondrial ribosomal protein L36 | 2.5912 |
| 1370344_at | Hspa4 | heat shock protein 4 | 2.5818 |
| 1386897_at | Prmt1 | protein arginine methyltransferase 1 | 2.5686 |
| 1374569_at | Grwd1 | glutamate-rich WD repeat containing 1 | 2.5531 |
| 1383736_at | Elavl2 | ELAV (embryonic lethal, abnormal vision, Drosophila)-like 2 (Hu antigen B) | 2.5411 |
| 1373946_at | Armc10 | armadillo repeat containing 10 | 2.5382 |
| 1376468_at | Hars | histidyl-tRNA synthetase | 2.5271 |
| 1390647_at | Phtf2 | putative homeodomain transcription factor 2 | 2.5097 |
| 1368273_at | Mapk6 | mitogen-activated protein kinase 6 | 2.5004 |
| 1371463_at | Phf5a | PHD finger protein 5A | 2.4986 |
| 1368032_at | Nolc1 | nucleolar and coiled-body phosphoprotein 1 | 2.4885 |
| 1373060_at | Chmp2b | chromatin modifying protein 2B | 2.4693 |
| 1398763_at | Timm23 | translocase of inner mitochondrial membrane 23 homolog (yeast) | 2.4471 |
| 1387201_at | Rnf138 | ring finger protein 138 | 2.4469 |
| 1387950_at | Nip7 | nuclear import 7 homolog (S. cerevisiae) | 2.4468 |
| 1382161_at | Mphosph10 | M-phase phosphoprotein 10 (U3 small nucleolar ribonucleoprotein) | 2.445 |
| 1382019_at | Alg5 | asparagine-linked glycosylation 5, dolichyl-phosphate beta-glucosyltransferase homolog (S. cerevisiae) | 2.4437 |
| 1379254_at | Tmem183a | transmembrane protein 183A | 2.4413 |
| 1389756_at | Melk | maternal embryonic leucine zipper kinase | 2.4276 |
| 1387186_at | Rab9a | RAB9A, member RAS oncogene family | 2.4162 |
| 1399075_at | Map3k7 | Mitogen activated protein kinase kinase kinase 7 | 2.4038 |
| 1375852_at | Hmgcr | 3-hydroxy-3-methylglutaryl-Coenzyme A reductase | 2.3966 |
| 1384435_at | Tmem68 | transmembrane protein 68 | 2.3823 |
| 1383522_at | Utx | ubiquitously transcribed tetratricopeptide repeat, X chromosome | 2.3663 |
| 1368668_at | Plaa | phospholipase A2, activating protein | 2.352 |
| 1369950_at | Cdk4 | cyclin-dependent kinase 4 | 2.3458 |
| 1386913_at | Pdpn | podoplanin | 2.3404 |
| 1371498_at | Jtv1 | JTV1 gene | 2.3333 |
| 1371403_at | Cct3 | chaperonin containing Tcp1, subunit 3 (gamma) | 2.3244 |
| 1399016_at | Myst2 | MYST histone acetyltransferase 2 | 2.3211 |
| 1367472_at | Uba1 | ubiquitin-like modifier activating enzyme 1 | 2.317 |
| 1371484_at | LOC690349 | hypothetical protein LOC690349 | 2.3055 |
| 1372363_at | Polr2h /// RGD1565904 | polymerase (RNA) II (DNA directed) polypeptide H /// similar to polymerase (RNA) II (DNA directed) polypeptide H | 2.2977 |
| 1376951_at | Mad2l1 | MAD2 (mitotic arrest deficient, homolog)-like 1 (yeast) | 2.2948 |
| 1390447_at | Stx3 | syntaxin 3 | 2.2896 |
| 1378124_at | Ppm1b | protein phosphatase 1B, magnesium dependent, beta isoform | 2.2869 |
| 1388979_at | Smndc1 | survival motor neuron domain containing 1 | 2.2793 |
| 1386881_at | Igfbp3 | insulin-like growth factor binding protein 3 | 2.2773 |
| 1371656_at | Cct4 | chaperonin containing Tcp1, subunit 4 (delta) | 2.2556 |
| 1391743_at | Elavl1 | ELAV (embryonic lethal, abnormal vision, Drosophila)-like 1 (Hu antigen R) | 2.253 |
| 1386872_at | Igf2r | insulin-like growth factor 2 receptor | 2.2447 |
| 1371876_at | Psmg2 | proteasome (prosome, macropain) assembly chaperone 2 | 2.2433 |
| 1373870_at | Fam98a | family with sequence similarity 98, member A | 2.2339 |
| 1367766_at | Nme2 | non-metastatic cells 2, protein (NM23B) expressed in | 2.2229 |
| 1373824_at | Cfdp1 | craniofacial development protein 1 | 2.194 |
| 1389248_at | Galk1 | galactokinase 1 | 2.1868 |
| 1370859_at | Pdia6 | protein disulfide isomerase family A, member 6 | 2.186 |
| 1371480_at | RGD1561797 | RGD1561797 | 2.1804 |
| 1392930_at | Armc1 | armadillo repeat containing 1 | 2.1661 |
| 1372155_at | Trim28 | tripartite motif-containing 28 | 2.1465 |
| 1387884_at | Psma5 | proteasome (prosome, macropain) subunit, alpha type 5 | 2.1221 |
| 1389184_at | Rpp30 | ribonuclease P/MRP 30 subunit (human) | 2.1175 |
| 1367831_at | Tp53 | tumor protein p53 | 2.1165 |
| 1373955_at | Ipo5 | importin 5 | 2.1063 |
| 1388830_at | Pkn2 | protein kinase N2 | 2.1041 |
| 1389576_at | Snrpb2 | small nuclear ribonucleoprotein polypeptide B'' | 2.1003 |
| 1387975_at | Ugcg | UDP-glucose ceramide glucosyltransferase | 2.0975 |
| 1367505_at | Znf644 | zinc finger protein 644 | 2.0884 |
| 1391475_at | Hnrpll | heterogeneous nuclear ribonucleoprotein L-like | 2.082 |
| 1367776_at | Cdc2 | cell division cycle 2, G1 to S and G2 to M | 2.0803 |
| 1367837_at | Psma4 | proteasome (prosome, macropain) subunit, alpha type 4 | 2.0768 |
| 1367818_at | Coq3 | coenzyme Q3 homolog, methyltransferase (S. cerevisiae) | 2.0642 |
| 1382154_at | Ptpn12 | protein tyrosine phosphatase, non-receptor type 12 | 2.058 |
| 1388550_at | Rad23b | RAD23 homolog B (S. cerevisiae) | 2.0565 |
| 1371830_at | Sae1 | SUMO1 activating enzyme subunit 1 | 2.0549 |
| 1369992_at | Psmd1 | proteasome (prosome, macropain) 26S subunit, non-ATPase, 1 | 2.0534 |
| 1374036_at | Mcm2 | minichromosome maintenance complex component 2 | 2.0525 |
| 1371042_at | Map4k3 | mitogen-activated protein kinase kinase kinase kinase 3 | 2.0458 |
| 1372255_at | Rars | arginyl-tRNA synthetase | 2.0443 |
| 1388723_at | Bre | brain and reproductive organ-expressed protein | 2.0428 |
| 1367575_at | Eno1 | enolase 1, (alpha) | 2.0424 |
| 1398800_at | Ywhab | tyrosine 3-monooxygenase/tryptophan 5-monooxygenase activation protein, beta polypeptide | 2.0295 |
| 1383502_at | Topbp1 | topoisomerase (DNA) II binding protein 1 | 2.0278 |
| 1370826_at | Nap1l1 | nucleosome assembly protein 1-like 1 | 2.0051 |
| 1387300_at | Crnkl1 | crooked neck pre-mRNA splicing factor-like 1 (Drosophila) | 2.0043 |

|  |  | **ESC genes specific to RMC** |  |
| --- | --- | --- | --- |
| **Probe Set ID** | **Gene Symbol** | **Gene Title** | **Fold Change** |
| 1372195_at | Tnnc2 | troponin C type 2 (fast) | 8.6024 |
| 1387117_at | Zranb2 | zinc finger, RAN-binding domain containing 2 | 4.598 |
| 1379691_at | Prpf38b | PRP38 pre-mRNA processing factor 38 (yeast) domain containing B | 4.3721 |
| 1370815_at | Nefh | neurofilament, heavy polypeptide | 4.2066 |
| 1398354_at | Ctnnal1 | catenin (cadherin associated protein), alpha-like 1 | 3.4127 |
| 1382056_at | Sfrs11 | splicing factor, arginine/serine-rich 11 | 3.3325 |
| 1379715_at | RGD1307882 | similar to CG9346-PA | 2.9758 |
| 1367570_at | Tagln | transgelin | 2.9554 |
| 1373654_at | Anxa8 | annexin A8 | 2.9225 |
| 1392965_a_at | Smoc2 | SPARC related modular calcium binding 2 | 2.7352 |
| 1395237_at | Eif5b | eukaryotic translation initiation factor 5B | 2.7087 |
| 1375128_at | Mtdh | Metadherin | 2.6164 |
| 1376799_a_at | Crlf1 | cytokine receptor-like factor 1 | 2.4905 |
| 1388539_at | Pkp2 | plakophilin 2 | 2.3106 |
| 1380577_at | Abcg2 | ATP-binding cassette, sub-family G (WHITE), member 2 | 2.3062 |
| 1368054_at | Lmna | lamin A | 2.2565 |
| 1392173_at | Zcchc3 | zinc finger, CCHC domain containing 3 | 2.2272 |
| 1373760_at | Tars2 | threonyl-tRNA synthetase 2, mitochondrial (putative) | 2.2069 |
| 1390305_at | Prkcbp1 | protein kinase C binding protein 1 | 2.1132 |
| 1395767_at | Cstf1 | cleavage stimulation factor, 3' pre-RNA, subunit 1 | 2.1104 |
| 1375058_at | Zrsr2 | zinc finger (CCCH type), RNA binding motif and serine/arginine rich 2 | 2.1065 |
| 1394292_at | Mrpl37 | mitochondrial ribosomal protein L37 | 2.0871 |
| 1380502_at | Zic2 | Zic family member 2 (odd-paired homolog, Drosophila) | 2.071 |
| 1396820_at | Hdac1 | histone deacetylase 1 | 2.0538 |
| 1370209_at | Klf9 | Kruppel-like factor 9 | 2.0462 |
| 1369329_at | Notch3 | Notch homolog 3 (Drosophila) | 2.0324 |

|  |  | **NSC genes specific to AMC** |  |
| --- | --- | --- | --- |
| **Probe Set ID** | **Gene Symbol** | **Gene Title** | **Fold Change** |
| 1390672_at | Rprm | reprimo, TP53 dependent G2 arrest mediator candidate | 19.125 |
| 1373784_at | Cct8 | chaperonin containing Tcp1, subunit 8 (theta) | 13.525 |
| 1380407_at | RGD1310352 | similar to HTGN29 protein; keratinocytes associated transmembrane protein 2 | 13.419 |
| 1383747_at | Ect2 | epithelial cell transforming sequence 2 oncogene | 12.498 |
| 1388928_at | Cfl2 | cofilin 2, muscle | 12.211 |
| 1383085_at | Sh3bgrl | SH3 domain binding glutamic acid-rich protein like | 9.7435 |
| 1387871_at | Cfl1 /// LOC502589 /// LOC688430 /// RGD1563644 | cofilin 1, non-muscle /// similar to Cofilin-1 (Cofilin, non-muscle isoform) /// similar to Cofilin-1 (Cofilin, non-muscle isoform) /// similar to Cofilin, non-muscle isoform (Cofilin-1) | 9.3235 |
| 1369953_a_at | Cd24 | CD24 molecule | 9.2544 |
| 1388340_at | Ns5atp9 | NS5A (hepatitis C virus) transactivated protein 9 | 9.1968 |
| 1370309_a_at | Hnrnpab | heterogeneous nuclear ribonucleoprotein A/B | 8.8712 |
| 1370948_a_at | LOC294446 /// LOC681252 /// Marcks | similar to Myristoylated alanine-rich C-kinase substrate (MARCKS) (ACAMP-81) /// similar to Myristoylated alanine-rich C-kinase substrate (MARCKS) (Protein kinase C substrate 80 kDa protein) /// myristoylated alanine rich protein kinase C substrate | 8.5949 |
| 1374638_at | Pex13 | peroxisomal biogenesis factor 13 | 8.4726 |
| 1370678_s_at | Maoa | monoamine oxidase A | 7.8728 |
| 1389534_at | Ube2e3 | ubiquitin-conjugating enzyme E2E 3, UBC4/5 homolog (yeast) | 7.5905 |
| 1375338_at | Rab10 | RAB10, member RAS oncogene family | 7.5637 |
| 1398758_at | Arf4 | ADP-ribosylation factor 4 | 7.3522 |
| 1375525_at | Mapre1 | microtubule-associated protein, RP/EB family, member 1 | 7.0496 |
| 1388582_at | Psme3 | proteasome (prosome, macropain) activator subunit 3 | 7.0045 |
| 1373595_at | Tmem43 | transmembrane protein 43 | 6.9166 |
| 1390485_at | March5 | membrane-associated ring finger (C3HC4) 5 | 6.9148 |
| 1387017_at | Sqle | squalene epoxidase | 6.7699 |
| 1369197_at | Apaf1 | apoptotic peptidase activating factor 1 | 6.6501 |
| 1369963_at | Pafah1b3 | platelet-activating factor acetylhydrolase, isoform 1b, subunit 3 | 6.5705 |
| 1382385_at | Psmc6 | proteasome (prosome, macropain) 26S subunit, ATPase, 6 | 6.4896 |
| 1373022_at | Rab1 | RAB1, member RAS oncogene family | 6.3274 |
| 1371949_at | Bzw1 | basic leucine zipper and W2 domains 1 | 6.3039 |
| 1370899_at | Sfpq | splicing factor proline/glutamine rich (polypyrimidine tract binding protein associated) | 6.2932 |
| 1399033_at | Cbfb | core-binding factor, beta subunit | 6.0758 |
| 1387398_at | Pkia | protein kinase (cAMP-dependent, catalytic) inhibitor alpha | 6.0141 |
| 1387857_at | Stx7 | syntaxin 7 | 5.899 |
| 1373917_at | Etf1 | Eukaryotic translation termination factor 1 | 5.692 |
| 1398888_at | H3f3b | H3 histone, family 3B | 5.6338 |
| 1372209_at | Cops5 | COP9 constitutive photomorphogenic homolog subunit 5 (Arabidopsis) | 5.5306 |
| 1390386_at | Casp3 | caspase 3, apoptosis related cysteine protease | 5.3212 |
| 1378264_at | Nasp | Nuclear autoantigenic sperm protein (histone-binding) | 5.2995 |
| 1369954_at | Idh1 | isocitrate dehydrogenase 1 (NADP+), soluble | 5.2005 |
| 1398250_at | Acot1 | acyl-CoA thioesterase 1 | 5.16 |
| 1391127_at | Cdc42 | Cell division cycle 42 (GTP binding protein) | 5.1257 |
| 1375652_at | Ssr3 | signal sequence receptor, gamma | 5.1035 |
| 1369971_a_at | Hnrpd | heterogeneous nuclear ribonucleoprotein D | 5.094 |
| 1372462_at | Acat2 | acetyl-Coenzyme A acetyltransferase 2 | 5.0498 |
| 1375337_at | Adam9 | ADAM metallopeptidase domain 9 (meltrin gamma) | 5.0154 |
| 1384465_at | Pspc1 | paraspeckle component 1 | 5.0087 |
| 1388378_at | Eif3c | eukaryotic translation initiation factor 3, subunit C | 4.9591 |
| 1398756_at | LOC300303 /// Npm1 | similar to Nucleophosmin (NPM) (Nucleolar phosphoprotein B23) (Numatrin) (Nucleolar protein NO38) /// nucleophosmin (nucleolar phosphoprotein B23, numatrin) | 4.9399 |
| 1390489_at | Syncrip | Synaptotagmin binding, cytoplasmic RNA interacting protein | 4.8936 |
| 1388581_at | Hn1 | hematological and neurological expressed 1 | 4.8833 |
| 1382680_at | Adfp | Adipose differentiation related protein | 4.8553 |
| 1367768_at | Lxn | latexin | 4.764 |
| 1372575_at | Wbp11 | WW domain binding protein 11 | 4.7604 |
| 1368214_at | Smad2 | SMAD family member 2 | 4.7514 |
| 1388996_at | Hdhd2 | haloacid dehalogenase-like hydrolase domain containing 2 | 4.7252 |
| 1371418_at | Cct2 | chaperonin containing TCP1, subunit 2 (beta) | 4.711 |
| 1389226_at | Stag1 | stromal antigen 1 | 4.6871 |
| 1375424_at | Actr2 | ARP2 actin-related protein 2 homolog (yeast) | 4.6831 |
| 1388755_at | Sec23a | Sec23 homolog A (S. cerevisiae) | 4.6661 |
| 1378178_at | Smc6l1 | SMC6 structural maintenance of chromosomes 6-like 1 (yeast) | 4.65 |
| 1372116_at | Mrps2 | mitochondrial ribosomal protein S2 | 4.6279 |
| 1368869_at | Akap12 | A kinase (PRKA) anchor protein 12 | 4.5495 |
| 1371653_at | Tpm4 | Tropomyosin 4 | 4.5474 |
| 1387275_at | Sox11 | SRY (sex determining region Y)-box 11 | 4.5022 |
| 1376727_at | Yipf4 | Yip1 domain family, member 4 | 4.4731 |
| 1374625_at | Hes6 | hairy and enhancer of split 6 (Drosophila) | 4.4412 |
| 1372566_at | Mtch2 | mitochondrial carrier homolog 2 (C. elegans) | 4.4249 |
| 1367613_at | Prdx1 | peroxiredoxin 1 | 4.3624 |
| 1398814_at | Rab11a | RAB11a, member RAS oncogene family | 4.33 |
| 1389480_at | Rwdd4a | RWD domain containing 4A | 4.3289 |
| 1367485_at | Tcea1 | transcription elongation factor A (SII) 1 | 4.3035 |
| 1367729_at | Oat | ornithine aminotransferase (gyrate atrophy) | 4.2756 |
| 1373846_at | Btf3l4 | basic transcription factor 3-like 4 | 4.2585 |
| 1369103_at | Fyn | FYN oncogene related to SRC, FGR, YES | 4.249 |
| 1367731_at | Gnb1 | guanine nucleotide binding protein (G protein), beta polypeptide 1 | 4.2345 |
| 1367488_at | Ankrd17 | ankyrin repeat domain 17 | 4.2225 |
| 1387806_at | Rap1b | RAP1B, member of RAS oncogene family | 4.2206 |
| 1367683_at | Kpna2 | karyopherin (importin) alpha 2 | 4.216 |
| 1371352_at | Hmgn2 | high mobility group nucleosomal binding domain 2 | 4.1954 |
| 1367890_at | Casp2 | caspase 2 | 4.1832 |
| 1386065_at | Ankrd57 | ankyrin repeat domain 57 | 4.1786 |
| 1377676_at | Nucks1 | nuclear casein kinase and cyclin-dependent kinase substrate 1 | 4.1448 |
| 1391201_at | Wdhd1 | WD repeat and HMG-box DNA binding protein 1 | 4.1387 |
| 1368852_at | Dnaja1 | DnaJ (Hsp40) homolog, subfamily A, member 1 | 4.1353 |
| 1384068_at | Ckap2 | cytoskeleton associated protein 2 | 4.1187 |
| 1371445_at | Lrrc59 | leucine rich repeat containing 59 | 4.0964 |
| 1383156_at | Kif2a | kinesin family member 2A | 4.0729 |
| 1375259_at | Eif4ebp2 | eukaryotic translation initiation factor 4E binding protein 2 | 4.0525 |
| 1385765_at | Lin9 /// LOC360888 | lin-9 homolog (C. elegans) /// similar to lin-9 homolog (C. elegans) | 4.0522 |
| 1373769_at | Pgm2 | phosphoglucomutase 2 | 4.0142 |
| 1372671_at | Rfk | riboflavin kinase | 3.9891 |
| 1383804_at | Dmrtb1 | DMRT-like family B with proline-rich C-terminal, 1 | 3.9872 |
| 1386394_at | Prps1 | phosphoribosyl pyrophosphate synthetase 1 | 3.9782 |
| 1368042_a_at | Hmg1l1 /// Hmgb1 /// LOC678705 /// LOC681718 /// RGD1562312 /// RGD1563012 /// RGD1563786 | high-mobility group (nonhistone chromosomal) protein 1-like 1 /// high mobility group box 1 /// hypothetical protein LOC678705 /// similar to High mobility group protein 1 (HMG-1) (High mobility group protein B1) (Amphoterin) (Heparin-binding protein p30) /// similar to High mobility group protein 1 (HMG-1) /// similar to High mobility group protein 1 (HMG-1) /// similar to Hmgb1 protein | 3.9596 |
| 1370258_at | Bzw2 | basic leucine zipper and W2 domains 2 | 3.9572 |
| 1398773_at | Khdrbs1 | KH domain containing, RNA binding, signal transduction associated 1 | 3.9389 |
| 1382146_at | Tspan6 | tetraspanin 6 | 3.9366 |
| 1388643_at | Fut8 | fucosyltransferase 8 (alpha (1,6) fucosyltransferase) | 3.9358 |
| 1368234_at | Prep | prolyl endopeptidase | 3.9299 |
| 1391387_s_at | Slbp | stem-loop binding protein | 3.9227 |
| 1370524_at | Ube2d2 | Ubiquitin-conjugating enzyme E2D 2 | 3.9077 |
| 1399160_a_at | Ube2d3 | ubiquitin-conjugating enzyme E2D 3 (UBC4/5 homolog, yeast) | 3.8946 |
| 1370890_at | Actr3 | ARP3 actin-related protein 3 homolog (yeast) | 3.8936 |
| 1367834_at | Srm | spermidine synthase | 3.8919 |
| 1398262_at | Prps2 | phosphoribosyl pyrophosphate synthetase 2 | 3.8814 |
| 1368878_at | Idi1 | isopentenyl-diphosphate delta isomerase 1 | 3.8687 |
| 1368308_at | Myc | myelocytomatosis oncogene | 3.835 |
| 1386908_at | Glrx1 | glutaredoxin 1 (thioltransferase) | 3.8276 |
| 1388562_at | Stard7 | StAR-related lipid transfer (START) domain containing 7 | 3.8204 |
| 1398874_at | Atxn10 | ataxin 10 | 3.8086 |
| 1369952_at | Pabpc1 | poly(A) binding protein, cytoplasmic 1 | 3.7796 |
| 1368642_at | Cdh2 | cadherin 2 | 3.7671 |
| 1389496_at | Akap7 | A kinase (PRKA) anchor protein 7 | 3.7522 |
| 1376606_a_at | Eny2 | enhancer of yellow 2 homolog (Drosophila) | 3.7453 |
| 1391415_at | Pno1 | partner of NOB1 homolog (S. cerevisiae) | 3.7362 |
| 1370213_at | Ybx1 | Y box binding protein 1 | 3.7191 |
| 1382099_at | Vps26a | vacuolar protein sorting 26 homolog A (S. pombe) | 3.7143 |
| 1375889_at | Sms | spermine synthase | 3.7039 |
| 1368189_at | Dhcr7 | 7-dehydrocholesterol reductase | 3.6913 |
| 1375119_at | Nedd4 | neural precursor cell expressed, developmentally down-regulated gene 4 | 3.6588 |
| 1398836_s_at | Actb | actin, beta | 3.6424 |
| 1381153_at | Anapc4 | anaphase promoting complex subunit 4 | 3.5894 |
| 1376065_at | Rrs1 | RRS1 ribosome biogenesis regulator homolog (S. cerevisiae) | 3.571 |
| 1389968_at | Eif3s10 | eukaryotic translation initiation factor 3, subunit 10 (theta) | 3.5688 |
| 1373408_at | Tbca | tubulin folding cofactor A | 3.5647 |
| 1388771_at | Cggbp1 | CGG triplet repeat binding protein 1 | 3.5626 |
| 1393242_at | RGD1310922 | similar to chromosome 16 open reading frame 33; minus -99 protein | 3.5563 |
| 1369642_at | Pafah1b2 | platelet-activating factor acetylhydrolase, isoform 1b, subunit 2 | 3.5476 |
| 1371962_at | Tufm | Tu translation elongation factor, mitochondrial | 3.546 |
| 1367832_at | Lypla1 | lysophospholipase 1 | 3.5409 |
| 1390272_at | Dph5 | DPH5 homolog (S. cerevisiae) | 3.5354 |
| 1368029_at | Gnai3 | guanine nucleotide binding protein (G protein), alpha inhibiting 3 | 3.5186 |
| 1377163_at | Inhbb | Inhibin beta-B | 3.4699 |
| 1398978_at | Ap1g1 | Adaptor-related protein complex 1, gamma 1 subunit | 3.4678 |
| 1373418_at | Eprs | glutamyl-prolyl-tRNA synthetase | 3.4617 |
| 1398904_at | Nono | non-POU domain containing, octamer-binding | 3.4584 |
| 1371777_at | Pabpc4 | poly A binding protein, cytoplasmic 4 | 3.4559 |
| 1374473_at | Ppp1r15b | protein phosphatase 1, regulatory (inhibitor) subunit 15b | 3.4508 |
| 1373080_at | Papola | poly (A) polymerase alpha | 3.4484 |
| 1398797_at | Hnrnpk | heterogeneous nuclear ribonucleoprotein K | 3.4439 |
| 1390022_at | Arpc5 | actin related protein 2/3 complex, subunit 5 | 3.4395 |
| 1371939_at | Caprin1 | cell cycle associated protein 1 | 3.4351 |
| 1388119_at | Hnrnpa3 | heterogeneous nuclear ribonucleoprotein A3 | 3.4339 |
| 1398439_a_at | Orc6l | origin recognition complex, subunit 6 like (yeast) | 3.4312 |
| 1371945_at | Ube2l3 | ubiquitin-conjugating enzyme E2L 3 | 3.4273 |
| 1388528_at | Fbl | fibrillarin | 3.4202 |
| 1371928_at | Cdca8 | cell division cycle associated 8 | 3.4068 |
| 1376143_at | Ddx54 | DEAD (Asp-Glu-Ala-Asp) box polypeptide 54 | 3.4002 |
| 1370188_at | Sfrs10 | splicing factor, arginine/serine-rich 10 (transformer 2 homolog, Drosophila) | 3.3884 |
| 1380443_at | Pwp1 | PWP1 homolog (S. cerevisiae) | 3.3871 |
| 1373894_at | Rab31 | RAB31, member RAS oncogene family | 3.3714 |
| 1367469_at | Eif4g2 /// Eif4g2-ps1 /// LOC678831 | eukaryotic translation initiation factor 4, gamma 2 /// eukaryotic translation initiation factor 4, gamma 2, pseudogene 1 /// similar to eukaryotic translation initiation factor 4 gamma, 2 | 3.3536 |
| 1367586_at | Ldha | lactate dehydrogenase A | 3.3412 |
| 1378320_at | Rlbp1 | retinaldehyde binding protein 1 | 3.341 |
| 1390692_at | Ctps | CTP synthase | 3.3248 |
| 1387921_at | Zc3h14 | zinc finger CCCH type containing 14 | 3.3198 |
| 1374571_at | Pigx | phosphatidylinositol glycan anchor biosynthesis, class X | 3.3109 |
| 1387863_at | Csde1 | cold shock domain containing E1, RNA binding | 3.2985 |
| 1383339_at | RGD1310358 | similar to NNX3 | 3.2941 |
| 1379582_a_at | Ccna2 | cyclin A2 | 3.2889 |
| 1374518_at | Tmem77 | transmembrane protein 77 | 3.2873 |
| 1389292_at | Rab18 | RAB18, member RAS oncogene family | 3.273 |
| 1394591_at | Zfp207 | zinc finger protein 207 | 3.2727 |
| 1373090_at | Ssr1 | signal sequence receptor, alpha | 3.2686 |
| 1367935_at | Smu1 | smu-1 suppressor of mec-8 and unc-52 homolog (C. elegans) | 3.267 |
| 1367590_at | Ran | RAN, member RAS oncogene family | 3.2657 |
| 1385157_at | Cbx1 | chromobox homolog 1 (HP1 beta homolog Drosophila ) | 3.2536 |
| 1386982_at | Mgat2 | mannosyl (alpha-1,6-)-glycoprotein beta-1,2-N-acetylglucosaminyltransferase | 3.2471 |
| 1373024_at | Ap3s1 | adaptor-related protein complex 3, sigma 1 subunit | 3.2373 |
| 1398810_at | Pdap1 | PDGFA associated protein 1 | 3.2303 |
| 1378127_at | Cul2 | cullin 2 | 3.2253 |
| 1389566_at | Ccnb2 | cyclin B2 | 3.2038 |
| 1383075_at | Ccnd1 | cyclin D1 | 3.201 |
| 1371960_at | Ythdf2 | YTH domain family, member 2 | 3.1958 |
| 1370357_at | Slc30a4 | solute carrier family 30 (zinc transporter), member 4 | 3.1945 |
| 1368405_at | Rala | v-ral simian leukemia viral oncogene homolog A (ras related) | 3.1901 |
| 1393247_at | Zfp26 | zinc finger protein 26 | 3.1901 |
| 1398757_at | Npm1 | nucleophosmin (nucleolar phosphoprotein B23, numatrin) | 3.1793 |
| 1389857_at | Wbp5 | WW domain binding protein 5 | 3.1767 |
| 1393407_at | Fbxw2 | F-box and WD repeat domain containing 2 | 3.176 |
| 1370003_at | Eef2 | eukaryotic translation elongation factor 2 | 3.1679 |
| 1385118_at | Eif2s1 /// LOC364604 /// LOC364984 | eukaryotic translation initiation factor 2, subunit 1 alpha /// hypothetical gene supported by NM_019356 /// hypothetical gene supported by NM_019356 | 3.1664 |
| 1374840_at | RGD1564921 | similar to peptidyl prolyl isomerase H | 3.1651 |
| 1381814_at | Srgap2 | SLIT-ROBO Rho GTPase activating protein 2 | 3.147 |
| 1389791_at | Cln8 | ceroid-lipofuscinosis, neuronal 8 | 3.1431 |
| 1383269_at | Rnf2 | ring finger protein 2 | 3.1366 |
| 1371851_at | Psmd6 | proteasome (prosome, macropain) 26S subunit, non-ATPase, 6 | 3.1318 |
| 1388865_at | Ppp4r2 | protein phosphatase 4, regulatory subunit 2 | 3.1299 |
| 1368002_at | Msh2 | mutS homolog 2 (E. coli) | 3.1126 |
| 1374747_at | Pftk1 | PFTAIRE protein kinase 1 | 3.0991 |
| 1370908_at | Hdac2 | histone deacetylase 2 | 3.0713 |
| 1388195_at | Cugbp2 | CUG triplet repeat, RNA binding protein 2 | 3.0617 |
| 1377850_at | Znrf1 | zinc and ring finger 1 | 3.0433 |
| 1367515_at | Cnot7 | CCR4-NOT transcription complex, subunit 7 | 3.0397 |
| 1391347_at | Rab8b | RAB8B, member RAS oncogene family | 3.0307 |
| 1389296_at | Ttc35 | tetratricopeptide repeat domain 35 | 3.0273 |
| 1367938_at | Ugdh | UDP-glucose dehydrogenase | 2.9758 |
| 1394854_at | Terf1 | telomeric repeat binding factor (NIMA-interacting) 1 | 2.9664 |
| 1371982_at | Dpy30 | dpy-30 homolog (C. elegans) | 2.9468 |
| 1394316_a_at | Tspan5 | tetraspanin 5 | 2.9458 |
| 1374565_at | Nek6 | NIMA (never in mitosis gene a)-related kinase 6 | 2.9406 |
| 1398839_at | Txn1 | thioredoxin 1 | 2.9385 |
| 1371544_at | Erh | enhancer of rudimentary homolog (Drosophila) | 2.937 |
| 1379583_at | Afg3l1 | AFG3(ATPase family gene 3)-like 1 (S. cerevisiae) | 2.926 |
| 1374067_at | RGD1308127 | similar to 2700078E11Rik protein | 2.9173 |
| 1372460_at | LOC502130 /// RGD1562760 /// Set | similar to SET protein (Phosphatase 2A inhibitor I2PP2A) (I-2PP2A) (Template-activating factor I) (TAF-I) (Liver regeneration-related protein LRRGR00002) /// RGD1562760 /// SET nuclear oncogene | 2.9153 |
| 1389293_at | Cpsf2 | cleavage and polyadenylation specific factor 2 | 2.9147 |
| 1398778_at | Psma1 | proteasome (prosome, macropain) subunit, alpha type 1 | 2.9127 |
| 1387856_at | Cnn3 | calponin 3, acidic | 2.8817 |
| 1389655_at | Ptrh2 | peptidyl-tRNA hydrolase 2 | 2.8778 |
| 1397363_at | Pvrl3 | Poliovirus receptor-related 3 | 2.8757 |
| 1371437_at | Sec13 | SEC13 homolog (S. cerevisiae) | 2.8556 |
| 1388810_at | Abce1 | ATP-binding cassette, sub-family E (OABP), member 1 | 2.8519 |
| 1373273_at | Prpf38a | PRP38 pre-mRNA processing factor 38 (yeast) domain containing A | 2.85 |
| 1382419_at | Cenpk | centromere protein K | 2.8488 |
| 1372558_at | Narg1 | NMDA receptor regulated 1 | 2.8345 |
| 1367656_at | Psmb7 | proteasome (prosome, macropain) subunit, beta type 7 | 2.8327 |
| 1372173_at | RGD1309198 | similar to U5 snRNP-specific protein (Prp8-binding) | 2.8262 |
| 1388514_at | Ppm1g | protein phosphatase 1G (formerly 2C), magnesium-dependent, gamma isoform | 2.8138 |
| 1398896_at | Arcn1 | archain 1 | 2.8136 |
| 1385090_at | Rad17 | RAD17 homolog (S. pombe) | 2.8077 |
| 1373380_at | Zc3h15 | zinc finger CCCH-type containing 15 | 2.7981 |
| 1368508_at | Psma3 /// Psma3l | proteasome (prosome, macropain) subunit, alpha type 3 /// proteasome subunit alpha type 3-like | 2.7971 |
| 1373063_at | Ube2k | ubiquitin-conjugating enzyme E2K (UBC1 homolog, yeast) | 2.797 |
| 1389575_at | RGD1311703 | similar to sid2057p | 2.7805 |
| 1372763_at | Nus1 | nuclear undecaprenyl pyrophosphate synthase 1 homolog (S. cerevisiae) | 2.7777 |
| 1371310_s_at | Serpinh1 | serine (or cysteine) peptidase inhibitor, clade H, member 1 | 2.7604 |
| 1398768_at | Rbbp7 | retinoblastoma binding protein 7 | 2.7533 |
| 1393412_at | Fam3c | family with sequence similarity 3, member C | 2.7499 |
| 1372303_at | Fam49b | family with sequence similarity 49, member B | 2.7477 |
| 1384280_at | Nusap1 | nucleolar and spindle associated protein 1 | 2.7393 |
| 1398813_at | Uba3 | ubiquitin-like modifier activating enzyme 3 | 2.7254 |
| 1390420_at | Cpxm1 | carboxypeptidase X (M14 family), member 1 | 2.7249 |
| 1388975_at | Sucla2 | succinate-CoA ligase, ADP-forming, beta subunit | 2.7181 |
| 1390650_at | Nup85 | nucleoporin 85kDa | 2.718 |
| 1393041_at | Smc2 | structural maintenance of chromosomes 2 | 2.7164 |
| 1388715_at | Gars | glycyl-tRNA synthetase | 2.7116 |
| 1374595_at | Tnks2 | tankyrase, TRF1-interacting ankyrin-related ADP-ribose polymerase 2 | 2.6977 |
| 1393173_at | Rasa1 | RAS p21 protein activator (GTPase activating protein) 1 | 2.6875 |
| 1388493_at | Ecop | EGFR-coamplified and overexpressed protein | 2.6853 |
| 1390115_at | Sec63 | SEC63 homolog (S. cerevisiae) | 2.6844 |
| 1398919_at | RGD1304704 | similar to Hypothetical protein CGI-99 | 2.6844 |
| 1385153_at | Zfp68 | zinc finger protein 68 | 2.6835 |
| 1380168_at | Etv4 | ets variant 4 | 2.6701 |
| 1372143_at | Ube2v2 | ubiquitin-conjugating enzyme E2 variant 2 | 2.6433 |
| 1392698_a_at | Gtf3c4 | general transcription factor IIIC, polypeptide 4 | 2.6396 |
| 1371818_at | Xpot | Exportin, tRNA (nuclear export receptor for tRNAs) | 2.633 |
| 1388960_at | Ppa1 | pyrophosphatase (inorganic) 1 | 2.6264 |
| 1373365_at | Cmpk1 | cytidine monophosphate (UMP-CMP) kinase 1, cytosolic | 2.6227 |
| 1383572_at | Zdhhc6 | zinc finger, DHHC domain containing 6 | 2.6184 |
| 1394079_at | Tbl1xr1 | transducin (beta)-like 1 X-linked receptor 1 | 2.6107 |
| 1388504_at | Rad21 | RAD21 homolog (S. pombe) | 2.6069 |
| 1383160_at | Chordc1 | cysteine and histidine-rich domain (CHORD)-containing 1 | 2.6046 |
| 1384615_at | Crop | Cisplatin resistance-associated overexpressed protein | 2.596 |
| 1371553_at | Mrpl36 | mitochondrial ribosomal protein L36 | 2.5912 |
| 1372720_at | Btbd1 | BTB (POZ) domain containing 1 | 2.5874 |
| 1383126_at | Akt1 | V-akt murine thymoma viral oncogene homolog 1 | 2.5848 |
| 1370344_at | Hspa4 | heat shock protein 4 | 2.5818 |
| 1386897_at | Prmt1 | protein arginine methyltransferase 1 | 2.5686 |
| 1387105_at | Zfp422 | zinc finger protein 422 | 2.5674 |
| 1398831_at | Psmb4 | proteasome (prosome, macropain) subunit, beta type 4 | 2.5649 |
| 1374569_at | Grwd1 | glutamate-rich WD repeat containing 1 | 2.5531 |
| 1389091_at | Usp3 | ubiquitin specific peptidase 3 | 2.5434 |
| 1376468_at | Hars | histidyl-tRNA synthetase | 2.5271 |
| 1390647_at | Phtf2 | putative homeodomain transcription factor 2 | 2.5097 |
| 1367580_at | Rpl10a | ribosomal protein L10A | 2.5057 |
| 1368273_at | Mapk6 | mitogen-activated protein kinase 6 | 2.5004 |
| 1371463_at | Phf5a | PHD finger protein 5A | 2.4986 |
| 1373060_at | Chmp2b | chromatin modifying protein 2B | 2.4693 |
| 1386967_at | Rhoq | ras homolog gene family, member Q | 2.458 |
| 1371436_at | Ddah2 | dimethylarginine dimethylaminohydrolase 2 | 2.4578 |
| 1398328_at | Foxk2 | forkhead box K2 | 2.4537 |
| 1386916_at | Aco1 | aconitase 1, soluble | 2.4529 |
| 1388382_at | LOC361985 | similar to NICE-3 | 2.4499 |
| 1398763_at | Timm23 | translocase of inner mitochondrial membrane 23 homolog (yeast) | 2.4471 |
| 1387201_at | Rnf138 | ring finger protein 138 | 2.4469 |
| 1387950_at | Nip7 | nuclear import 7 homolog (S. cerevisiae) | 2.4468 |
| 1382161_at | Mphosph10 | M-phase phosphoprotein 10 (U3 small nucleolar ribonucleoprotein) | 2.445 |
| 1382019_at | Alg5 | asparagine-linked glycosylation 5, dolichyl-phosphate beta-glucosyltransferase homolog (S. cerevisiae) | 2.4437 |
| 1379254_at | Tmem183a | transmembrane protein 183A | 2.4413 |
| 1395316_at | Mageh1 | melanoma antigen, family H, 1 | 2.4307 |
| 1389756_at | Melk | maternal embryonic leucine zipper kinase | 2.4276 |
| 1387186_at | Rab9a | RAB9A, member RAS oncogene family | 2.4162 |
| 1399075_at | Map3k7 | Mitogen activated protein kinase kinase kinase 7 | 2.4038 |
| 1375852_at | Hmgcr | 3-hydroxy-3-methylglutaryl-Coenzyme A reductase | 2.3966 |
| 1384161_at | Csnk1e | Casein kinase 1, epsilon | 2.392 |
| 1370180_at | Nudt4 | nudix (nucleoside diphosphate linked moiety X)-type motif 4 | 2.3847 |
| 1384435_at | Tmem68 | transmembrane protein 68 | 2.3823 |
| 1368668_at | Plaa | phospholipase A2, activating protein | 2.352 |
| 1398606_at | Golim4 | golgi integral membrane protein 4 | 2.3466 |
| 1369950_at | Cdk4 | cyclin-dependent kinase 4 | 2.3458 |
| 1386913_at | Pdpn | podoplanin | 2.3404 |
| 1377194_a_at | Ccdc90b | coiled-coil domain containing 90B | 2.3357 |
| 1367606_at | Rps3a | ribosomal protein S3a | 2.3336 |
| 1371403_at | Cct3 | chaperonin containing Tcp1, subunit 3 (gamma) | 2.3244 |
| 1367472_at | Uba1 | ubiquitin-like modifier activating enzyme 1 | 2.317 |
| 1371484_at | LOC690349 | hypothetical protein LOC690349 | 2.3055 |
| 1376951_at | Mad2l1 | MAD2 (mitotic arrest deficient, homolog)-like 1 (yeast) | 2.2948 |
| 1378124_at | Ppm1b | protein phosphatase 1B, magnesium dependent, beta isoform | 2.2869 |
| 1372122_at | Tsg101 | tumor susceptibility gene 101 | 2.2867 |
| 1388979_at | Smndc1 | survival motor neuron domain containing 1 | 2.2793 |
| 1386881_at | Igfbp3 | insulin-like growth factor binding protein 3 | 2.2773 |
| 1372156_at | Tmem97 | transmembrane protein 97 | 2.2699 |
| 1398405_at | Sept6 | septin 6 | 2.2675 |
| 1371696_at | Gpr56 | G protein-coupled receptor 56 | 2.2564 |
| 1371656_at | Cct4 | chaperonin containing Tcp1, subunit 4 (delta) | 2.2556 |
| 1391743_at | Elavl1 | ELAV (embryonic lethal, abnormal vision, Drosophila)-like 1 (Hu antigen R) | 2.253 |
| 1398934_at | Map3k7ip2 | mitogen-activated protein kinase kinase kinase 7 interacting protein 2 | 2.252 |
| 1392629_a_at | Zcrb1 | zinc finger CCHC-type and RNA binding motif 1 | 2.2458 |
| 1371876_at | Psmg2 | proteasome (prosome, macropain) assembly chaperone 2 | 2.2433 |
| 1373870_at | Fam98a | family with sequence similarity 98, member A | 2.2339 |
| 1367766_at | Nme2 | non-metastatic cells 2, protein (NM23B) expressed in | 2.2229 |
| 1373824_at | Cfdp1 | craniofacial development protein 1 | 2.194 |
| 1371662_at | Kars | lysyl-tRNA synthetase | 2.1934 |
| 1370859_at | Pdia6 | protein disulfide isomerase family A, member 6 | 2.186 |
| 1371480_at | RGD1561797 | RGD1561797 | 2.1804 |
| 1389521_at | Ivns1abp | influenza virus NS1A binding protein | 2.1766 |
| 1371461_at | Fam54b | family with sequence similarity 54, member B | 2.1692 |
| 1392930_at | Armc1 | armadillo repeat containing 1 | 2.1661 |
| 1380235_at | Hs2st1 | heparan sulfate 2-O-sulfotransferase 1 | 2.149 |
| 1372155_at | Trim28 | tripartite motif-containing 28 | 2.1465 |
| 1367662_at | Hsd17b10 | hydroxysteroid (17-beta) dehydrogenase 10 | 2.144 |
| 1370536_at | Prmt3 | protein arginine methyltransferase 3 | 2.1412 |
| 1387670_at | Gpd2 | glycerol-3-phosphate dehydrogenase 2, mitochondrial | 2.1267 |
| 1387884_at | Psma5 | proteasome (prosome, macropain) subunit, alpha type 5 | 2.1221 |
| 1389184_at | Rpp30 | ribonuclease P/MRP 30 subunit (human) | 2.1175 |
| 1367831_at | Tp53 | tumor protein p53 | 2.1165 |
| 1387797_at | Rab7a | RAB7A, member RAS oncogene family | 2.1125 |
| 1373955_at | Ipo5 | importin 5 | 2.1063 |
| 1367698_a_at | Sept9 | septin 9 | 2.1059 |
| 1388830_at | Pkn2 | protein kinase N2 | 2.1041 |
| 1389576_at | Snrpb2 | small nuclear ribonucleoprotein polypeptide B'' | 2.1003 |
| 1379645_at | Pbrm1 | polybromo 1 | 2.0965 |
| 1367900_at | Gyg1 | glycogenin 1 | 2.0948 |
| 1372715_at | Sfxn1 | sideroflexin 1 | 2.0947 |
| 1367505_at | Znf644 | zinc finger protein 644 | 2.0884 |
| 1391475_at | Hnrpll | heterogeneous nuclear ribonucleoprotein L-like | 2.082 |
| 1367776_at | Cdc2 | cell division cycle 2, G1 to S and G2 to M | 2.0803 |
| 1367818_at | Coq3 | coenzyme Q3 homolog, methyltransferase (S. cerevisiae) | 2.0642 |
| 1388550_at | Rad23b | RAD23 homolog B (S. cerevisiae) | 2.0565 |
| 1371830_at | Sae1 | SUMO1 activating enzyme subunit 1 | 2.0549 |
| 1369992_at | Psmd1 | proteasome (prosome, macropain) 26S subunit, non-ATPase, 1 | 2.0534 |
| 1374036_at | Mcm2 | minichromosome maintenance complex component 2 | 2.0525 |
| 1371042_at | Map4k3 | mitogen-activated protein kinase kinase kinase kinase 3 | 2.0458 |
| 1372255_at | Rars | arginyl-tRNA synthetase | 2.0443 |
| 1367575_at | Eno1 | enolase 1, (alpha) | 2.0424 |
| 1373907_at | Trappc4 | trafficking protein particle complex 4 | 2.0419 |
| 1398800_at | Ywhab | tyrosine 3-monooxygenase/tryptophan 5-monooxygenase activation protein, beta polypeptide | 2.0295 |
| 1383502_at | Topbp1 | topoisomerase (DNA) II binding protein 1 | 2.0278 |
| 1369638_at | Eef2k | eukaryotic elongation factor-2 kinase | 2.0269 |
| 1371908_at | Nxt1 | NTF2-like export factor 1 | 2.0257 |
| 1386875_a_at | Clta | clathrin, light chain (Lca) | 2.0214 |
| 1371365_at | Ube2s | ubiquitin-conjugating enzyme E2S | 2.0207 |
| 1398932_at | Hint1 | histidine triad nucleotide binding protein 1 | 2.0133 |
| 1371923_at | Lpcat1 | lysophosphatidylcholine acyltransferase 1 | 2.0101 |
| 1370826_at | Nap1l1 | nucleosome assembly protein 1-like 1 | 2.0051 |
| 1387300_at | Crnkl1 | crooked neck pre-mRNA splicing factor-like 1 (Drosophila) | 2.0043 |

|  |  | **NSC genes specific to RMC** |  |
| --- | --- | --- | --- |
| **Probe Set ID** | **Gene Symbol** | **Gene Title** | **Fold Change** |
| 1387117_at | Zranb2 | zinc finger, RAN-binding domain containing 2 | 4.598 |
| 1370412_at | Tnnt1 | troponin T type 1 (skeletal, slow) | 4.4062 |
| 1379046_at | Mlec | malectin | 3.5937 |
| 1374061_at | Cd302 | CD302 molecule | 3.3742 |
| 1382056_at | Sfrs11 | splicing factor, arginine/serine-rich 11 | 3.3325 |
| 1372531_at | Ppfibp2 | PTPRF interacting protein, binding protein 2 (liprin beta 2) | 3.2355 |
| 1379715_at | RGD1307882 | similar to CG9346-PA | 2.9758 |
| 1392561_at | Atp5c1 | ATP synthase, H+ transporting, mitochondrial F1 complex, gamma polypeptide 1 | 2.9722 |
| 1391995_at | Trim37 | tripartite motif-containing 37 | 2.8666 |
| 1395237_at | Eif5b | eukaryotic translation initiation factor 5B | 2.7087 |
| 1385163_at | Rnf20 | ring finger protein 20 | 2.6209 |
| 1375128_at | Mtdh | Metadherin | 2.6164 |
| 1398663_at | Zfp61 | zinc finger protein 61 | 2.4437 |
| 1384182_at | Fermt2 | fermitin family homolog 2 (Drosophila) | 2.3256 |
| 1389620_at | Suv420h2 | suppressor of variegation 4-20 homolog 2 (Drosophila) | 2.2717 |
| 1389306_at | Matn2 | matrilin 2 | 2.2574 |
| 1373760_at | Tars2 | threonyl-tRNA synthetase 2, mitochondrial (putative) | 2.2069 |
| 1371104_at | Srebf1 | sterol regulatory element binding transcription factor 1 | 2.2028 |
| 1367612_at | Mgst1 | microsomal glutathione S-transferase 1 | 2.1862 |
| 1390305_at | Prkcbp1 | protein kinase C binding protein 1 | 2.1132 |
| 1394292_at | Mrpl37 | mitochondrial ribosomal protein L37 | 2.0871 |
| 1396820_at | Hdac1 | histone deacetylase 1 | 2.0538 |
| 1373050_at | Tbc1d1 | TBC1 domain family, member 1 | 2.05 |
| 1370820_at | Fbxo6 | F-box protein 6 | 2.045 |
| 1397946_at | Sec14l1 | SEC14-like 1 (S. cerevisiae) | 2.0441 |
| 1369329_at | Notch3 | Notch homolog 3 (Drosophila) | 2.0324 |
| 1384337_x_at | Cln3 | ceroid-lipofuscinosis, neuronal 3 | 2.0291 |
| 1389120_at | Kcnc3 | potassium voltage gated channel, Shaw-related subfamily, member 3 | 2.0057 |

|  |  | **HSC genes specific to AMC** |  |
| --- | --- | --- | --- |
| **Probe Set ID** | **Gene Symbol** | **Gene Title** | **Fold Change** |
| 1390672_at | Rprm | reprimo, TP53 dependent G2 arrest mediator candidate | 19.125 |
| 1373458_at | Bex4 | brain expressed gene 4 | 9.4991 |
| 1376648_at | Mycn | v-myc myelocytomatosis viral related oncogene, neuroblastoma derived (avian) | 8.6884 |
| 1389534_at | Ube2e3 | ubiquitin-conjugating enzyme E2E 3, UBC4/5 homolog (yeast) | 7.5905 |
| 1370281_at | Fabp5 | fatty acid binding protein 5, epidermal | 6.5852 |
| 1368782_at | Sstr2 | somatostatin receptor 2 | 6.5297 |
| 1370899_at | Sfpq | splicing factor proline/glutamine rich (polypyrimidine tract binding protein associated) | 6.2932 |
| 1387398_at | Pkia | protein kinase (cAMP-dependent, catalytic) inhibitor alpha | 6.0141 |
| 1390386_at | Casp3 | caspase 3, apoptosis related cysteine protease | 5.3212 |
| 1398250_at | Acot1 | acyl-CoA thioesterase 1 | 5.16 |
| 1383401_at | Tes | testis derived transcript | 5.0625 |
| 1375337_at | Adam9 | ADAM metallopeptidase domain 9 (meltrin gamma) | 5.0154 |
| 1368175_at | Zhx1 | zinc fingers and homeoboxes 1 | 4.9074 |
| 1386882_at | Dynlt1 | dynein light chain Tctex-type 1 | 4.8568 |
| 1368214_at | Smad2 | SMAD family member 2 | 4.7514 |
| 1388712_at | Cyth3 | cytohesin 3 | 4.7187 |
| 1388755_at | Sec23a | Sec23 homolog A (S. cerevisiae) | 4.6661 |
| 1372116_at | Mrps2 | mitochondrial ribosomal protein S2 | 4.6279 |
| 1399119_at | Znf292 | zinc finger protein 292 | 4.3921 |
| 1398814_at | Rab11a | RAB11a, member RAS oncogene family | 4.33 |
| 1398350_at | Basp1 | brain abundant, membrane attached signal protein 1 | 4.3051 |
| 1388196_at | Nckap1 | NCK-associated protein 1 | 4.268 |
| 1367731_at | Gnb1 | guanine nucleotide binding protein (G protein), beta polypeptide 1 | 4.2345 |
| 1367488_at | Ankrd17 | ankyrin repeat domain 17 | 4.2225 |
| 1377097_at | Cox6b2 | cytochrome c oxidase subunit VIb polypeptide 2 | 4.1067 |
| 1383156_at | Kif2a | kinesin family member 2A | 4.0729 |
| 1373769_at | Pgm2 | phosphoglucomutase 2 | 4.0142 |
| 1368122_at | Rnf103 | ring finger protein 103 | 3.9452 |
| 1388643_at | Fut8 | fucosyltransferase 8 (alpha (1,6) fucosyltransferase) | 3.9358 |
| 1370524_at | Ube2d2 | Ubiquitin-conjugating enzyme E2D 2 | 3.9077 |
| 1383766_at | Sgcb | sarcoglycan, beta (dystrophin-associated glycoprotein) | 3.8373 |
| 1368308_at | Myc | myelocytomatosis oncogene | 3.835 |
| 1390802_at | RGD1306839 | similar to RIKEN cDNA 5033414D02 | 3.7345 |
| 1375889_at | Sms | spermine synthase | 3.7039 |
| 1367663_at | Psme1 | proteasome (prosome, macropain) activator subunit 1 | 3.6823 |
| 1375119_at | Nedd4 | neural precursor cell expressed, developmentally down-regulated gene 4 | 3.6588 |
| 1369972_at | Fbxo21 | F-box protein 21 | 3.6568 |
| 1381153_at | Anapc4 | anaphase promoting complex subunit 4 | 3.5894 |
| 1388771_at | Cggbp1 | CGG triplet repeat binding protein 1 | 3.5626 |
| 1369642_at | Pafah1b2 | platelet-activating factor acetylhydrolase, isoform 1b, subunit 2 | 3.5476 |
| 1390272_at | Dph5 | DPH5 homolog (S. cerevisiae) | 3.5354 |
| 1373418_at | Eprs | glutamyl-prolyl-tRNA synthetase | 3.4617 |
| 1388679_at | Tbc1d14 | TBC1 domain family, member 14 | 3.4342 |
| 1388631_at | Azi2 | 5-azacytidine induced 2 | 3.4308 |
| 1386096_at | Mtf2 | metal response element binding transcription factor 2 | 3.3769 |
| 1367469_at | Eif4g2 /// Eif4g2-ps1 /// LOC678831 | eukaryotic translation initiation factor 4, gamma 2 /// eukaryotic translation initiation factor 4, gamma 2, pseudogene 1 /// similar to eukaryotic translation initiation factor 4 gamma, 2 | 3.3536 |
| 1387921_at | Zc3h14 | zinc finger CCCH type containing 14 | 3.3198 |
| 1370250_at | Ube2i | ubiquitin-conjugating enzyme E2I | 3.3128 |
| 1374571_at | Pigx | phosphatidylinositol glycan anchor biosynthesis, class X | 3.3109 |
| 1383339_at | RGD1310358 | similar to NNX3 | 3.2941 |
| 1389292_at | Rab18 | RAB18, member RAS oncogene family | 3.273 |
| 1367728_at | Tsn | translin | 3.2222 |
| 1383075_at | Ccnd1 | cyclin D1 | 3.201 |
| 1389857_at | Wbp5 | WW domain binding protein 5 | 3.1767 |
| 1389791_at | Cln8 | ceroid-lipofuscinosis, neuronal 8 | 3.1431 |
| 1367759_at | H1f0 | H1 histone family, member 0 | 3.1325 |
| 1387025_at | Dync1i1 | dynein cytoplasmic 1 intermediate chain 1 | 3.124 |
| 1368002_at | Msh2 | mutS homolog 2 (E. coli) | 3.1126 |
| 1389309_at | Sbno1 | strawberry notch homolog 1 (Drosophila) | 3.1109 |
| 1374747_at | Pftk1 | PFTAIRE protein kinase 1 | 3.0991 |
| 1388330_at | Vkorc1 | vitamin K epoxide reductase complex, subunit 1 | 3.0627 |
| 1367515_at | Cnot7 | CCR4-NOT transcription complex, subunit 7 | 3.0397 |
| 1389528_s_at | Jun | Jun oncogene | 3.0095 |
| 1380202_at | Znf821 | zinc finger protein 821 | 2.9973 |
| 1387154_at | Npy | neuropeptide Y | 2.9739 |
| 1374565_at | Nek6 | NIMA (never in mitosis gene a)-related kinase 6 | 2.9406 |
| 1389293_at | Cpsf2 | cleavage and polyadenylation specific factor 2 | 2.9147 |
| 1374660_at | Nsmce4a | non-SMC element 4 homolog A (S. cerevisiae) | 2.9057 |
| 1398424_at | Wsb2 | WD repeat and SOCS box-containing 2 | 2.8979 |
| 1387060_at | Klf6 | Kruppel-like factor 6 | 2.8977 |
| 1367939_at | Rbp1 | retinol binding protein 1, cellular | 2.88 |
| 1397363_at | Pvrl3 | Poliovirus receptor-related 3 | 2.8757 |
| 1388428_at | Dtd1 | D-tyrosyl-tRNA deacylase 1 homolog (S. cerevisiae) | 2.8436 |
| 1371360_at | Ndrg1 | N-myc downstream regulated gene 1 | 2.8387 |
| 1379364_at | Camsap1 | calmodulin regulated spectrin-associated protein 1 | 2.8297 |
| 1398896_at | Arcn1 | archain 1 | 2.8136 |
| 1388802_at | Bex1 | brain expressed gene 1 | 2.7963 |
| 1367734_at | Akr1b1 | aldo-keto reductase family 1, member B1 (aldose reductase) | 2.779 |
| 1371310_s_at | Serpinh1 | serine (or cysteine) peptidase inhibitor, clade H, member 1 | 2.7604 |
| 1389081_at | Vps37b | vacuolar protein sorting 37 homolog B (S. cerevisiae) | 2.7574 |
| 1393412_at | Fam3c | family with sequence similarity 3, member C | 2.7499 |
| 1398780_at | Rabac1 | Rab acceptor 1 (prenylated) | 2.7357 |
| 1373048_at | Actr10 | actin-related protein 10 homolog (S. cerevisiae) | 2.7331 |
| 1390420_at | Cpxm1 | carboxypeptidase X (M14 family), member 1 | 2.7249 |
| 1388715_at | Gars | glycyl-tRNA synthetase | 2.7116 |
| 1393173_at | Rasa1 | RAS p21 protein activator (GTPase activating protein) 1 | 2.6875 |
| 1385153_at | Zfp68 | zinc finger protein 68 | 2.6835 |
| 1398803_at | Dync1h1 | dynein cytoplasmic 1 heavy chain 1 | 2.6624 |
| 1395045_at | Ndufa7 | NADH dehydrogenase (ubiquinone) 1 alpha subcomplex, 7 (B14.5a) | 2.6513 |
| 1370282_at | Csrp2 | cysteine and glycine-rich protein 2 | 2.641 |
| 1371818_at | Xpot | Exportin, tRNA (nuclear export receptor for tRNAs) | 2.633 |
| 1388960_at | Ppa1 | pyrophosphatase (inorganic) 1 | 2.6264 |
| 1370344_at | Hspa4 | heat shock protein 4 | 2.5818 |
| 1387280_a_at | Slc7a5 | solute carrier family 7 (cationic amino acid transporter, y+ system), member 5 | 2.5654 |
| 1377023_at | Dusp2 | dual specificity phosphatase 2 | 2.5647 |
| 1374569_at | Grwd1 | glutamate-rich WD repeat containing 1 | 2.5531 |
| 1389091_at | Usp3 | ubiquitin specific peptidase 3 | 2.5434 |
| 1372391_at | Rpl7l1 | ribosomal protein L7-like 1 | 2.5126 |
| 1371947_at | Ndn | necdin homolog (mouse) | 2.5112 |
| 1390647_at | Phtf2 | putative homeodomain transcription factor 2 | 2.5097 |
| 1367580_at | Rpl10a | ribosomal protein L10A | 2.5057 |
| 1374549_at | Rbm4b | RNA binding motif protein 4B | 2.4738 |
| 1375631_at | Plekhb2 | pleckstrin homology domain containing, family B (evectins) member 2 | 2.4672 |
| 1370813_at | Gstm5 | glutathione S-transferase, mu 5 | 2.4582 |
| 1386967_at | Rhoq | ras homolog gene family, member Q | 2.458 |
| 1398328_at | Foxk2 | forkhead box K2 | 2.4537 |
| 1387201_at | Rnf138 | ring finger protein 138 | 2.4469 |
| 1382161_at | Mphosph10 | M-phase phosphoprotein 10 (U3 small nucleolar ribonucleoprotein) | 2.445 |
| 1379254_at | Tmem183a | transmembrane protein 183A | 2.4413 |
| 1388941_at | Amz2 | archaelysin family metallopeptidase 2 | 2.3904 |
| 1371533_at | Dctn6 | dynactin 6 | 2.3803 |
| 1387769_a_at | Id3 | inhibitor of DNA binding 3 | 2.3788 |
| 1377194_a_at | Ccdc90b | coiled-coil domain containing 90B | 2.3357 |
| 1371498_at | Jtv1 | JTV1 gene | 2.3333 |
| 1373100_at | LOC499779 | similar to RIKEN cDNA 2900010J23 | 2.3044 |
| 1377103_at | Midn | midnolin | 2.296 |
| 1372122_at | Tsg101 | tumor susceptibility gene 101 | 2.2867 |
| 1377935_at | Cldnd1 | Claudin domain containing 1 | 2.2565 |
| 1371696_at | Gpr56 | G protein-coupled receptor 56 | 2.2564 |
| 1389381_at | Sqstm1 | sequestosome 1 | 2.2461 |
| 1372439_at | Col4a1 | collagen, type IV, alpha 1 | 2.2135 |
| 1398759_at | Tsc22d1 | TSC22 domain family, member 1 | 2.2026 |
| 1389868_at | RGD1564560 | similar to RCK | 2.151 |
| 1380235_at | Hs2st1 | heparan sulfate 2-O-sulfotransferase 1 | 2.149 |
| 1388568_at | Eif3d | eukaryotic translation initiation factor 3, subunit D | 2.1457 |
| 1388730_at | Cdc42ep4 | CDC42 effector protein (Rho GTPase binding) 4 | 2.1442 |
| 1387670_at | Gpd2 | glycerol-3-phosphate dehydrogenase 2, mitochondrial | 2.1267 |
| 1392885_at | Mbd1 | methyl-CpG binding domain protein 1 | 2.1202 |
| 1374516_at | Chtf8 | CTF8, chromosome transmission fidelity factor 8 homolog (S. cerevisiae) | 2.1014 |
| 1398890_at | MGC93975 | similar to 2310044H10Rik protein | 2.0977 |
| 1372364_a_at | Ntan1 | N-terminal asparagine amidase | 2.0945 |
| 1391453_at | Ebi3 | Epstein-Barr virus induced 3 | 2.0887 |
| 1367505_at | Znf644 | zinc finger protein 644 | 2.0884 |
| 1386985_at | Gstm1 | glutathione S-transferase mu 1 | 2.0783 |
| 1377341_at | RGD1305455 | similar to hypothetical protein FLJ10925 | 2.0634 |
| 1382154_at | Ptpn12 | protein tyrosine phosphatase, non-receptor type 12 | 2.058 |
| 1385487_at | Lsm1 | LSM1 homolog, U6 small nuclear RNA associated (S. cerevisiae) | 2.056 |
| 1371042_at | Map4k3 | mitogen-activated protein kinase kinase kinase kinase 3 | 2.0458 |
| 1373388_at | Sppl3 | signal peptide peptidase 3 | 2.0401 |
| 1388819_at | Scamp1 | secretory carrier membrane protein 1 | 2.0356 |
| 1398800_at | Ywhab | tyrosine 3-monooxygenase/tryptophan 5-monooxygenase activation protein, beta polypeptide | 2.0295 |
| 1379651_at | Foxp1 | Forkhead box P1 | 2.0145 |

|  |  | **HSC genes specific to RMC** |  |
| --- | --- | --- | --- |
| **Probe Set ID** | **Gene Symbol** | **Gene Title** | **Fold Change** |
| 1375813_at | Akap8l | A kinase (PRKA) anchor protein 8-like | 5.6932 |
| 1378864_at | Lrrc23 | leucine rich repeat containing 23 | 5.1395 |
| 1389874_at | Zrsr1 | zinc finger (CCCH type), RNA binding motif and serine/arginine rich 1 | 5.0348 |
| 1379691_at | Prpf38b | PRP38 pre-mRNA processing factor 38 (yeast) domain containing B | 4.3721 |
| 1386931_at | Tnni3 | troponin I type 3 (cardiac) | 4.0048 |
| 1375213_at | Pck2 | phosphoenolpyruvate carboxykinase 2 (mitochondrial) | 3.8945 |
| 1376102_at | Tmbim1 | transmembrane BAX inhibitor motif containing 1 | 3.7938 |
| 1387010_s_at | Scn1b | sodium channel, voltage-gated, type I, beta | 3.6699 |
| 1385074_at | Smarca2 | SWI/SNF related, matrix associated, actin dependent regulator of chromatin, subfamily a, member 2 | 3.663 |
| 1387916_at | Cyp4f6 | cytochrome P450 4F6 | 3.4649 |
| 1392916_at | Map7 | microtubule-associated protein 7 | 3.263 |
| 1368321_at | Egr1 | early growth response 1 | 3.2473 |
| 1377124_at | Mtf1 | metal-regulatory transcription factor 1 | 3.2416 |
| 1372531_at | Ppfibp2 | PTPRF interacting protein, binding protein 2 (liprin beta 2) | 3.2355 |
| 1388740_at | Fermt3 | fermitin family homolog 3 (Drosophila) | 3.0629 |
| 1395419_at | Mll1 | myeloid/lymphoid or mixed-lineage leukemia 1 | 2.9906 |
| 1387874_at | Dbp | D site of albumin promoter (albumin D-box) binding protein | 2.9682 |
| 1398297_at | Mapk12 | mitogen-activated protein kinase 12 | 2.9091 |
| 1387834_at | Matk | megakaryocyte-associated tyrosine kinase | 2.8995 |
| 1387908_at | Rasd1 | RAS, dexamethasone-induced 1 | 2.8931 |
| 1385043_at | Inadl | InaD-like (Drosophila) | 2.8349 |
| 1371959_at | LOC690131 | similar to H2A histone family, member O | 2.8217 |
| 1388710_at | Rreb1 | ras responsive element binding protein 1 | 2.8181 |
| 1389774_at | Znf23 | zinc finger protein 23 (KOX 16) | 2.806 |
| 1374725_at | Mov10 | Moloney leukemia virus 10 | 2.7372 |
| 1380989_at | Numa1 | nuclear mitotic apparatus protein 1 | 2.7358 |
| 1389515_at | Rfxank | regulatory factor X-associated ankyrin-containing protein | 2.6709 |
| 1398318_at | Muc1 | mucin 1, cell surface associated | 2.6672 |
| 1374117_at | Baiap2 | BAI1-associated protein 2 | 2.6217 |
| 1389339_at | Arsa | Arylsulfatase A | 2.6182 |
| 1382055_at | Rtkn | rhotekin | 2.6178 |
| 1372232_at | Wbp1 | WW domain binding protein 1 | 2.5074 |
| 1371997_at | Akr1cl2 | aldo-keto reductase family 1, member C-like 2 | 2.4959 |
| 1385779_at | Chd4 | chromodomain helicase DNA binding protein 4 | 2.4932 |
| 1373750_at | Leprel2 | leprecan-like 2 | 2.4659 |
| 1381339_at | Eif3k | eukaryotic translation initiation factor 3, subunit K | 2.4413 |
| 1381120_at | Invs | inversin | 2.4202 |
| 1374621_at | Taf1c | TATA box binding protein (Tbp)-associated factor, RNA polymerase I, C | 2.3723 |
| 1388539_at | Pkp2 | plakophilin 2 | 2.3106 |
| 1380577_at | Abcg2 | ATP-binding cassette, sub-family G (WHITE), member 2 | 2.3062 |
| 1377241_at | Znf503 | zinc finger protein 503 | 2.3051 |
| 1379633_a_at | Uba7 | ubiquitin-like modifier activating enzyme 7 | 2.2975 |
| 1374694_at | Ankrd28 | ankyrin repeat domain 28 | 2.2893 |
| 1389778_a_at | Tceb3 | transcription elongation factor B (SIII), polypeptide 3 | 2.2887 |
| 1387788_at | Junb | jun B proto-oncogene | 2.2878 |
| 1387252_at | Sec14l2 | SEC14-like 2 (S. cerevisiae) | 2.2858 |
| 1389002_at | Tln1 | talin 1 | 2.2627 |
| 1368054_at | Lmna | lamin A | 2.2565 |
| 1374855_at | Per1 | period homolog 1 (Drosophila) | 2.2415 |
| 1371664_at | Pxn | paxillin | 2.2373 |
| 1375478_at | Zfp362 /// Zscan20 | zinc finger protein 362 /// zinc finger and SCAN domain containing 20 | 2.232 |
| 1392173_at | Zcchc3 | zinc finger, CCHC domain containing 3 | 2.2272 |
| 1373760_at | Tars2 | threonyl-tRNA synthetase 2, mitochondrial (putative) | 2.2069 |
| 1371104_at | Srebf1 | sterol regulatory element binding transcription factor 1 | 2.2028 |
| 1369984_at | Cox17 | cytochrome c oxidase, subunit XVII assembly protein homolog (S. cerevisiae) | 2.1992 |
| 1375613_at | Gata6 | GATA binding protein 6 | 2.1637 |
| 1368772_at | Slc4a3 | solute carrier family 4 (anion exchanger), member 3 | 2.1577 |
| 1369590_a_at | Ddit3 | DNA-damage inducible transcript 3 | 2.1394 |
| 1374653_at | Fam73b | family with sequence similarity 73, member B | 2.1393 |
| 1395767_at | Cstf1 | cleavage stimulation factor, 3' pre-RNA, subunit 1 | 2.1104 |
| 1375058_at | Zrsr2 | zinc finger (CCCH type), RNA binding motif and serine/arginine rich 2 | 2.1065 |
| 1393067_at | Tek | TEK tyrosine kinase, endothelial | 2.1025 |
| 1375177_at | Klf13 | Kruppel-like factor 13 | 2.1024 |
| 1397607_at | RGD1310862 | similar to adult retina protein | 2.1012 |
| 1376176_at | Mast3 | microtubule associated serine/threonine kinase 3 | 2.0802 |
| 1375351_at | Ssbp4 | single stranded DNA binding protein 4 | 2.08 |
| 1375134_at | Dda1 | DET1 and DDB1 associated 1 | 2.0796 |
| 1393155_at | Plk3 | polo-like kinase 3 (Drosophila) | 2.079 |
| 1386998_at | Aldoc | aldolase C, fructose-bisphosphate | 2.077 |
| 1387776_at | Tgm2 | transglutaminase 2, C polypeptide | 2.0755 |
| 1371560_at | Irf3 | interferon regulatory factor 3 | 2.0647 |
| 1368749_at | Kcns1 | potassium voltage-gated channel, delayed-rectifier, subfamily S, member 1 | 2.0605 |
| 1368235_at | Clk3 | CDC-like kinase 3 | 2.0597 |
| 1370209_at | Klf9 | Kruppel-like factor 9 | 2.0462 |
| 1386935_at | Nr4a1 | nuclear receptor subfamily 4, group A, member 1 | 2.046 |
| 1370820_at | Fbxo6 | F-box protein 6 | 2.045 |
| 1368072_at | Btg3 | B-cell translocation gene 3 | 2.0372 |
| 1377484_at | Fbxl3 | F-box and leucine-rich repeat protein 3 | 2.0351 |
| 1373503_at | Lrch4 | leucine-rich repeats and calponin homology (CH) domain containing 4 | 2.0036 |
| 1381012_at | Serpinf1 | serine (or cysteine) peptidase inhibitor, clade F, member 1 | 2.001 |

|  |  | **All Stem cell specific genes in AMC** |  |  |
| --- | --- | --- | --- | --- |
| **Probe Set ID** | **Gene Symbol** | **Gene Title** | **Fold Change** | **Function** |
| 1390672_at | Rprm | reprimo, TP53 dependent G2 arrest mediator candidate | 19.125 | cell cycle arrest |
| 1373784_at | Cct8 | chaperonin containing Tcp1, subunit 8 (theta) | 13.525 | protein folding |
| 1380407_at | RGD1310352 | similar to HTGN29 protein; keratinocytes associated transmembrane protein 2 | 13.419 | --- |
| 1383747_at | Ect2 | epithelial cell transforming sequence 2 oncogene | 12.498 | cell morphogenesis |
| 1388928_at | Cfl2 | cofilin 2, muscle | 12.211 | protein binding |
| 1383085_at | Sh3bgrl | SH3 domain binding glutamic acid-rich protein like | 9.7435 | --- |
| 1373458_at | Bex4 | brain expressed gene 4 | 9.4991 | --- |
| 1387871_at | Cfl1 /// LOC502589 /// LOC688430 /// RGD1563644 | cofilin 1, non-muscle /// similar to Cofilin-1 (Cofilin, non-muscle isoform) /// similar to Cofilin-1 (Cofilin, non-muscle isoform) /// similar to Cofilin, non-muscle isoform (Cofilin-1) | 9.3235 | cytokinesis |
| 1369953_a_at | Cd24 | CD24 molecule | 9.2544 | response to hypoxia |
| 1388340_at | Ns5atp9 | NS5A (hepatitis C virus) transactivated protein 9 | 9.1968 | --- |
| 1370309_a_at | Hnrnpab | heterogeneous nuclear ribonucleoprotein A/B | 8.8712 | epithelial to mesenchymal transition |
| 1376648_at | Mycn | v-myc myelocytomatosis viral related oncogene, neuroblastoma derived (avian) | 8.6884 | regulation of transcription, DNA-dependent |
| 1370948_a_at | LOC294446 /// LOC681252 /// Marcks | similar to Myristoylated alanine-rich C-kinase substrate (MARCKS) (ACAMP-81) /// similar to Myristoylated alanine-rich C-kinase substrate (MARCKS) (Protein kinase C substrate 80 kDa protein) /// myristoylated alanine rich protein kinase C substrate | 8.5949 | actin binding |
| 1374638_at | Pex13 | peroxisomal biogenesis factor 13 | 8.4726 | fatty acid alpha-oxidation |
| 1370678_s_at | Maoa | monoamine oxidase A | 7.8728 | catecholamine metabolic process |
| 1377772_at | Tmeff1 | transmembrane protein with EGF-like and two follistatin-like domains 1 | 7.7994 | multicellular organismal development |
| 1389534_at | Ube2e3 | ubiquitin-conjugating enzyme E2E 3, UBC4/5 homolog (yeast) | 7.5905 | modification-dependent protein catabolic process |
| 1375338_at | Rab10 | RAB10, member RAS oncogene family | 7.5637 | regulation of transcription, DNA-dependent |
| 1398758_at | Arf4 | ADP-ribosylation factor 4 | 7.3522 | transport |
| 1375525_at | Mapre1 | microtubule-associated protein, RP/EB family, member 1 | 7.0496 | cell cycle |
| 1388582_at | Psme3 | proteasome (prosome, macropain) activator subunit 3 | 7.0045 | cell adhesion |
| 1373595_at | Tmem43 | transmembrane protein 43 | 6.9166 | --- |
| 1390485_at | March5 | membrane-associated ring finger (C3HC4) 5 | 6.9148 | zinc ion binding |
| 1387017_at | Sqle | squalene epoxidase | 6.7699 | cellular aromatic compound metabolic process |
| 1369197_at | Apaf1 | apoptotic peptidase activating factor 1 | 6.6501 | neural tube closure |
| 1370281_at | Fabp5 | fatty acid binding protein 5, epidermal | 6.5852 | glucose metabolic process |
| 1369963_at | Pafah1b3 | platelet-activating factor acetylhydrolase, isoform 1b, subunit 3 | 6.5705 | lipid metabolic process |
| 1368782_at | Sstr2 | somatostatin receptor 2 | 6.5297 | regulation of muscle contraction |
| 1382385_at | Psmc6 | proteasome (prosome, macropain) 26S subunit, ATPase, 6 | 6.4896 | protein catabolic process |
| 1371779_at | Snx6 | sorting nexin 6 | 6.3942 | cell communication |
| 1373022_at | Rab1 | RAB1, member RAS oncogene family | 6.3274 | regulation of transcription, DNA-dependent |
| 1371949_at | Bzw1 | basic leucine zipper and W2 domains 1 | 6.3039 | transcription |
| 1370899_at | Sfpq | splicing factor proline/glutamine rich (polypyrimidine tract binding protein associated) | 6.2932 | nucleotide binding |
| 1399033_at | Cbfb | core-binding factor, beta subunit | 6.0758 | ossification |
| 1387398_at | Pkia | protein kinase (cAMP-dependent, catalytic) inhibitor alpha | 6.0141 | negative regulation of transcription from RNA polymerase II promoter |
| 1387857_at | Stx7 | syntaxin 7 | 5.899 | intracellular protein transport |
| 1373917_at | Etf1 | Eukaryotic translation termination factor 1 | 5.692 | translation |
| 1398888_at | H3f3b | H3 histone, family 3B | 5.6338 | nucleosome assembly |
| 1372209_at | Cops5 | COP9 constitutive photomorphogenic homolog subunit 5 (Arabidopsis) | 5.5306 | protein deneddylation |
| 1390386_at | Casp3 | caspase 3, apoptosis related cysteine protease | 5.3212 | response to hypoxia |
| 1378264_at | Nasp | Nuclear autoantigenic sperm protein (histone-binding) | 5.2995 | blastocyst development |
| 1369954_at | Idh1 | isocitrate dehydrogenase 1 (NADP+), soluble | 5.2005 | glyoxylate cycle |
| 1398250_at | Acot1 | acyl-CoA thioesterase 1 | 5.16 | very-long-chain fatty acid metabolic process |
| 1391127_at | Cdc42 | Cell division cycle 42 (GTP binding protein) | 5.1257 | regulation of mitosis |
| 1375652_at | Ssr3 | signal sequence receptor, gamma | 5.1035 | cotranslational protein targeting to membrane |
| 1369971_a_at | Hnrpd | heterogeneous nuclear ribonucleoprotein D | 5.094 | nuclear-transcribed mRNA catabolic process, exonucleolytic |
| 1383401_at | Tes | testis derived transcript | 5.0625 | zinc ion binding |
| 1372462_at | Acat2 | acetyl-Coenzyme A acetyltransferase 2 | 5.0498 | metabolic process |
| 1375337_at | Adam9 | ADAM metallopeptidase domain 9 (meltrin gamma) | 5.0154 | activation of MAPKK activity |
| 1384465_at | Pspc1 | paraspeckle component 1 | 5.0087 | transcription |
| 1388378_at | Eif3c | eukaryotic translation initiation factor 3, subunit C | 4.9591 | translation |
| 1398756_at | LOC300303 /// Npm1 | similar to Nucleophosmin (NPM) (Nucleolar phosphoprotein B23) (Numatrin) (Nucleolar protein NO38) /// nucleophosmin (nucleolar phosphoprotein B23, numatrin) | 4.9399 | ribosomal large subunit export from nucleus |
| 1368175_at | Zhx1 | zinc fingers and homeoboxes 1 | 4.9074 | transcription |
| 1390489_at | Syncrip | Synaptotagmin binding, cytoplasmic RNA interacting protein | 4.8936 | mRNA processing |
| 1388581_at | Hn1 | hematological and neurological expressed 1 | 4.8833 | --- |
| 1386882_at | Dynlt1 | dynein light chain Tctex-type 1 | 4.8568 | microtubule-based process |
| 1382680_at | Adfp | Adipose differentiation related protein | 4.8553 | response to organic cyclic substance |
| 1367768_at | Lxn | latexin | 4.764 | detection of temperature stimulus involved in sensory perception of pain |
| 1372575_at | Wbp11 | WW domain binding protein 11 | 4.7604 | rRNA processing |
| 1368214_at | Smad2 | SMAD family member 2 | 4.7514 | in utero embryonic development |
| 1388996_at | Hdhd2 | haloacid dehalogenase-like hydrolase domain containing 2 | 4.7252 | metabolic process |
| 1388712_at | Cyth3 | cytohesin 3 | 4.7187 | regulation of ARF protein signal transduction |
| 1371418_at | Cct2 | chaperonin containing TCP1, subunit 2 (beta) | 4.711 | protein folding |
| 1389226_at | Stag1 | stromal antigen 1 | 4.6871 | protein binding |
| 1375424_at | Actr2 | ARP2 actin-related protein 2 homolog (yeast) | 4.6831 | nucleotide binding |
| 1388755_at | Sec23a | Sec23 homolog A (S. cerevisiae) | 4.6661 | transport |
| 1378178_at | Smc6l1 | SMC6 structural maintenance of chromosomes 6-like 1 (yeast) | 4.65 | ATP binding |
| 1372116_at | Mrps2 | mitochondrial ribosomal protein S2 | 4.6279 | --- |
| 1398903_at | Esd | esterase D/formylglutathione hydrolase | 4.616 | carboxylesterase activity |
| 1368869_at | Akap12 | A kinase (PRKA) anchor protein 12 | 4.5495 | protein targeting |
| 1371653_at | Tpm4 | Tropomyosin 4 | 4.5474 | muscle contraction |
| 1387275_at | Sox11 | SRY (sex determining region Y)-box 11 | 4.5022 | kidney development |
| 1376727_at | Yipf4 | Yip1 domain family, member 4 | 4.4731 | --- |
| 1374625_at | Hes6 | hairy and enhancer of split 6 (Drosophila) | 4.4412 | transcription |
| 1372566_at | Mtch2 | mitochondrial carrier homolog 2 (C. elegans) | 4.4249 | transport |
| 1399119_at | Znf292 | zinc finger protein 292 | 4.3921 | transcription factor binding |
| 1367631_at | Ctgf | connective tissue growth factor | 4.374 | cartilage condensation |
| 1367613_at | Prdx1 | peroxiredoxin 1 | 4.3624 | response to reactive oxygen species |
| 1370959_at | Col3a1 | collagen, type III, alpha 1 | 4.3557 | skeletal system development |
| 1398814_at | Rab11a | RAB11a, member RAS oncogene family | 4.33 | GTP catabolic process |
| 1389480_at | Rwdd4a | RWD domain containing 4A | 4.3289 | --- |
| 1398350_at | Basp1 | brain abundant, membrane attached signal protein 1 | 4.3051 | calmodulin binding |
| 1367485_at | Tcea1 | transcription elongation factor A (SII) 1 | 4.3035 | transcription |
| 1367729_at | Oat | ornithine aminotransferase (gyrate atrophy) | 4.2756 | ornithine metabolic process |
| 1388196_at | Nckap1 | NCK-associated protein 1 | 4.268 | in utero embryonic development |
| 1373846_at | Btf3l4 | basic transcription factor 3-like 4 | 4.2585 | --- |
| 1369103_at | Fyn | FYN oncogene related to SRC, FGR, YES | 4.249 | neuron migration |
| 1367731_at | Gnb1 | guanine nucleotide binding protein (G protein), beta polypeptide 1 | 4.2345 | signal transduction |
| 1367488_at | Ankrd17 | ankyrin repeat domain 17 | 4.2225 | mismatch repair |
| 1387806_at | Rap1b | RAP1B, member of RAS oncogene family | 4.2206 | signal transduction |
| 1367683_at | Kpna2 | karyopherin (importin) alpha 2 | 4.216 | protein import into nucleus |
| 1375538_at | Vcl | Vinculin | 4.2035 | cell adhesion |
| 1371352_at | Hmgn2 | high mobility group nucleosomal binding domain 2 | 4.1954 | DNA binding |
| 1367890_at | Casp2 | caspase 2 | 4.1832 | proteolysis |
| 1386065_at | Ankrd57 | ankyrin repeat domain 57 | 4.1786 | --- |
| 1377676_at | Nucks1 | nuclear casein kinase and cyclin-dependent kinase substrate 1 | 4.1448 | regulation of cell cycle |
| 1391201_at | Wdhd1 | WD repeat and HMG-box DNA binding protein 1 | 4.1387 | --- |
| 1368852_at | Dnaja1 | DnaJ (Hsp40) homolog, subfamily A, member 1 | 4.1353 | protein folding |
| 1384068_at | Ckap2 | cytoskeleton associated protein 2 | 4.1187 | --- |
| 1377097_at | Cox6b2 | cytochrome c oxidase subunit VIb polypeptide 2 | 4.1067 | cytochrome-c oxidase activity |
| 1371445_at | Lrrc59 | leucine rich repeat containing 59 | 4.0964 | protein binding |
| 1383156_at | Kif2a | kinesin family member 2A | 4.0729 | microtubule-based movement |
| 1375259_at | Eif4ebp2 | eukaryotic translation initiation factor 4E binding protein 2 | 4.0525 | insulin receptor signaling pathway |
| 1385765_at | Lin9 /// LOC360888 | lin-9 homolog (C. elegans) /// similar to lin-9 homolog (C. elegans) | 4.0522 | --- |
| 1370869_at | Bcat1 | branched chain aminotransferase 1, cytosolic | 4.0145 | metabolic process |
| 1373769_at | Pgm2 | phosphoglucomutase 2 | 4.0142 | carbohydrate metabolic process |
| 1372671_at | Rfk | riboflavin kinase | 3.9891 | riboflavin biosynthetic process |
| 1383804_at | Dmrtb1 | DMRT-like family B with proline-rich C-terminal, 1 | 3.9872 | regulation of transcription, DNA-dependent |
| 1386683_at | Atrx | alpha thalassemia/mental retardation syndrome X-linked (RAD54 homolog, S. cerevisiae) | 3.9841 | DNA repair |
| 1386394_at | Prps1 | phosphoribosyl pyrophosphate synthetase 1 | 3.9782 | purine base metabolic process |
| 1368042_a_at | Hmg1l1 /// Hmgb1 /// LOC678705 /// LOC681718 /// RGD1562312 /// RGD1563012 /// RGD1563786 | high-mobility group (nonhistone chromosomal) protein 1-like 1 /// high mobility group box 1 /// hypothetical protein LOC678705 /// similar to High mobility group protein 1 (HMG-1) (High mobility group protein B1) (Amphoterin) (Heparin-binding protein p30) /// similar to High mobility group protein 1 (HMG-1) /// similar to High mobility group protein 1 (HMG-1) /// similar to Hmgb1 protein | 3.9596 | cell morphogenesis |
| 1370258_at | Bzw2 | basic leucine zipper and W2 domains 2 | 3.9572 | multicellular organismal development |
| 1368122_at | Rnf103 | ring finger protein 103 | 3.9452 | protein binding |
| 1398773_at | Khdrbs1 | KH domain containing, RNA binding, signal transduction associated 1 | 3.9389 | G2/M transition of mitotic cell cycle |
| 1382146_at | Tspan6 | tetraspanin 6 | 3.9366 | positive regulation of I-kappaB kinase/NF-kappaB cascade |
| 1388643_at | Fut8 | fucosyltransferase 8 (alpha (1,6) fucosyltransferase) | 3.9358 | transforming growth factor beta receptor signaling pathway |
| 1368234_at | Prep | prolyl endopeptidase | 3.9299 | proteolysis |
| 1391387_s_at | Slbp | stem-loop binding protein | 3.9227 | histone mRNA 3'-end processing |
| 1370524_at | Ube2d2 | Ubiquitin-conjugating enzyme E2D 2 | 3.9077 | protein ubiquitination |
| 1399160_a_at | Ube2d3 | ubiquitin-conjugating enzyme E2D 3 (UBC4/5 homolog, yeast) | 3.8946 | ubiquitin-dependent protein catabolic process |
| 1370890_at | Actr3 | ARP3 actin-related protein 3 homolog (yeast) | 3.8936 | response to carbohydrate stimulus |
| 1367834_at | Srm | spermidine synthase | 3.8919 | spermidine biosynthetic process |
| 1398262_at | Prps2 | phosphoribosyl pyrophosphate synthetase 2 | 3.8814 | AMP biosynthetic process |
| 1385248_a_at | Ogn | osteoglycin | 3.8731 | protein binding |
| 1368878_at | Idi1 | isopentenyl-diphosphate delta isomerase 1 | 3.8687 | steroid biosynthetic process |
| 1383766_at | Sgcb | sarcoglycan, beta (dystrophin-associated glycoprotein) | 3.8373 | cytoskeleton organization |
| 1368308_at | Myc | myelocytomatosis oncogene | 3.835 | B cell apoptosis |
| 1386908_at | Glrx1 | glutaredoxin 1 (thioltransferase) | 3.8276 | transport |
| 1388562_at | Stard7 | StAR-related lipid transfer (START) domain containing 7 | 3.8204 | --- |
| 1398874_at | Atxn10 | ataxin 10 | 3.8086 | nervous system development |
| 1369952_at | Pabpc1 | poly(A) binding protein, cytoplasmic 1 | 3.7796 | mRNA processing |
| 1368642_at | Cdh2 | cadherin 2 | 3.7671 | cell adhesion |
| 1389496_at | Akap7 | A kinase (PRKA) anchor protein 7 | 3.7522 | protein localization |
| 1368582_at | Slc7a3 | solute carrier family 7 (cationic amino acid transporter, y+ system), member 3 | 3.7468 | transport |
| 1376606_a_at | Eny2 | enhancer of yellow 2 homolog (Drosophila) | 3.7453 | histone deubiquitination |
| 1391415_at | Pno1 | partner of NOB1 homolog (S. cerevisiae) | 3.7362 | RNA binding |
| 1390802_at | RGD1306839 | similar to RIKEN cDNA 5033414D02 | 3.7345 | --- |
| 1370213_at | Ybx1 | Y box binding protein 1 | 3.7191 | negative regulation of transcription from RNA polymerase II promoter |
| 1382099_at | Vps26a | vacuolar protein sorting 26 homolog A (S. pombe) | 3.7143 | transport |
| 1375889_at | Sms | spermine synthase | 3.7039 | spermine biosynthetic process |
| 1368189_at | Dhcr7 | 7-dehydrocholesterol reductase | 3.6913 | blood vessel development |
| 1367663_at | Psme1 | proteasome (prosome, macropain) activator subunit 1 | 3.6823 | antigen processing and presentation of exogenous antigen |
| 1385874_at | Usp42 | Ubiquitin specific peptidase 42 | 3.6708 | --- |
| 1375119_at | Nedd4 | neural precursor cell expressed, developmentally down-regulated gene 4 | 3.6588 | protein modification process |
| 1369972_at | Fbxo21 | F-box protein 21 | 3.6568 | serine-type endopeptidase inhibitor activity |
| 1398836_s_at | Actb | actin, beta | 3.6424 | axonogenesis |
| 1381153_at | Anapc4 | anaphase promoting complex subunit 4 | 3.5894 | anaphase-promoting complex-dependent proteasomal ubiquitin-dependent protein catabolic process |
| 1376065_at | Rrs1 | RRS1 ribosome biogenesis regulator homolog (S. cerevisiae) | 3.571 | ribosome biogenesis |
| 1389968_at | Eif3s10 | eukaryotic translation initiation factor 3, subunit 10 (theta) | 3.5688 | formation of translation initiation complex |
| 1373408_at | Tbca | tubulin folding cofactor A | 3.5647 | tubulin complex assembly |
| 1388771_at | Cggbp1 | CGG triplet repeat binding protein 1 | 3.5626 | --- |
| 1393242_at | RGD1310922 | similar to chromosome 16 open reading frame 33; minus -99 protein | 3.5563 | --- |
| 1369642_at | Pafah1b2 | platelet-activating factor acetylhydrolase, isoform 1b, subunit 2 | 3.5476 | lipid metabolic process |
| 1371962_at | Tufm | Tu translation elongation factor, mitochondrial | 3.546 | translation |
| 1367832_at | Lypla1 | lysophospholipase 1 | 3.5409 | lipid metabolic process |
| 1390272_at | Dph5 | DPH5 homolog (S. cerevisiae) | 3.5354 | metabolic process |
| 1368029_at | Gnai3 | guanine nucleotide binding protein (G protein), alpha inhibiting 3 | 3.5186 | vesicle fusion |
| 1377163_at | Inhbb | Inhibin beta-B | 3.4699 | response to external stimulus |
| 1398978_at | Ap1g1 | Adaptor-related protein complex 1, gamma 1 subunit | 3.4678 | microtubule cytoskeleton organization |
| 1373418_at | Eprs | glutamyl-prolyl-tRNA synthetase | 3.4617 | translation |
| 1398904_at | Nono | non-POU domain containing, octamer-binding | 3.4584 | DNA repair |
| 1371777_at | Pabpc4 | poly A binding protein, cytoplasmic 4 | 3.4559 | nucleotide binding |
| 1374473_at | Ppp1r15b | protein phosphatase 1, regulatory (inhibitor) subunit 15b | 3.4508 | response to stress |
| 1373080_at | Papola | poly (A) polymerase alpha | 3.4484 | nuclear mRNA splicing, via spliceosome |
| 1398797_at | Hnrnpk | heterogeneous nuclear ribonucleoprotein K | 3.4439 | nuclear mRNA splicing, via spliceosome |
| 1390022_at | Arpc5 | actin related protein 2/3 complex, subunit 5 | 3.4395 | regulation of actin filament polymerization |
| 1371939_at | Caprin1 | cell cycle associated protein 1 | 3.4351 | protein binding |
| 1388679_at | Tbc1d14 | TBC1 domain family, member 14 | 3.4342 | regulation of Rab GTPase activity |
| 1388119_at | Hnrnpa3 | heterogeneous nuclear ribonucleoprotein A3 | 3.4339 | nuclear mRNA splicing, via spliceosome |
| 1398439_a_at | Orc6l | origin recognition complex, subunit 6 like (yeast) | 3.4312 | DNA replication |
| 1388631_at | Azi2 | 5-azacytidine induced 2 | 3.4308 | I-kappaB kinase/NF-kappaB cascade |
| 1371945_at | Ube2l3 | ubiquitin-conjugating enzyme E2L 3 | 3.4273 | protein ubiquitination |
| 1388528_at | Fbl | fibrillarin | 3.4202 | rRNA processing |
| 1371928_at | Cdca8 | cell division cycle associated 8 | 3.4068 | mitotic metaphase |
| 1376143_at | Ddx54 | DEAD (Asp-Glu-Ala-Asp) box polypeptide 54 | 3.4002 | RNA processing |
| 1370188_at | Sfrs10 | splicing factor, arginine/serine-rich 10 (transformer 2 homolog, Drosophila) | 3.3884 | response to reactive oxygen species |
| 1380443_at | Pwp1 | PWP1 homolog (S. cerevisiae) | 3.3871 | --- |
| 1386096_at | Mtf2 | metal response element binding transcription factor 2 | 3.3769 | nucleic acid binding |
| 1373894_at | Rab31 | RAB31, member RAS oncogene family | 3.3714 | small GTPase mediated signal transduction |
| 1367469_at | Eif4g2 /// Eif4g2-ps1 /// LOC678831 | eukaryotic translation initiation factor 4, gamma 2 /// eukaryotic translation initiation factor 4, gamma 2, pseudogene 1 /// similar to eukaryotic translation initiation factor 4 gamma, 2 | 3.3536 | regulation of translational initiation |
| 1367586_at | Ldha | lactate dehydrogenase A | 3.3412 | response to hypoxia |
| 1378320_at | Rlbp1 | retinaldehyde binding protein 1 | 3.341 | 11-cis retinal binding |
| 1390692_at | Ctps | CTP synthase | 3.3248 | pyrimidine nucleotide biosynthetic process |
| 1387921_at | Zc3h14 | zinc finger CCCH type containing 14 | 3.3198 | nucleic acid binding |
| 1370250_at | Ube2i | ubiquitin-conjugating enzyme E2I | 3.3128 | ubiquitin-dependent protein catabolic process |
| 1374571_at | Pigx | phosphatidylinositol glycan anchor biosynthesis, class X | 3.3109 | GPI anchor biosynthetic process |
| 1387863_at | Csde1 | cold shock domain containing E1, RNA binding | 3.2985 | regulation of transcription, DNA-dependent |
| 1383339_at | RGD1310358 | similar to NNX3 | 3.2941 | regulation of transcription from RNA polymerase II promoter |
| 1379582_a_at | Ccna2 | cyclin A2 | 3.2889 | cell cycle |
| 1374518_at | Tmem77 | transmembrane protein 77 | 3.2873 | --- |
| 1389292_at | Rab18 | RAB18, member RAS oncogene family | 3.273 | regulation of transcription, DNA-dependent |
| 1394591_at | Zfp207 | zinc finger protein 207 | 3.2727 | zinc ion binding |
| 1373090_at | Ssr1 | signal sequence receptor, alpha | 3.2686 | receptor activity |
| 1367935_at | Smu1 | smu-1 suppressor of mec-8 and unc-52 homolog (C. elegans) | 3.267 | --- |
| 1367590_at | Ran | RAN, member RAS oncogene family | 3.2657 | microtubule cytoskeleton organization |
| 1385157_at | Cbx1 | chromobox homolog 1 (HP1 beta homolog Drosophila ) | 3.2536 | chromatin assembly or disassembly |
| 1386982_at | Mgat2 | mannosyl (alpha-1,6-)-glycoprotein beta-1,2-N-acetylglucosaminyltransferase | 3.2471 | protein amino acid N-linked glycosylation |
| 1373024_at | Ap3s1 | adaptor-related protein complex 3, sigma 1 subunit | 3.2373 | transport |
| 1398810_at | Pdap1 | PDGFA associated protein 1 | 3.2303 | --- |
| 1378127_at | Cul2 | cullin 2 | 3.2253 | ubiquitin-dependent protein catabolic process |
| 1367728_at | Tsn | translin | 3.2222 | RNA binding |
| 1371375_at | Dstn | destrin | 3.2147 | actin binding |
| 1389566_at | Ccnb2 | cyclin B2 | 3.2038 | in utero embryonic development |
| 1383075_at | Ccnd1 | cyclin D1 | 3.201 | G1/S transition of mitotic cell cycle |
| 1371960_at | Ythdf2 | YTH domain family, member 2 | 3.1958 | --- |
| 1370357_at | Slc30a4 | solute carrier family 30 (zinc transporter), member 4 | 3.1945 | transport |
| 1368405_at | Rala | v-ral simian leukemia viral oncogene homolog A (ras related) | 3.1901 | signal transduction |
| 1393247_at | Zfp26 | zinc finger protein 26 | 3.1901 | transcription |
| 1398786_at | Psmb2 | proteasome (prosome, macropain) subunit, beta type 2 | 3.1812 | response to organic nitrogen |
| 1398757_at | Npm1 | nucleophosmin (nucleolar phosphoprotein B23, numatrin) | 3.1793 | ribosomal large subunit export from nucleus |
| 1389857_at | Wbp5 | WW domain binding protein 5 | 3.1767 | protein binding |
| 1393407_at | Fbxw2 | F-box and WD repeat domain containing 2 | 3.176 | protein binding |
| 1370003_at | Eef2 | eukaryotic translation elongation factor 2 | 3.1679 | translation |
| 1385118_at | Eif2s1 /// LOC364604 /// LOC364984 | eukaryotic translation initiation factor 2, subunit 1 alpha /// hypothetical gene supported by NM_019356 /// hypothetical gene supported by NM_019356 | 3.1664 | translation |
| 1374840_at | RGD1564921 | similar to peptidyl prolyl isomerase H | 3.1651 | peptidyl-prolyl cis-trans isomerase activity |
| 1381814_at | Srgap2 | SLIT-ROBO Rho GTPase activating protein 2 | 3.147 | signal transduction |
| 1389791_at | Cln8 | ceroid-lipofuscinosis, neuronal 8 | 3.1431 | age-dependent response to oxidative stress |
| 1383269_at | Rnf2 | ring finger protein 2 | 3.1366 | negative regulation of transcription from RNA polymerase II promoter |
| 1367759_at | H1f0 | H1 histone family, member 0 | 3.1325 | nucleosome assembly |
| 1371851_at | Psmd6 | proteasome (prosome, macropain) 26S subunit, non-ATPase, 6 | 3.1318 | anaphase-promoting complex-dependent proteasomal ubiquitin-dependent protein catabolic process |
| 1388865_at | Ppp4r2 | protein phosphatase 4, regulatory subunit 2 | 3.1299 | protein binding |
| 1370909_at | Nup62 | nucleoporin 62 | 3.128 | transport |
| 1387025_at | Dync1i1 | dynein cytoplasmic 1 intermediate chain 1 | 3.124 | vesicle transport along microtubule |
| 1368002_at | Msh2 | mutS homolog 2 (E. coli) | 3.1126 | in utero embryonic development |
| 1389309_at | Sbno1 | strawberry notch homolog 1 (Drosophila) | 3.1109 | --- |
| 1374747_at | Pftk1 | PFTAIRE protein kinase 1 | 3.0991 | protein kinase activity |
| 1388483_at | Cfl2 | cofilin 2, muscle | 3.0851 | protein binding |
| 1370908_at | Hdac2 | histone deacetylase 2 | 3.0713 | positive regulation of cell proliferation |
| 1367713_at | Eif2s1 | eukaryotic translation initiation factor 2, subunit 1 alpha | 3.0664 | translation |
| 1388126_at | Minpp1 | multiple inositol polyphosphate histidine phosphatase 1 | 3.063 | phosphoinositide-mediated signaling |
| 1388330_at | Vkorc1 | vitamin K epoxide reductase complex, subunit 1 | 3.0627 | response to organic nitrogen |
| 1388195_at | Cugbp2 | CUG triplet repeat, RNA binding protein 2 | 3.0617 | mRNA splice site selection |
| 1377850_at | Znrf1 | zinc and ring finger 1 | 3.0433 | --- |
| 1367515_at | Cnot7 | CCR4-NOT transcription complex, subunit 7 | 3.0397 | positive regulation of transcription from RNA polymerase II promoter |
| 1391347_at | Rab8b | RAB8B, member RAS oncogene family | 3.0307 | transport |
| 1389296_at | Ttc35 | tetratricopeptide repeat domain 35 | 3.0273 | binding |
| 1398784_at | C1qbp | complement component 1, q subcomponent binding protein | 3.012 | immune response |
| 1389528_s_at | Jun | Jun oncogene | 3.0095 | angiogenesis |
| 1380202_at | Znf821 | zinc finger protein 821 | 2.9973 | zinc ion binding |
| 1367938_at | Ugdh | UDP-glucose dehydrogenase | 2.9758 | gastrulation with mouth forming second |
| 1387154_at | Npy | neuropeptide Y | 2.9739 | neuropeptide signaling pathway |
| 1394854_at | Terf1 | telomeric repeat binding factor (NIMA-interacting) 1 | 2.9664 | telomere maintenance via telomerase |
| 1371982_at | Dpy30 | dpy-30 homolog (C. elegans) | 2.9468 | identical protein binding |
| 1394316_a_at | Tspan5 | tetraspanin 5 | 2.9458 | cell adhesion |
| 1374565_at | Nek6 | NIMA (never in mitosis gene a)-related kinase 6 | 2.9406 | protein amino acid phosphorylation |
| 1398839_at | Txn1 | thioredoxin 1 | 2.9385 | transport |
| 1371544_at | Erh | enhancer of rudimentary homolog (Drosophila) | 2.937 | protein binding |
| 1379583_at | Afg3l1 | AFG3(ATPase family gene 3)-like 1 (S. cerevisiae) | 2.926 | proteolysis |
| 1389162_at | Nfu1 | NFU1 iron-sulfur cluster scaffold homolog (S. cerevisiae) | 2.9234 | iron-sulfur cluster assembly |
| 1374067_at | RGD1308127 | similar to 2700078E11Rik protein | 2.9173 | ubiquitin-dependent protein catabolic process |
| 1372460_at | LOC502130 /// RGD1562760 /// Set | similar to SET protein (Phosphatase 2A inhibitor I2PP2A) (I-2PP2A) (Template-activating factor I) (TAF-I) (Liver regeneration-related protein LRRGR00002) /// RGD1562760 /// SET nuclear oncogene | 2.9153 | nucleosome assembly |
| 1389293_at | Cpsf2 | cleavage and polyadenylation specific factor 2 | 2.9147 | nuclear mRNA splicing, via spliceosome |
| 1398778_at | Psma1 | proteasome (prosome, macropain) subunit, alpha type 1 | 2.9127 | ubiquitin-dependent protein catabolic process |
| 1374660_at | Nsmce4a | non-SMC element 4 homolog A (S. cerevisiae) | 2.9057 | --- |
| 1398424_at | Wsb2 | WD repeat and SOCS box-containing 2 | 2.8979 | intracellular signaling cascade |
| 1387060_at | Klf6 | Kruppel-like factor 6 | 2.8977 | transcription |
| 1373267_at | Sh3yl1 | SH3 domain containing, Ysc84-like 1 (S. cerevisiae) | 2.8858 | protein binding |
| 1387856_at | Cnn3 | calponin 3, acidic | 2.8817 | actomyosin structure organization |
| 1367939_at | Rbp1 | retinol binding protein 1, cellular | 2.88 | transport |
| 1389655_at | Ptrh2 | peptidyl-tRNA hydrolase 2 | 2.8778 | translation |
| 1397363_at | Pvrl3 | Poliovirus receptor-related 3 | 2.8757 | --- |
| 1371518_at | Nid1 | Nidogen 1 | 2.8619 | cell adhesion |
| 1371437_at | Sec13 | SEC13 homolog (S. cerevisiae) | 2.8556 | transport |
| 1388810_at | Abce1 | ATP-binding cassette, sub-family E (OABP), member 1 | 2.8519 | nucleotide binding |
| 1373273_at | Prpf38a | PRP38 pre-mRNA processing factor 38 (yeast) domain containing A | 2.85 | --- |
| 1382419_at | Cenpk | centromere protein K | 2.8488 | positive regulation of transcription from RNA polymerase II promoter |
| 1388428_at | Dtd1 | D-tyrosyl-tRNA deacylase 1 homolog (S. cerevisiae) | 2.8436 | D-amino acid catabolic process |
| 1371360_at | Ndrg1 | N-myc downstream regulated gene 1 | 2.8387 | mast cell activation |
| 1372558_at | Narg1 | NMDA receptor regulated 1 | 2.8345 | N-terminal protein amino acid acetylation |
| 1367656_at | Psmb7 | proteasome (prosome, macropain) subunit, beta type 7 | 2.8327 | proteolysis involved in cellular protein catabolic process |
| 1379364_at | Camsap1 | calmodulin regulated spectrin-associated protein 1 | 2.8297 | --- |
| 1372173_at | RGD1309198 | similar to U5 snRNP-specific protein (Prp8-binding) | 2.8262 | --- |
| 1388514_at | Ppm1g | protein phosphatase 1G (formerly 2C), magnesium-dependent, gamma isoform | 2.8138 | protein amino acid dephosphorylation |
| 1398896_at | Arcn1 | archain 1 | 2.8136 | transport |
| 1385090_at | Rad17 | RAD17 homolog (S. pombe) | 2.8077 | DNA damage checkpoint |
| 1373380_at | Zc3h15 | zinc finger CCCH-type containing 15 | 2.7981 | cytokine-mediated signaling pathway |
| 1368508_at | Psma3 /// Psma3l | proteasome (prosome, macropain) subunit, alpha type 3 /// proteasome subunit alpha type 3-like | 2.7971 | ubiquitin-dependent protein catabolic process |
| 1373063_at | Ube2k | ubiquitin-conjugating enzyme E2K (UBC1 homolog, yeast) | 2.797 | post-translational protein modification |
| 1388802_at | Bex1 | brain expressed gene 1 | 2.7963 | positive regulation of neuroblast proliferation |
| 1389575_at | RGD1311703 | similar to sid2057p | 2.7805 | --- |
| 1367734_at | Akr1b1 | aldo-keto reductase family 1, member B1 (aldose reductase) | 2.779 | sorbitol biosynthetic process |
| 1372763_at | Nus1 | nuclear undecaprenyl pyrophosphate synthase 1 homolog (S. cerevisiae) | 2.7777 | transferase activity, transferring alkyl or aryl (other than methyl) groups |
| 1371310_s_at | Serpinh1 | serine (or cysteine) peptidase inhibitor, clade H, member 1 | 2.7604 | response to stress |
| 1389081_at | Vps37b | vacuolar protein sorting 37 homolog B (S. cerevisiae) | 2.7574 | --- |
| 1398768_at | Rbbp7 | retinoblastoma binding protein 7 | 2.7533 | negative regulation of transcription from RNA polymerase II promoter |
| 1393412_at | Fam3c | family with sequence similarity 3, member C | 2.7499 | --- |
| 1372303_at | Fam49b | family with sequence similarity 49, member B | 2.7477 | --- |
| 1368450_at | Myo5a | myosin Va | 2.7463 | melanin metabolic process |
| 1384280_at | Nusap1 | nucleolar and spindle associated protein 1 | 2.7393 | cytokinesis after mitosis |
| 1398780_at | Rabac1 | Rab acceptor 1 (prenylated) | 2.7357 | vesicle-mediated transport |
| 1373048_at | Actr10 | actin-related protein 10 homolog (S. cerevisiae) | 2.7331 | microtubule-based movement |
| 1398813_at | Uba3 | ubiquitin-like modifier activating enzyme 3 | 2.7254 | mitotic cell cycle |
| 1390420_at | Cpxm1 | carboxypeptidase X (M14 family), member 1 | 2.7249 | proteolysis |
| 1388975_at | Sucla2 | succinate-CoA ligase, ADP-forming, beta subunit | 2.7181 | tricarboxylic acid cycle |
| 1390650_at | Nup85 | nucleoporin 85kDa | 2.718 | transport |
| 1393041_at | Smc2 | structural maintenance of chromosomes 2 | 2.7164 | mitotic chromosome condensation |
| 1388715_at | Gars | glycyl-tRNA synthetase | 2.7116 | translation |
| 1374595_at | Tnks2 | tankyrase, TRF1-interacting ankyrin-related ADP-ribose polymerase 2 | 2.6977 | telomere maintenance |
| 1393173_at | Rasa1 | RAS p21 protein activator (GTPase activating protein) 1 | 2.6875 | cytokinesis |
| 1389546_at | Amotl2 | angiomotin like 2 | 2.6859 | protein binding |
| 1388493_at | Ecop | EGFR-coamplified and overexpressed protein | 2.6853 | --- |
| 1390115_at | Sec63 | SEC63 homolog (S. cerevisiae) | 2.6844 | --- |
| 1398919_at | RGD1304704 | similar to Hypothetical protein CGI-99 | 2.6844 | identical protein binding |
| 1385153_at | Zfp68 | zinc finger protein 68 | 2.6835 | regulation of transcription, DNA-dependent |
| 1380168_at | Etv4 | ets variant 4 | 2.6701 | regulation of transcription, DNA-dependent |
| 1398803_at | Dync1h1 | dynein cytoplasmic 1 heavy chain 1 | 2.6624 | microtubule-based movement |
| 1395045_at | Ndufa7 | NADH dehydrogenase (ubiquinone) 1 alpha subcomplex, 7 (B14.5a) | 2.6513 | ATP synthesis coupled electron transport |
| 1372143_at | Ube2v2 | ubiquitin-conjugating enzyme E2 variant 2 | 2.6433 | DNA double-strand break processing |
| 1370282_at | Csrp2 | cysteine and glycine-rich protein 2 | 2.641 | multicellular organismal development |
| 1392698_a_at | Gtf3c4 | general transcription factor IIIC, polypeptide 4 | 2.6396 | protein binding |
| 1371818_at | Xpot | Exportin, tRNA (nuclear export receptor for tRNAs) | 2.633 | tRNA export from nucleus |
| 1388960_at | Ppa1 | pyrophosphatase (inorganic) 1 | 2.6264 | phosphate metabolic process |
| 1373365_at | Cmpk1 | cytidine monophosphate (UMP-CMP) kinase 1, cytosolic | 2.6227 | nucleobase, nucleoside, nucleotide and nucleic acid metabolic process |
| 1383572_at | Zdhhc6 | zinc finger, DHHC domain containing 6 | 2.6184 | zinc ion binding |
| 1394079_at | Tbl1xr1 | transducin (beta)-like 1 X-linked receptor 1 | 2.6107 | proteasomal ubiquitin-dependent protein catabolic process |
| 1388504_at | Rad21 | RAD21 homolog (S. pombe) | 2.6069 | protein binding |
| 1383160_at | Chordc1 | cysteine and histidine-rich domain (CHORD)-containing 1 | 2.6046 | calcium ion binding |
| 1384615_at | Crop | Cisplatin resistance-associated overexpressed protein | 2.596 | apoptosis |
| 1371553_at | Mrpl36 | mitochondrial ribosomal protein L36 | 2.5912 | translation |
| 1372720_at | Btbd1 | BTB (POZ) domain containing 1 | 2.5874 | protein binding |
| 1383126_at | Akt1 | V-akt murine thymoma viral oncogene homolog 1 | 2.5848 | protein import into nucleus, translocation |
| 1370344_at | Hspa4 | heat shock protein 4 | 2.5818 | response to stress |
| 1386897_at | Prmt1 | protein arginine methyltransferase 1 | 2.5686 | in utero embryonic development |
| 1387105_at | Zfp422 | zinc finger protein 422 | 2.5674 | transcription |
| 1387280_a_at | Slc7a5 | solute carrier family 7 (cationic amino acid transporter, y+ system), member 5 | 2.5654 | transport |
| 1398831_at | Psmb4 | proteasome (prosome, macropain) subunit, beta type 4 | 2.5649 | anaphase-promoting complex-dependent proteasomal ubiquitin-dependent protein catabolic process |
| 1377023_at | Dusp2 | dual specificity phosphatase 2 | 2.5647 | protein amino acid dephosphorylation |
| 1374569_at | Grwd1 | glutamate-rich WD repeat containing 1 | 2.5531 | --- |
| 1389091_at | Usp3 | ubiquitin specific peptidase 3 | 2.5434 | ubiquitin-dependent protein catabolic process |
| 1383736_at | Elavl2 | ELAV (embryonic lethal, abnormal vision, Drosophila)-like 2 (Hu antigen B) | 2.5411 | nucleotide binding |
| 1373946_at | Armc10 | armadillo repeat containing 10 | 2.5382 | regulation of growth |
| 1376468_at | Hars | histidyl-tRNA synthetase | 2.5271 | translation |
| 1372391_at | Rpl7l1 | ribosomal protein L7-like 1 | 2.5126 | translation |
| 1371947_at | Ndn | necdin homolog (mouse) | 2.5112 | neuron migration |
| 1390647_at | Phtf2 | putative homeodomain transcription factor 2 | 2.5097 | --- |
| 1367580_at | Rpl10a | ribosomal protein L10A | 2.5057 | RNA processing |
| 1368273_at | Mapk6 | mitogen-activated protein kinase 6 | 2.5004 | protein amino acid phosphorylation |
| 1371463_at | Phf5a | PHD finger protein 5A | 2.4986 | nuclear mRNA splicing, via spliceosome |
| 1368032_at | Nolc1 | nucleolar and coiled-body phosphoprotein 1 | 2.4885 | response to osmotic stress |
| 1374549_at | Rbm4b | RNA binding motif protein 4B | 2.4738 | mRNA processing |
| 1373060_at | Chmp2b | chromatin modifying protein 2B | 2.4693 | protein transport |
| 1375631_at | Plekhb2 | pleckstrin homology domain containing, family B (evectins) member 2 | 2.4672 | protein binding |
| 1370813_at | Gstm5 | glutathione S-transferase, mu 5 | 2.4582 | metabolic process |
| 1386967_at | Rhoq | ras homolog gene family, member Q | 2.458 | GTP catabolic process |
| 1371436_at | Ddah2 | dimethylarginine dimethylaminohydrolase 2 | 2.4578 | arginine metabolic process |
| 1398328_at | Foxk2 | forkhead box K2 | 2.4537 | regulation of transcription, DNA-dependent |
| 1386916_at | Aco1 | aconitase 1, soluble | 2.4529 | tricarboxylic acid cycle |
| 1388382_at | LOC361985 | similar to NICE-3 | 2.4499 | --- |
| 1398763_at | Timm23 | translocase of inner mitochondrial membrane 23 homolog (yeast) | 2.4471 | transport |
| 1387201_at | Rnf138 | ring finger protein 138 | 2.4469 | Wnt receptor signaling pathway |
| 1387950_at | Nip7 | nuclear import 7 homolog (S. cerevisiae) | 2.4468 | ribosome biogenesis |
| 1382161_at | Mphosph10 | M-phase phosphoprotein 10 (U3 small nucleolar ribonucleoprotein) | 2.445 | rRNA processing |
| 1382019_at | Alg5 | asparagine-linked glycosylation 5, dolichyl-phosphate beta-glucosyltransferase homolog (S. cerevisiae) | 2.4437 | determination of left/right symmetry |
| 1379254_at | Tmem183a | transmembrane protein 183A | 2.4413 | --- |
| 1395316_at | Mageh1 | melanoma antigen, family H, 1 | 2.4307 | nerve growth factor receptor signaling pathway |
| 1389756_at | Melk | maternal embryonic leucine zipper kinase | 2.4276 | protein kinase activity |
| 1387186_at | Rab9a | RAB9A, member RAS oncogene family | 2.4162 | transport |
| 1399075_at | Map3k7 | Mitogen activated protein kinase kinase kinase 7 | 2.4038 | MAPKKK cascade |
| 1375852_at | Hmgcr | 3-hydroxy-3-methylglutaryl-Coenzyme A reductase | 2.3966 | steroid biosynthetic process |
| 1384161_at | Csnk1e | Casein kinase 1, epsilon | 2.392 | protein amino acid phosphorylation |
| 1388941_at | Amz2 | archaelysin family metallopeptidase 2 | 2.3904 | peptidase activity |
| 1370180_at | Nudt4 | nudix (nucleoside diphosphate linked moiety X)-type motif 4 | 2.3847 | phosphoinositide metabolic process |
| 1384435_at | Tmem68 | transmembrane protein 68 | 2.3823 | --- |
| 1371533_at | Dctn6 | dynactin 6 | 2.3803 | transferase activity |
| 1387769_a_at | Id3 | inhibitor of DNA binding 3 | 2.3788 | negative regulation of transcription from RNA polymerase II promoter |
| 1383522_at | Utx | ubiquitously transcribed tetratricopeptide repeat, X chromosome | 2.3663 | binding |
| 1368668_at | Plaa | phospholipase A2, activating protein | 2.352 | inflammatory response |
| 1398606_at | Golim4 | golgi integral membrane protein 4 | 2.3466 | transport |
| 1369950_at | Cdk4 | cyclin-dependent kinase 4 | 2.3458 | G1/S transition of mitotic cell cycle |
| 1386913_at | Pdpn | podoplanin | 2.3404 | cell morphogenesis |
| 1377194_a_at | Ccdc90b | coiled-coil domain containing 90B | 2.3357 | --- |
| 1367606_at | Rps3a | ribosomal protein S3a | 2.3336 | translation |
| 1371498_at | Jtv1 | JTV1 gene | 2.3333 | translation |
| 1371403_at | Cct3 | chaperonin containing Tcp1, subunit 3 (gamma) | 2.3244 | protein folding |
| 1399016_at | Myst2 | MYST histone acetyltransferase 2 | 2.3211 | DNA replication |
| 1367472_at | Uba1 | ubiquitin-like modifier activating enzyme 1 | 2.317 | protein modification process |
| 1371484_at | LOC690349 | hypothetical protein LOC690349 | 2.3055 | --- |
| 1373100_at | LOC499779 | similar to RIKEN cDNA 2900010J23 | 2.3044 | --- |
| 1372363_at | Polr2h /// RGD1565904 | polymerase (RNA) II (DNA directed) polypeptide H /// similar to polymerase (RNA) II (DNA directed) polypeptide H | 2.2977 | nuclear mRNA splicing, via spliceosome |
| 1377103_at | Midn | midnolin | 2.296 | --- |
| 1376951_at | Mad2l1 | MAD2 (mitotic arrest deficient, homolog)-like 1 (yeast) | 2.2948 | mitotic sister chromatid segregation |
| 1390447_at | Stx3 | syntaxin 3 | 2.2896 | transport |
| 1378124_at | Ppm1b | protein phosphatase 1B, magnesium dependent, beta isoform | 2.2869 | protein amino acid dephosphorylation |
| 1372122_at | Tsg101 | tumor susceptibility gene 101 | 2.2867 | regulation of cell growth |
| 1388979_at | Smndc1 | survival motor neuron domain containing 1 | 2.2793 | spliceosome assembly |
| 1386881_at | Igfbp3 | insulin-like growth factor binding protein 3 | 2.2773 | regulation of cell growth |
| 1372156_at | Tmem97 | transmembrane protein 97 | 2.2699 | --- |
| 1398405_at | Sept6 | septin 6 | 2.2675 | cell cycle |
| 1377935_at | Cldnd1 | Claudin domain containing 1 | 2.2565 | --- |
| 1371696_at | Gpr56 | G protein-coupled receptor 56 | 2.2564 | cell adhesion |
| 1371656_at | Cct4 | chaperonin containing Tcp1, subunit 4 (delta) | 2.2556 | protein folding |
| 1391743_at | Elavl1 | ELAV (embryonic lethal, abnormal vision, Drosophila)-like 1 (Hu antigen R) | 2.253 | mRNA stabilization |
| 1398934_at | Map3k7ip2 | mitogen-activated protein kinase kinase kinase 7 interacting protein 2 | 2.252 | positive regulation of I-kappaB kinase/NF-kappaB cascade |
| 1389381_at | Sqstm1 | sequestosome 1 | 2.2461 | positive regulation of protein amino acid phosphorylation |
| 1392629_a_at | Zcrb1 | zinc finger CCHC-type and RNA binding motif 1 | 2.2458 | mRNA processing |
| 1386872_at | Igf2r | insulin-like growth factor 2 receptor | 2.2447 | liver development |
| 1371876_at | Psmg2 | proteasome (prosome, macropain) assembly chaperone 2 | 2.2433 | apoptosis |
| 1373870_at | Fam98a | family with sequence similarity 98, member A | 2.2339 | --- |
| 1367766_at | Nme2 | non-metastatic cells 2, protein (NM23B) expressed in | 2.2229 | negative regulation of myeloid leukocyte differentiation |
| 1372439_at | Col4a1 | collagen, type IV, alpha 1 | 2.2135 | epithelial cell differentiation |
| 1398759_at | Tsc22d1 | TSC22 domain family, member 1 | 2.2026 | transcription |
| 1373824_at | Cfdp1 | craniofacial development protein 1 | 2.194 | anti-apoptosis |
| 1371662_at | Kars | lysyl-tRNA synthetase | 2.1934 | translation |
| 1389248_at | Galk1 | galactokinase 1 | 2.1868 | galactose metabolic process |
| 1370859_at | Pdia6 | protein disulfide isomerase family A, member 6 | 2.186 | protein folding |
| 1371480_at | RGD1561797 | RGD1561797 | 2.1804 | cell cycle |
| 1389521_at | Ivns1abp | influenza virus NS1A binding protein | 2.1766 | protein binding |
| 1371461_at | Fam54b | family with sequence similarity 54, member B | 2.1692 | --- |
| 1392930_at | Armc1 | armadillo repeat containing 1 | 2.1661 | metal ion transport |
| 1389868_at | RGD1564560 | similar to RCK | 2.151 | nucleic acid binding |
| 1380235_at | Hs2st1 | heparan sulfate 2-O-sulfotransferase 1 | 2.149 | heparan sulfate proteoglycan biosynthetic process, polysaccharide chain biosynthetic process |
| 1372155_at | Trim28 | tripartite motif-containing 28 | 2.1465 | negative regulation of transcription from RNA polymerase II promoter |
| 1388568_at | Eif3d | eukaryotic translation initiation factor 3, subunit D | 2.1457 | formation of translation initiation complex |
| 1388730_at | Cdc42ep4 | CDC42 effector protein (Rho GTPase binding) 4 | 2.1442 | Rho protein signal transduction |
| 1367662_at | Hsd17b10 | hydroxysteroid (17-beta) dehydrogenase 10 | 2.144 | cell aging |
| 1370536_at | Prmt3 | protein arginine methyltransferase 3 | 2.1412 | protein amino acid methylation |
| 1387670_at | Gpd2 | glycerol-3-phosphate dehydrogenase 2, mitochondrial | 2.1267 | glycerol-3-phosphate metabolic process |
| 1387884_at | Psma5 | proteasome (prosome, macropain) subunit, alpha type 5 | 2.1221 | ubiquitin-dependent protein catabolic process |
| 1392885_at | Mbd1 | methyl-CpG binding domain protein 1 | 2.1202 | DNA binding |
| 1389184_at | Rpp30 | ribonuclease P/MRP 30 subunit (human) | 2.1175 | tRNA processing |
| 1367831_at | Tp53 | tumor protein p53 | 2.1165 | protein import into nucleus, translocation |
| 1387797_at | Rab7a | RAB7A, member RAS oncogene family | 2.1125 | transport |
| 1373955_at | Ipo5 | importin 5 | 2.1063 | intracellular protein transport |
| 1367698_a_at | Sept9 | septin 9 | 2.1059 | DNA topological change |
| 1388830_at | Pkn2 | protein kinase N2 | 2.1041 | protein amino acid phosphorylation |
| 1374516_at | Chtf8 | CTF8, chromosome transmission fidelity factor 8 homolog (S. cerevisiae) | 2.1014 | DNA replication |
| 1389576_at | Snrpb2 | small nuclear ribonucleoprotein polypeptide B'' | 2.1003 | nuclear mRNA splicing, via spliceosome |
| 1398890_at | MGC93975 | similar to 2310044H10Rik protein | 2.0977 | --- |
| 1387975_at | Ugcg | UDP-glucose ceramide glucosyltransferase | 2.0975 | lipid metabolic process |
| 1379645_at | Pbrm1 | polybromo 1 | 2.0965 | placenta development |
| 1367900_at | Gyg1 | glycogenin 1 | 2.0948 | glycogen biosynthetic process |
| 1372715_at | Sfxn1 | sideroflexin 1 | 2.0947 | transport |
| 1372364_a_at | Ntan1 | N-terminal asparagine amidase | 2.0945 | memory |
| 1391453_at | Ebi3 | Epstein-Barr virus induced 3 | 2.0887 | cytokine receptor activity |
| 1367505_at | Znf644 | zinc finger protein 644 | 2.0884 | zinc ion binding |
| 1391475_at | Hnrpll | heterogeneous nuclear ribonucleoprotein L-like | 2.082 | mRNA processing |
| 1367776_at | Cdc2 | cell division cycle 2, G1 to S and G2 to M | 2.0803 | G1 phase of mitotic cell cycle |
| 1386985_at | Gstm1 | glutathione S-transferase mu 1 | 2.0783 | glutathione metabolic process |
| 1367837_at | Psma4 | proteasome (prosome, macropain) subunit, alpha type 4 | 2.0768 | ubiquitin-dependent protein catabolic process |
| 1367818_at | Coq3 | coenzyme Q3 homolog, methyltransferase (S. cerevisiae) | 2.0642 | glycerol metabolic process |
| 1377341_at | RGD1305455 | similar to hypothetical protein FLJ10925 | 2.0634 | --- |
| 1382154_at | Ptpn12 | protein tyrosine phosphatase, non-receptor type 12 | 2.058 | protein amino acid dephosphorylation |
| 1388550_at | Rad23b | RAD23 homolog B (S. cerevisiae) | 2.0565 | nucleotide-excision repair, DNA damage removal |
| 1385487_at | Lsm1 | LSM1 homolog, U6 small nuclear RNA associated (S. cerevisiae) | 2.056 | protein binding |
| 1371830_at | Sae1 | SUMO1 activating enzyme subunit 1 | 2.0549 | protein modification process |
| 1369992_at | Psmd1 | proteasome (prosome, macropain) 26S subunit, non-ATPase, 1 | 2.0534 | anaphase-promoting complex-dependent proteasomal ubiquitin-dependent protein catabolic process |
| 1374036_at | Mcm2 | minichromosome maintenance complex component 2 | 2.0525 | DNA replication |
| 1371042_at | Map4k3 | mitogen-activated protein kinase kinase kinase kinase 3 | 2.0458 | protein amino acid phosphorylation |
| 1372255_at | Rars | arginyl-tRNA synthetase | 2.0443 | translation |
| 1388723_at | Bre | brain and reproductive organ-expressed protein | 2.0428 | DNA repair |
| 1367575_at | Eno1 | enolase 1, (alpha) | 2.0424 | glycolysis |
| 1373907_at | Trappc4 | trafficking protein particle complex 4 | 2.0419 | transport |
| 1373388_at | Sppl3 | signal peptide peptidase 3 | 2.0401 | aspartic-type endopeptidase activity |
| 1388819_at | Scamp1 | secretory carrier membrane protein 1 | 2.0356 | transport |
| 1398800_at | Ywhab | tyrosine 3-monooxygenase/tryptophan 5-monooxygenase activation protein, beta polypeptide | 2.0295 | protein targeting |
| 1383502_at | Topbp1 | topoisomerase (DNA) II binding protein 1 | 2.0278 | response to DNA damage stimulus |
| 1369638_at | Eef2k | eukaryotic elongation factor-2 kinase | 2.0269 | protein amino acid phosphorylation |
| 1371908_at | Nxt1 | NTF2-like export factor 1 | 2.0257 | RNA export from nucleus |
| 1386875_a_at | Clta | clathrin, light chain (Lca) | 2.0214 | intracellular protein transport |
| 1371365_at | Ube2s | ubiquitin-conjugating enzyme E2S | 2.0207 | modification-dependent protein catabolic process |
| 1379651_at | Foxp1 | Forkhead box P1 | 2.0145 | negative regulation of transcription from RNA polymerase II promoter |
| 1398932_at | Hint1 | histidine triad nucleotide binding protein 1 | 2.0133 | intracellular protein transport |
| 1371923_at | Lpcat1 | lysophosphatidylcholine acyltransferase 1 | 2.0101 | metabolic process |
| 1370826_at | Nap1l1 | nucleosome assembly protein 1-like 1 | 2.0051 | nucleosome assembly |
| 1387300_at | Crnkl1 | crooked neck pre-mRNA splicing factor-like 1 (Drosophila) | 2.0043 | spliceosome assembly |

|  |  | **All Stem cell specific genes in RMC** |  |  |
| --- | --- | --- | --- | --- |
| **Probe Set ID** | **Gene Symbol** | **Gene Title** | **Fold Change** | **Function** |
| 1372195_at | Tnnc2 | troponin C type 2 (fast) | 8.6024 | skeletal muscle contraction |
| 1375813_at | Akap8l | A kinase (PRKA) anchor protein 8-like | 5.6932 | DNA binding |
| 1378864_at | Lrrc23 | leucine rich repeat containing 23 | 5.1395 | protein binding |
| 1389874_at | Zrsr1 | zinc finger (CCCH type), RNA binding motif and serine/arginine rich 1 | 5.0348 | nucleotide binding |
| 1387117_at | Zranb2 | zinc finger, RAN-binding domain containing 2 | 4.598 | mRNA processing |
| 1370412_at | Tnnt1 | troponin T type 1 (skeletal, slow) | 4.4062 | skeletal muscle contraction |
| 1379691_at | Prpf38b | PRP38 pre-mRNA processing factor 38 (yeast) domain containing B | 4.3721 | mRNA processing |
| 1370815_at | Nefh | neurofilament, heavy polypeptide | 4.2066 | microtubule cytoskeleton organization |
| 1386931_at | Tnni3 | troponin I type 3 (cardiac) | 4.0048 | vasculogenesis |
| 1375213_at | Pck2 | phosphoenolpyruvate carboxykinase 2 (mitochondrial) | 3.8945 | gluconeogenesis |
| 1376102_at | Tmbim1 | transmembrane BAX inhibitor motif containing 1 | 3.7938 | --- |
| 1387010_s_at | Scn1b | sodium channel, voltage-gated, type I, beta | 3.6699 | transport |
| 1385074_at | Smarca2 | SWI/SNF related, matrix associated, actin dependent regulator of chromatin, subfamily a, member 2 | 3.663 | negative regulation of cell proliferation |
| 1379046_at | Mlec | malectin | 3.5937 | carbohydrate metabolic process |
| 1387916_at | Cyp4f6 | cytochrome P450 4F6 | 3.4649 | leukotriene metabolic process |
| 1398354_at | Ctnnal1 | catenin (cadherin associated protein), alpha-like 1 | 3.4127 | --- |
| 1374061_at | Cd302 | CD302 molecule | 3.3742 | binding |
| 1382056_at | Sfrs11 | splicing factor, arginine/serine-rich 11 | 3.3325 | nuclear mRNA splicing, via spliceosome |
| 1392916_at | Map7 | microtubule-associated protein 7 | 3.263 | cell morphogenesis |
| 1368321_at | Egr1 | early growth response 1 | 3.2473 | negative regulation of transcription from RNA polymerase II promoter |
| 1377124_at | Mtf1 | metal-regulatory transcription factor 1 | 3.2416 | regulation of transcription, DNA-dependent |
| 1372531_at | Ppfibp2 | PTPRF interacting protein, binding protein 2 (liprin beta 2) | 3.2355 | DNA integration |
| 1388740_at | Fermt3 | fermitin family homolog 3 (Drosophila) | 3.0629 | protein binding |
| 1395419_at | Mll1 | myeloid/lymphoid or mixed-lineage leukemia 1 | 2.9906 | DNA repair |
| 1379715_at | RGD1307882 | similar to CG9346-PA | 2.9758 | RNA processing |
| 1392561_at | Atp5c1 | ATP synthase, H+ transporting, mitochondrial F1 complex, gamma polypeptide 1 | 2.9722 | oxidative phosphorylation |
| 1387874_at | Dbp | D site of albumin promoter (albumin D-box) binding protein | 2.9682 | transcription |
| 1367570_at | Tagln | transgelin | 2.9554 | cytoskeleton organization |
| 1373654_at | Anxa8 | annexin A8 | 2.9225 | blood coagulation |
| 1398297_at | Mapk12 | mitogen-activated protein kinase 12 | 2.9091 | glycogen biosynthetic process |
| 1387834_at | Matk | megakaryocyte-associated tyrosine kinase | 2.8995 | protein amino acid phosphorylation |
| 1387908_at | Rasd1 | RAS, dexamethasone-induced 1 | 2.8931 | signal transduction |
| 1391995_at | Trim37 | tripartite motif-containing 37 | 2.8666 | protein binding |
| 1385043_at | Inadl | InaD-like (Drosophila) | 2.8349 | protein binding |
| 1371959_at | LOC690131 | similar to H2A histone family, member O | 2.8217 | nucleosome assembly |
| 1388710_at | Rreb1 | ras responsive element binding protein 1 | 2.8181 | nucleic acid binding |
| 1389774_at | Znf23 | zinc finger protein 23 (KOX 16) | 2.806 | regulation of transcription, DNA-dependent |
| 1374725_at | Mov10 | Moloney leukemia virus 10 | 2.7372 | --- |
| 1380989_at | Numa1 | nuclear mitotic apparatus protein 1 | 2.7358 | signal transduction |
| 1392965_a_at | Smoc2 | SPARC related modular calcium binding 2 | 2.7352 | positive regulation of cell-substrate adhesion |
| 1395237_at | Eif5b | eukaryotic translation initiation factor 5B | 2.7087 | translation |
| 1389515_at | Rfxank | regulatory factor X-associated ankyrin-containing protein | 2.6709 | Ras protein signal transduction |
| 1398318_at | Muc1 | mucin 1, cell surface associated | 2.6672 | female pregnancy |
| 1374117_at | Baiap2 | BAI1-associated protein 2 | 2.6217 | signal transduction |
| 1385163_at | Rnf20 | ring finger protein 20 | 2.6209 | protein polyubiquitination |
| 1389339_at | Arsa | Arylsulfatase A | 2.6182 | autophagy |
| 1382055_at | Rtkn | rhotekin | 2.6178 | apoptosis |
| 1375128_at | Mtdh | Metadherin | 2.6164 | negative regulation of transcription from RNA polymerase II promoter |
| 1372232_at | Wbp1 | WW domain binding protein 1 | 2.5074 | protein binding |
| 1371997_at | Akr1cl2 | aldo-keto reductase family 1, member C-like 2 | 2.4959 | oxidation reduction |
| 1385779_at | Chd4 | chromodomain helicase DNA binding protein 4 | 2.4932 | chromatin assembly or disassembly |
| 1376799_a_at | Crlf1 | cytokine receptor-like factor 1 | 2.4905 | cytokine binding |
| 1373750_at | Leprel2 | leprecan-like 2 | 2.4659 | oxidation reduction |
| 1398663_at | Zfp61 | zinc finger protein 61 | 2.4437 | transcription |
| 1381339_at | Eif3k | eukaryotic translation initiation factor 3, subunit K | 2.4413 | regulation of translational initiation |
| 1381120_at | Invs | inversin | 2.4202 | kidney development |
| 1374621_at | Taf1c | TATA box binding protein (Tbp)-associated factor, RNA polymerase I, C | 2.3723 | transcription |
| 1384182_at | Fermt2 | fermitin family homolog 2 (Drosophila) | 2.3256 | --- |
| 1388539_at | Pkp2 | plakophilin 2 | 2.3106 | heart development |
| 1380577_at | Abcg2 | ATP-binding cassette, sub-family G (WHITE), member 2 | 2.3062 | transport |
| 1377241_at | Znf503 | zinc finger protein 503 | 2.3051 | zinc ion binding |
| 1379633_a_at | Uba7 | ubiquitin-like modifier activating enzyme 7 | 2.2975 | protein modification process |
| 1374694_at | Ankrd28 | ankyrin repeat domain 28 | 2.2893 | protein binding |
| 1389778_a_at | Tceb3 | transcription elongation factor B (SIII), polypeptide 3 | 2.2887 | transcription |
| 1387788_at | Junb | jun B proto-oncogene | 2.2878 | vasculogenesis |
| 1387252_at | Sec14l2 | SEC14-like 2 (S. cerevisiae) | 2.2858 | transcription |
| 1389620_at | Suv420h2 | suppressor of variegation 4-20 homolog 2 (Drosophila) | 2.2717 | transcription |
| 1389002_at | Tln1 | talin 1 | 2.2627 | cell-substrate junction assembly |
| 1389306_at | Matn2 | matrilin 2 | 2.2574 | zinc ion binding |
| 1368054_at | Lmna | lamin A | 2.2565 | nuclear envelope organization |
| 1374855_at | Per1 | period homolog 1 (Drosophila) | 2.2415 | transcription |
| 1371664_at | Pxn | paxillin | 2.2373 | cell adhesion |
| 1375478_at | Zfp362 /// Zscan20 | zinc finger protein 362 /// zinc finger and SCAN domain containing 20 | 2.232 | regulation of transcription, DNA-dependent |
| 1392173_at | Zcchc3 | zinc finger, CCHC domain containing 3 | 2.2272 | nucleic acid binding |
| 1373760_at | Tars2 | threonyl-tRNA synthetase 2, mitochondrial (putative) | 2.2069 | translation |
| 1371104_at | Srebf1 | sterol regulatory element binding transcription factor 1 | 2.2028 | transcription |
| 1369984_at | Cox17 | cytochrome c oxidase, subunit XVII assembly protein homolog (S. cerevisiae) | 2.1992 | copper ion transport |
| 1367612_at | Mgst1 | microsomal glutathione S-transferase 1 | 2.1862 | glutathione metabolic process |
| 1375613_at | Gata6 | GATA binding protein 6 | 2.1637 | in utero embryonic development |
| 1368772_at | Slc4a3 | solute carrier family 4 (anion exchanger), member 3 | 2.1577 | transport |
| 1369590_a_at | Ddit3 | DNA-damage inducible transcript 3 | 2.1394 | response to amphetamine |
| 1374653_at | Fam73b | family with sequence similarity 73, member B | 2.1393 | --- |
| 1390305_at | Prkcbp1 | protein kinase C binding protein 1 | 2.1132 | protein binding |
| 1395767_at | Cstf1 | cleavage stimulation factor, 3' pre-RNA, subunit 1 | 2.1104 | nuclear mRNA splicing, via spliceosome |
| 1375058_at | Zrsr2 | zinc finger (CCCH type), RNA binding motif and serine/arginine rich 2 | 2.1065 | nucleotide binding |
| 1393067_at | Tek | TEK tyrosine kinase, endothelial | 2.1025 | angiogenesis |
| 1375177_at | Klf13 | Kruppel-like factor 13 | 2.1024 | positive regulation of transcription from RNA polymerase II promoter |
| 1397607_at | RGD1310862 | similar to adult retina protein | 2.1012 | regulation of transcription, DNA-dependent |
| 1394292_at | Mrpl37 | mitochondrial ribosomal protein L37 | 2.0871 | --- |
| 1376176_at | Mast3 | microtubule associated serine/threonine kinase 3 | 2.0802 | protein amino acid phosphorylation |
| 1375351_at | Ssbp4 | single stranded DNA binding protein 4 | 2.08 | DNA binding |
| 1375134_at | Dda1 | DET1 and DDB1 associated 1 | 2.0796 | --- |
| 1393155_at | Plk3 | polo-like kinase 3 (Drosophila) | 2.079 | protein amino acid phosphorylation |
| 1386998_at | Aldoc | aldolase C, fructose-bisphosphate | 2.077 | response to hypoxia |
| 1387776_at | Tgm2 | transglutaminase 2, C polypeptide | 2.0755 | blood vessel remodeling |
| 1380502_at | Zic2 | Zic family member 2 (odd-paired homolog, Drosophila) | 2.071 | neural tube closure |
| 1371560_at | Irf3 | interferon regulatory factor 3 | 2.0647 | transcription |
| 1368749_at | Kcns1 | potassium voltage-gated channel, delayed-rectifier, subfamily S, member 1 | 2.0605 | transport |
| 1368235_at | Clk3 | CDC-like kinase 3 | 2.0597 | protein amino acid phosphorylation |
| 1396820_at | Hdac1 | histone deacetylase 1 | 2.0538 | transcription |
| 1373050_at | Tbc1d1 | TBC1 domain family, member 1 | 2.05 | regulation of protein localization |
| 1370209_at | Klf9 | Kruppel-like factor 9 | 2.0462 | transcription |
| 1386935_at | Nr4a1 | nuclear receptor subfamily 4, group A, member 1 | 2.046 | transcription |
| 1370820_at | Fbxo6 | F-box protein 6 | 2.045 | glycoprotein catabolic process |
| 1397946_at | Sec14l1 | SEC14-like 1 (S. cerevisiae) | 2.0441 | --- |
| 1368072_at | Btg3 | B-cell translocation gene 3 | 2.0372 | response to oxidative stress |
| 1377484_at | Fbxl3 | F-box and leucine-rich repeat protein 3 | 2.0351 | protein binding |
| 1369329_at | Notch3 | Notch homolog 3 (Drosophila) | 2.0324 | transcription |
| 1384337_x_at | Cln3 | ceroid-lipofuscinosis, neuronal 3 | 2.0291 | autophagic vacuole fusion |
| 1389120_at | Kcnc3 | potassium voltage gated channel, Shaw-related subfamily, member 3 | 2.0057 | transport |
| 1373503_at | Lrch4 | leucine-rich repeats and calponin homology (CH) domain containing 4 | 2.0036 | negative regulation of transcription |
| 1381012_at | Serpinf1 | serine (or cysteine) peptidase inhibitor, clade F, member 1 | 2.001 | kidney development |
