# Supplementary material for: Transcriptome analysis of amoeboid and ramified microglia isolated from the corpus callosum of rat brain
Source: BMC Neurosci. 2012 Jun 14;13:64. doi: 10.1186/1471-2202-13-64 (PMC3441342; doi:10.1186/1471-2202-13-64)
Supplement: Additional file 10 — Sheet S8. Expression of cytokines in AMC and RMC. [file 1471-2202-13-64-S10.docx]

| **Probe Set ID** | **Gene Symbol** | **Gene Title** | **Average AMC expression** | **Average RMC expression** |
| --- | --- | --- | --- | --- |
| 1369653_at | Tgfbr2 | transforming growth factor, beta receptor II | 2.433333333 | 10.6 |
| 1370790_at | Ifng | interferon gamma | 5.9 | 50.93333333 |
| 1388033_at | Cxcl3 | chemokine (C-X-C motif) ligand 3 | 6.233333333 | 54.33333333 |
| 1370082_at | Tgfb1 | transforming growth factor, beta 1 | 6.266666667 | 21.23333333 |
| 1369889_at | Ifnb1 | interferon beta 1, fibroblast | 6.833333333 | 81.83333333 |
| 1387691_at | Tnf | tumor necrosis factor (TNF superfamily, member 2) | 8.733333333 | 44.96666667 |
| 1370633_at | Cxcl3 | chemokine (C-X-C motif) ligand 3 | 8.733333333 | 19.6 |
| 1368800_at | Cd40lg | CD40 ligand | 9.266666667 | 26.4 |
| 1369255_at | Il1r1 | interleukin 1 receptor, type I | 11.33333333 | 54.06666667 |
| 1367973_at | Ccl2 | chemokine (C-C motif) ligand 2 | 12.36666667 | 38.3 |
| 1391384_at | Tnf | tumor necrosis factor (TNF superfamily, member 2) | 13.66666667 | 13.3 |
| 1387655_at | Cxcl12 | chemokine (C-X-C motif) ligand 12 (stromal cell-derived factor 1) | 15.1 | 102.3333333 |
| 1368657_at | Mmp3 | matrix metallopeptidase 3 | 15.36666667 | 60.4 |
| 1368134_a_at | Il4ra | interleukin 4 receptor, alpha | 19.2 | 128.2666667 |
| 1393182_at | Cxcl3 | chemokine (C-X-C motif) ligand 3 | 19.23333333 | 41.73333333 |
| 1369191_at | Il6 | interleukin 6 | 20.86666667 | 14.93333333 |
| 1370750_a_at | Il1r1 | interleukin 1 receptor, type I | 21.26666667 | 115.5 |
| 1369315_at | Il12a | interleukin 12a | 29.3 | 136.7333333 |
| 1388234_at | Ifna1 | interferon-alpha 1 | 30.2 | 81.86666667 |
| 1368490_at | Cd14 | CD14 molecule | 36.2 | 37.8 |
| 1368678_at | Bdnf | brain derived neurotrophic factor | 41.86666667 | 55.2 |
| 1369565_at | Il12b | interleukin 12b | 45 | 67.23333333 |
| 1369504_at | Tgfbr1 | transforming growth factor, beta receptor 1 | 45.16666667 | 82.6 |
| 1388032_a_at | Cxcl3 | chemokine (C-X-C motif) ligand 3 | 45.73333333 | 104.7 |
| 1369266_at | Il13ra2 | interleukin 13 receptor, alpha 2 | 49.4 | 93.56666667 |
| 1370634_x_at | Cxcl3 | chemokine (C-X-C motif) ligand 3 | 50.13333333 | 39.2 |
| 1369219_at | Tgfbr3 | transforming growth factor, beta receptor III | 52.86666667 | 173.1333333 |
| 1369983_at | Ccl5 | chemokine (C-C motif) ligand 5 | 53.63333333 | 87.33333333 |
| 1387484_at | Tgfbr3 | transforming growth factor, beta receptor III | 68.56666667 | 102.3 |
| 1398256_at | Il1b | interleukin 1 beta | 71 | 115.0666667 |
| 1368677_at | Bdnf | brain derived neurotrophic factor | 72.13333333 | 313 |
| 1370832_at | Ccl4 | chemokine (C-C motif) ligand 4 | 83.76666667 | 119.4 |
| 1369815_at | Ccl3 | chemokine (C-C motif) ligand 3 | 90.8 | 144.3333333 |
| 1388784_at | Csf1r | colony stimulating factor 1 receptor | 118.0333333 | 285.3 |
| 1369665_a_at | Il18 | interleukin 18 | 122.2333333 | 335.9666667 |
| 1377698_at | Cd40 | CD40 molecule, TNF receptor superfamily member 5 | 227.8333333 | 109.2 |
| 1388583_at | Cxcl12 | chemokine (C-X-C motif) ligand 12 (stromal cell-derived factor 1) | 330.4666667 | 425.4666667 |
| 1369956_at | Ifngr1 | interferon gamma receptor 1 | 994.7666667 | 566.4666667 |
| 1369633_at | Cxcl12 | chemokine (C-X-C motif) ligand 12 (stromal cell-derived factor 1) | 1035.133333 | 557.7666667 |
| 1376636_at | Tgfbr1 | transforming growth factor, beta receptor 1 | 1346.966667 | 996.2 |
